# Forcing the Side‐on π‐Coordination of a C≡C Triple Bond to Technetium Using the *As*,*CC*,*As* Alkyne Pincer Ligand 1,2‐Bis(2‐(diisopropylarsaneyl)‐4‐(trifluoromethyl)phenyl)Ethyne

**DOI:** 10.1002/anie.6878935

**Published:** 2026-05-02

**Authors:** Maximilian Roca Jungfer, Moritz Johannes Ernst, Lukas Eberle, Marius Kesselring, Guilhem Claude, Joachim Ballmann

**Affiliations:** ^1^ Institut Für Nukleare Entsorgung (INE) Karlsruher Institut für Technologie (KIT) Eggenstein‐Leopoldshafen Germany; ^2^ Fachbereich Biologie, Chemie, Pharmazie Institut Für Anorganische Chemie Freie Universität Berlin Berlin Germany; ^3^ Anorganisch Chemisches Institut Ruprecht‐Karls‐Universität Heidelberg Heidelberg Germany; ^4^ Corporate Member of Freie Universität Berlin and Humboldt Universität Zu Berlin Klinik für Nuklearmedizin/Radiopharmazie Charité – Universitätsmedizin Berlin Berlin Germany

**Keywords:** ^99m^Tc, alkyne, radioactive, side‐on, technetium

## Abstract

Alkyne complexes are known for transition metals across the d‐block with exception of the radioelement technetium despite considerable synthetic efforts. DFT calculations suggest that this is not inherent to the transition metal but a consequence of the overall ligand sphere. The arsenic‐based tolane ligand 1,2‐bis(2‐(diisopropylarsaneyl)‐4‐(trifluoromethyl)phenyl)ethyne (L*^i^* ^Pr^) forces a coordination of the central alkyne moiety onto the metal through ligand design. The stable, crystalline Tc(III) and Tc(V) alkyne complexes *mer*‐[Tc^III^Cl_3_(κ^4^‐*As*,*CC*,*As*‐L*^i^* ^Pr^)], *mer*‐[Tc^V^NX_2_(κ^4^‐*As*,*CC*,*As*‐L*^i^* ^Pr^)] (X = Cl, Br) and *cis,trans*,*mer*‐[Tc^V^N(CN)Cl(κ^4^‐*As*, *CC*, *As*‐L*^i^* ^Pr^)] alongside their rhenium homologs *mer*‐[Re^V^Cl_3_(κ^4^‐*As*,*CC*,*As*‐L*^i^* ^Pr^)] and *mer*‐[Re^V^NCl_2_(κ^4^‐*As*,*CC*,*As*‐L*^i^* ^Pr^)] have been prepared and fully characterized. According to spectroscopic and DFT analyses, the technetium complexes represent robust, classical 2e^−^ alkyne complexes, while a different situation was found for *mer*‐[Re^V^Cl_3_(κ^4^‐*As*,*CC*,*As*‐L*^i^* ^Pr^)] with a formally oxidized metal ion and reduced 4e^−^ donor ligand. This has general implications for π‐ligand coordination in group 7 and potentially for neighboring elements. Successful translation to the medicinally relevant nuclear isomer ^99m^Tc proves the viability of alkyne donors as building blocks for stable chelation of technetium at the tracer level.

## Graphical abstract

Technetium alkyne complexes have eluded isolation for decades with the only indicators for their existence being the isolation of vinylidene carbenes or their follow‐up reaction products despite plentiful of reports for neighboring elements. Stable, crystalline Tc(III) and Tc(V) alkyne complexes (and their Re analogs) have now been prepared and fully characterized enabled by a tolane‐based *As*,*CC*,*As* ligand containing two flanking arsine tethers. Surprising differences and similarities in reactivity and electronic structure between Tc compounds and the analogous Re complexes were found and investigated by extensive theoretical analyses. Tc has a lower tendency for adopting a metallacyclopropene resonance structure compared to rhenium rationalizing the difficulties previously observed for the isolation of Tc alkynes and providing a framework for future investigations of this previously inaccessible compound class. The stability of the technetium alkyne complexes allowed a ready translation to the medicinally relevant nuclear isomer ^99m^Tc, suggesting C≡C triple bonds as suitable building blocks for radiopharmaceutical applications.

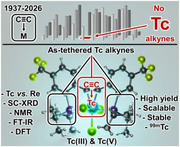

## 1 Introduction

**FIGURE 1:**
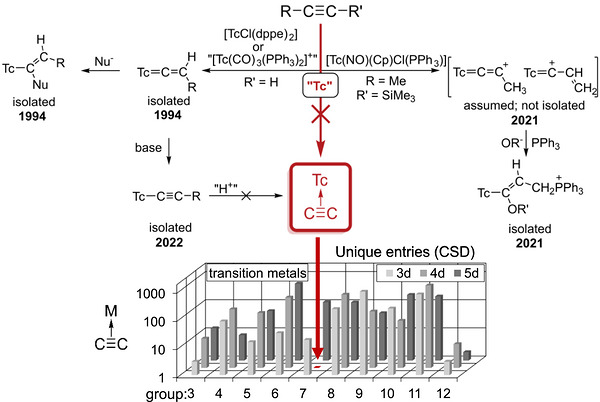
State of the field of technetium alkyne complexes [1, 2, 3, 4, 5, 6, 33, 34].

Since their discovery, alkyne complexes have been isolated for all but one element of the central d‐block: the artificial radioelement technetium [1, 2, 3, 4, 5, 6]. This is not only especially surprising given the abundance, ease of formation, and structural variety of alkyne complexes containing the heavier homolog of technetium, rhenium (examples in references) [7, 8, 9, 10, 11, 12, 13, 14, 15, 16, 17, 18, 19, 20, 21, 22, 23, 24, 25, 26, 27, 28, 29, 30, 31, 32], which often resembles technetium in its chemistry but also contrasts with the behavior of other neighboring elements in the periodic table (Figure 1 [1, 2, 3, 4, 5, 6, 33, 34], for additional information see S5.1, S5.11).

**FIGURE 2:**
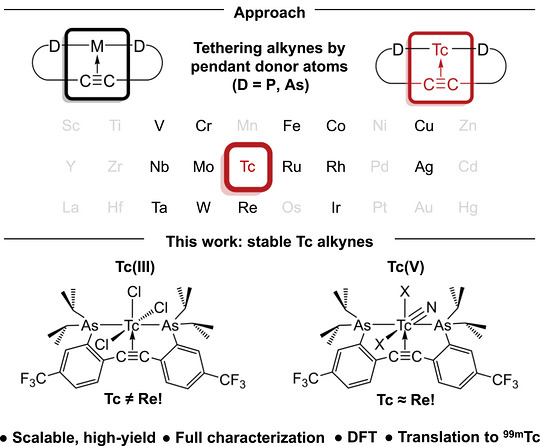
The approach used in this work based on previous literature precedent for *D*,*CC*,*D* (D = P, As) complexes across the periodic table [72, 73, 74, 75, 76, 77, 78, 79, 80, 81, 82, 83, 84, 85], and the scope of this work.

Probing the coordination of alkynes to technetium is therefore of fundamental interest to organometallic chemistry [1, 2, 3, 4, 5, 6], also considering that an π‐η^2^‐alkene and π‐η^2^‐nitrile have been isolated in traces inadvertently [35, 36], and some arene or cyclopentadienyl π‐complexes are known [1, 37, 38]. Plentiful attempts to bind alkyne ligands to technetium have been made during the past decades and indeed some reaction products requiring the intermediary coordination of an alkyne (followed by the immediate isomerization to a vinylidene carbene) but also an insertion product with a technetium hydride could be isolated [1, 2, 3, 4, 5, 6, 39]. Besides a fundamental interest, side‐on bonded alkyne ligands increase the number of donor atoms per formal vacant coordination site at the metal, which, if installed into the scaffold of a polydentate chelator, could lead to a significant stabilization of the resulting chelate complexes. Both organotechnetium and chelate complexes separately represent invaluable workhorses in radiopharmaceutical imaging using the short‐lived γ‐emitting nuclear technetium isomer ^99m^Tc (e.g., ^99m^Tc‐sestamibi or ^99m^Tc‐MAG3) [40, 41]. Also, other organotechnetium complexes such as those containing, e.g., {Tc^I^(CO)_3_}^+^ or Tc‐coordinating cyclopentadienyl ligands are recent, robust and versatile additions to the tool box of nuclear medicinal researchers [42, 43, 44, 45, 46, 47, 48, 49, 50, 51, 52, 53, 54, 55, 56, 57, 58, 59, 60, 61, 62, 63, 64, 65, 66, 67, 68, 69, 70, 71]. The introduction of extra donor atoms to a potential alkyne ligand may also favor the coordination of the alkyne moiety kinetically. Tolane‐based pincer ligands containing a central alkyne unit were reported to form complexes with the desired coordination mode for a number of elements in the periodic table (Figure 2; compiled in S5.2–S5.4) [72, 73, 74, 75, 76, 77, 78, 79, 80, 81, 82, 83, 84, 85].

To prevent a very common, and not yet fully understood [86], cyclization side‐reaction of such potential ligands, a fluorinated, arsenic‐based ligand was recently developed [84, 85]. The choice of the ligand substitution pattern for this study was a compromise of reactivity, stability, and solubility. Tentatively, As was chosen over P as the P derivatives are more electron‐rich and therefore more prone to cyclization. Similarly, the CF_3_ groups deplete the ligands electron density further, reducing the tendency for cyclization to pentalenes and stabilizing the ligands against oxidation of the ligand backbone, while simultaneously increasing the solubility of formed complexes. While *^t^* Bu substituents at the pnictogen atom usually stabilize the formed complexes by steric shielding of the metal, they hinder the kinetic introduction of the ligand and destabilize the ligand framework (at least for P derivatives) for cyclization. Hence, *^t^* Bu and *^i^* Pr *As*,*CC*,*As* ligands with CF_3_‐substitution at the flanks were chosen as the tentatively most promising candidates for the present work.

In this work, the first structurally authenticated complexes of technetium containing side‐on bonded alkyne ligands — not only with the long‐lived ^99^Tc but also with its medicinally relevant nuclear isomer ^99m^Tc — are reported. The coordinated alkyne is tethered by flanking dialkylarylarsine groups in an *As*,*CC*,*As* pincer ligand, forcing a π‐coordination of the alkyne to technetium.

## 2 Results and Discussion

To understand whether the endeavor for technetium alkyne complexes is even theoretically feasible, preceding density functional theory (DFT) calculations on the B3LYP‐D3/def2‐QZVPP level with an implicit solvation model for tetrahydrofuran were performed for hypothetical zero valent [M^0^···(HC≡CH)] (M = 3d, 4d, 5d metal) model complexes with a side‐on π‐coordinating alkyne and their [M^0^═C═CH_2_] vinylidene isomers for all d‐group elements (details are given as Supporting Information: S4.15). Both isomers were stable for technetium and rhenium and the bonding parameter trends for the group 7 elements fit well in between those observed for group 6 and group 8. Thus, it can be concluded that the experimentally observed, problematic isomerization of side‐on coordination of alkynes to vinylidene carbenes is not intrinsic but rather subject to influences of the accompanying ligand sphere around the metal. This was computationally confirmed by calculations on the B3LYP‐D3/def2‐QZVPP level for isostructural model complexes of groups 6, 7, and 8 with a [MNCl_2_(AsH_3_)_2_(C_2_H_2_)]^+/0/−^ (M = Cr, Mo, W, Mn, Tc, Re, Fe, Ru, Os) donor set (details are given as Supporting Information: S4.16). Although the nitrido complexes show a clear energetic preference of the vinylidene structure, a reasonable chance for the existence of technetium alkyne complexes under the correct circumstances was deduced as the differences were relatively small in the order of ca. 20 kJ/mol. The group 7 elements Mn, Tc, and Re feature stable complexes in over seven different oxidation states. Given that alkyne complexes can draw significant stabilization from backdonation of electron rich metal centers [87, 88], and that low‐valent rhenium alkynes appear to be somewhat more readily available [7, 8], low‐valent alkyne complexes were focused initially. First, experiments with a sterically encumbered and normally more robust *tert*‐butyl substituted potential *As*,*CC*,*As* ligand and [Tc^III^Cl_3_(PPh_3_)_2_(NCCH_3_)] led to cyclization of the arsine‐substituted alkyne as verified by x‐ray diffraction (see S2.8, S5.9) and intractable decomposition of the technetium starting material. Such reactions are frequently observed and often complex involving multi‐step ionic and radical pathways, thus, further analysis is beyond the scope of the present communication [86].

### 2.1 Technetium(III) Alkyne Complex

**SCHEME 1:**
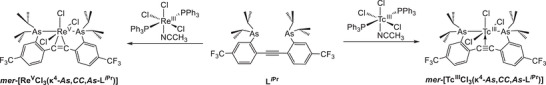
Reaction of L*^i^* ^Pr^ with isostructural starting materials [M^III^Cl_3_(PPh_3_)_2_(NCCH_3_)] (M = Tc, Re).

The fluorinated, *iso*‐propyl substituted ligand L*^i^* ^Pr^ instead reacted with both, the technetium(III) and rhenium(III) starting materials, [Tc^III^Cl_3_(PPh_3_)_2_(NCCH_3_)] and [Re^III^Cl_3_(PPh_3_)_2_(NCCH_3_)] giving the desired products [MCl_3_(κ^4^‐*As*,*CC*,*As*‐L*^i^* ^Pr^)] (Scheme 1).

**FIGURE 3:**
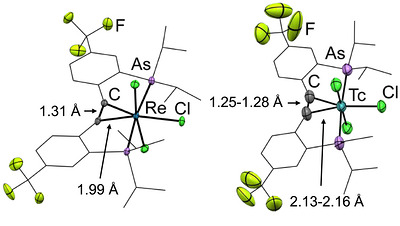
Molecular structures of the alkyne complexes *mer*‐[Re^V^Cl_3_(κ^4^‐*As*,*CC*,*As*‐L*^i^* ^Pr^)] and *mer*‐[Tc^III^Cl_3_(κ^4^‐*As*,*CC*,*As*‐L*^i^* ^Pr^)] (bond parameters compiled in Table 1). Ellipsoids are shown at 50% probability. Hydrogen atoms, disorder of the freely rotating CF_3_ groups and further labels are omitted for clarity. Skeletal carbon atoms are shown as wireframe for clarity.

While the ligand exchange reactions with L*^i^* ^Pr^ are robust and not directly sensitive to ambient oxygen or water, inadvertent oxidation of the released PPh_3_ must be avoided for isolation of the target products in high yield and purity due to co‐crystallization of OPPh_3_ (crystal quality consistently low; see S2.4, S5.5). The rhenium‐containing product was isolated as a diamagnetic green–gray powder, while the technetium‐containing product was isolated as paramagnetic deep red microcrystals (*µ*_eff_(CD_2_Cl_2_) ≈ 2.3). As expected for the octahedral d^4^ system containing Tc(III), the ^1^H NMR spectrum of this product is not instructive. The ^19^F{^1^H} NMR spectrum on the other hand shows only a single, narrow resonance for the CF_3_ groups at ca. −20 ppm being indicative of a symmetric ligand arrangement, however, ambiguity remains due to the clear influence of electron spin density in proximity to the CF_3_ groups. In contrast, a symmetric arrangement of the ligand in the diamagnetic rhenium complex can be concluded unambiguously from the observation of two sets of NMR resonances for the methyl groups of the *iso*‐propyl substituents (*syn*/*anti* to the aromatic ring). The ^13^C NMR resonance of the alkyne carbon atoms in the rhenium complex are shifted by almost 90–174 ppm from 95 ppm observed for the same carbon atoms in the free ligand [85], suggesting a considerable contribution of a rhenacyclopropene resonance structure [72, 73, 77, 89, 90, 91, 92, 93]. Consistent with the spectroscopic properties, the rhenium complex could tentatively be formulated as *mer*‐[Re^V^Cl_3_(κ^4^‐*As*,*CC*,*As*‐L*^i^* ^Pr^)] with a dianionic 4e^−^ donor alkyne unit and a d^2^ metal ion. Contrastingly for technetium, a classical side‐on alkyne complex, *mer*‐[Tc^III^Cl_3_(κ^4^‐*As*,*CC*,*As*‐L*^i^* ^Pr^)], with the alkyne acting as a 2e^−^ π‐donor was derived. A similar electronic situation had been observed for non‐fluorinated *D*,*CC*,*D* (D = P, As) rhenium complexes before [73]. However, this remarkable difference in electronic structure between the isostructural complexes of technetium and rhenium warranted a more in‐depth analysis and, hence, dark red single crystals of the technetium compound as well as dark green single crystals of the rhenium complex were grown by slow evaporation of CH_2_Cl_2_ solutions and analyzed by x‐ray diffraction. The solid‐state structures of both products are shown in Figure 3.

**TABLE 1:** Selected bond lengths and angles in the {MCl_3_}‐containing complexes of L*^i^* ^Pr^. The second set of parameters for *mer*‐[Tc^III^Cl_3_(κ^4^‐*As*,*CC*,*As*‐L*^i^* ^Pr^)]·0.5CH_2_Cl_2_ are those of a second independent molecule in the asymmetric unit.

Bond lengths [Å]	*mer*‐[Re^V^Cl_3_(κ^4^‐*As*,*CC*,*As*‐L*^i^* ^Pr^)]	*mer*‐[Tc^III^Cl_3_(κ^4^‐*As*,*CC*,*As*‐L*^i^* ^Pr^)]·0.5CH_2_Cl_2_
C≡C	1.309(4)	1.25(2)|1.28(2)
M‐C_C≡C_	1.986(2) 1.994(3)	2.13(1)|2.13(1) 2.13(1)|2.16(1)
M···centroid(C≡C)	1.879	2.037|2.048
M‐Cl_trans‐C≡C_	2.4399(7)	2.423(3)|2.433(3)
M‐Cl_trans‐Cl_	2.3878(7) 2.3884(8)	2.343(4)|2.338(3) 2.365(4)|2.358(3)
M‐As	2.5512(7) 2.5515(7)	2.572(1)|2.564(1) 2.572(1)|2565(2)
Angles [°]		
As‐M···centroid(C≡C)	93.8 93.8	92.4|92.5 92.8|93.2
Cl‐M···centroid(C≡C)	94.7 94.9 179.1	89.1|89.0 89.4|89.2 179.3|178.8
Ar‐C(≡CR)‐M	136.4(2) 136.7(2)	129.7(9)|128.8(8) 131.1(9)|130.2(8)
Ar‐C≡C	152.3(3) 152.8(3)	155(1)|156(1) 158(1)|159(1)
As‐M‐As	172.37(2)	174.61(9)|172.79(5)
Cl‐M‐Cl	84.49(3) 85.94(3) 170.42(3)	90.0(1)|89.6(1) 91.6(1)|92.1(1) 177.3(1)|177.8(1)

**FIGURE 4:**
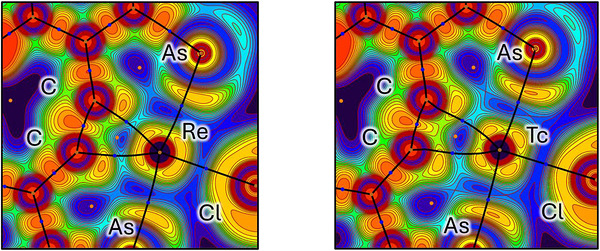
DFT‐based Electron localization function (ELF) maps of *mer*‐[Re^V^Cl_3_(κ [4]‐*As*,*CC*,*As*‐L*^i^* ^Pr^)] and *mer*‐[Tc^III^Cl_3_(κ [4]‐*As*,*CC*,*As*‐L*^i^* ^Pr^)] including topological features of the electron density (brown: (3,‐3) critical points; blue: (3,‐1) critical points; orange: (3,+1) critical points; black: bond paths connecting (3,‐3) and (3,‐1) critical points) of *ρ*(*r*) in the M···C≡C plane.

The significant elongation of the C≡C bond and contraction of the alkyne distance to rhenium (1.31 Å, 1.88 Å) in *mer*‐[Re^V^Cl_3_(κ^4^‐*As*,*CC*,*As*‐L*^i^* ^Pr^)] compared to the contracted C≡C bond and elongation of the alkyne distance to technetium (< 1.25 Å, > 2.00 Å) in *mer*‐[Tc^III^Cl_3_(κ^4^‐*As*,*CC*,*As*‐L*^i^* ^Pr^)] is a clear indicator of the different bonding situations in the structurally similar complexes. In line with this interpretation, the Ar─C≡C angles are smaller for the rhenium compound compared to its technetium counterpart. DFT calculations on the B3LYP‐D3/def2‐TZVP (d^2^: s, d^4^‐ls: t, d^4^‐hs: quint) revealed a low energy singlet Re^V^ (d^2^) configuration (d^4^‐ls: +3 kJ/mol, d^4^‐hs: +222 kJ/mol) consistent with the diamagnetism of the isolated complex matching the experimental geometry (exp.: Re‐C ≈ 2.00 Å; calc.: d^2^ ≈ 2.00 Å; d^4^‐ls ≈ 2.13 Å; d^4^‐hs ≈ 2.16 Å). Contrastingly and in accordance with the experimental observations, a paramagnetic d^4^‐ls (Tc^III^) configuration with two unpaired electrons is energetically preferred for technetium (d^2^: +24 kJ/mol, d^4^‐hs: +222 kJ/mol) and consistent with the observed x‐ray structure (exp.: Tc‐C ≈ 2.15 Å; calc.: d^2^ ≈ 2.00 Å; d^4^‐ls ≈ 2.15 Å; d^4^‐hs ≈ 2.20 Å). In the hypothetical (see S5.7) MnCl_3_ analogs, the d^4^‐hs configuration featuring an uncoordinated C≡C was most stable (d^2^: +245 kJ/mol; d^4^‐ls: +96 kJ/mol; details are provided as Supporting Information: S4.1) suggesting that the metallacyclopropene resonance structure contributes less when moving up the group 7 elements due to gradually increased spin‐pairing when moving down the group. Previously reported topological analyses of experimentally determined electron densities of 2e^−^ and 4e^−^ alkynes proved that a convex distortion of the bond paths is observed for 4e^−^ alkyne donors, while a concave shape is characteristic of 2e^−^ alkyne ligands [93]. An analogous topological analysis of the calculated electron density *ρ*(*r*) in the present case revealed a concave shape of the bond paths connecting the Tc‐C (3,‐1) critical points (“bond”) with the (3,‐3) critical points (atomic positions) for the most stable electron configuration of *mer*‐[Tc^III^Cl_3_(κ^4^‐*As*,*CC*,*As*‐L*^i^* ^Pr^)], consistent with the presumed 2e^−^ donor mode of the central alkyne unit (details S4.1). Contrastingly and characteristic for the presumed 4e^−^ donor situation, convex bond paths are obtained for the {ReCl_3_} analog. The topological features are depicted on electron localization function (ELF) mappings in the M···C·C plane in Figure 4 (complementary plots: S4.2–S4.14).

**FIGURE 5:**
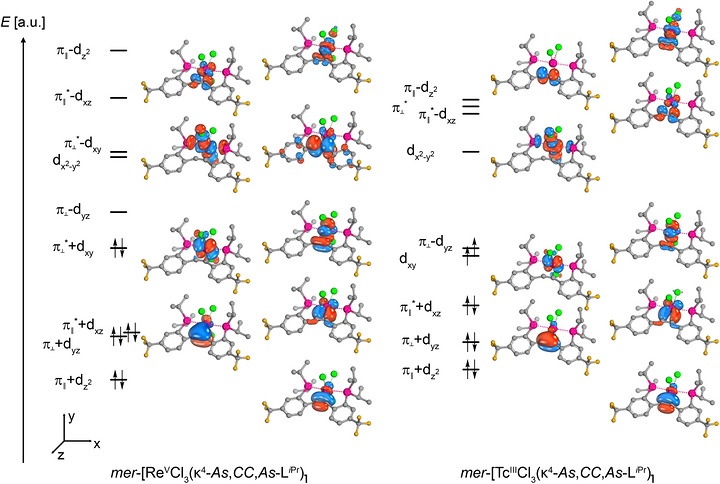
CAS(8,9) orbital interactions in *mer*‐[Re^V^Cl_3_(κ^4^‐*As*,*CC*,*As*‐L*^i^* ^Pr^)] (left) and *mer*‐[Tc^III^Cl_3_(κ^4^‐*As*,*CC*,*As*‐L*^i^* ^Pr^)] (right). The CAS is constructed from the alkyne π orbitals (π_∥_, π_⊥_, π_∥_*, π_⊥_*) and the five metal d orbitals respectively. Further ligand interactions were omitted.

To better understand the d orbital splitting in both Tc and Re compounds, the geometry around the rhenium ion in the complex was assessed using the SHAPE algorithm (see Table S9) [94, 95, 96, 97]. In both cases an octahedral geometry with a centroid(C≡C)+2As+3Cl donor set was a closer fit than all other potential hexacoordinate geometries with a shape measure of 0.65 (including idealized heptacoordinate geometries with a 2C+2As+3Cl donor set and a minimum shape measure of 4.95 for a pentagonal bipyramidal geometry). The formal d‐orbital splitting of Re derived from an octahedral ligand field is, thus, qualitatively understood when thinking about the considerable bond length differences between the co‐ligand donor atoms with the d_z_^2^ orbital oriented in the M···centroid(C≡C) axis. As such, the lowest lying orbital of nearly pure d character should be oriented in direction of the As–M–As axis—lying in the *xy* plane. Natural bonding orbital (NBO) analyses using NBO6.0 confirm this interpretation and only a single Re‐centered d‐orbital, d_xy_, can be localized for the singlet structure of *mer*‐[Re^V^Cl_3_(κ^4^‐*As*,*CC*,*As*‐L*^i^* ^Pr^)], while one doubly filled and two singly filled orbitals with mainly metal‐centered d orbital character can be identified for *mer*‐[Tc^III^Cl_3_(κ^4^‐*As*,*CC*,*As*‐L*^i^* ^Pr^)]. Complete active space self‐consistent field (CAS‐SCF) multireference calculations are consistent with the energetic predictions of the DFT calculations providing the lowest energy for a singlet d^2^ electron configuration for Re and a triplet ls‐d^4^ for Tc (details given in S4.1). The orbitals involved in the alkyne···M interaction according to the underlying d‐orbital energy splitting can be evaluated from such calculations as depicted in Figure 5.

The suspected formal oxidation states of the central metal ions and the *As*, *CC*, *As* ligands were finally confirmed by localized orbital bonding analysis (LOBA) [98]. Although the assignment of oxidation states especially by theoretical methods is strictly formal and can be ambiguous [99, 100], the exhaustive body of evidence in the present case suggests that *mer*‐[Re^V^Cl_3_(κ^4^‐*As*,*CC*,*As*‐L*^i^* ^Pr^)] is indeed best described as a formal Re^V^ species with a dianionic 4e^−^ alkyne donor, while a neutral pincer, 2e^−^ donor ligand and Tc(III) are formulated for *mer*‐[Tc^III^Cl_3_(κ^4^‐*As*,*CC*,*As*‐L*^i^* ^Pr^)]. The formation of a metallacyclopropene resonance structure through backdonation of the metals d electrons into alkyne π* orbitals and contraction of the M─C bonds contributes significantly to the stabilization of low valent transition metal alkyne complexes. Therefore, the lack of this resonance structure can explain the difficulties that are frequently encountered during the attempted syntheses of low valent technetium alkyne complexes. Even with the tethered alkyne ligands of the present communication, reactions with common starting materials containing the metal in even lower oxidation states, that is, those with the *fac*‐{Tc(CO)_3_}^+^, {Tc^I^(NO)}^2+^ or {Tc^II^(NO)}^3+^ cores, but also reactions with a slightly varied *tert*‐butyl substituted ligand led to undesired side‐reactions in our hands and no technetium‐containing products were identified (for details see S2.9, S5.8).

### 2.2 Technetium(V) Alkyne Complexes

**SCHEME 2:**
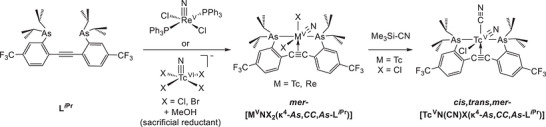
Reaction of L*^i^* ^Pr^ with rhenium and technetium nitrido starting materials.

Thus, complexes with technetium in a higher formal oxidation state that would favor the formation of classic 2e^−^ alkyne complexes became naturally interesting and related reactions are compiled in Scheme 2.

**FIGURE 6:**
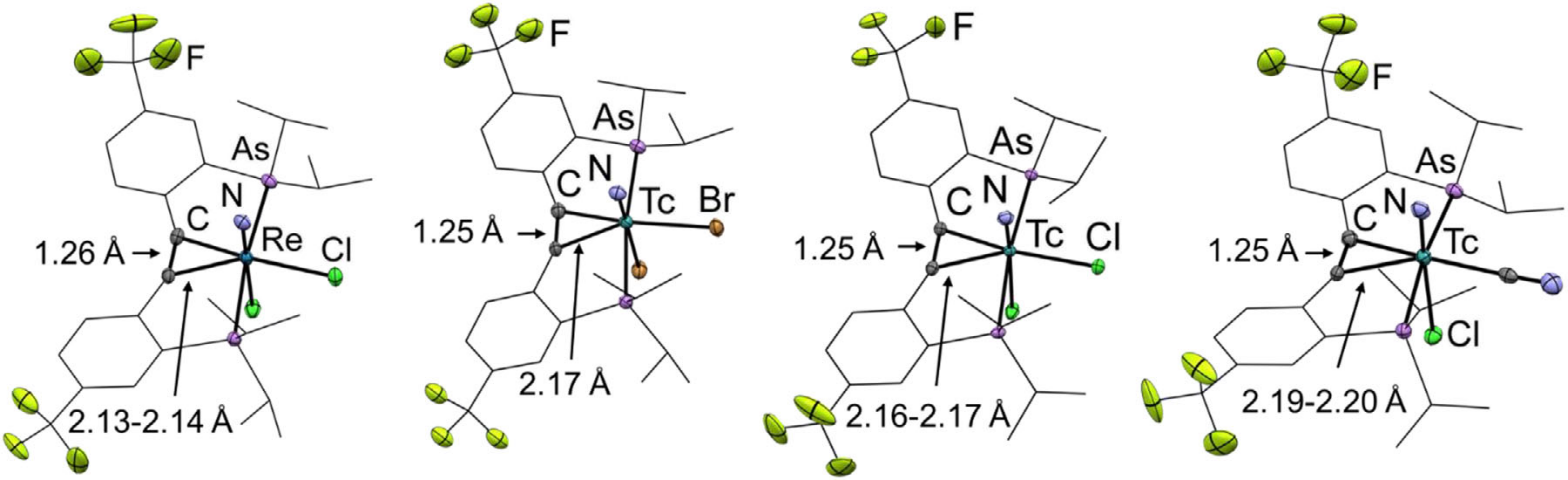
Molecular structures of the alkyne complexes *mer*‐[M^V^NCl_2_(κ^4^‐*As*,*CC*,*As*‐L*^i^* ^Pr^)] (M = Tc, Re), *mer*‐[Tc^V^NBr_2_(κ^4^‐*As*,*CC*,*As*‐L*^i^* ^Pr^)] and *cis,trans*,*mer*‐[Tc^V^N(CN)Cl(κ^4^‐*As*,*CC*,*As*‐L*^i^* ^Pr^)]. Ellipsoids are shown at 50% probability. Hydrogen atoms, disorder of the freely rotating CF_3_ groups and further labels are omitted for clarity. Skeletal carbon atoms are shown as wireframe for clarity. The bond parameters are compared in Table 2.

A remarkable difference between rhenium and technetium was observed during the attempted synthesis of nitrido metal(V) complexes. While the nearly insoluble rhenium(V) complex *trans*‐[Re^V^NCl_2_(PPh_3_)_2_] readily exchanges the two triphenylphosphine ligands for the pincer ligand forming *mer*‐[Re^V^NCl_2_(κ^4^‐*As*,*CC*,*As*‐L*^i^* ^Pr^)] in good yields after some days, the technetium analog *trans*‐[Tc^V^NCl_2_(PPh_3_)_2_] proved practically inert. Instead and as an established work‐around for the inertness of *trans*‐[Tc^V^NCl_2_(PPh_3_)_2_] in technetium chemistry, a reaction with [Tc^VI^NX_4_]^−^ (X = Cl, Br) using methanol as a sacrificial reductant readily led to the formation of *mer*‐[Tc^V^NX_2_(κ^4^‐*As*,*CC*,*As*‐L*^i^* ^Pr^)] in excellent yields. The ^13^C NMR resonances of the alkyne carbon atoms in *mer*‐[Tc^V^NX_2_(κ^4^‐*As*,*CC*,*As*‐L*^i^* ^Pr^)] are observed around 110 ppm, while those of *mer*‐[Re^V^NCl_2_(κ^4^‐*As*,*CC*,*As*‐L*^i^* ^Pr^)] are found around 120 ppm, both of which are consistent with a formulation as side‐on bonded alkyne complexes acting as classic 2e^−^ donor ligands when comparing it to the ^13^C NMR resonance observed for the same atoms in the free ligand L*^i^* ^Pr^ (95 ppm). DFT calculations on the B3LYP‐D3/def2‐TZVP level confirm the assignment as classic 2e^−^ donor alkyne units based on topological descriptors. The ^1^H NMR spectra of the nitrido complexes show a characteristic set of four resonances reflecting each potential orientation of the methyl groups in the *iso*‐propyl substituents of the complexes (*syn*/*anti* to M≡N, *syn*/*anti* to the aromatic ring) as well as two resonances for the tertiary C─H protons (*syn*/*anti* to M≡N) verifying the expectedly lower molecular symmetry compared to the trichloro complexes described above. The oxidation states of the products are readily verified by their characteristic ν(Tc≡N) around 1040 cm^−1^, which is in the common range for Tc(V) nitrido complexes and somewhat red‐shifted compared to the analogous ν(Re≡N) 1054 cm^−1^. No appreciable decomposition was observed even when heating for hours at 100°C in air. The reactivity and derivatization potential of the nitrido complexes as platforms for systematic studies on the technetium–alkyne bonds or for potential labeling with biologically relevant molecules by ligand exchange procedures was probed by reactions using silver or silyl‐based halide scavengers and bidentate ligands like bipyridine or *N*,*N*‐dialkylbenzoylthiourea derivatives. The alkyne complexes generally appear to be inert toward bidentate ligands and the starting material was recovered unchanged even after prolonged heating in boiling toluene. The more aggressive derivatization agent Me_3_Si─CN cleanly reacted with *mer*‐[Tc^V^NCl_2_(κ^4^‐*As*,*CC*,*As*‐L*^i^* ^Pr^)] forming a single, well‐defined product in near quantitative isolated yield: *cis,trans*,*mer*‐[Tc^V^N(CN)Cl(κ^4^‐*As*,*CC*,*As*‐L*^i^* ^Pr^)], while preliminary reactions with other silyl reagents were so far less successful (i.e., no reaction between *mer*‐[Tc^V^NBr_2_(κ^4^‐*As*,*CC*,*As*‐L*^i^* ^Pr^)] and Me_3_Si─N_3_ and decomposition during the crystallization of the initial product formed from the reaction between *mer*‐[Tc^V^NCl_2_(κ ^4^‐*As*,*CC*,*As*‐L*^i^* ^Pr^)] and Me_3_Si‐NCS; for further details see: S2.7, S5.10). The product is readily identified as a mono cyanido complex by the presence of a single C≡N stretching frequency at 2139 cm^‒1^ in the infrared spectrum, while the stereochemical *trans*‐arrangement of the cyanido ligand to the alkyne can be inferred from the blue‐shift of ν(RC≡CR) to 1956 cm ^‒1^ whereas ν(Tc≡N) remains essentially unchanged at 1038 cm^‒1^ compared to the starting material. Even prolonged heating using an excess of Me_3_Si─CN did not lead to a second ligand exchange reaction or decomposition. X‐ray quality crystals of the product were obtained directly from the reaction mixture confirming the spectroscopic assignment as a mono cyanido complex. The other technetium nitrido complexes also crystallized from the reaction mixture as large, colorless blocks that were suitable for x‐ray diffraction (solid‐state structures shown in Figure 6, selected bonding parameters are compared in Table 2).

**TABLE 2:** Selected bond lengths and angles in the rhenium and technetium nitrido alkyne complexes.

Bond lengths [Å]	*mer*‐[Re^V^NCl_2_ (κ^4^‐*As*,*CC*,*As*‐L*^i^* ^Pr^)]	*mer*‐[Tc^V^NBr_2_ (κ^4^‐*As*,*CC*,*As*‐L*^i^* ^Pr^)]	*mer*‐[Tc^V^NCl_2_ (κ^4^‐*As*,*CC*,*As*‐L*^i^* ^Pr^)]	*cis,trans*,*mer*‐ [Tc^V^N(CN)Cl (κ^4^‐*As*,*CC*,*As*‐L*^i^* ^Pr^)]
C≡C	1.262(4)	1.250(6)	1.254(3)	1.248(6)
M‐C_C≡C_	2.129(3) 2.137(3)	2.165(4) 2.167(4)	2.161(2) 2.165(2)	2.193(4) 2.195(4)
M···centroid(C≡C)	2.038	2.074	2.070	2.103
M≡N	1.671(2)	1.634(3)	1.630(2)	1.680(1)
M‐X_trans‐C≡C_	2.4191(8)	2.5661(7)	2.4207(7)	2.151(4)
M‐X_trans‐M≡N_	2.6980(8)	2.9003(7)	2.7262(7)	2.680(1)
M‐As	2.5490(5) 2.5541(5)	2.5423(6) 2.5504(6)	2.5402(5) 2.5464(5)	2.5439(6) 2.5442(6)
C≡N	—	—	—	1.103(6)
Angles [°]				
N≡M···centroid(C≡C)	99.6	100.1	99.4	99.9
As‐M···centroid(C≡C)	92.5 93.1	92.3 92.8	92.2 92.9	92.4 92.6
X‐M···centroid(C≡C)	79.8 162.0	78.6 162.0	78.8 162.1	82.6 163.7
Ar‐C(≡CR)‐M	130.7(2) 131.1(2)	129.1(3) 129.6(3)	129.2(2) 129.7(2)	128.0(3) 128.1(3)
Ar‐C≡C	156.2(3) 156.4(3)	157.3(4) 157.6(4)	157.4(2) 157.4(2)	157.9(4) 158.4(4)
N≡M‐X	98.43(9) 176.08(9)	97.8(1) 177.5(1)	98.54(7) 175.45(7)	86.1(1) 177.4(1)
N≡M‐As	92.07(9) 92.22(9)	93.0(1) 93.2(2)	92.32(7) 92.48(7)	91.8(1) 92.7(1)
As‐M‐As	172.35(2)	171.19(2)	172.36(2)	172.62(2)
X‐M‐X	82.35(2)	83.53(2)	83.40(2)	82.9(1)

### 2.3 ^99m^Tc Alkyne Complex

**SCHEME 3:**
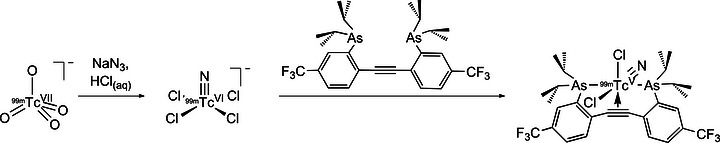
Preparation of *mer*‐[^99m^Tc^V^NCl_2_(κ^4^‐*As*,*CC*,*As*‐L*^i^* ^Pr^)].

The presented technetium alkyne complexes can be isolated under ambient conditions and they are relatively inert toward competing ligands. They are therefore sufficiently robust under conditions that are feasible for potential radiopharmaceutical applications and the synthesis of ^99m^Tc complexes became interesting. While the arsenic‐based complexes of the present study are obviously not directly suitable for such applications due to the toxicity and high lipophilicity of organoarsenic compounds, the translation to ^99m^Tc proves the viability of the initial hypothesis that the integration of alkyne donors into chelator frameworks can result in exceedingly stable donor sets for the tethering biomolecules to the radiometal. Metal‐tethering of small peptides by pendant alkyne donors has been achieved, e.g., for tungsten before. [101] In a proof of concept, a synthetic protocol akin to the procedure for the ^99^Tc compound described above was derived *via* [^99m^Tc^VI^NCl_4_]^−^ to provide *mer*‐[^99m^Tc^V^NCl_2_(κ^4^‐*As*,*CC*,*As*‐L*^i^* ^Pr^)] (Scheme 3).

It is well‐known that protocols for the preparation of the ^99m^Tc nitrido precursor frequently lead to complex mixtures of variable composition [102]. This is not a serious issue as the final products can commonly be purified easily from traces of the inadvertently formed left‐over side‐products by standard solid state extraction following immobilization on a reverse phase chromatographic column after chelation [102]. This holds true in the present reaction, where inadvertent side‐products from the starting material synthesis were readily removed by chromatographic purification. Once formed, the chelate complex remained stable as verified by repeated, decay‐corrected HPLC analysis of the purified complex *mer*‐[^99m^Tc^V^NCl_2_(κ^4^‐*As*,*CC*,*As*‐L*^i^* ^Pr^)]. The HPLC trace did not show significant decomposition during the decay time of ^99m^Tc suggesting alkyne‐units as suitable donor building blocks for the generation of stable technetium chelate complexes for medicinal applications.

## 3 Conclusion

Alkyne complexes of technetium — a compound class that had eluded isolation attempts for decades with technetium being the last d‐block element in periods 4, 5, and 6 without known side‐on alkyne ligands — have finally been isolated by forcing the side‐on coordination of an internal C≡C triple bond using an *As*,*CC*,*As* tolane‐based pincer ligand. Technetium alkyne complexes have a lower tendency to form 4e^−^ metallacyclopropene resonance structures in comparison to their isostructural heavier homolog rhenium. The electronic differences have consequences for the reactivity of the technetium compounds, especially when compared with their rhenium congeners and are at least in part responsible for the difficulties encountered in previous preparation attempts. The alkyne motif was proven to coordinate the medicinally relevant nuclear isomer ^99m^Tc at the tracer level under formation of stable chelates that structurally resemble their ^99^Tc and Re analogs. Thus, C≡C triple bonds are suggested as valuable design motifs for future radiopharmaceutical chelators providing excellent stability for tethering of technetium to biologically active molecules.

## Author Contributions

**Maximilian Roca Jungfer**: conceptualization, methodology, investigation, validation, funding acquisition, visualization, writing – original draft, writing – review and editing, project administration, resources, supervision, data curation, formal analysis, software. **Moritz Johannes Ernst**: formal analysis, investigation, methodology, writing ‐ review & editing, translation. **Lukas Eberle**: formal analysis, investigation, methodology, writing ‐ review & editing. **Marius Kesselring**: investigation. **Guilhem Claude**: formal analysis, investigation, resources, writing ‐ review & editing. **Joachim Ballmann**: funding acquisition, investigation, resources.

## Funding

Fonds der Chemischen Industrie (FCI), Liebig fellowship to MRJ. The state of Baden‐Württemberg through bwHPC (JUSTUS cluster). German Research Foundation grant INST 40/467‐1 FUGG (JUSTUS cluster) and BA 4859/3‐2 (LE, JB). Open Access enabled by Karlsruhe Institute of Technology KIT Publication Fund (Project DEAL).

## Conflicts of Interest

The authors declare no conflicts of interest.

## Supporting information




**Supporting File 1**: Experimental details (S1), Crystallographic data (S2), Spectral data (S3), Computational data (S4), Additional figures and explanatory text (S5), an excel sheet containing tabular information about the literature surveys and compact calculation information and crystallographic information files (CIF/CheckCIF) are provided as Supporting Information. All data are available in the main text or the supplementary materials. CCDC 2521631 to CCDC 2521634 and CCDC 2521636 to CCDC 2521640 contain the supplementary crystallographic data for this paper. The data can be obtained free of charge from The Cambridge Crystallographic Data Centre via www.ccdc.cam.ac.uk/structures. Original data (e.g., spectra files) are available through reasonable requests by contacting the corresponding author. The procedures for the preparation of all materials are contained in the Supporting Information.
The authors have cited additional references within the Supporting Information [103, 104, 105, 106, 107, 108, 109, 110, 111, 112, 113, 114, 115, 116, 117, 118, 119, 120, 121, 122, 123, 124, 125, 126, 127, 128, 129, 130, 131, 132, 133, 134, 135, 136, 137, 138, 139, 140, 141, 142, 143, 144, 145, 146, 147, 148, 149, 150].

**Supporting File 2**: anie72357‐sup‐0002‐Data.zip.

## Data Availability

The data that supports the findings of this study are available in the supplementary material of this article. Original data (e.g., spectra files) are available from the corresponding author upon reasonable request.
