# Supplementary material for: Forcing the Side‐on π‐Coordination of a C≡C Triple Bond to Technetium Using the As,CC,As Alkyne Pincer Ligand 1,2‐Bis(2‐(diisopropylarsaneyl)‐4‐(trifluoromethyl)phenyl)Ethyne
Source: Angew Chem Int Ed Engl. 2026 May 2;65(25):e6878935. doi: 10.1002/anie.6878935 (PMC13266937; doi:10.1002/anie.6878935)
Supplement: Supplementary file 1 — Supporting File 1: Experimental details (S1), Crystallographic data (S2), Spectral data (S3), Computational data (S4), Additional figures and explanatory text (S5), an excel sheet containing tabular information about the literature surveys and compact calculation information and crystallographic information files (CIF/CheckCIF) are provided as Supporting Information. All data are available in the main text or the supplementary materials. CCDC 2521631 to CCDC 2521634 and CCDC 2521636 to CCDC 2521640 contain the supplementary crystallographic data for this paper. The data can be obtained free of charge from The Cambridge Crystallographic Data Centre via www.ccdc.cam.ac.uk/structures. Original data (e.g., spectra files) are available through reasonable requests by contacting the corresponding author. The procedures for the preparation of all materials are contained in the Supporting Information. The authors have cited additional references within the Supporting Information [103, 104, 105, 106, 107, 108, 109, 110, 111, 112, 113, 114, 115, 116, 117, 118, 119, 120, 121, 122, 123, 124, 125, 126, 127, 128, 129, 130, 131, 132, 133, 134, 135, 136, 137, 138, 139, 140, 141, 142, 143, 144, 145, 146, 147, 148, 149, 150]. [file ANIE-65-e6878935-s002.pdf]

Supporting information for the paper entitled:

Forcing the Side-on π-Coordination of a C≡C Triple Bond to Technetium Using the *As,CC,As* Alkyne Pincer Ligand 1,2-Bis(2-(diisopropylarsaneyl)-4-(trifluoromethyl)phenyl)ethyne

M. Roca Jungfer,[a],*, M. J. Ernst,[b] L. Eberle,[c] M. Kesselring,[b],[d] G. Claude,[d] J. Ballmann[c]

[1]Karlsruher Institut für Technologie, Institut für Nukleare Entsorgung; Eggenstein-Leopoldshafen, 76344, Germany.

[2]Freie Universität Berlin, Berlin, 14195, Germany.

[3]Ruprecht-Karls-Universität Heidelberg; Heidelberg, 69120, Germany.

[4]Charité – Universitätsmedizin Berlin, corporate member of Freie Universität Berlin and Humboldt Universität zu Berlin, Klinik für Nuklearmedizin/Radiopharmazie, Forum 4, 13353 Berlin, Germany.

*Corresponding author. Email: m.roca.jungfer@gmail.com; maximilian.roca-jungfer@partner.kit.edu.

Table of contents

Part S1: Experimental details 4

S1.1 Safety preambel 4

S1.2 General information 4

S1.3 *mer*-[$Tc^{III}Cl_3$(κ^4-*As*,*CC*,*As*-L^{iPr})] 5

S1.4 *mer*-[$Tc^{V}NBr_2$(κ^4-*As*,*CC*,*As*-L^{iPr})] 5

S1.5 *mer*-[$Tc^{V}NCl_2$(κ^4-*As*,*CC*,*As*-L^{iPr})] 6

S1.6 *cis,trans,mer*-[Tc^{V}N(CN)Cl(κ^4-*As*,*CC*,*As*-L^{iPr})] 7

S1.7 *mer*-[$Re^{III}Cl_3$(κ^4-*As*,*CC*,*As*-L^{iPr})] 8

S1.8 *mer*-[$Re^{V}NCl_2$(κ^4-*As*,*CC*,*As*-L^{iPr})] 8

Part S2: Crystallographic data 10

S2.1 Crystal data and structure determination parameters. 10

S2.1 *mer*-[$Tc^{V}NBr_2$(κ^4-*As*,*CC*,*As*-L^{iPr})]. 14

S2.2 *mer*-[$Tc^{V}NCl_2$(κ^4-*As*,*CC*,*As*-L^{iPr})]. 15

S2.3 *cis,trans,mer*-[Tc^{V}N(CN)Cl(κ^4-*As*,*CC*,*As*-L^{iPr})]. 16

S2.4 *mer*-[$Tc^{III}Cl_3$(κ^4-*As*,*CC*,*As*-L^{iPr})]. 17

S2.5 *mer*-[$Re^{V}NCl_2$(κ^4-*As*,*CC*,*As*-L^{iPr})]. 19

S2.6 *mer*-[$Re^{V}Cl_3$(κ^4-*As*,*CC*,*As*-L^{iPr})]. 20

S2.7 ($L^{iPr}(OH)_2)_2[Tc^{V}N(NCS)_4$(THF)]$Cl_2$·3THF. 21

S2.8 Cyclization product of L^{tBu}. 25

S2.9 [Ag^{I}(κ^2-*As*,*CC*,*As*-L^{iPr})](BF_4). 26

Part 3: Spectral data 27

S3.1 ^{1}H & ^{13}C NMR assignment overview 27

S3.2 *mer*-[$Tc^{V}NBr_2$(κ^4-*As*,*CC*,*As*-L^{iPr})]. 28

S3.3 *mer*-[$Tc^{V}NCl_2$(κ^4-*As*,*CC*,*As*-L^{iPr})]. 31

S3.4 *cis,trans,mer*-[Tc^{V}N(CN)Cl(κ^4-*As*,*CC*,*As*-L^{iPr})]. 38

S3.5 *mer*-[$Tc^{III}Cl_3$(κ^4-*As*,*CC*,*As*-L^{iPr})]. 40

S3.6 *mer*-[$Re^{V}NCl_2$(κ^4-*As*,*CC*,*As*-L^{iPr})]. 42

S3.7 *mer*-[$Re^{V}Cl_3$(κ^4-*As*,*CC*,*As*-L^{iPr})]. 50

S3.8 *mer*-[$^{99m}Tc^{V}NCl_2$(κ^4-*As*,*CC*,*As*-L^{iPr})]. 57

Part S4: Computational data 59

S4.1 Computational details: L^{iPr} complexes 59

S4.2 *mer*-[$Tc^{V}NBr_2$(κ^4-*As*,*CC*,*As*-L^{iPr})]. 63

S4.3 *mer*-[$Tc^{V}NCl_2$(κ^4-*As*,*CC*,*As*-L^{iPr})]. 65

S4.4 *cis,trans,mer*-[Tc^{V}N(CN)Cl(κ^4-*As*,*CC*,*As*-L^{iPr})]. 67

S4.5 *mer*-[$Tc^{III}Cl_3$(κ^4-*As*,*CC*,*As*-L^{iPr})]: singlet/d^2 69

S4.6 *mer*-[$Tc^{III}Cl_3$(κ^4-*As*,*CC*,*As*-L^{iPr})]: triplet/d^4-ls; energetically preferred ... 71

S4.7 *mer*-[$Tc^{III}Cl_3$(κ^4-*As*,*CC*,*As*-L^{iPr})]: quintet/d^4-hs ... 74

S4.8 *mer*-[$Re^{V}NCl_2$(κ^4-*As*,*CC*,*As*-L^{iPr})]. ... 77

S4.9 *mer*-[$Re^{V}Cl_3$(κ^4-*As*,*CC*,*As*-L^{iPr})]. Singlet/d^2; energetically preferred ... 79

S4.10 *mer*-[$Re^{V}Cl_3$(κ^4-*As*,*CC*,*As*-L^{iPr})]. Triplet/d^4-ls ... 81

S4.11 *mer*-[$Re^{V}Cl_3$(κ^4-*As*,*CC*,*As*-L^{iPr})]. Triplet/d^4-ls ... 84

S4.12 *mer*-[$Mn^{III}Cl_3$(κ^4-*As*,*CC*,*As*-L^{iPr})]: singlet/d^2 ... 87

S4.13 *mer*-[$Mn^{III}Cl_3$(κ^4-*As*,*CC*,*As*-L^{iPr})]: triplet/d^4-ls ... 89

S4.14 *mer*-[$Mn^{III}Cl_3$(κ^2-*As*,*CC*,*As*-L^{iPr})]: quintet/d^4-hs; energetically preferred ... 92

S4.15 Computational details: alkyne vs. vinylidene carbene coordination for zero valent d-block elements ... 95

S4.16 Computational details: alkyne vs. vinylidene carbene coordination for isostructural [M···(HC≡CH)N$(Cl)_2(AsH_3)_2$]$^{+,0,-}$ and [M=(C=CH_2)N$(Cl)_2(AsH_3)_2$]$^{+,0,-}$ model complexes of groups 6, 7 and 8 ... 103

Part S5: Additional figures and explanatory text ... 106

S5.1 Literature survey data on alkyne complexes of the d-block elements ... 106

S5.2 C≡C bond lengths in structurally characterized *E*,(*CC*),*E* (E = P, As) complexes.[72-85] ... 107

S5.3 M-$C_{C≡C}$ bond lengths in structurally characterized *E*,(*CC*),*E* (E = P, As) complexes.[72-85] ... 111

S5.4 ^{13}C NMR chemical shifts in *E*,*CC*,*E* (E = P, As) complexes.[72-85] ... 115

S5.5 $OPPh_3$ during reactions of [$Tc^{III}Cl_3(PPh_3)_2(NCCH_3)$] ... 118

S5.6 Reactivity differences between *mer*-[MCl_3(κ^4-*As*,*CC*,*As*-L^{iPr})] (M = Tc, Re). ... 118

S5.7 Attempted preparation of [$MnCl_3$(κ^4-*As*,*CC*,*As*-L^{iPr})]. ... 118

S5.8 Attempted preparation of [$Tc(CO)_3$(κ^4-*As*,*CC*,*As*-L^{iPr})]$^+$. ... 118

S5.9 Attempted preparation of [$TcCl_3$(κ^4-*As*,*CC*,*As*-L^{tBu})] ... 118

S5.10 Attempted preparation of [TcN(NCS)Cl(κ^4-*As*,*CC*,*As*-L^{iPr})] & [TcN(N_3)Br(κ^4-*As*,*CC*,*As*-L^{iPr})]. ... 119

S5.11 Overview over attempted reactions of alkynes with technetium starting materials. 120

Part S6: References ... 123

Part S1: Experimental details

S1.1 Safety preambel

Radiatiation precaution: All synthetic work with the long-lived isotope ^{99}Tc was performed in a laboratory approved for the handling of radioactive material. Glass walls of the flasks provide appropriate protection of the primary beta emission of ^{99}Tc. Secondary X-rays (bremsstrahlung) become important only when larger amounts of the compounds are handled as solids. All personnel working in this project was permanently monitored for potential contaminations.

Toxicity precaution: Arsenic compounds, in particular liquid organoarsenic derivatives (e.g. $^{i}Pr_2AsCl$) are highly toxic. All operations with these compounds were carried out in a well-ventilated fume hood using appropriate personal safety equipment. The use of thick-walled rubber gloves and safety shields (e.g. during distillation) is highly recommended. Arsenic-containing waste was oxidatively hydrolyzed (H_2O_2) to As_2O_3 and stored separately from other waste chemicals. Note that As_2O_3 is toxic as well and requires separate disposal. Due to legally binding restrictions, elemental analysis of arsenic compounds is prohibited as toxic As_2O_3 vapors are produced during combustion. Hence, we were not allowed to perform elemental analysis on all As-containing compounds reported herein.

S1.2 General information

Air- and moisture-sensitive reactions were carried out under Argon using standard Schlenk techniques. L^{iPr},[84] L^{tBu},[85] $[Tc^{III}Cl_3(PPh_3)_2(NCCH_3)]$,[103] and $NBu_4[Tc^{VI}NX_4]$,[102] were prepared by published protocols. $Na^{99m}Tc^{VII}O_4$ (50 µL, 671 MBq) was obtained from a commercial ^{99}Mo/^{99m}Tc generator system (Monrol®). The precursor $NBu_4[^{99m}Tc^{VI}NCl_4]$ was prepared by modifying the reported procedure for $PPh_4[^{99m}Tc^{VI}NCl_4]$,[102] followed by the provided ligand exchange protocol. Activity counting was determined using a borehole counter (Isomed, Nuvia, Germany). HPLC was performed using the HPLC system Knauer Azura (P6.1L) coupled with UV (254 nm) and radiometric (NaI, Gabi Star, Elysia Raytest, Germany) detectors. The radiochemical yield of $[^{99m}Tc^{VI}NCl_4]^-$ was determined for an aliquot (300 µL) of the used $[^{99m}Tc^{VI}NCl_4]^-$ extract (2.1 mL; methyl ethyl ketone). HPLC method for all analyses: Mobile phase A (100% $NCCH_3$ + 0.1% CF_3COOH); mobile phase B (deionized H_2O + 0.1% CF_3COOH); elution gradient: 10% A/90% B over 30 min to 90% A/10% B; flow rate: 0.8 mL/min. All other reagents and solvents were purchased from commercial suppliers. All chemicals were used as received. Absorption correction was performed using SADABS,[104] structures were solved with SHELXT-2018/2 (Sheldrick 2015),[105] and refined using the SHELXL-2018/3 (Sheldrick, 2018).[106] The Bruker programs APEX, APEX2, SMART, SAINT, SAINT-Plus were used for data processing.[107] Visualization was done using Mercury.[108] Structure solution, refinement and finalization was done using OLEX2.[109] CCDC 2521631 to CCDC 2521634 and CCDC 2521636 to CCDC 2521640 contain the supplementary crystallographic data for this paper. The data can be obtained free of charge from The Cambridge Crystallographic Data Centre via www.ccdc.cam.ac.uk/structures. A survey of available solid state structures was collected using Conquest to search through the CSD database (version of record Oct. 2025), which were prepared for analysis in Mercury.[33, 34] ^{1}H, ^{13}C{^{1}H}, ^{19}F and 2D NMR spectra of technetium compounds were recorded at 20°C on ^{1}H, ^{13}C{^{1}H}, ^{19}F and 2D NMR spectra of rhenium compounds were recorded at 20°C on a Bruker Avance II 400 or on a Bruker Avance II 600 spectrometer. Chemical shifts (δ) are indicated in parts per million (ppm) and are referenced to the residual signal of the deuterated solvent relative to the external calibration standard trimethylsilane: CD_2Cl_2

or $CDCl_3$ (5.32 ppm or 7.26 ppm for ^{1}H NMR). ^{19}F NMR chemical shifts are referenced to the external calibration standard $CFCl_3$ in CH_2Cl_2. Coupling constants (*J*) are indicated in hertz (Hz). Multiplicities are abbreviated as follows: s – singlet, d – doublet, t – triplet, hept – septet, m – multiplet, br – broad signal. Spectral data are provided as Supporting Information (Section S2). Infrared spectra of technetium compounds were measured as KBr pellets on a FT-IR spectrometer (Shimadzu IR Affinity-1), while IR spectra of rhenium compounds were collected from neat powder on a FT-IR spectrometer (Bruker LUMOS) with a Germanium ATR crystal. High resolution mass spectra were recorded on a Bruker ApexQe FT-ICR spectrometer (electrospray ionization, ESI) in positive ion mode or on a JEOL AccuTOF GCx orthogonal-acceleration time-of-flight (oaTOF) mass spectrometer (liquid injection field desorption ionization, LIFDI).

S1.3 *mer*-[$Tc^{III}Cl_3$(κ^4-*As*,*CC*,*As*-L^{iPr})]

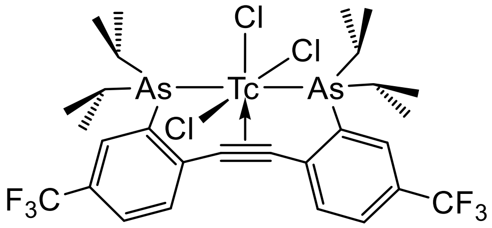

A mixture of L^{iPr} (255 mg, 0.40 mmol) and [$Tc^{III}Cl_3(PPh_3)_2(NCCH_3)$] (308 mg, 0.40 mmol) in CH_2Cl_2 (3 mL) was stirred under Ar for 6 h at RT. The solvent was evaporated in vacuum and the red, powdered residue was washed with pentane to remove the released PPh_3. Red powder. Yield: 335 mg (0.40 mmol, quantitative). Single crystals suitable for X-ray diffraction of [$TcCl_3$(κ^4-*As*,*CC*,*As*-L^{iPr})] were either grown from the isolated powder as CH_2Cl_2 solvate or with co-crystallized $OPPh_3$ when directly evaporating the reaction mixture in air. **FT-IR (KBr; transmission):** $\tilde{\nu}$ [cm^{-1}] = 3057 (w), 2960 (m), 2924 (m), 2888 (m), 1655 (w), 1597 (m), 1462 (m), 1438 (m), 1393 (m), 1321 (vs), 1255 (m), 1230 (m), 1174 (s), 1124 (vs), 1073 (s), 1039 (m), 998 (w), 930 (w), 905 (w), 878 (w), 843 (m), 750 (m), 722 (s), 695 (m), 541 (vs), 463 (w), 430 (w). **^{1}H NMR (399.4 MHz, CD_2Cl_2, 20°C; paramagnetic):** δ (ppm) 18.1 (s), 12.4 (s), 9.1 (s), 8.8 (s), 7.7 (s), 7.6 (s), 7.5 (s), 5.4 (s), 2.9 (s), 2.2 (s), 1.-1.3 (m, broad), 0.9 (s), -7.7 (s). **^{19}F{^{1}H} NMR (375.8 MHz, CD_2Cl_2, 20°C)**: δ (ppm) -19.9 (s, C*F*$_3$). **μ_{eff}(CD_2Cl_2; two independent measurements):** 2.2; 2.3.

S1.4 *mer*-[$Tc^{V}NBr_2$(κ^4-*As*,*CC*,*As*-L^{iPr})]

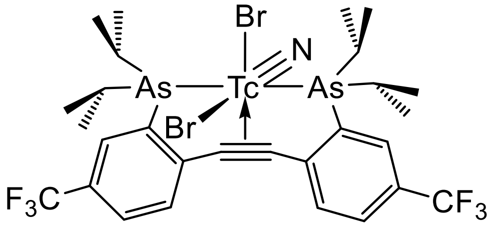

A suspension of L^{iPr} (32 mg, 50 µmol) in CH_3CN (0.5 mL) was added to a brilliant blue solution of $NBu_4[Tc^{VI}NBr_4]$ (34 mg, 50 µmol) in CH_3CN (0.5 mL). After addition of MeOH (2 drops) as a sacrificial reductant, the mixture slowly discolored, while heated at reflux for 2 h. Large colorless blocks of the product had formed and the mixture was cooled. It was stored in a refrigerator overnight to complete crystallization, filtered and the crystals were washed with a few drops of thawing CH_3CN to remove traces of NBu_4^+ salts. After drying, the colorless product was washed with pentane (0.5 mL) to remove traces of unreacted ligand. Colorless powder or yellowish blocks. Yield: 18 mg

(20 µmol, 40%). The crystals were suitable for X-ray diffraction. **FT-IR (KBr; transmission):** $\tilde{\nu}$ [cm^{-1}] = 1937 (w, v, RC≡CR), 1042 (m, v, Tc≡N). **^{1}H NMR (399.4 MHz, CD_2Cl_2, 20°C):** δ (ppm) 8.39 (d, $^{3}J_{H\text{-}H}$ = 8.1 Hz, 2H, ^{Ar}H, *m*-As, *m*-CF_3, *o*-C≡C), 8.05 (s, 2H, ^{Ar}H, *o*-As, *o*-CF_3, *m*-C≡C), 7.99 (d, $^{3}J_{H\text{-}H}$ = 8.1 Hz, 2H, ^{Ar}H, *p*-As, *o*-CF_3, *m*-C≡C), 3.92 (hept, $^{3}J_{H\text{-}H}$ = 7.3 Hz, 2H, C*H*(CH_3)$_2$, underneath equatorial plane; *anti* to Tc≡N), 3.34 (hept, $^{3}J_{H\text{-}H}$ = 6.9 Hz, 2H, C*H*(CH_3)$_2$, above equatorial plane; *syn* to Tc≡N), 1.79 (dd, $^{3}J_{H\text{-}H}$ = 9.5 Hz, , $^{3}J_{H\text{-}H}$ = 7.1 Hz, 12H, CH(C*H*$_3$)$_2$, above equatorial plane; *syn* to Tc≡N, pointed away from aromatic ring & below equatorial plane; *anti* to Tc≡N, pointed away from aromatic ring), 1.64 (d, $^{3}J_{H\text{-}H}$ = 7.2 Hz, 6H, CH(C*H*$_3$)$_2$, below equatorial plane; *anti* to Tc≡N, pointed towards aromatic ring), 1.15 (d, $^{3}J_{H\text{-}H}$ = 7.0 Hz, 6H, CH(C*H*$_3$)$_2$, above equatorial plane; *syn* to Tc≡N, pointed towards aromatic ring). .**^{13}C{^{1}H} NMR (100.4 MHz, CD_2Cl_2, 20°C):** δ (ppm) 148.4 (s, $^{q}C^{Ar}$-AsR_2), 139.0 (s, $^{q}C^{Ar}$-C≡C), 134.4 (s, C^{Ar}-H, *m*-As, *m*-CF_3, *o*-C≡C), 131.8 (q, $^{2}J_{C\text{-}F}$ = 33 Hz, $^{q}C^{Ar}$-CF_3), 130.7 (q, $^{3}J_{C\text{-}F}$ = 4 Hz, C^{Ar}-H, *o*-As, *o*-CF_3, *m*-C≡C), 128.9 (q, $^{3}J_{C\text{-}F}$ = 3 Hz, C^{Ar}-H, *p*-As, *o*-CF_3, *m*-C≡C), 125.5 (q, $^{1}J_{C\text{-}F}$ ≈ 250 Hz, *C*F$_3$; due to extremely low intensity and poor resolution of the ^{13}C{^{1}H} resonance the shift and coupling constant were estimated from the available data for the two central lines), 112.1 (s, C≡C), 34.6 (s, *C*H(CH_3)$_2$, underneath equatorial plane; *anti* to Tc≡N), 31.4 (s, *C*H(CH_3)$_2$, above equatorial plane; *syn* to Tc≡N), 22.4 (s, CH(*C*H$_3$)$_2$, below equatorial plane; *anti* to Tc≡N, pointed towards aromatic ring), 21.7 (s, CH(*C*H$_3$)$_2$, below equatorial plane; *anti* to Tc≡N, pointed away from aromatic ring), 21.2 (s, CH(*C*H$_3$)$_2$, above equatorial plane; *syn* to Tc≡N, pointed away from aromatic ring), 19.6 (s, CH(*C*H$_3$)$_2$, above equatorial plane; *syn* to Tc≡N, pointed towards aromatic ring). **^{19}F{^{1}H} NMR (375.8 MHz, CD_2Cl_2, 20°C)**: δ (ppm) -62.7 (s, C*F*$_3$).

S1.5 *mer*-[$Tc^{V}NCl_2$(κ^4-*As*,*CC*,*As*-L^{iPr})]

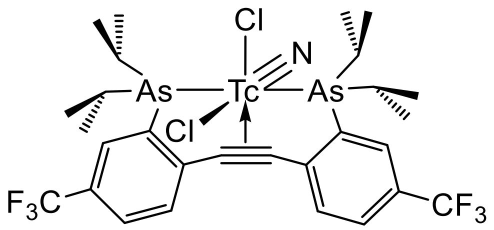

A) *mer*-[$^{99}Tc^{V}NCl_2$(κ^4-*As*,*CC*,*As*-L^{iPr})]. L^{iPr} (250 mg, 0.39 mmol) was added to a solution of NBu_4[$Tc^{VI}NCl_4$] (190 mg, 0.39 mmol) in CH_3CN (3 mL), followed by MeOH (0.5 mL) and CH_2Cl_2 (3 mL). The mixture was heated at reflux for 15 min. Crystals of the product formed shortly thereafter. The reaction was then opened to air and heated until the total volume was reduced to ca. 0.5 mL. It was stored in a refrigerator overnight to complete the crystallization of the product. The solid was filtered off and rapidly washed with a few drops of thawing CH_3CN to remove traces of NBu_4^+ salts. After drying, the colorless product was washed with pentane (1 mL) to remove traces of unreacted ligand. Additional fractions can be recovered from the filtrate and washing solutions as the product is partially soluble in acetonitrile. Colorless powder or pinkish blocks. Yield: 262 mg (0.32 mmol, 82%). The crystals were suitable for X-ray diffraction. **FT-IR (KBr; transmission):** $\tilde{\nu}$ [cm^{-1}] = 1938 (w, v, RC≡CR), 1040 (m, v, Tc≡N). **^{1}H NMR (399.4 MHz, CD_2Cl_2, 20°C):** δ (ppm) 8.37 (d, $^{3}J_{H\text{-}H}$ = 8.1 Hz, 2H, ^{Ar}H, *m*-As, *m*-CF_3, *o*-C≡C), 8.04 (s, 2H, ^{Ar}H, *o*-As, *o*-CF_3, *m*-C≡C), 7.99 (d, $^{3}J_{H\text{-}H}$ = 8.2 Hz, 2H, ^{Ar}H, *p*-As, *o*-CF_3, *m*-C≡C), 3.69 (pd, $^{3}J_{H\text{-}H}$ = 7.2 Hz, $^{3}J_{H\text{-}H}$ = 1.9 Hz, 2H, C*H*(CH_3)$_2$, underneath equatorial plane; *anti* to Tc≡N), 3.33 (pd, $^{3}J_{H\text{-}H}$ = 7.2 Hz, $^{3}J_{H\text{-}H}$ = 1.9 Hz, 2H, C*H*(CH_3)$_2$, above equatorial plane; *syn* to Tc≡N), 1.79 (dd, $^{3}J_{H\text{-}H}$ = 7.0 Hz, $^{3}J_{H\text{-}H}$ = 1.7 Hz, 6H, CH(C*H*$_3$)$_2$, above equatorial plane; *syn* to Tc≡N, pointed away from aromatic ring), 1.73 (dd, $^{3}J_{H\text{-}H}$ = 7.3 Hz, $^{3}J_{H\text{-}H}$ = 1.7 Hz, 6H, CH(C*H*$_3$)$_2$, below equatorial plane; *anti* to Tc≡N, pointed

away from aromatic ring), 1.62 (dd, $^3J_{H\text{-}H}$ = 7.2 Hz, $^3J_{H\text{-}H}$ = 1.8 Hz, 6H, CH(CH_3)$_2$, below equatorial plane; *anti* to Tc≡N, pointed towards aromatic ring), 1.20 (dd, $^3J_{H\text{-}H}$ = 7.0 Hz, , $^3J_{H\text{-}H}$ = 1.6 Hz, 6H, CH(CH_3)$_2$, above equatorial plane; *syn* to Tc≡N, pointed towards aromatic ring). .**^{13}C{^{1}H} NMR (100.4 MHz, CD_2Cl_2, 20°C):** δ (ppm) 148.0 (s, $^qC^{Ar}$-AsR$_2$), 139.5 (s, $^qC^{Ar}$-C≡C), 134.8 (s, C^{Ar}-H, *m*-As, *m*-CF$_3$, *o*-C≡C), 131.9 (q, $^2J_{C\text{-}F}$ = 33 Hz, $^qC^{Ar}$-CF$_3$), 130.6 (q, $^3J_{C\text{-}F}$ = 4 Hz, C^{Ar}-H, *o*-As, *o*-CF$_3$, *m*-C≡C), 129.0 (q, $^3J_{C\text{-}F}$ = 3 Hz, C^{Ar}-H, *p*-As, *o*-CF$_3$, *m*-C≡C), 124.0 (q, CF_3; due to extremely low intensity and poor resolution of the ^{13}C{^{1}H} resonance the reported shift is that of the singlet observed in broadband decoupled ^{13}C{^{19}F,^{1}H}), 111.9 (s, C≡C), 33.3 (s, CH(CH$_3$)$_2$, underneath equatorial plane; *anti* to Tc≡N), 31.1 (s, CH(CH$_3$)$_2$, above equatorial plane; *syn* to Tc≡N), 21.7 (s, CH(CH$_3$)$_2$, below equatorial plane; *anti* to Tc≡N, pointed towards aromatic ring), 21.4 (s, CH(CH$_3$)$_2$, below equatorial plane; *anti* to Tc≡N, pointed away from aromatic ring), 20.8 (s, CH(CH$_3$)$_2$, above equatorial plane; *syn* to Tc≡N, pointed away from aromatic ring), 19.7 (s, CH(CH$_3$)$_2$, above equatorial plane; *syn* to Tc≡N, pointed towards aromatic ring). **^{19}F{^{1}H} NMR (375.8 MHz, CD_2Cl_2, 20°C)**: δ (ppm) -62.7 (2 overlapping s, CF_3).

B) *mer*-[^{99m}Tc^{V}NCl$_2$(κ^4-*As*,*CC*,*As*-L^{iPr})]. An aqueous, saline generator eluate of Na^{99m}Tc^{VII}O$_4$ (50 µL, activity ca. 200-671 MBq) was mixed with conc. HCl$_{(aq.)}$ (32%, 5 mL). An aqueous solution of NaN$_3$ (50 mg, 0.8 mmol; in 125 µL) was added carefully. The mixture was heated at boiling temperature in an open vial for 20 minutes. After cooling to room temperature, an aqueous solution of NBu$_4$Cl (40 mg; in 500 µL) was added to the formed [^{99m}Tc^{VI}NCl$_4$]$^-$. The mixture was stirred without heating for 5 min. The formed [^{99m}Tc^{VI}NCl$_4$]$^-$ was extracted with methyl ethyl ketone (5 mL) by stirring the biphasic mixture for another 15 minutes at room temperature. The organic phase was transferred into a new reaction vial. L^{iPr} (68 µg, 150 nmol) was added to an aliquot of the thus obtained organic stock solution (2.1 mL) and the mixture was heated at boiling temperature in an open vial for 15 min forming the desired [^{99m}Tc^{V}NCl$_2$(κ^4-*As*,*CC*,*As*-L^{iPr})]. The product was purified *via* a pre-conditioned C$_{18}$ cartridge (pre-conditioning: 2 mL,CH$_3$CN:H$_2$O/25:75). The product solution was loaded on the cartridge by diluting an aliquot of the product mixture (300 µL) with water (600 µL). The product was purified by elution of impurities with CH$_3$CN and water (5 mL, CH$_3$CN:H$_2$O/25:75), while the purified product was eluted with CH$_3$CN (300 µL). Radiochemical yield: 32.40 MBq (based on decay-corrected activity at t_0 = 44.95 MBq: 47%)

S1.6 *cis,trans,mer*-[Tc^{V}N(CN)Cl(κ^4-*As*,*CC*,*As*-L^{iPr})]

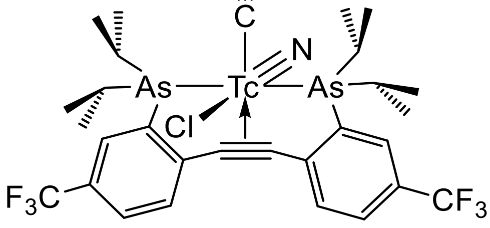

Me$_3$Si-CN (1 drop) was added to a solution of [TcNCl$_2$(κ^4-*As*,*CC*,*As*-L^{iPr})] (15 mg, 18 µmol) in THF (0.5 mL). The mixture was heated at reflux for 30 min and cooled to RT causing the formation of small colorless cubes of the product. Pentane (3 mL) was added to complete the precipitation and the crystals were filtered off, washed with hexane and dried. Colorless cubes. Yield: 13 mg (16 µmol, 89%). The crystals were suitable for X-ray diffraction. **FT-IR (KBr; transmission):** $\tilde{\nu}$ [cm^{-1}] = 2139 (vw, v, C≡N), 1956 (w, v, RC≡CR), 1038 (m, v, Tc≡N).**^{1}H NMR (399.4 MHz, CD_2Cl_2, 20°C):** δ (ppm) 8.44 (d, $^3J_{H\text{-}H}$ = 8.1 Hz, 2H, ^{Ar}H, *m*-As, *m*-CF$_3$, *o*-C≡C), 8.07 (s, 2H, ^{Ar}H, *o*-As, *o*-CF$_3$,

m-C≡C), 8.02 (d, $^3J_{H\text{-}H}$ = 8.3 Hz, 2H, ^{Ar}H, *p*-As, *o*-CF_3, *m*-C≡C), 3.54 (hept, $^3J_{H\text{-}H}$ = 7.2 Hz, 2H, C*H*(CH_3)$_2$, underneath equatorial plane; *anti* to Tc≡N), 3.31 (hept, $^3J_{H\text{-}H}$ = 7.0 Hz, 2H, C*H*(CH_3)$_2$, above equatorial plane; *syn* to Tc≡N), 1.81 (d, $^3J_{H\text{-}H}$ = 6.9 Hz, $^3J_{H\text{-}H}$ = 1.7 Hz, 6H, CH(C*H*$_3$)$_2$, above equatorial plane; *syn* to Tc≡N, pointed away from aromatic ring), 1.72 (pseudo t, $^3J_{H\text{-}H}$ = 6.4 Hz, 12H, CH(C*H*$_3$)$_2$, below equatorial plane; *anti* to Tc≡N, pointed away from aromatic ring & below equatorial plane; *anti* to Tc≡N, pointed towards aromatic ring), 1.11 (d, $^3J_{H\text{-}H}$ = 6.9 Hz, , 6H, CH(C*H*$_3$)$_2$, above equatorial plane; *syn* to Tc≡N, pointed towards aromatic ring). **^{19}F{^{1}H} NMR (375.8 MHz, CD_2Cl_2, 20°C)**: δ (ppm) -62.7 (s, C*F*$_3$).

S1.7 *mer*-[Re^{III}Cl$_3$(κ^4-*As*,*CC*,*As*-L^{iPr})]

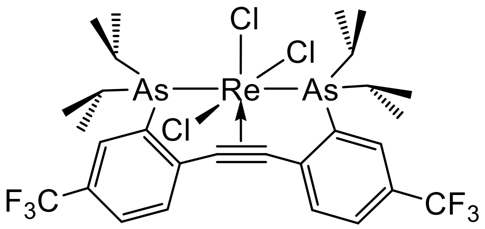

In an Argon-filled glovebox L^{iPr} (1.00 g, 1.58 mmol) and [ReCl$_3$(PPh$_3$)$_2$(NCCH$_3$)] (1.31 g, 1.53 mmol) were suspended in toluene (20 mL) in a PTFE-stopcock equipped ampoule. The reaction mixture was stirred at 110°C overnight. The reaction mixture was cooled to -35°C, and the crystalline product filtered off. The residue was washed with diethyl ether (20 mL) and pentane (20 mL) to yield grey-green crystals (1.16 g, 1.25 mmol, 82 %). **FT-IR (ATR):** $\tilde{\nu}$ [cm^{-1}] = 3056 (w), 2958 (m), 2921 (m), 2887 (m), 1597 (m), 1577 (w), 1494 (w), 1460 (m), 1390 (m), 1318 (vs), 1252 (m), 1228 (m), 1170 (m), 1161 (m), 1122 (vs), 1067 (vs), 1038 (m), 964 (w), 920 (m), 877 (m), 835 (s), 797 (w), 735 (s), 695 (m), 638 (m), 557 (m), 528 (m), 462 (m), 401 (m). **LIFDI+ (CH_2Cl_2):** m/z = 926.0006 ([M]$^+$ calcd. 925.9604). **^{1}H NMR (600.2 MHz, $CDCl_3$, 20°C):** δ (ppm) 8.55 (s, 2H, ^{Ar}H, *o*-As, *o*-CF_3, *m*-C≡C), 8.27 (d, $^3J_{H\text{-}H}$ = 8.0 Hz, 2H, ^{Ar}H, *p*-As, *o*-CF_3, *m*-C≡C), 7.80 (d, $^3J_{H\text{-}H}$ = 8.0 Hz, 2H, ^{Ar}H, *m*-As, *m*-CF_3, *o*-C≡C), 3.61 (hept, $^3J_{H\text{-}H}$ = 7.2 Hz, 4H, C*H*(CH_3)$_2$), 1.89 (d, $^3J_{H\text{-}H}$ = 7.2 Hz, 12H, CH(C*H*$_3$)$_2$, pointed away from aromatic ring), 1.77 (d, $^3J_{H\text{-}H}$ = 7.2 Hz, 12H, CH(C*H*$_3$)$_2$, pointed towards aromatic ring). **^{13}C{^{1}H} NMR (150.9 MHz, $CDCl_3$, 20°C):** δ (ppm) 174.0 (s, C≡C), 156.8 (s, ^{q}*C*^{Ar}-AsR$_2$), 137.5 (s, ^{q}*C*^{Ar}-C≡C), 132.1 (q, $^2J_{C\text{-}F}$ = 33 Hz, ^{q}*C*^{Ar}-CF_3), 129.2 (s, *C*^{Ar}-H, *m*-As, *m*-CF_3, *o*-C≡C), 128.0 (q, $^3J_{C\text{-}F}$ = 4 Hz, *C*^{Ar}-H, *o*-As, *o*-CF_3, *m*-C≡C), 126.0 (q, $^3J_{C\text{-}F}$ = 4 Hz, *C*^{Ar}-H, *p*-As, *o*-CF_3, *m*-C≡C), 121.8 (q, $^1J_{C\text{-}F}$ ≈ 273 Hz, *C*F$_3$), 31.0 (s, *C*H(CH_3)$_2$) 23.2 (s, CH(*C*H$_3$)$_2$, pointed towards aromatic ring), 21.2 (s, CH(*C*H$_3$)$_2$, pointed away from aromatic ring). **^{19}F{^{1}H} NMR (564.7 MHz, $CDCl_3$, 20°C)**: δ (ppm) -61.5 (s, C*F*$_3$).

S1.8 *mer*-[Re^{V}NCl$_2$(κ^4-*As*,*CC*,*As*-L^{iPr})]

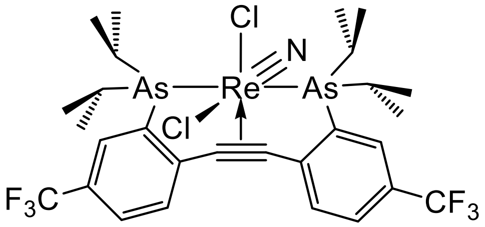

In an Argon-filled glovebox L^{iPr} (100 mg, 157 µmol) and [ReNCl$_2$(PPh$_3$)$_2$] (125 mg, 157 µmol) were suspended in dry acetonitrile (3 mL) in a PTFE-stopcock equipped ampoule. The orange reaction mixture was stirred for four days at 80°C gradually discoloring. After cooling to room temperature, the solvent was evaporated in vacuum. The residue was washed with diethyl ether (3 mL) and pentane (5 mL) to yield a

colorless powder (118 mg, 130 µmol, 83%). Single crystals suitable for X-ray diffraction were obtained by slow evaporation of a CH_2Cl_2 solution. **FT-IR (ATR):** $\tilde{\nu}$ [cm^{-1}] = 1899 (w, v, RC≡CR), 1054 (m, v, Re≡N). **ESI+ (CH_2Cl_2/MeOH):** m/z = 870.0271 ($[M-Cl]^+$ calcd. 870.0270). **^1H NMR (600.2 MHz, $CDCl_3$, 20°C):** δ (ppm) 8.31 (d, $^3J_{H-H}$ = 8.0 Hz, 2H, ^{Ar}H, *m*-As, *m*-CF_3, *o*-C≡C), 7.95 (s, 2H, ^{Ar}H, *o*-As, *o*-CF_3, *m*-C≡C), 7.93 (d, $^3J_{H-H}$ = 7.7 Hz, 2H, ^{Ar}H, *p*-As, *o*-CF_3, *m*-C≡C), 3.77 (hept, $^3J_{H-H}$ = 7.3 Hz, 2H, $CH(CH_3)_2$, underneath equatorial plane; *anti* to Re≡N), 3.29 (hept, $^3J_{H-H}$ = 6.9 Hz, 2H, $CH(CH_3)_2$, above equatorial plane; *syn* to Re≡N), 1.82 (d, $^3J_{H-H}$ = 6.9 Hz, 6H, $CH(CH_3)_2$, above equatorial plane; *syn* to Re≡N, pointed away from aromatic ring), 1.77 (d, $^3J_{H-H}$ = 7.3 Hz, 6H, $CH(CH_3)_2$, below equatorial plane; *anti* to Re≡N, pointed away from aromatic ring), 1.62 (dd, $^3J_{H-H}$ = 7.2 Hz, $^3J_{H-H}$ = 1.8 Hz, 6H, $CH(CH_3)_2$, below equatorial plane; *anti* to Re≡N, pointed towards aromatic ring), 1.20 (d, $^3J_{H-H}$ = 7.0 Hz, 6H, $CH(CH_3)_2$, above equatorial plane; *syn* to Re≡N, pointed towards aromatic ring). **$^{13}C\{^1H\}$ NMR (150.9 MHz, $CDCl_3$, 20°C):** δ (ppm) 149.3 (s, $^qC^{Ar}$-AsR_2), 142.2 (s, $^qC^{Ar}$-C≡C), 134.3 (s, C^{Ar}-H, *m*-As, *m*-CF_3, *o*-C≡C), 131.5 (q, $^2J_{C-F}$ = 33 Hz, $^qC^{Ar}$-CF_3), 130.6 (q, $^3J_{C-F}$ = 4 Hz, C^{Ar}-H, *o*-As, *o*-CF_3, *m*-C≡C), 128.8 (q, $^3J_{C-F}$ = 4 Hz, C^{Ar}-H, *p*-As, *o*-CF_3, *m*-C≡C), 123.5 (q, $^1J_{C-F}$ ≈ 273 Hz, CF_3), 121.5 (s, C≡C), 31.7 (s, $CH(CH_3)_2$, underneath equatorial plane; *anti* to Re≡N), 30.2 (s, $CH(CH_3)_2$, above equatorial plane; *syn* to Re≡N), 21.8 (s, $CH(CH_3)_2$, below equatorial plane; *anti* to Re≡N, pointed towards aromatic ring), 21.2 (s, $CH(CH_3)_2$, below equatorial plane; *anti* to Re≡N, pointed away from aromatic ring), 20.6 (s, $CH(CH_3)_2$, above equatorial plane; *syn* to Re≡N, pointed away from aromatic ring), 19.3 (s, $CH(CH_3)_2$, above equatorial plane; *syn* to Re≡N, pointed towards aromatic ring). **$^{19}F\{^1H\}$ NMR (564.7 MHz, $CDCl_3$, 20°C)**: δ (ppm) -62.6 (2 overlapping s, CF_3).

Part S2: Crystallographic data

Absorption correction was performed using SADABS,[104] based on the Laue symmetry of the reciprocal space, structures were solved with SHELXT-2018/2 (Sheldrick 2015),[105] and refined against F2 with a full-matrix least-squares algorithm using the SHELXL-2018/3 (Sheldrick, 2018) software.[106] The Bruker programs APEX, APEX2, SMART, SAINT, SAINT-Plus were used for data processing.[107] CCDC 2521631 to CCDC 2521634 and CCDC 2521636 to CCDC 2521640 contain the supplementary X-ray crystallographic data for this paper. Mercury was used to generate the structure pictures.[108] Structure solution, refinement and finalization was done using OLEX2.[109] The data can be obtained free of charge from The Cambridge Crystallographic Data Centre via www.ccdc.cam.ac.uk/structures.

S2.1 Crystal data and structure determination parameters.

Table S1. Crystal data and structure determination parameters.

Name	*mer*-[$Tc^{V}NBr_2$(*As*,*CC*,*As*-L^{iPr})]	*mer*-[$Tc^{V}NCl_2$(*As*,*CC*,*As*-L^{iPr})]	*mer*-[$Tc^{V}N(CN)Cl$(*As*,*CC*,*As*-L^{iPr})]
Chemical formula	$C_{28}H_{34}As_2Br_2F_6NTc$	$C_{28}H_{34}As_2Cl_2F_6NTc$	$C_{29}H_{34}As_2ClF_6N_2Tc$
M_r	906.22	817.3	807.87
Crystal system, space group	Orthorhombic, *Pbca*	Orthorhombic, *Pbca*	Orthorhombic, *Pbca*
Temperature (K)	125	100	100
a, *b*, *c* (Å)	14.5030(11), 14.6529(12), 31.153(2)	14.2175(6), 14.6633(7), 30.5523(15)	14.5954(5), 14.3348(6), 30.8016(13)
α, β, γ (°)	90, 90, 90	90, 90, 90	90, 90, 90
V (Å^3)	6620.4(9)	6369.4(5)	6444.4(4)
Z	8	8	8
Radiation type	Mo *K*α	Mo *K*α	Mo *K*α
μ (mm^{-1})	4.89	2.74	2.62
Crystal size (mm)	0.20 × 0.15 × 0.15	0.65 × 0.41 × 0.40	0.09 × 0.09 × 0.03
Diffractometer	Bruker *APEX*-II CCD	Bruker *APEX*-II CCD	Bruker *APEX*-II CCD
Absorption correction	Multi-scan	Multi-scan	Multi-scan
T_{min}, T_{max}	0.451, 0.745	0.250, 0.396	0.642, 0.746
No. of measured, independent and observed [$I > 2\sigma(I)$] reflections	78191, 7041, 6005	120035, 10149, 9056	69254, 7997, 6044
R_{int}	0.054	0.038	0.054
$(\sin \theta/\lambda)_{max}$ (Å^{-1})	0.633	0.726	0.667
$R[F^2 > 2\sigma(F^2)]$, $wR(F^2)$, S	0.034, 0.098, 1.07	0.032, 0.077, 1.09	0.042, 0.114, 1.19
No. of reflections	7041	10149	7997
No. of parameters	357	369	406
No. of restraints	60	0	21
H-atom treatment	H-atom parameters constrained<br>$w = 1/[\sigma^2(F_o^2) + (0.0506P)^2 + 19.3509P]$<br>where $P = (F_o^2 + 2F_c^2)/3$	H-atom parameters constrained<br>$w = 1/[\sigma^2(F_o^2) + (0.0225P)^2 + 18.0174P]$<br>where $P = (F_o^2 + 2F_c^2)/3$	H-atom parameters constrained<br>$w = 1/[\sigma^2(F_o^2) + (0.0306P)^2 + 32.7853P]$<br>where $P = (F_o^2 + 2F_c^2)/3$
$\Delta\rho_{max}$, $\Delta\rho_{min}$ (e Å^{-3})	1.04, −1.08	1.13, −0.82	1.25, −0.94
CCDC number	2521631	2521632	2521633

Table S1. Crystal data and structure determination parameters (continued).

Name	*mer*-[$Tc^{III}Cl_3$(*As*,*CC*,*As*-L^{iPr})] ·0.5CH_2Cl_2	*mer*-[$Tc^{III}Cl_3$(*As*,*CC*,*As*-L^{iPr})] ·$OPPh_3$	*mer*-[$Re^{V}NCl_2$(*As*,*CC*,*As*-L^{iPr})]
Chemical formula	$C_{28}H_{34}As_2Cl_3F_6Tc\cdot 0.5(CH_2Cl_2)$	$C_{28}H_{34}As_2Cl_3F_6Tc\cdot C_{18}H_{15}OP$	$C_{28}H_{34}As_2Cl_2F_6NRe$
M_r	881.2	1117.01	905.5
Crystal system, space group	Triclinic, $P\bar{1}$	Triclinic, $P\bar{1}$	Orthorhombic, *Pbca*
Temperature (K)	125	125	120
a, *b*, *c* (Å)	8.4937(9), 14.9163(16), 26.902(3)	8.954(3), 13.837(4), 20.288(6)	14.2644(1), 14.6871(1), 30.6259(2)
α, β, γ (°)	86.226(3), 86.724(3), 76.053(4)	86.296(11), 80.820(12), 82.146(12)	90, 90, 90
V ($Å^3$)	3297.7(6)	2455.8(12)	6416.21(8)
Z	4	2	8
Radiation type	Mo *K*α	Mo *K*α	Cu *K*α
μ (mm^{-1})	2.81	1.88	11.72
Crystal size (mm)	0.13 × 0.12 × 0.10	0.1 × 0.05 × 0.05	0.16 × 0.12 × 0.08
Diffractometer	Bruker *APEX*-II CCD	Bruker *APEX*-II CCD	SuperNova, Dual, Cu at home/near, Eos
Absorption correction	Multi-scan	Multi-scan	Gaussian
T_{min}, T_{max}	0.563, 0.745	0.495, 0.745	0.301, 0.646
No. of measured, independent and observed [$I > 2\sigma(I)$] reflections	11212, 11212, 8592	28572, 8933, 2924	119475, 6172, 6095
R_{int}	Three component twin	0.246	0.06
$(\sin \theta/\lambda)_{max}$ ($Å^{-1}$)	0.606	0.604	0.614
$R[F^2 > 2\sigma(F^2)]$, $wR(F^2)$, S	0.080, 0.212, 1.04	0.169, 0.482, 1.14	0.024, 0.063, 1.15
No. of reflections	11212	8933	6172
No. of parameters	766	542	389
No. of restraints	936	662	46
H-atom treatment	H-atom parameters constrained	H-atom parameters constrained	H-atom parameters constrained
	$w = 1/[\sigma^2(F_o^2) + (0.0733P)^2 + 66.7622P]$ where $P = (F_o^2 + 2F_c^2)/3$	$w = 1/[\sigma^2(F_o^2) + (0.2P)^2]$ where $P = (F_o^2 + 2F_c^2)/3$	$w = 1/[\sigma^2(F_o^2) + (0.0294P)^2 + 10.8648P]$ where $P = (F_o^2 + 2F_c^2)/3$
$\Delta\rho_{max}$, $\Delta\rho_{min}$ (e $Å^{-3}$)	1.16, −1.22	4.50, −1.34	0.70, −0.62
CCDC number	2521634	Not deposited; poor data quality	2521636

Table S1. Crystal data and structure determination parameters (continued).

Name	*mer*-[$Re^{V}Cl_3$(*As*,*CC*,*As*-L^{iPr})]	($L^{iPr}(OH)_2)_2[Tc^{V}N(NCS)_4(THF)]Cl_2$·3THF	Cyclization product of L^{tBu}
Chemical formula	$C_{28}H_{34}As_2Cl_3F_6Re$	$2(C_{28}H_{36}As_2F_6O_2)\cdot C_4N_5S_4Tc\cdot 4(C_4H_8O)\cdot 2(Cl)$	$C_{24}H_{24}As_2F_6$
M_r	926.94	2040.46	576.27
Crystal system, space group	Monoclinic, *I*2/*a*	Triclinic, $P\bar{1}$	Triclinic, $P\bar{1}$
Temperature (K)	120	125	125
a, *b*, *c* (Å)	20.4923(3), 12.7556(1), 25.8354(3)	11.6095(5), 17.3910(8), 24.2886(11)	10.184(3), 11.730(4), 11.837(3)
α, β, γ (°)	90, 109.329(2), 90	71.299(2), 86.959(2), 77.894(2)	113.293(11), 96.330(11), 109.067(11)
V ($Å^3$)	6372.50(15)	4541.2(4)	1180.0(6)
Z	8	2	2
Radiation type	Mo Kα	Mo Kα	Mo Kα
μ (mm^{-1})	6.18	1.83	2.89
Crystal size (mm)	0.19 × 0.17 × 0.15	0.41 × 0.2 × 0.11	0.3 × 0.2 × 0.2
Diffractometer	SuperNova, Dual, Cu at home/near, Eos	Bruker APEX-II CCD	Bruker APEX-II CCD
Absorption correction	Gaussian	Multi-scan	Multi-scan
T_{min}, T_{max}	0.640, 1.000	0.556, 0.694	0.252, 0.491
No. of measured, independent and observed [$I > 2\sigma(I)$] reflections	58442, 12513, 10089	120510, 19289, 14845	45446, 5293, 4118
R_{int}	0.063	0.045	0.091
$(\sin \theta/\lambda)_{max}$ ($Å^{-1}$)	0.793	0.633	0.651
$R[F^2 > 2\sigma(F^2)]$, $wR(F^2)$, *S*	0.033, 0.069, 1.03	0.048, 0.137, 1.06	0.057, 0.177, 1.04
No. of reflections	12513	19289	5293
No. of parameters	370	1125	323
No. of restraints	0	1246	39
H-atom treatment	H-atom parameters constrained	H atoms treated by a mixture of independent and constrained refinement	H-atom parameters constrained
	w = 1/[σ2(Fo2) + (0.0211P)2]	w = 1/[σ2(Fo2) + (0.0553P)2 + 13.6537P]	w = 1/[σ2(Fo2) + (0.1222P)2 + 1.5985P]
	where P = (Fo2 + 2Fc2)/3	where P = (Fo2 + 2Fc2)/3	where P = (Fo2 + 2Fc2)/3
$\Delta\rho_{max}$, $\Delta\rho_{min}$ (e $Å^{-3}$)	1.68, −1.57	1.70, −1.28	2.00, −1.79
CCDC number	2521637	2521638	2521639

Table S1. Crystal data and structure determination parameters (continued).

Name	[Ag^{I}(*As*,*CC*,*As*-L^{iPr})](BF$_4$)
Chemical formula	$C_{28}H_{34}AgAs_2F_6 \cdot BF_4$
M_r	829.07
Crystal system, space group	Monoclinic, $P2_1/c$
Temperature (K)	150
a, *b*, *c* (Å)	14.8020(7), 11.1539(5), 20.8655(10)
α, β, γ (°)	110.347(2)
V (Å^3)	3229.9(3)
Z	4
Radiation type	Mo Kα
μ (mm^{-1})	2.73
Crystal size (mm)	0.14 × 0.12 × 0.11
Diffractometer	Bruker APEX-II CCD
Absorption correction	Multi-scan
T_{min}, T_{max}	0.533, 0.746
No. of measured, independent and observed [$I > 2\sigma(I)$] reflections	75598, 7140, 5509
R_{int}	0.058
$(\sin \theta/\lambda)_{max}$ (Å^{-1})	0.642
$R[F^2 > 2\sigma(F^2)]$, $wR(F^2)$, S	0.054, 0.140, 1.07
No. of reflections	7140
No. of parameters	403
No. of restraints	3
H-atom treatment	H-atom parameters constrained<br>w = 1/[σ2(Fo2) + (0.0362P)2 + 27.6335P]<br>where P = (Fo2 + 2Fc2)/3
$\Delta\rho_{max}$, $\Delta\rho_{min}$ (e Å^{-3})	1.38, −1.23
CCDC number	2521640

S2.1 *mer*-[Tc^{V}NBr$_2$(κ^4-*As*,*CC*,*As*-L^{iPr})].

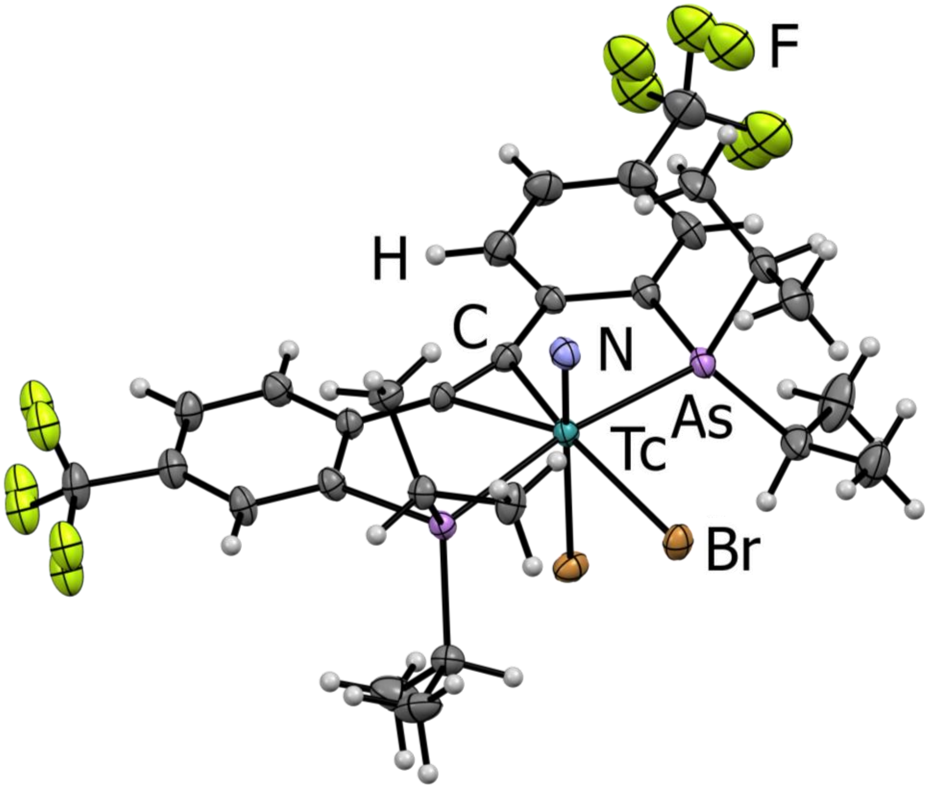

Figure S1. Ellipsoid representation (50% probability) of *mer*-[Tc^{V}NBr$_2$(κ^4-*As*,*CC*,*As*-L^{iPr})]. Further labels are omitted for clarity. The fluorine atoms of the CF$_3$ groups are disordered over two positions.

S2.2 *mer*-[$Tc^{V}NCl_2$(κ^4-*As*,*CC*,*As*-L^{iPr})].

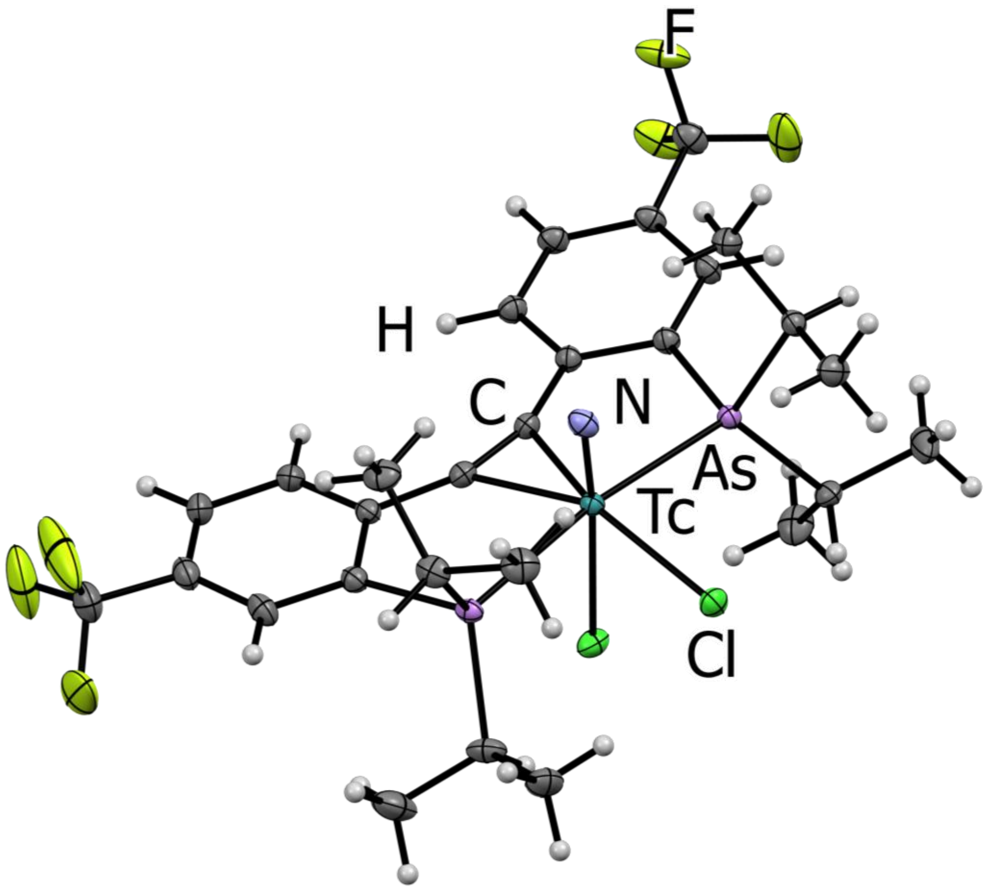

Figure S2. Ellipsoid representation (50% probability) of *mer*-[$Tc^{V}NCl_2$(κ^4-*As*,*CC*,*As*-L^{iPr})]. Further labels are omitted for clarity.

S2.3 *cis*,*trans*,*mer*-[Tc^{V}N(CN)Cl(κ^{4}-*As*,*CC*,*As*-L^{iPr})].

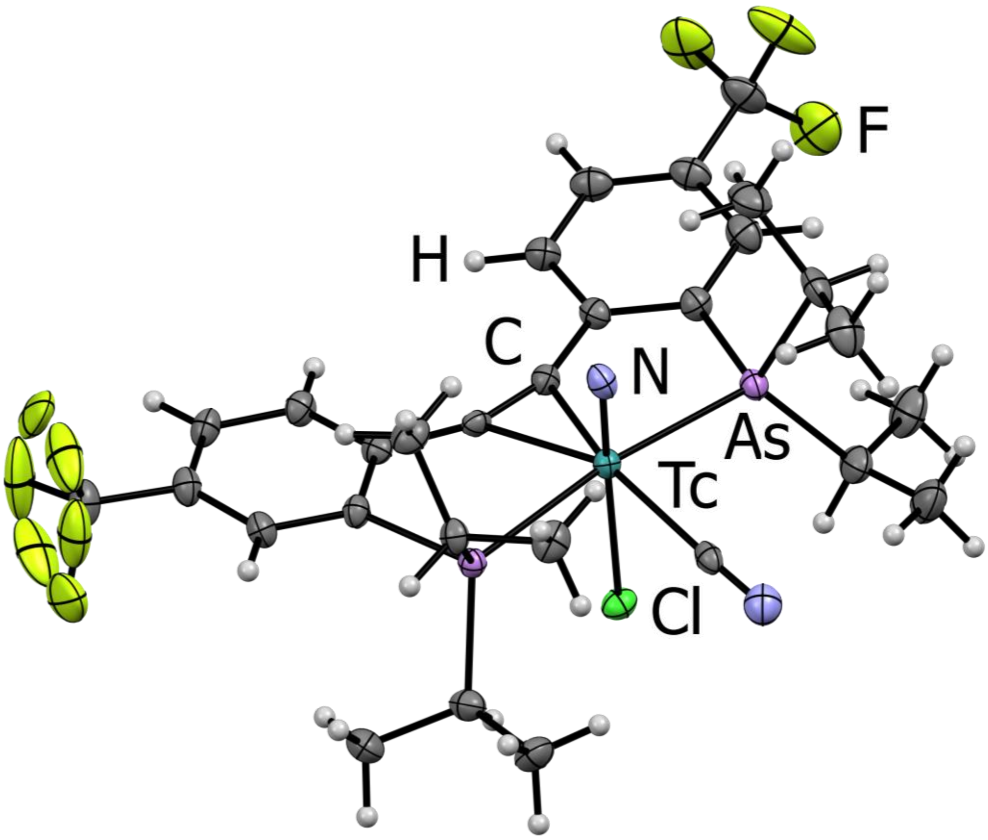

Figure S3. Ellipsoid representation (50% probability) of *mer*-[Tc^{V}N(CN)Cl(κ^{4}-*As*,*CC*,*As*-L^{iPr})]. Further labels are omitted for clarity. The fluorine atoms of one CF_3 group are disordered over two positions.

S2.4 *mer*-[$Tc^{III}Cl_3$(κ^4-*As*,*CC*,*As*-L^{iPr})].

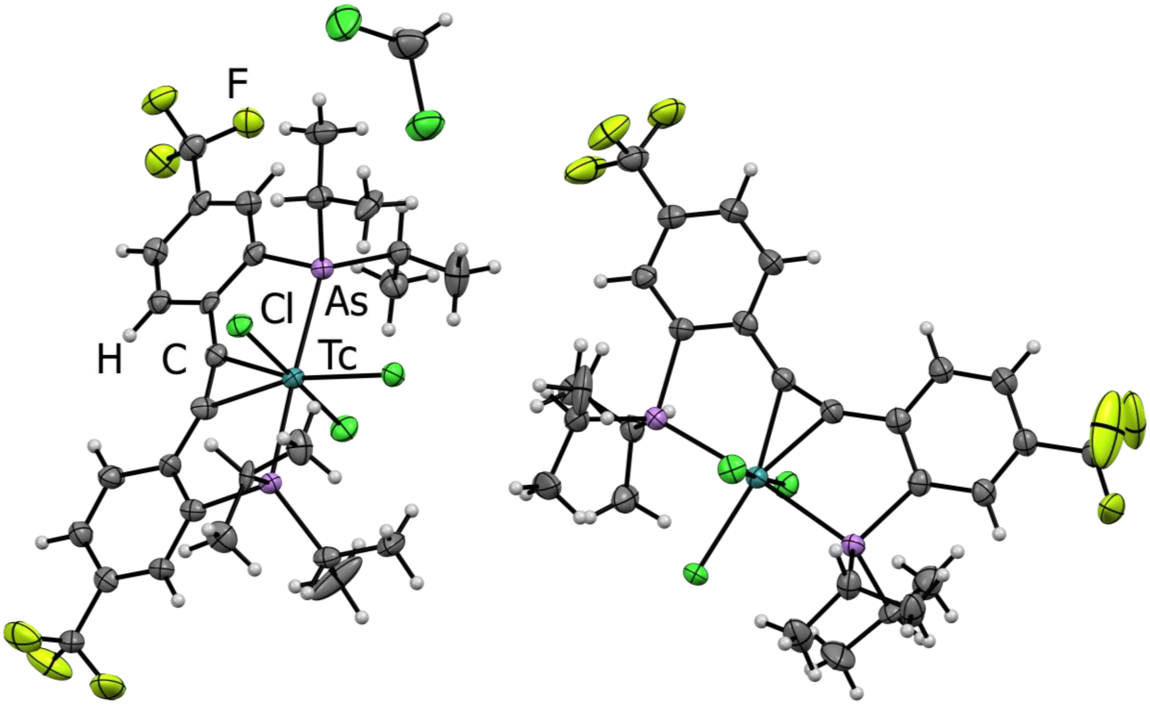

Figure S4. Ellipsoid representation (50% probability) of *mer*-[$Tc^{III}Cl_3$(κ^4-*As*,*CC*,*As*-L^{iPr})]·0.5CH_2Cl_2. Further labels are omitted for clarity. Two independent molecules are found in the asymmetric unit.

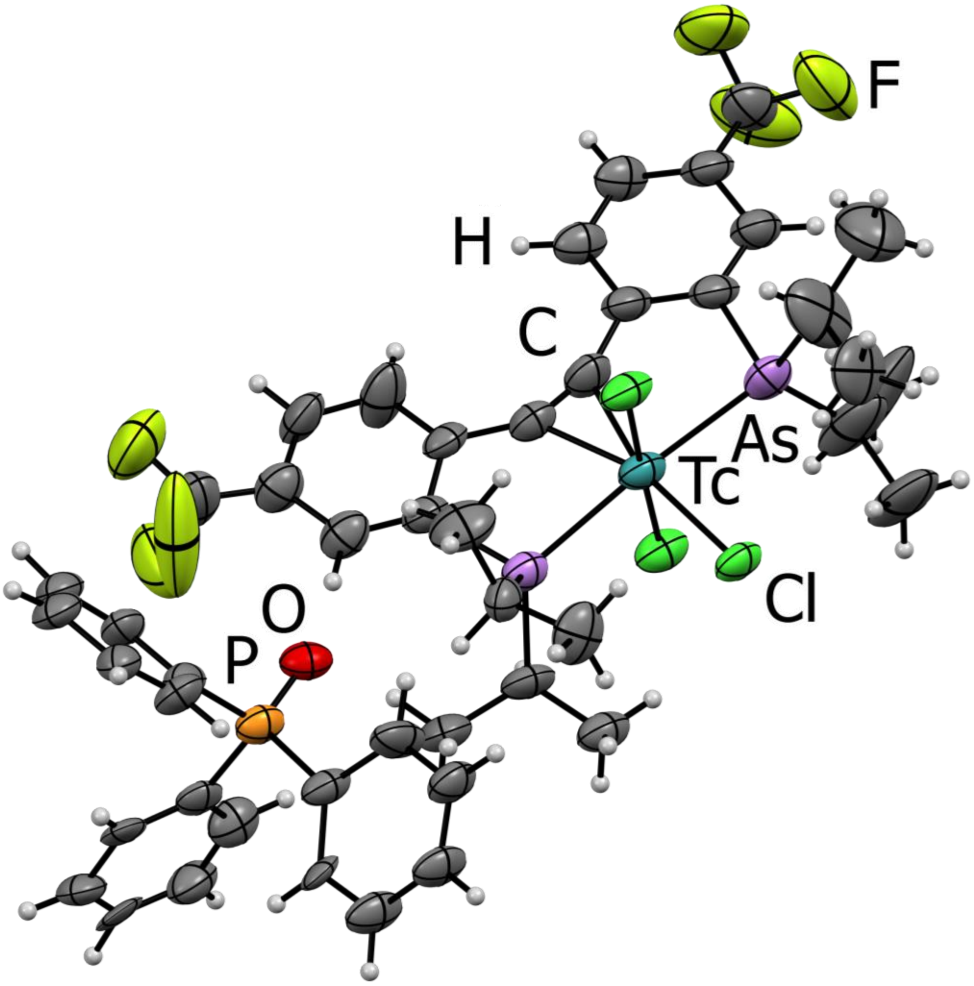

Figure S5. Ellipsoid representation (50% probability) of *mer*-[$Tc^{III}Cl_3$(κ^4-*As*,*CC*,*As*-L^{iPr})]·$OPPh_3$. Further labels are omitted for clarity.

S2.5 *mer*-[$Re^{V}NCl_2$(κ^4-*As*,*CC*,*As*-L^{iPr})].

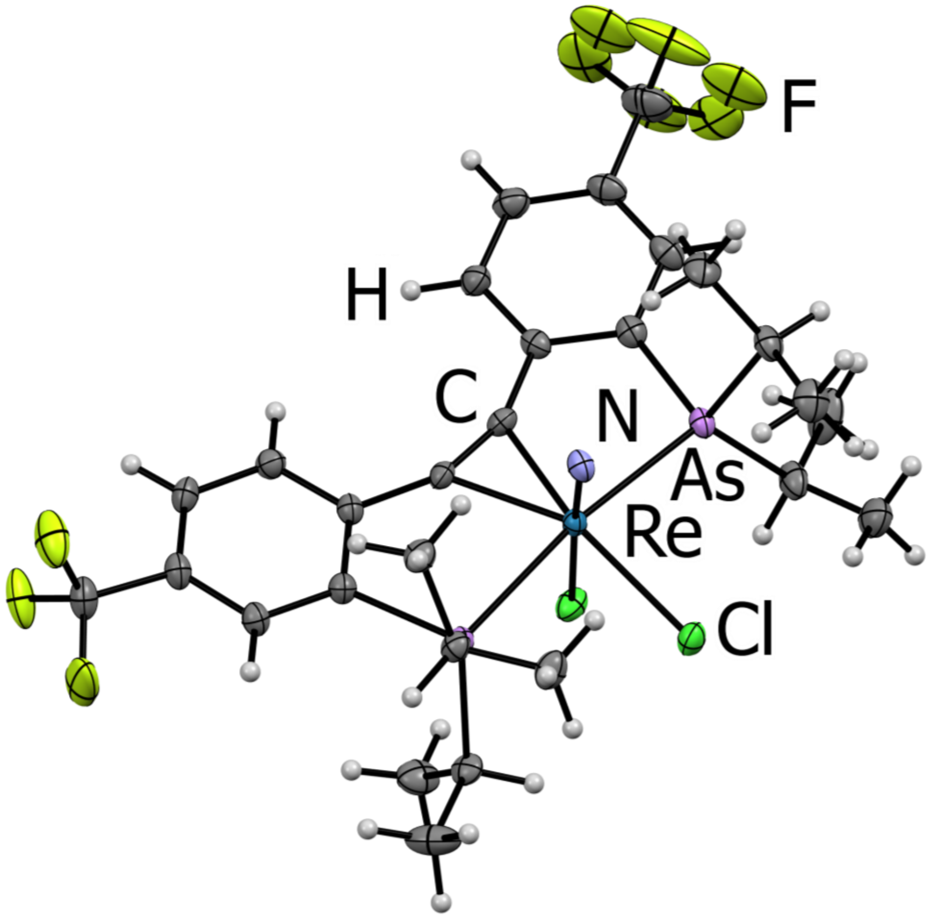

Figure S6. Ellipsoid representation (50% probability) of *mer*-[$Re^{V}NCl_2$(κ^4-*As*,*CC*,*As*-L^{iPr})]. Further labels are omitted for clarity. The fluorine atoms of one CF_3 group are disordered over two positions.

S2.6 *mer*-[Re^{V}Cl$_3$(κ^4-*As*,*CC*,*As*-L^{iPr})].

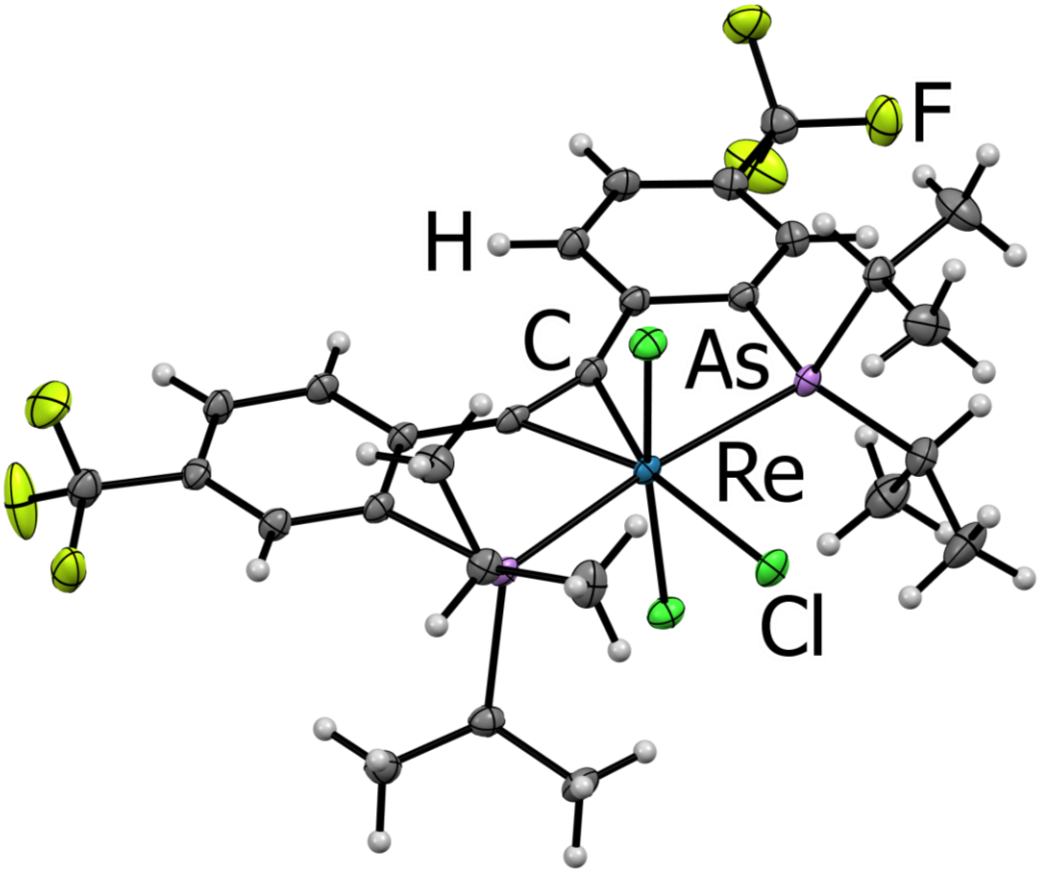

Figure S7. Ellipsoid representation (50% probability) of *mer*-[Re^{V}Cl$_3$(κ^4-*As*,*CC*,*As*-L^{iPr})]. Further labels are omitted for clarity.

S2.7 (L^{iPr}(OH)$_2$)$_2$[Tc^{V}N(NCS)$_4$(THF)]Cl$_2$·3THF.

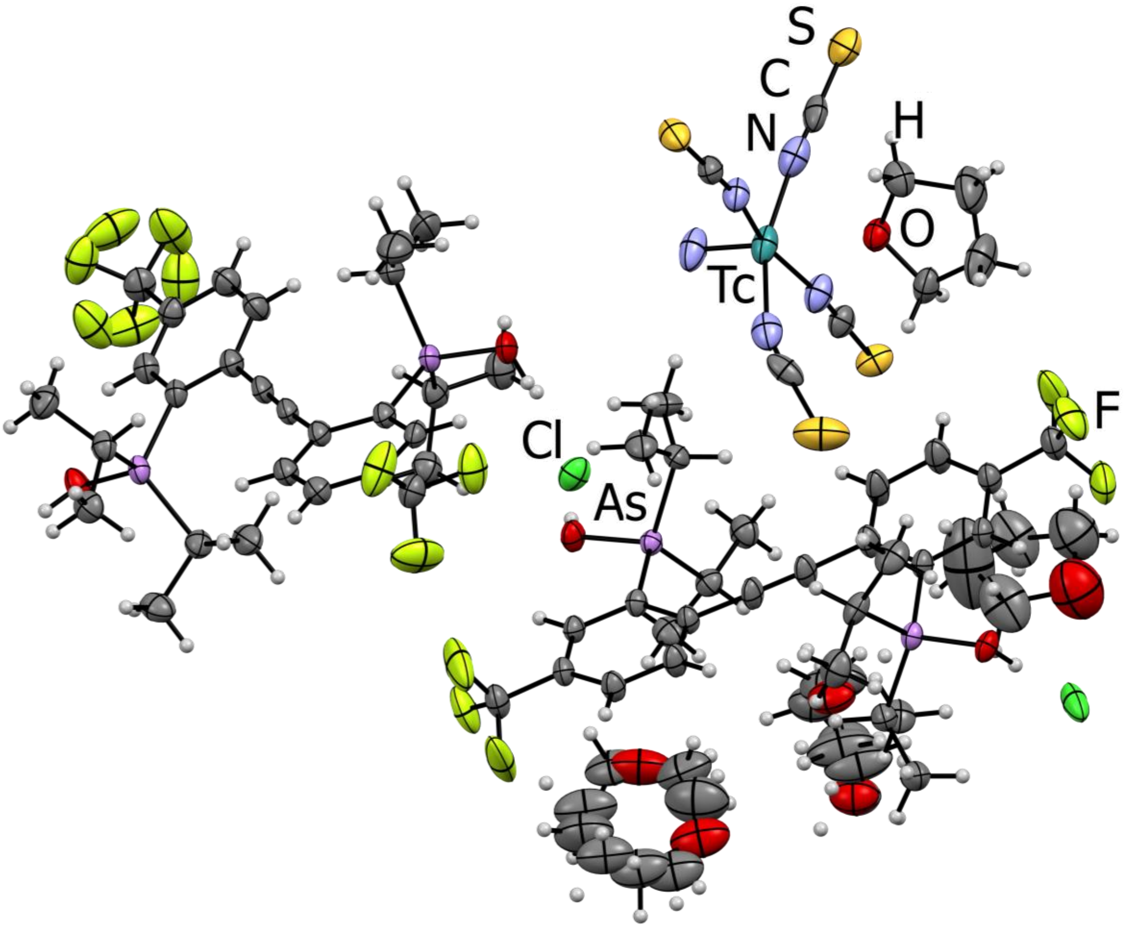

Figure S8. Ellipsoid representation (50% probability) of (L^{iPr}(OH)$_2$)$_2$[Tc^{V}N(NCS)$_4$(THF)]Cl$_2$·3THF. Further labels are omitted for clarity. Two of the co-crystallized solvent molecules (THF) and one of the CF$_3$ groups are disordered over two positions each.

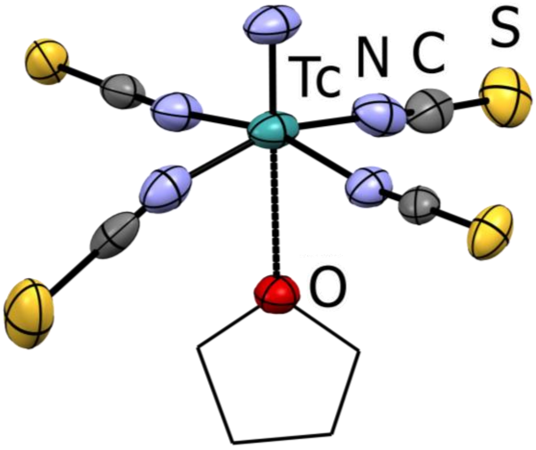

Figure S9. Ellipsoid representation (50% probability) of the anion $[Tc^{V}N(NCS)_4(THF)]^{2-}$ in $(L^{iPr}(OH)_2)_2[Tc^{V}N(NCS)_4(THF)]Cl_2{\cdot}3THF$. Hydrogen atoms and further labels are omitted and some skeletal carbon atoms are shown as wireframe for clarity.

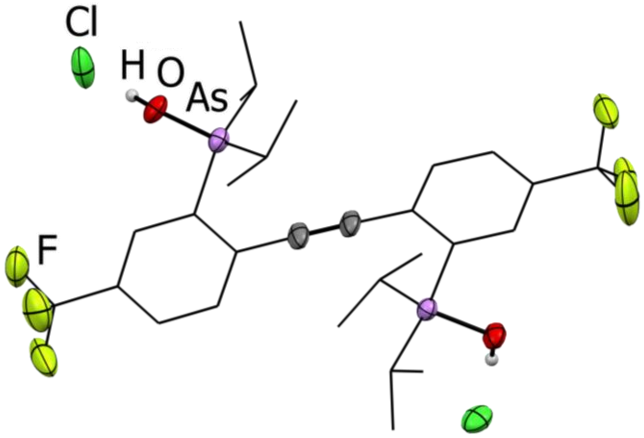

Figure S10. Ellipsoid representation (50% probability) of the $(L^{iPr}(OH)_2)Cl_2$ subunit in $(L^{iPr}(OH)_2)_2[Tc^{V}N(NCS)_4(THF)]Cl_2{\cdot}3THF$. Further hydrogen atoms and labels are omitted and some skeletal carbon atoms are shown as wireframe for clarity.

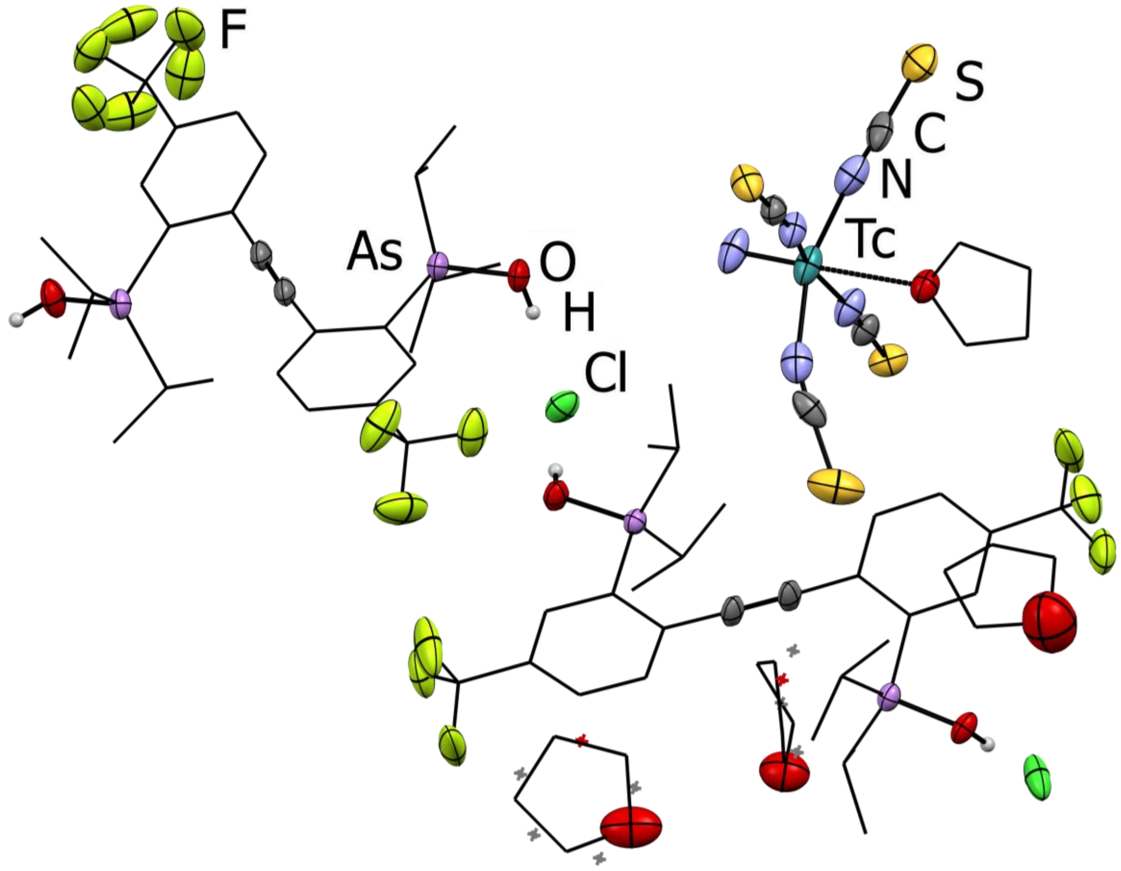

Figure S11. Ellipsoid representation (50% probability) of $(L^{iPr}(OH)_2)_2[Tc^VN(NCS)_4(THF)]Cl_2·3THF$. Further labels are omitted and some skeletal carbon atoms are shown as wireframe for clarity. Two of the co-crystallized solvent molecules (THF) and one of the CF_3 groups are disordered over two positions each.

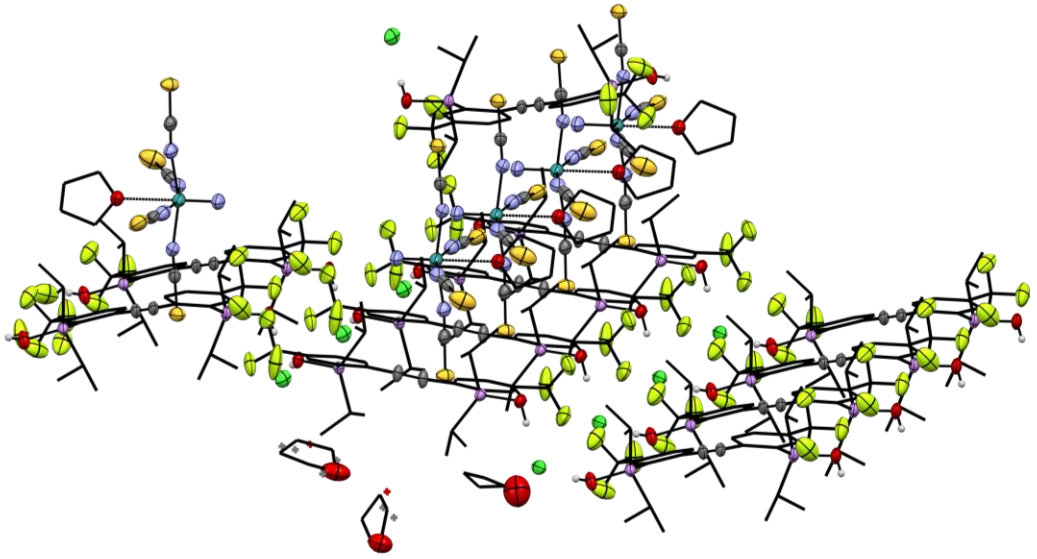

Figure S12. Packing of $(L^{iPr}(OH)_2)_2[Tc^VN(NCS)_4(THF)]Cl_2·3THF$, view 1. Further hydrogen atoms and labels are omitted and some skeletal carbon atoms are shown as wireframe for clarity.

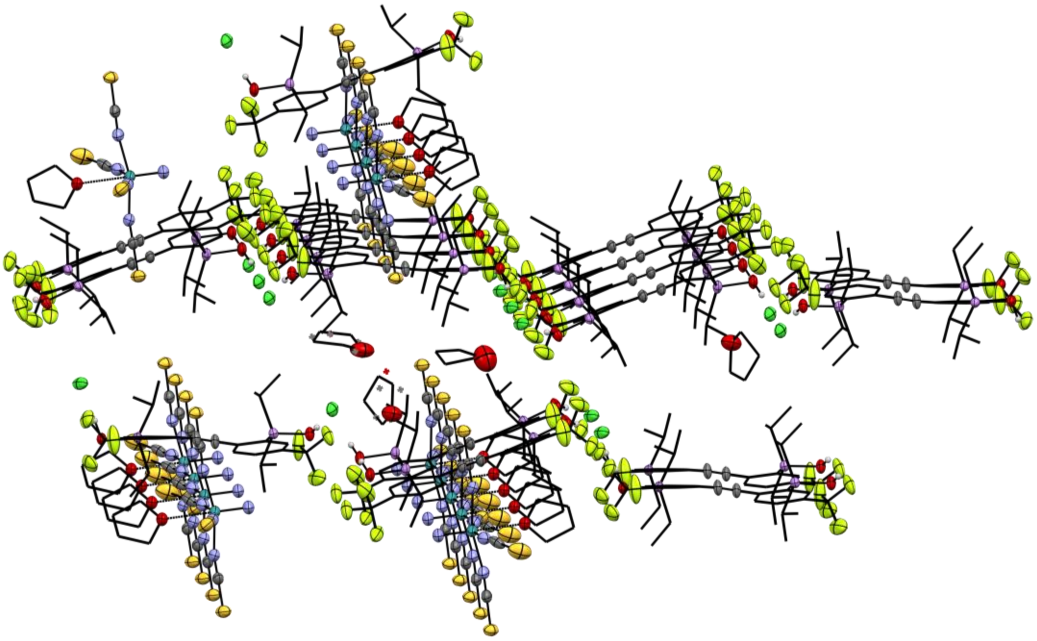

Figure S13. Packing of $(L^{iPr}(OH)_2)_2[Tc^VN(NCS)_4(THF)]Cl_2{\cdot}3THF$, view 2. The assembly of $\{(L^{iPr}(OH)_2)_2Cl_2\}^{2+}$ sheets intercepted by layers of solvent accessible voids filled with THF as well as infinite chains of S···S contacting $[Tc^VN(NCS)_4(THF)]^{2-}$ molecules is highlighted. Further hydrogen atoms and labels are omitted and some skeletal carbon atoms are shown as wireframe for clarity.

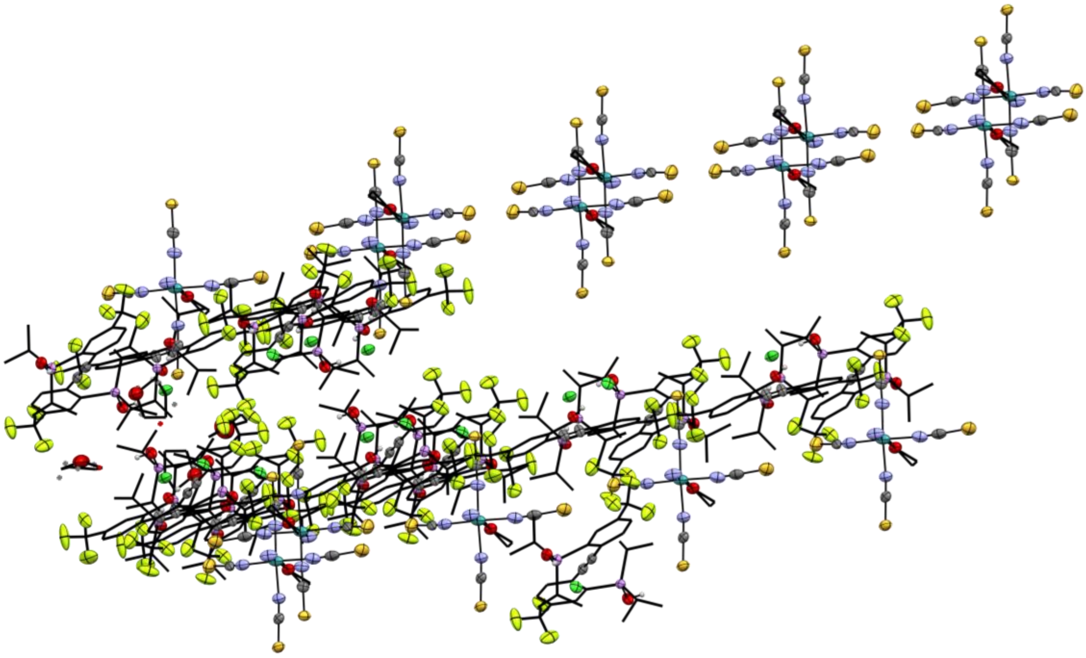

Figure S14. Packing of $(L^{iPr}(OH)_2)_2[Tc^VN(NCS)_4(THF)]Cl_2{\cdot}3THF$, view 3. The assembly of the parallelly oriented infinite chains of S···S contacting $[Tc^VN(NCS)_4(THF)]^{2-}$ molecules is highlighted. Hydrogen atoms and labels are omitted and some skeletal carbon atoms are shown as wireframe for clarity.

S2.8 Cyclization product of L^{tBu}.

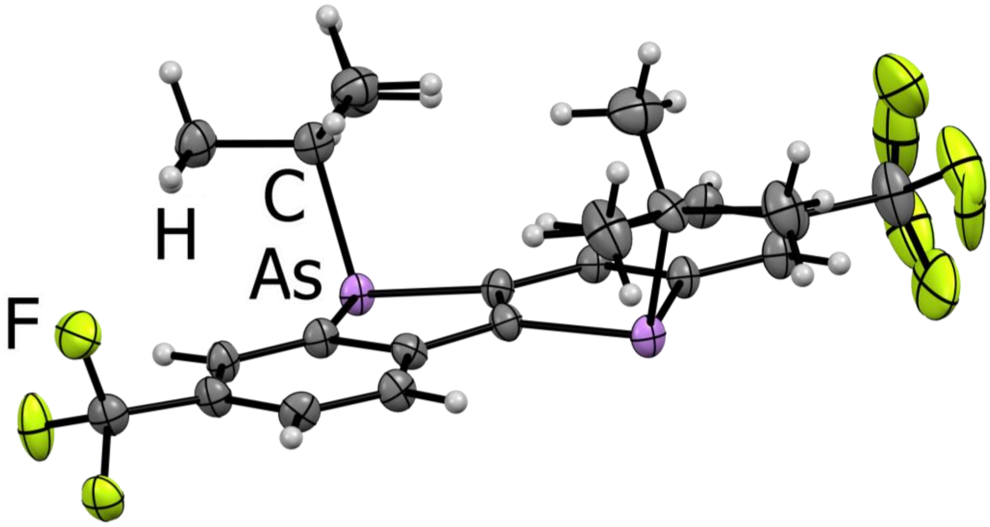

Figure S15. Ellipsoid representation (50% probability) of the cyclization product of L^{tBu}. Further labels are omitted for clarity. The fluorine atoms of one CF_3 group are disordered over two positions.

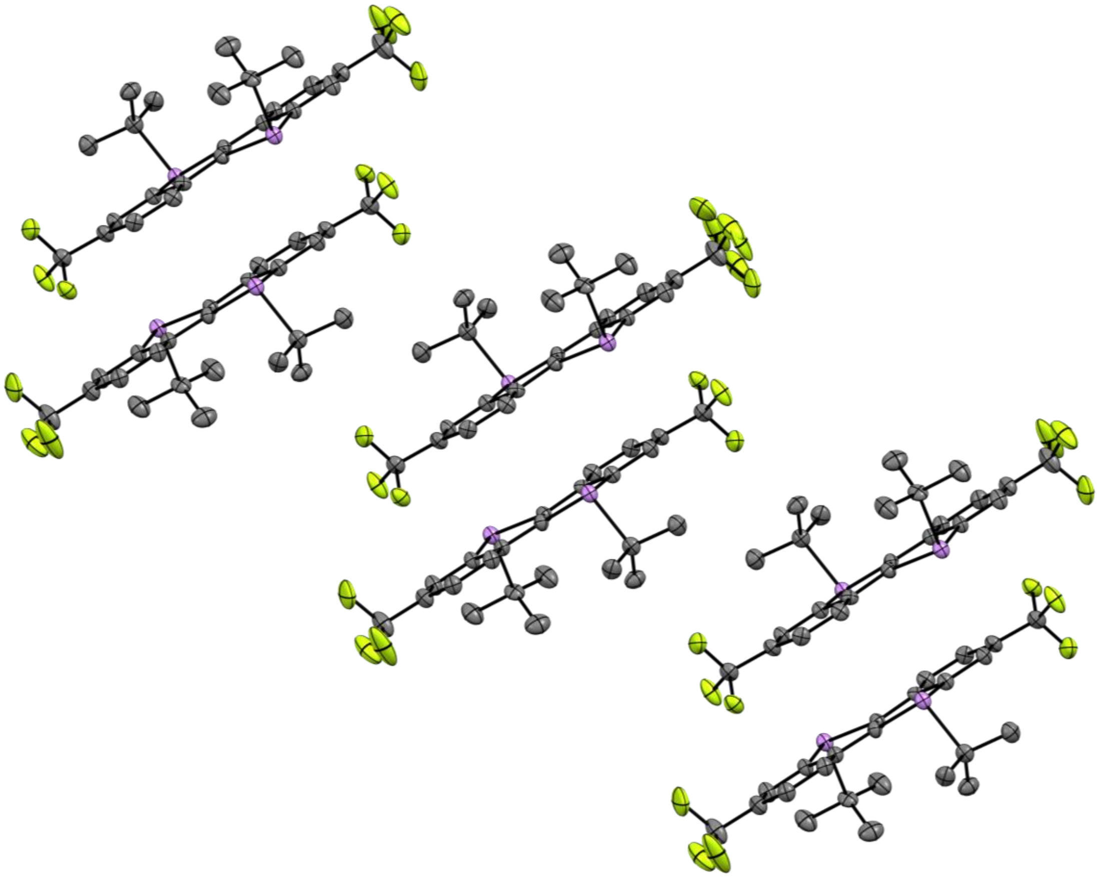

Figure S16. Packing of the cyclization product of L^{tBu} highlighting a dimeric, π-stacked arrangement. Hydrogen atoms and labels are omitted for clarity.

S2.9 [Ag^{I}(κ^{2}-*As*,*CC*,*As*-L^{iPr})](BF$_4$).

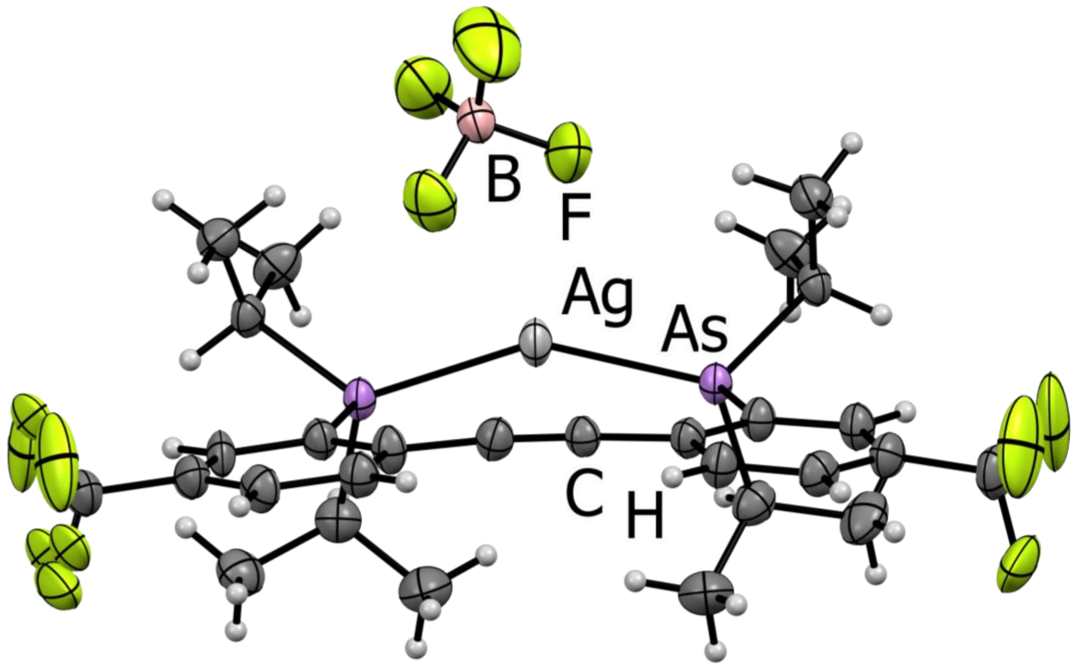

Figure S17. Ellipsoid representation (50% probability) of [Ag^{I}(κ^{2}-*As*,*CC*,*As*-L^{iPr})](BF$_4$). Further labels are omitted for clarity.

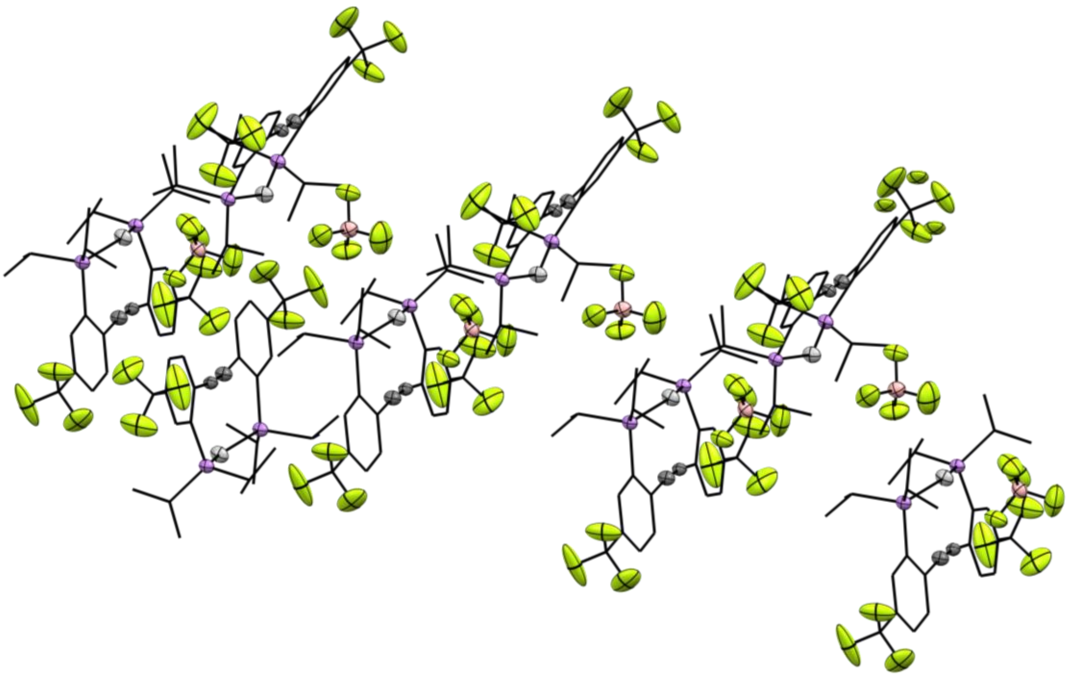

Figure S18. Packing of [Ag^{I}(κ^{2}-*As*,*CC*,*As*-L^{iPr})](BF$_4$). Hydrogen atoms and labels are omitted and some skeletal carbon atoms are shown as wireframe for clarity.

Part 3: Spectral data

S3.1 ^{1}H & ^{13}C NMR assignment overview

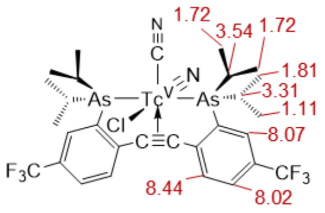

[Tc^{V}N(CN)Cl(κ^{4}-*As*,*CC*,*As*-L^{iPr})]

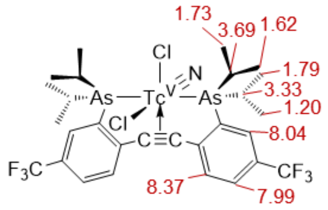

[Tc^{V}NCl$_{2}$(κ^{4}-*As*,*CC*,*As*-L^{iPr})]

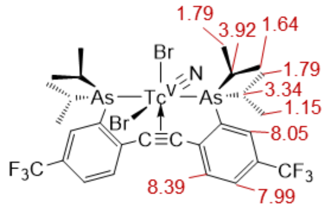

[Tc^{V}NBr$_{2}$(κ^{4}-*As*,*CC*,*As*-L^{iPr})]

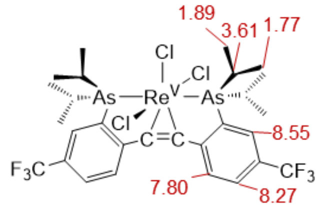

[Re^{V}Cl$_{3}$(κ^{4}-*As*,*CC*,*As*-L^{iPr})]

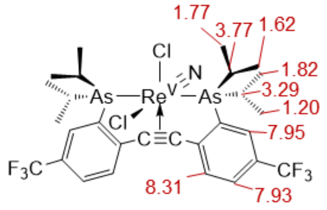

[Re^{V}NCl$_{2}$(κ^{4}-*As*,*CC*,*As*-L^{iPr})]

Figure S19. ^{1}H NMR chemical shift assignments for technetium and rhenium alkyne complexes.

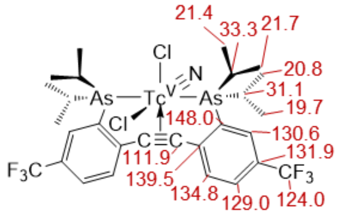

[Tc^{V}NCl$_{2}$(κ^{4}-*As*,*CC*,*As*-L^{iPr})]

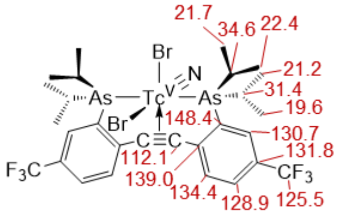

[Tc^{V}NBr$_{2}$(κ^{4}-*As*,*CC*,*As*-L^{iPr})]

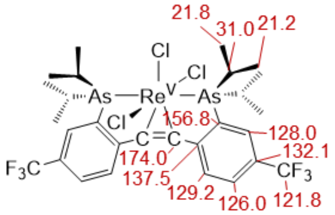

[Re^{V}Cl$_{3}$(κ^{4}-*As*,*CC*,*As*-L^{iPr})]

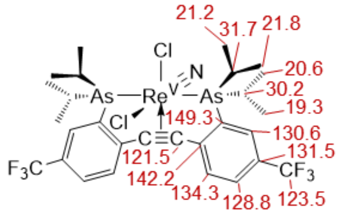

[Re^{V}NCl$_{2}$(κ^{4}-*As*,*CC*,*As*-L^{iPr})]

Figure S20. ^{13}C NMR chemical shift assignments for technetium and rhenium alkyne complexes.

S3.2 *mer*-[Tc^{V}NBr$_2$(κ^4-*As*,*CC*,*As*-L^{iPr})].

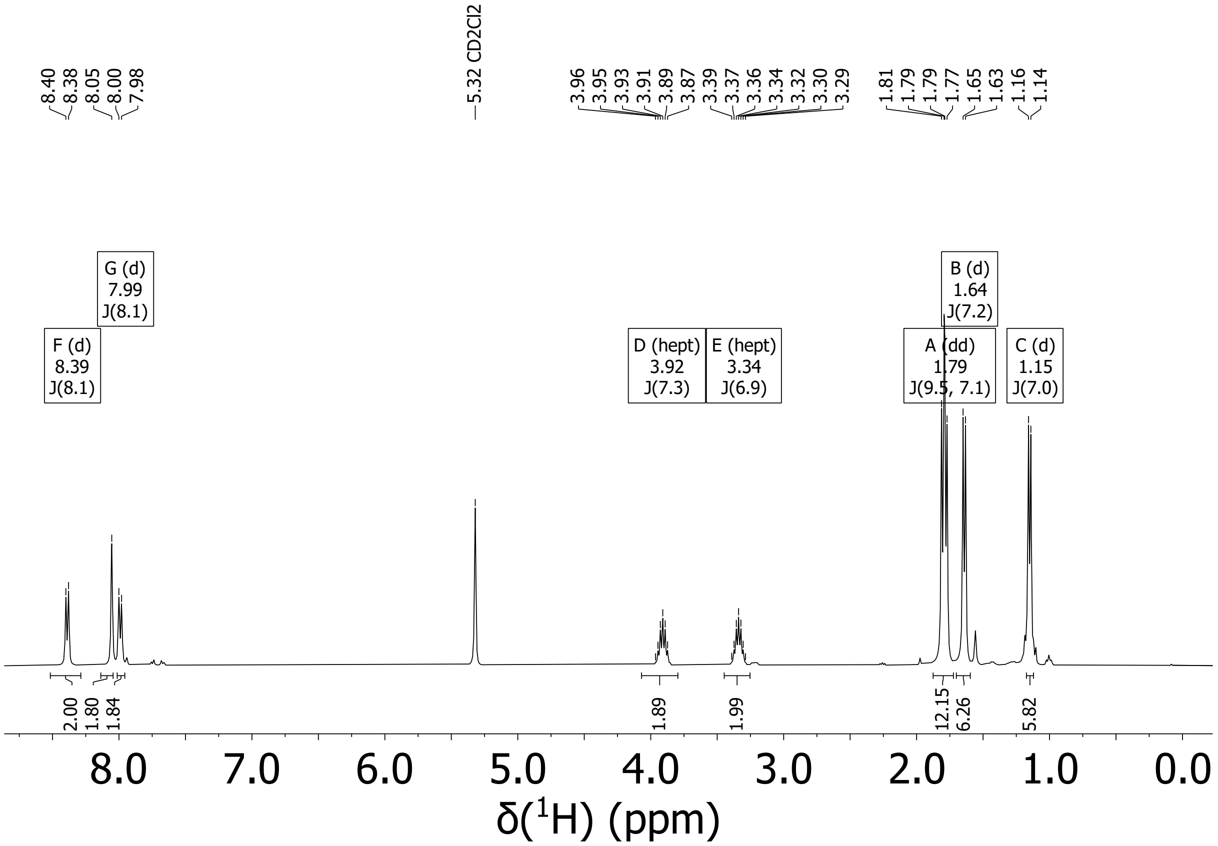

Figure S21. ^{1}H NMR spectrum of *mer*-[Tc^{V}NBr$_2$(κ^4-*As*,*CC*,*As*-L^{iPr})] in CD_2Cl_2.

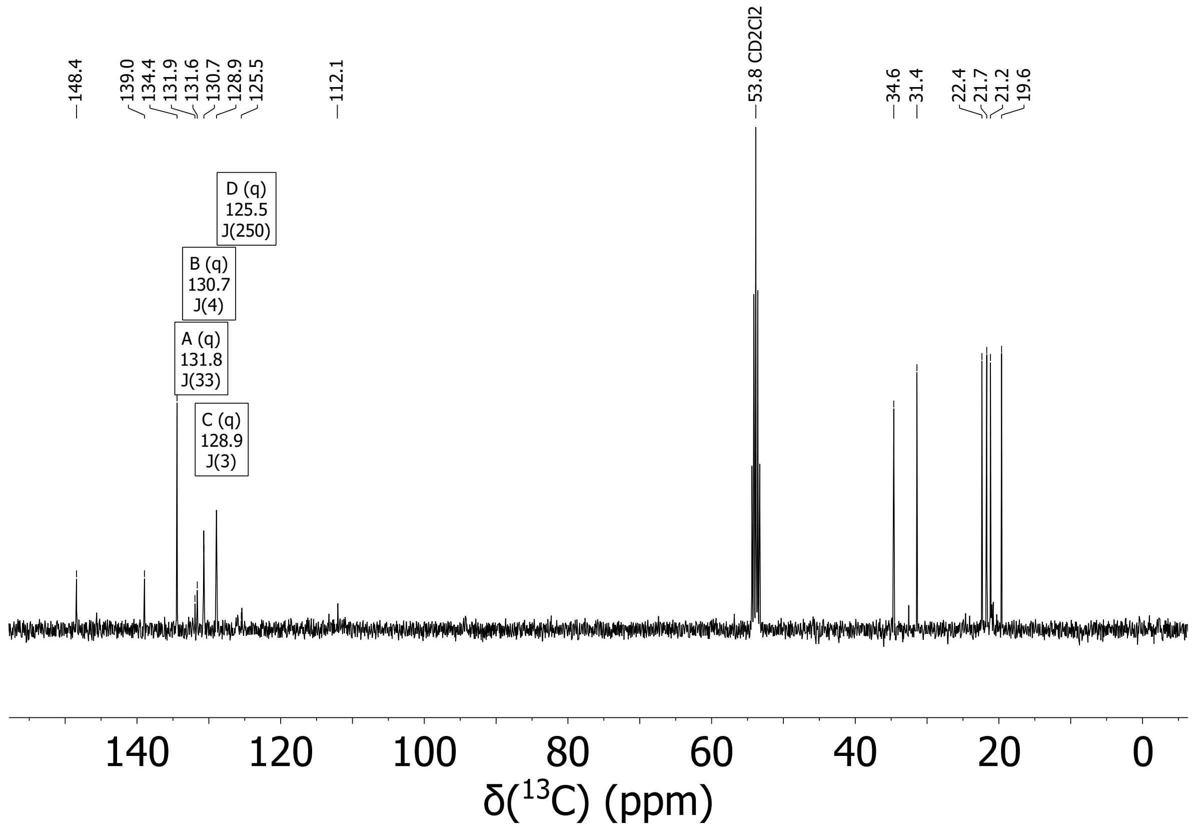

Figure S22. ^{13}C{^{1}H} NMR spectrum of *mer*-[Tc^{V}NBr$_2$(κ^4-*As*,*CC*,*As*-L^{iPr})] in CD_2Cl_2.

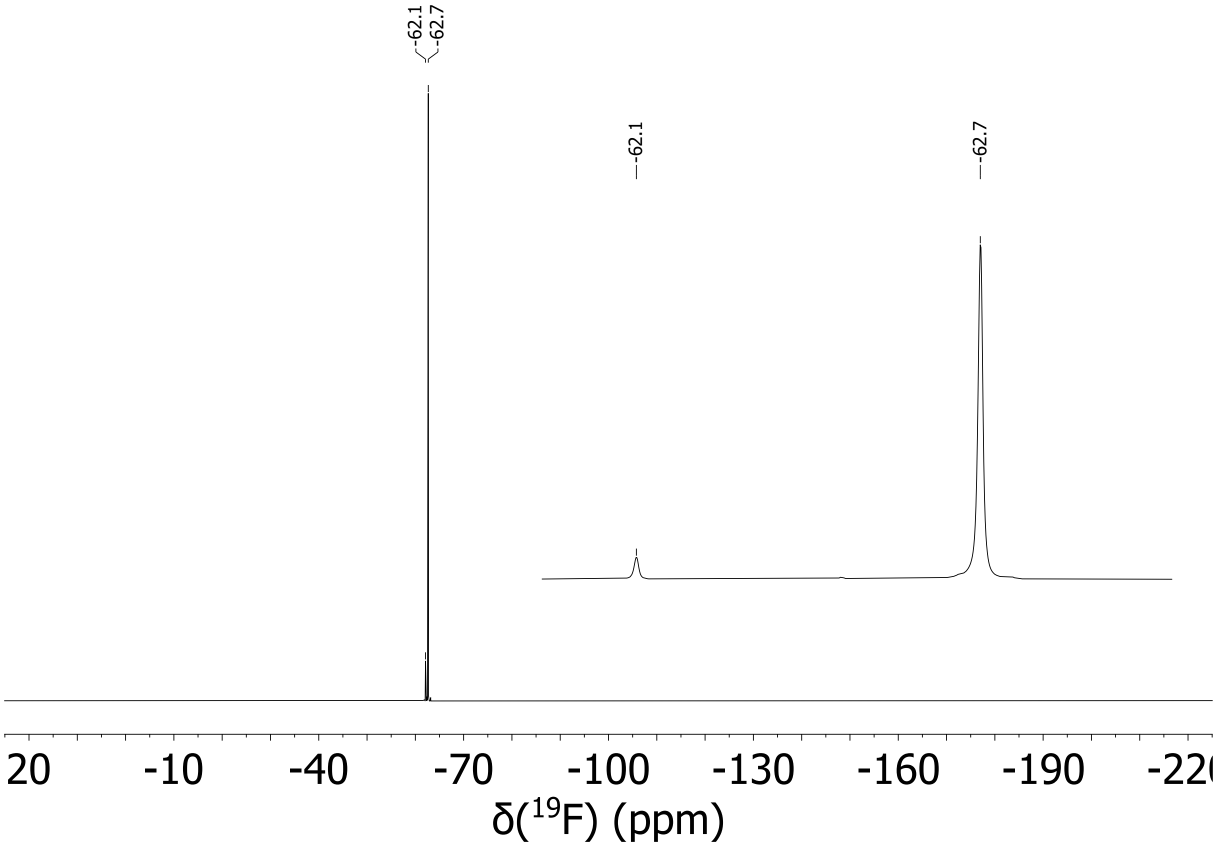

Figure S23. ^{19}F NMR spectrum of *mer*-[$Tc^{V}NBr_2$(κ^4-*As*,*CC*,*As*-L^{iPr})] in CD_2Cl_2. The additional resonance at -62.1 ppm (< 3%) results from a trace of left-over free ligand.

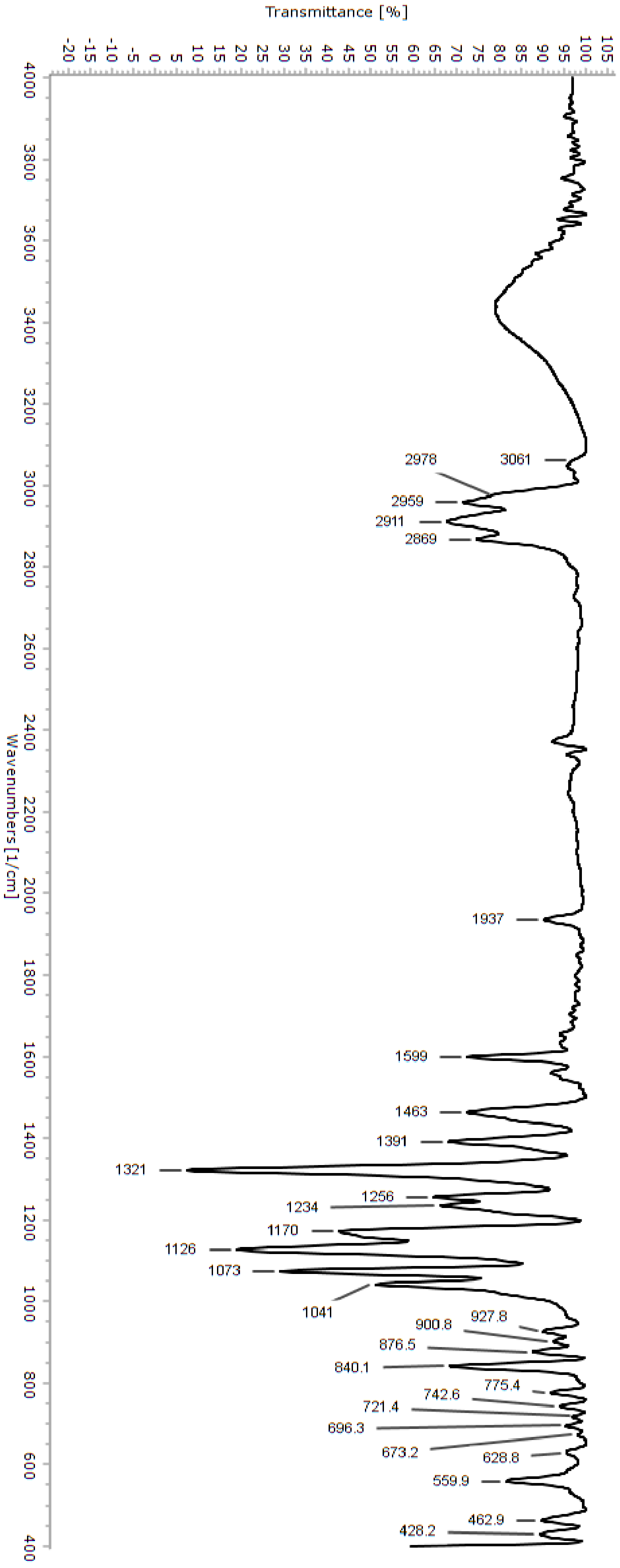

Figure S24. IR spectrum of *mer*-[$Tc^{V}NBr_2$(κ^4-*As*,*CC*,*As*-L^{iPr})].

S3.3 *mer*-[$Tc^{V}NCl_2$(κ^4-*As*,*CC*,*As*-L^{iPr})].

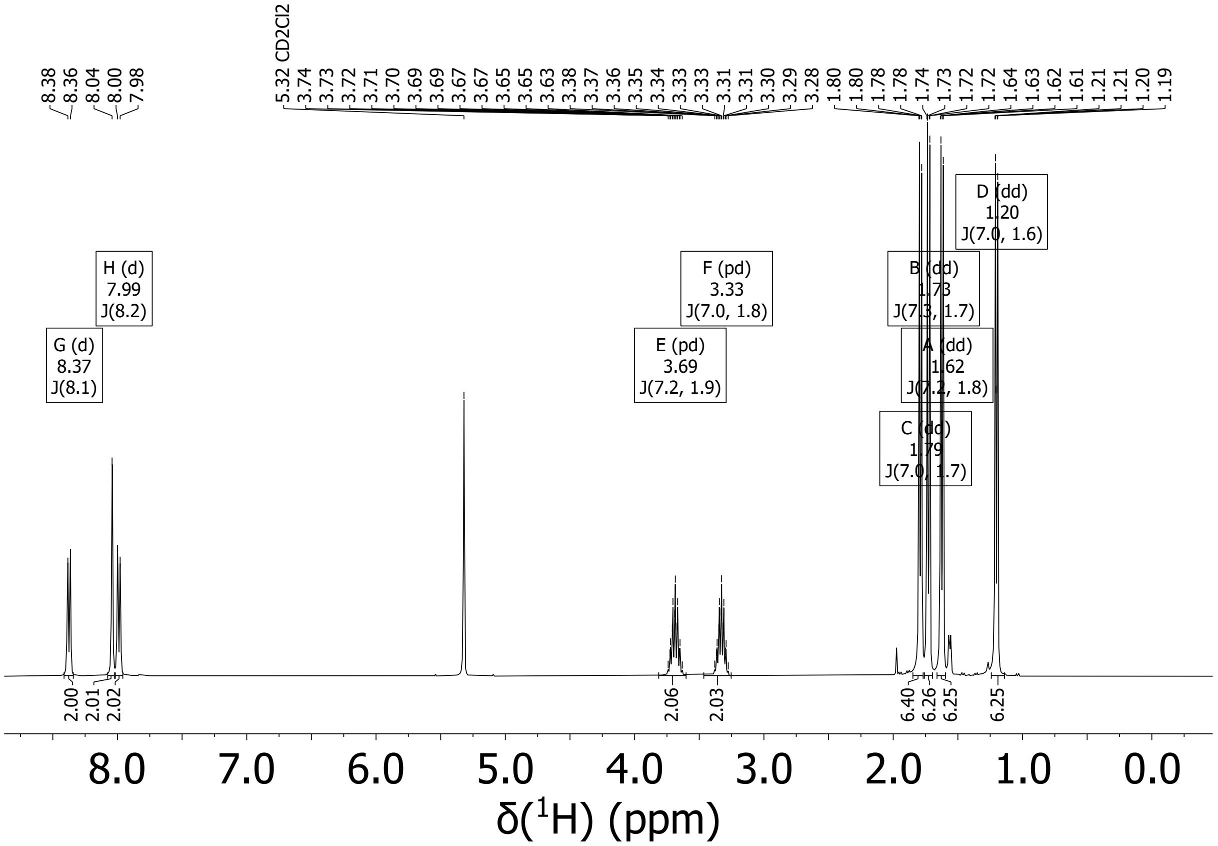

Figure S25. ^{1}H NMR spectrum of *mer*-[$Tc^{V}NCl_2$(κ^4-*As*,*CC*,*As*-L^{iPr})] in CD_2Cl_2.

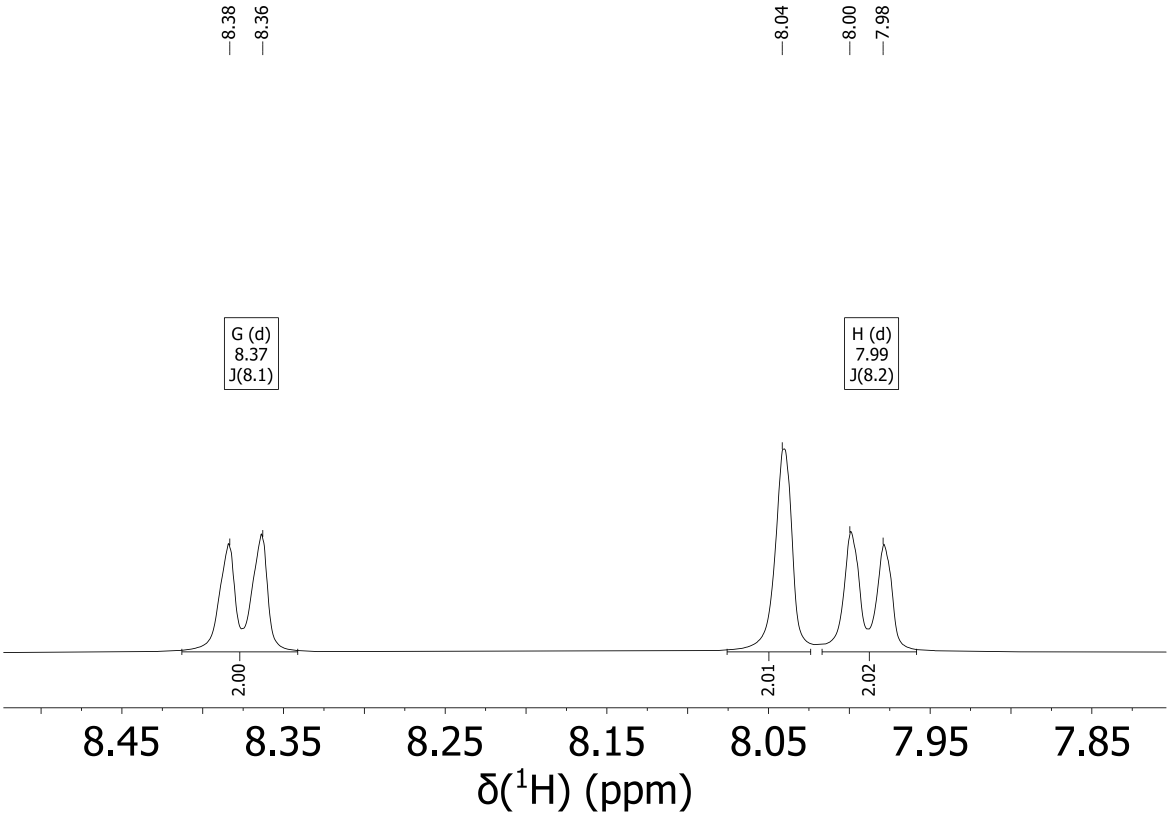

Figure S26. ^{1}H NMR spectrum of *mer*-[$Tc^{V}NCl_2$(κ^4-*As*,*CC*,*As*-L^{iPr})] in CD_2Cl_2. Zoom on aromatic region.

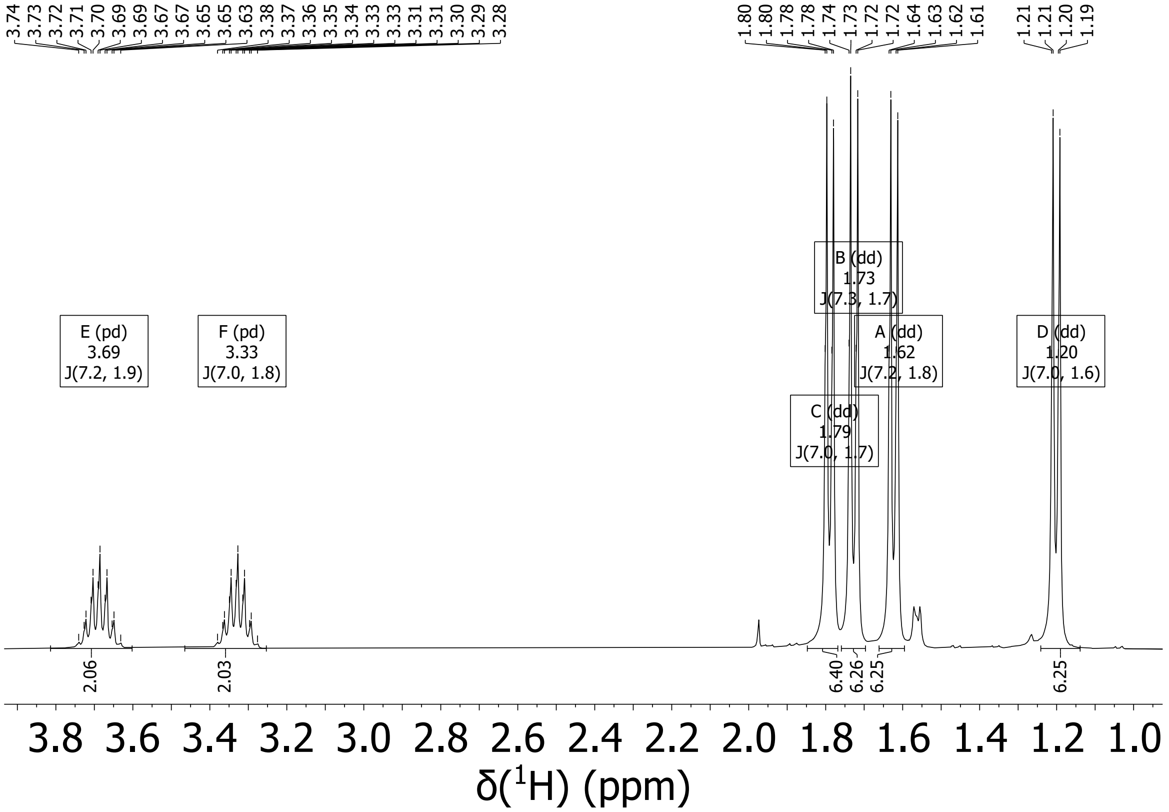

Figure S27. ^{1}H NMR spectrum of *mer*-[Tc^{V}NCl$_2$(κ^4-*As*,*CC*,*As*-L^{iPr})] in CD$_2$Cl$_2$. Zoom on aliphatic region.

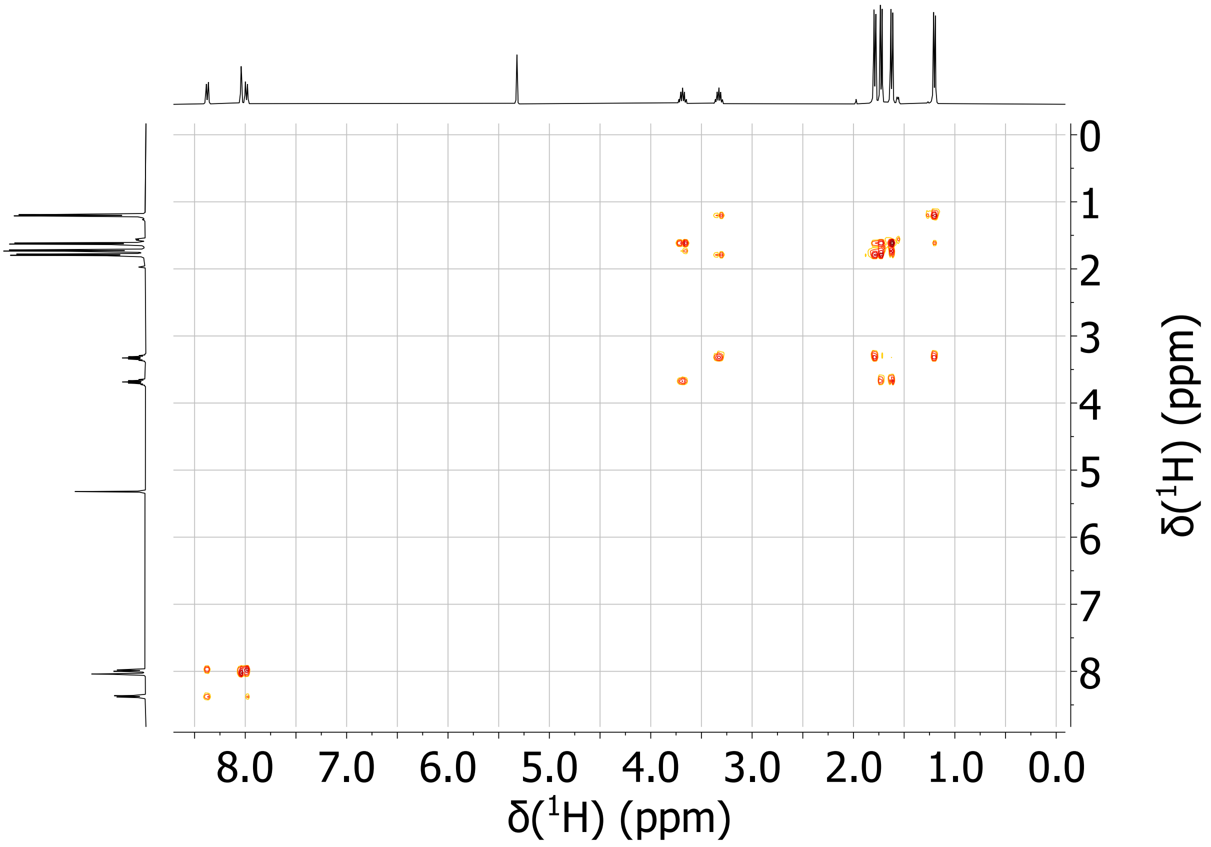

Figure S28. ^{1}H,^{1}H-COSY NMR spectrum of *mer*-[Tc^{V}NCl$_2$(κ^4-*As*,*CC*,*As*-L^{iPr})] in CD$_2$Cl$_2$.

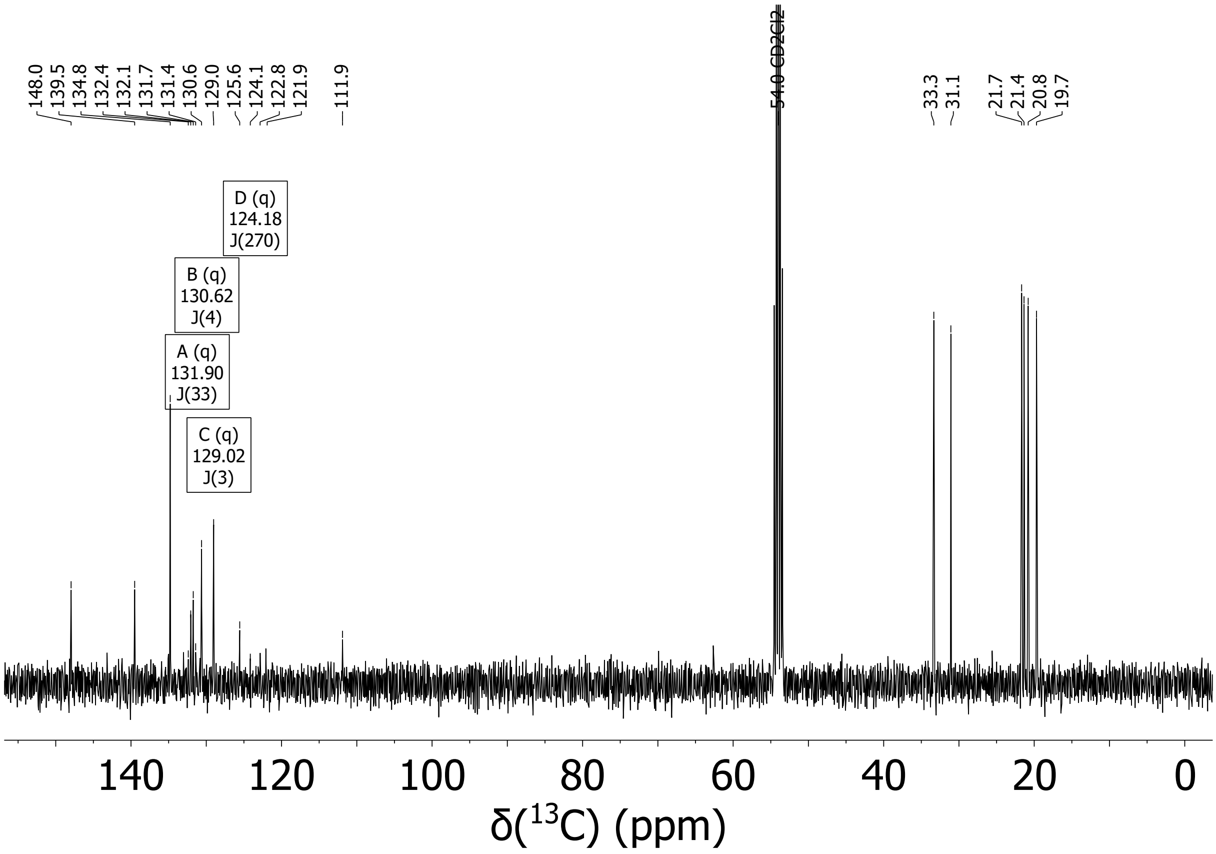

Figure S29. $^{13}C\{^{1}H\}$ NMR spectrum of *mer*-[$Tc^{V}NCl_2$(κ^4-*As*,*CC*,*As*-L^{iPr})] in CD_2Cl_2.

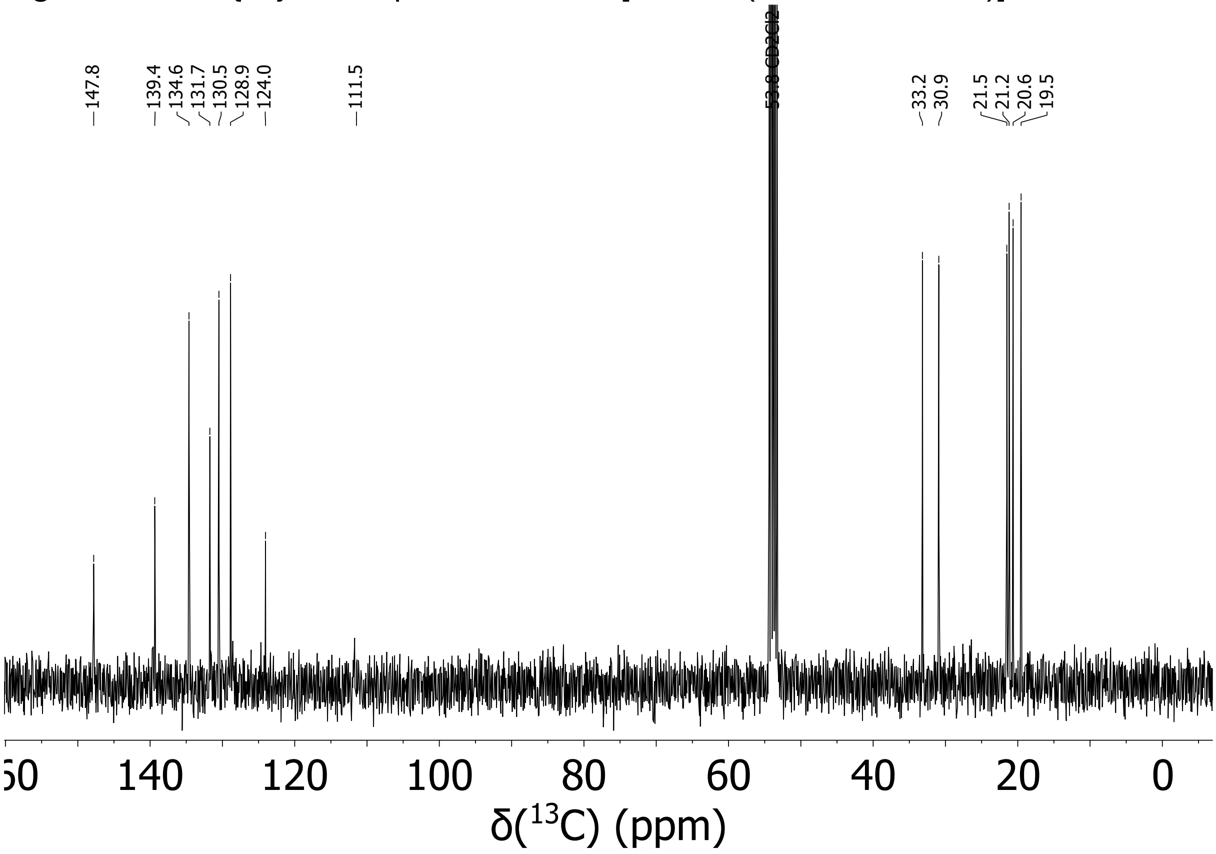

Figure S30. $^{13}C\{^{1}H,^{19}F\}$ broadband decoupled NMR spectrum of *mer*-[$Tc^{V}NCl_2$(κ^4-*As*,*CC*,*As*-L^{iPr})] in CD_2Cl_2.

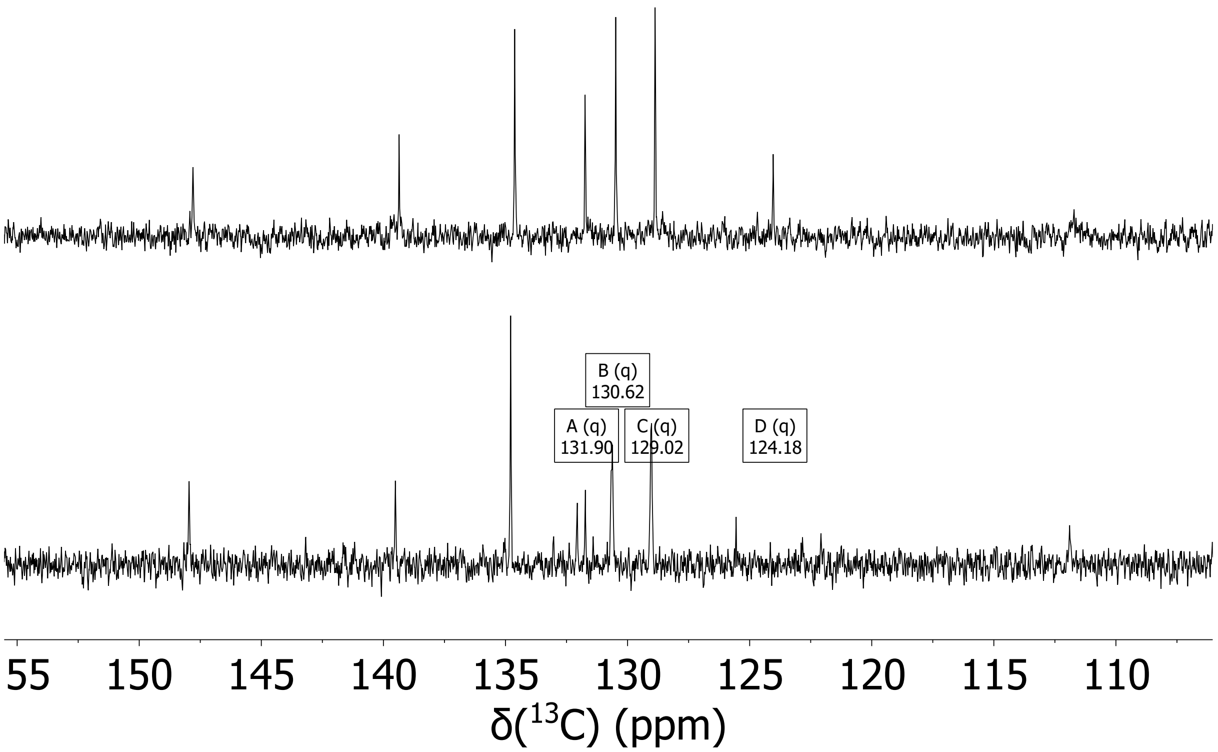

Figure S31. Overlay of $^{13}C\{^{1}H\}$ and $^{13}C\{^{1}H,^{19}F\}$ NMR spectra of *mer*-[$Tc^{V}NCl_2$(κ^4-*As*,*CC*,*As*-L^{iPr})] in CD_2Cl_2.

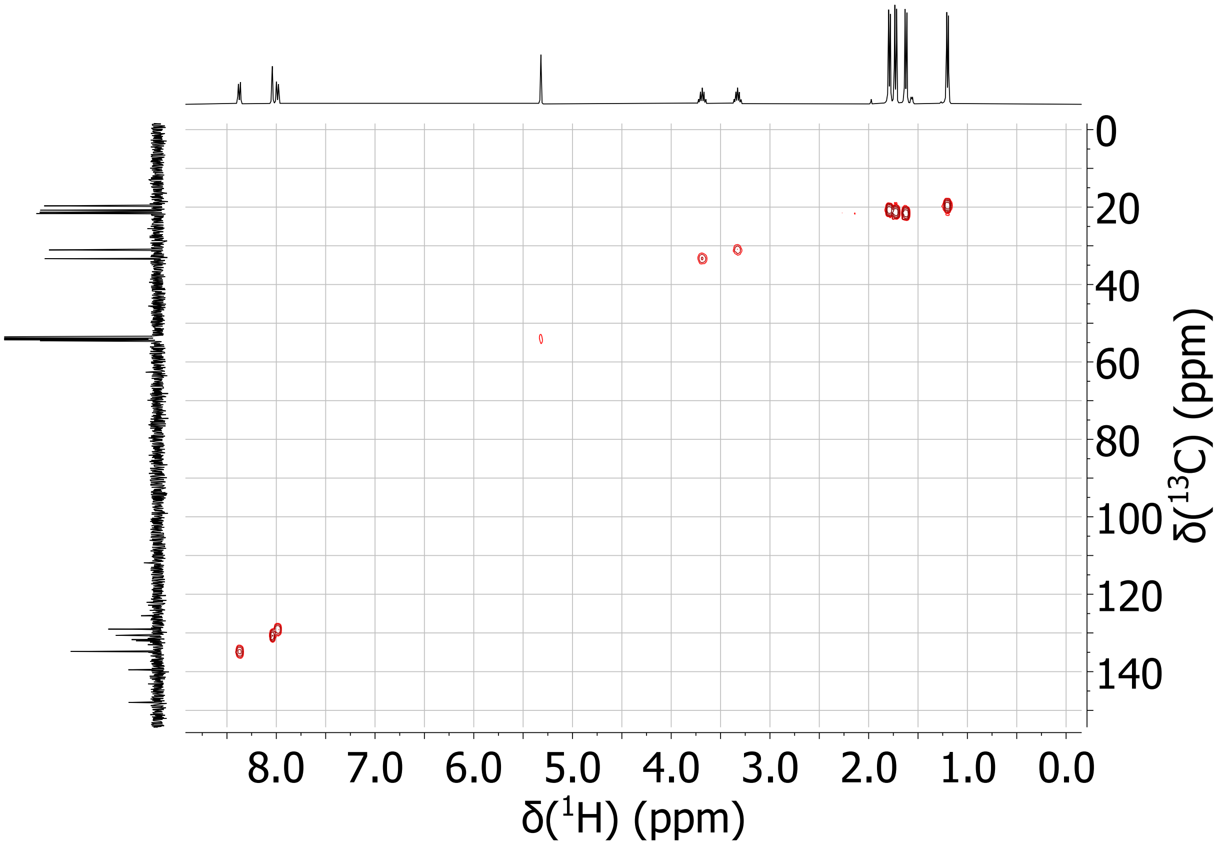

Figure S32. $^{1}H,^{13}C$-HSQC NMR spectrum of *mer*-[$Tc^{V}NCl_2$(κ^4-*As*,*CC*,*As*-L^{iPr})] in CD_2Cl_2.

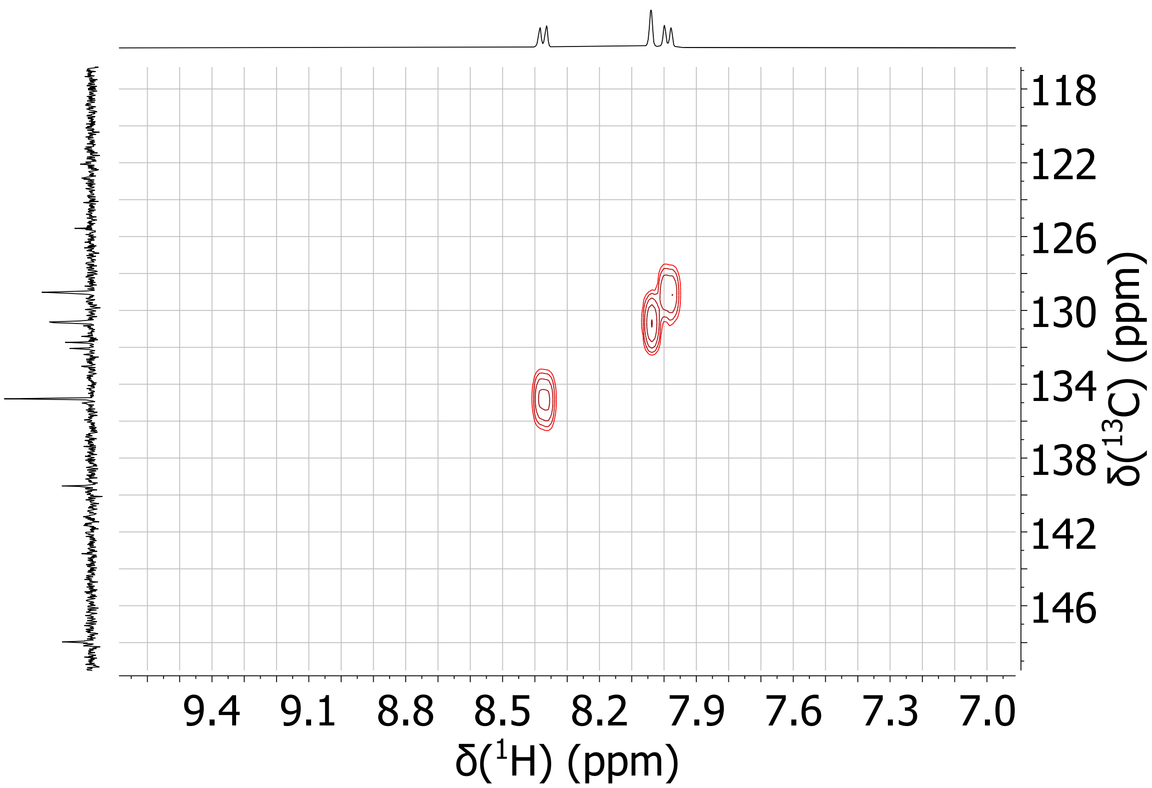

Figure S33. $^{1}H,^{13}C$-HSQC NMR spectrum of *mer*-[$Tc^{V}NCl_2$(κ^4-*As*,*CC*,*As*-L^{iPr})] in CD_2Cl_2. Zoom on the aromatic region.

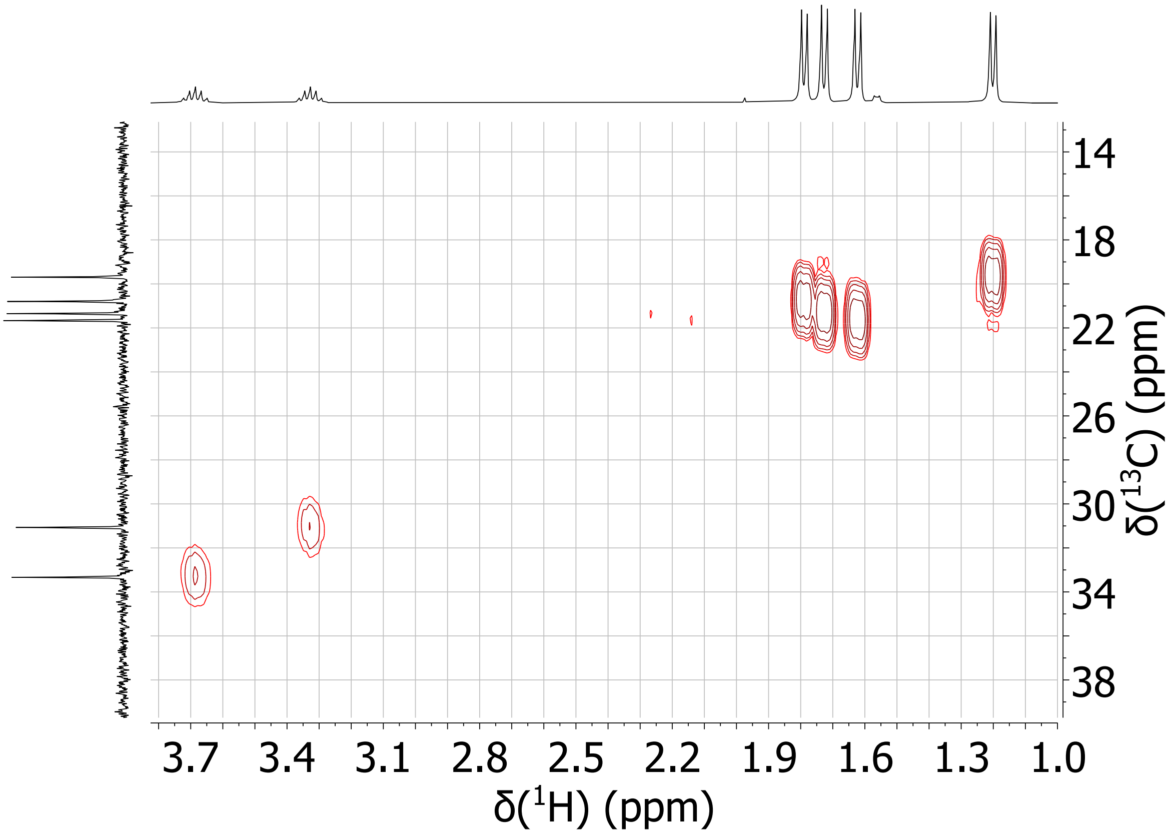

Figure S34. $^{1}H,^{13}C$-HSQC NMR spectrum of *mer*-[$Tc^{V}NCl_2$(κ^4-*As*,*CC*,*As*-L^{iPr})] in CD_2Cl_2. Zoom on the aliphatic region.

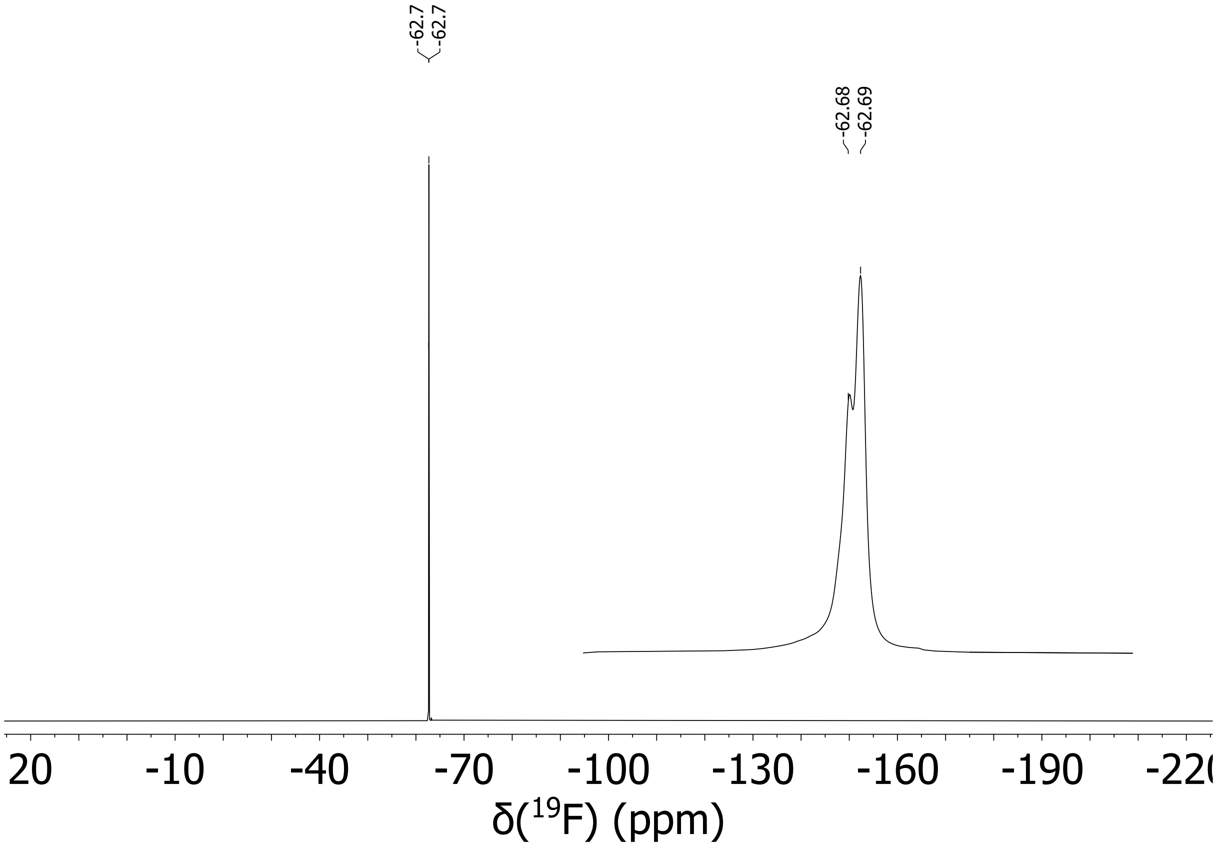

Figure S35. ^{19}F NMR spectrum of *mer*-[$Tc^{V}NCl_2$(κ^4-*As*,*CC*,*As*-L^{iPr})] in CD_2Cl_2.

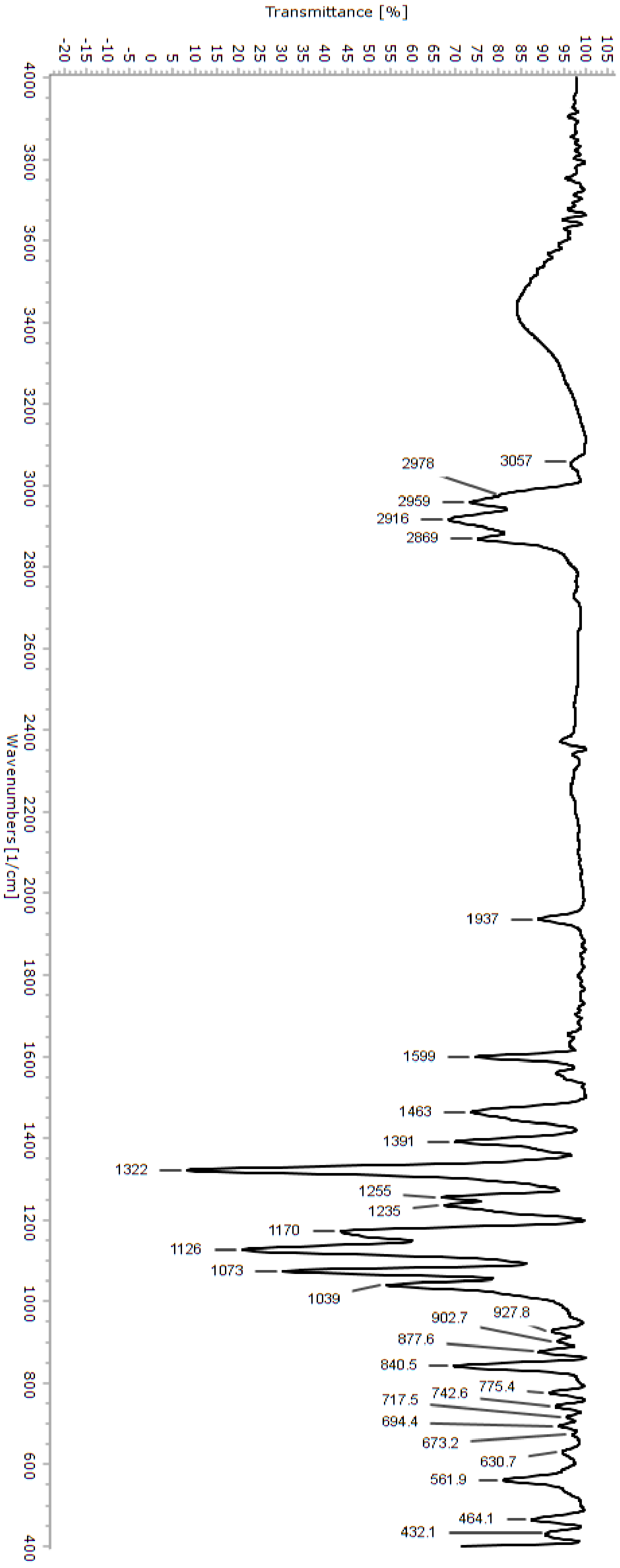

Figure S36. IR spectrum of *mer*-[$Tc^{V}NCl_2$(κ^4-*As*,*CC*,*As*-L^{iPr})].

S3.4 *cis,trans,mer*-[Tc^{V}N(CN)Cl(κ^4-*As*,*CC*,*As*-L^{iPr})].

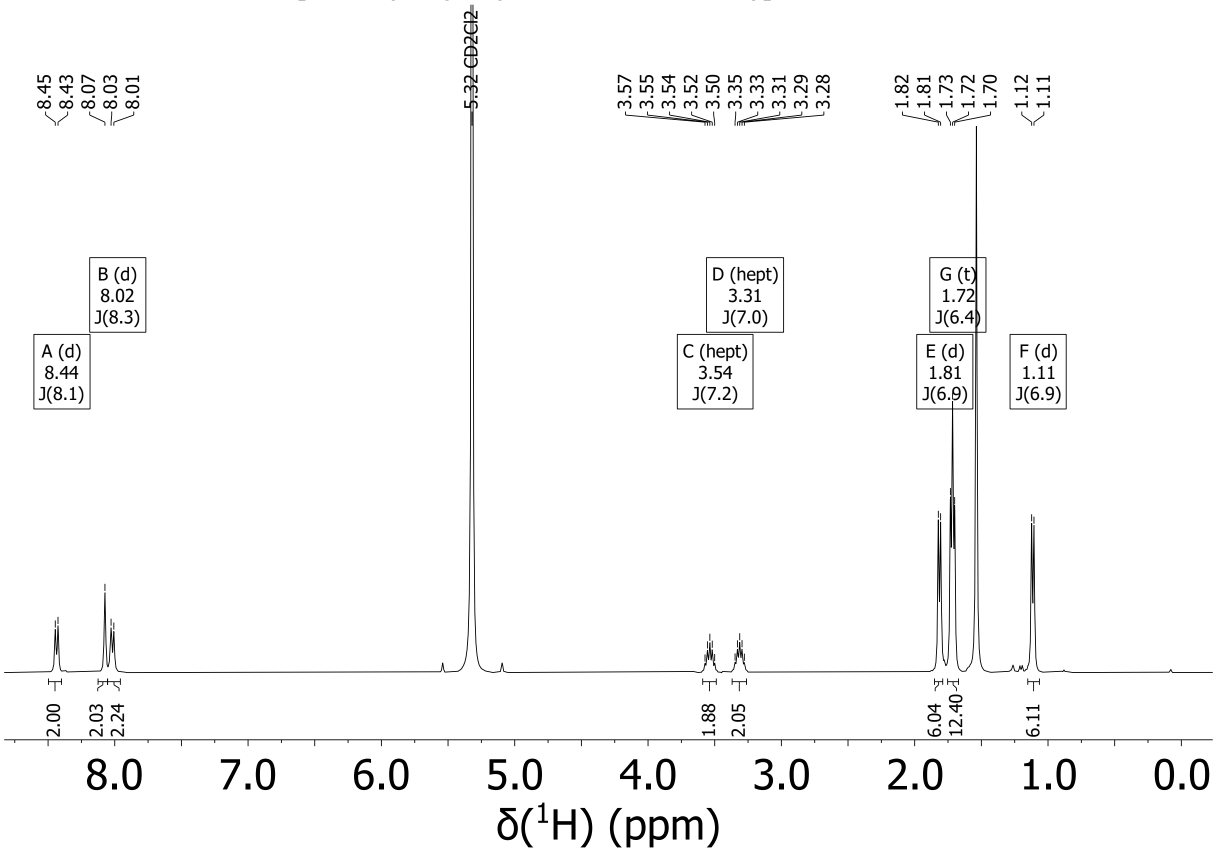

Figure S37. ^{1}H NMR spectrum of *mer*-[Tc^{V}N(CN)Cl(κ^4-*As*,*CC*,*As*-L^{iPr})] in CD_2Cl_2. Identified relevant impurities: 3.4 ppm: MeOH, 2.5 ppm: DMSO, 1.5 ppm: H_2O.

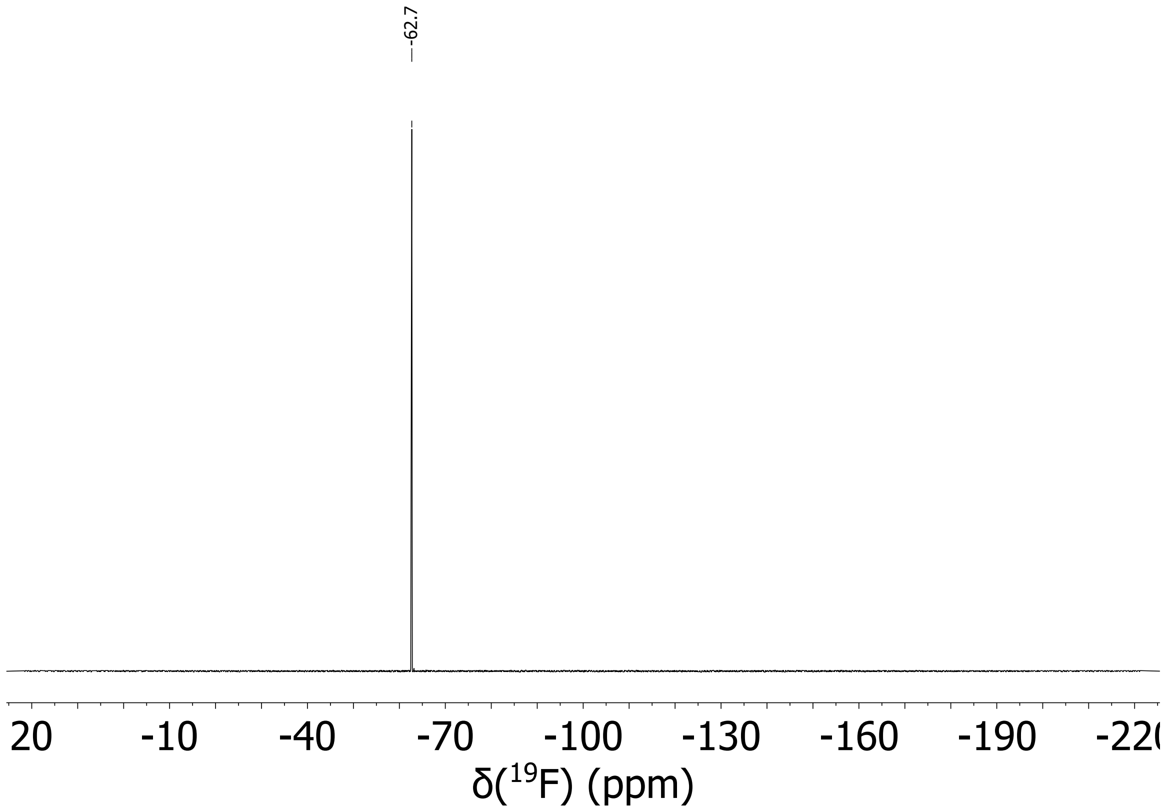

Figure S38. ^{19}F NMR spectrum of *mer*-[Tc^{V}N(CN)Cl(κ^4-*As*,*CC*,*As*-L^{iPr})] in CD_2Cl_2.

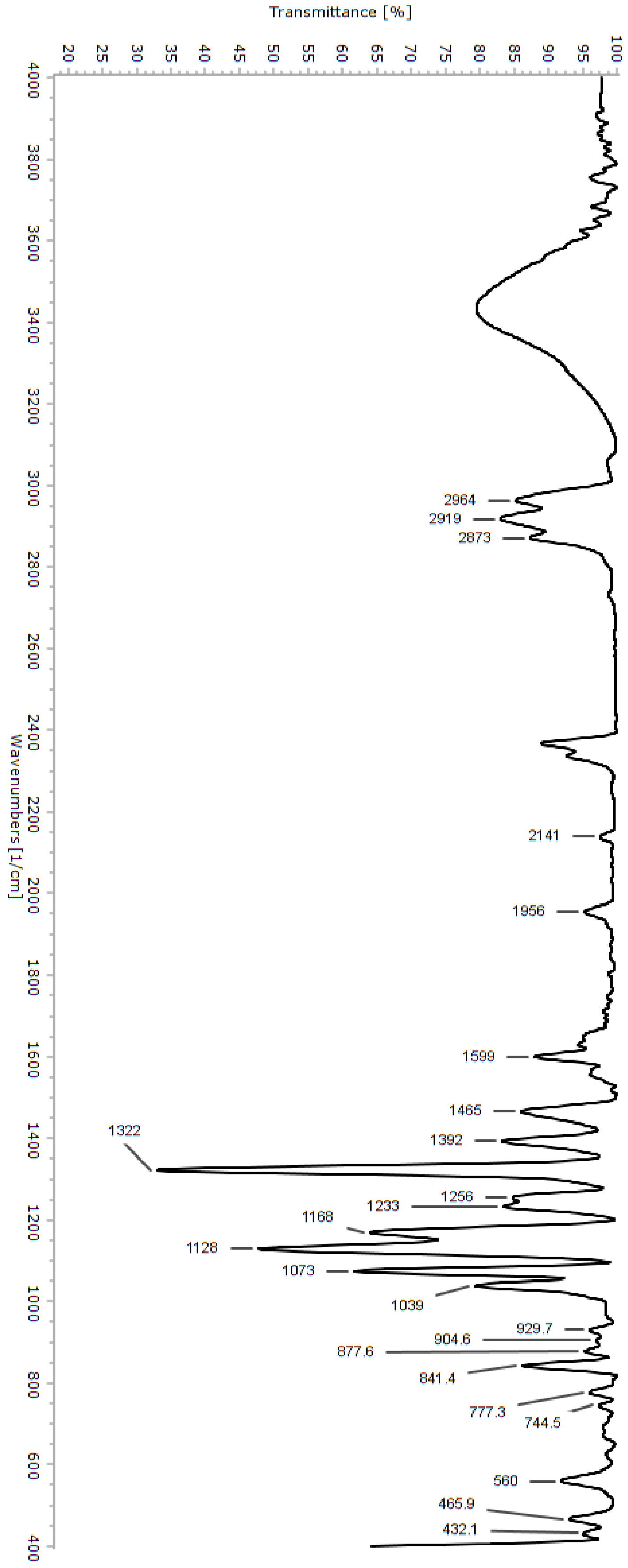

Figure S39. IR spectrum of *mer*-[Tc^{V}N(CN)Cl(κ^{4}-*As*,*CC*,*As*-L^{iPr})].

S3.5 *mer*-[Tc^{III}Cl$_3$(κ^4-*As*,*CC*,*As*-L^{iPr})].

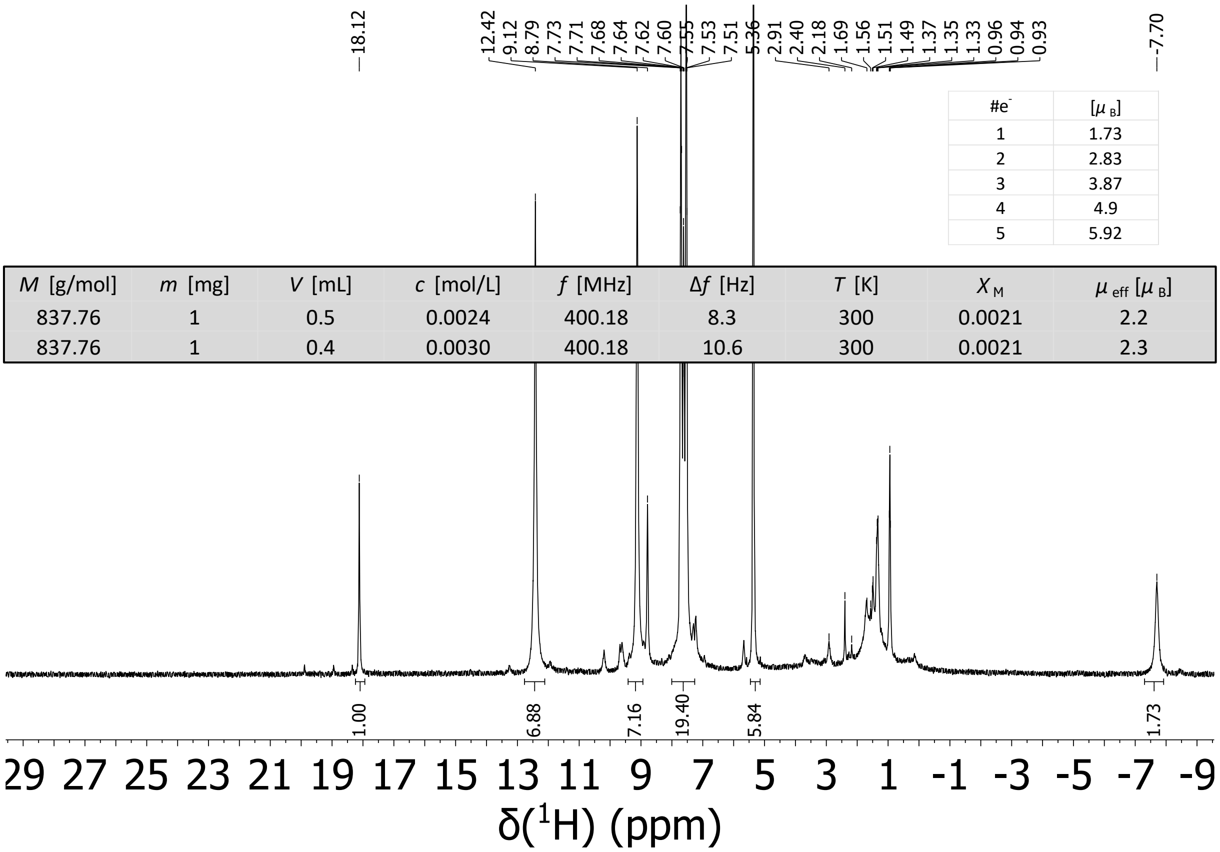

#e$^-$	[μ_B]
1	1.73
2	2.83
3	3.87
4	4.9
5	5.92

M [g/mol]	*m* [mg]	*V* [mL]	*c* [mol/L]	*f* [MHz]	Δ*f* [Hz]	*T* [K]	X_M	μ_{eff} [μ_B]
837.76	1	0.5	0.0024	400.18	8.3	300	0.0021	2.2
837.76	1	0.4	0.0030	400.18	10.6	300	0.0021	2.3

Figure S40. ^{1}H NMR spectrum and Evans NMR data of paramagnetic *mer*-[Tc^{III}Cl$_3$(κ^4-*As*,*CC*,*As*-L^{iPr})] in CD_2Cl_2.

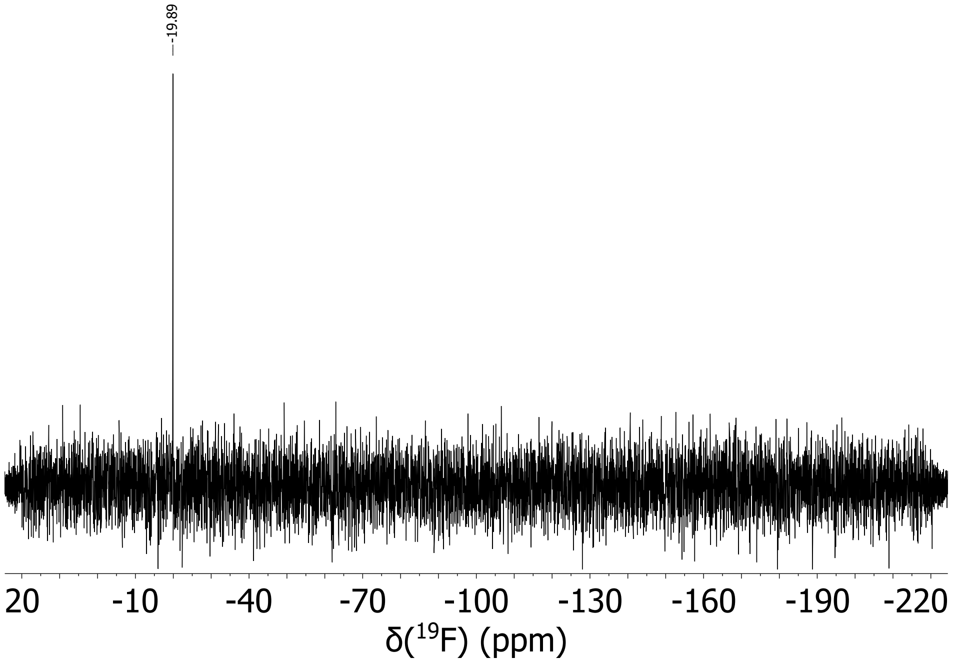

Figure S41. ^{19}F{^{1}H} NMR spectrum of paramagnetic *mer*-[Tc^{III}Cl$_3$(κ^4-*As*,*CC*,*As*-L^{iPr})] in CD_2Cl_2.

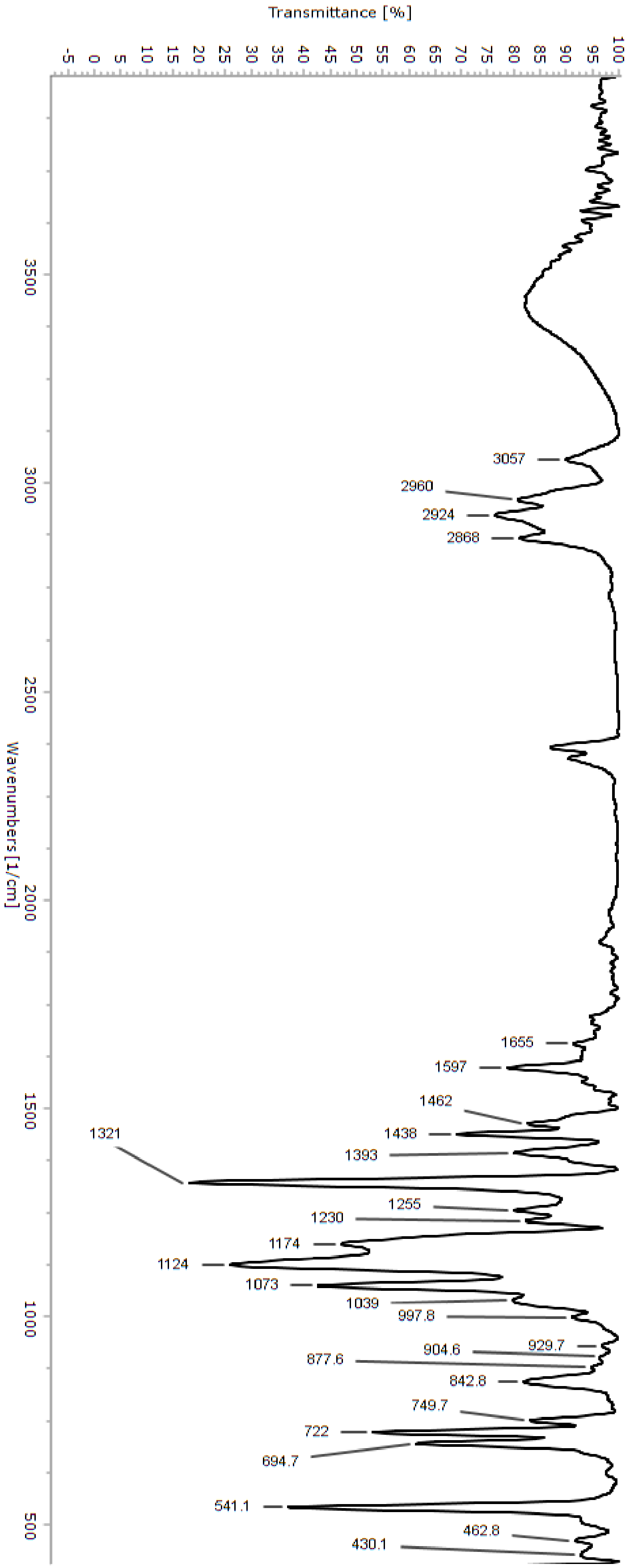

Figure S42. IR spectrum of *mer*-[$Tc^{III}Cl_3$(κ^4-*As*,*CC*,*As*-L^{iPr})].

S3.6 *mer*-[$Re^{V}NCl_2$(κ^4-*As*,*CC*,*As*-L^{iPr})].

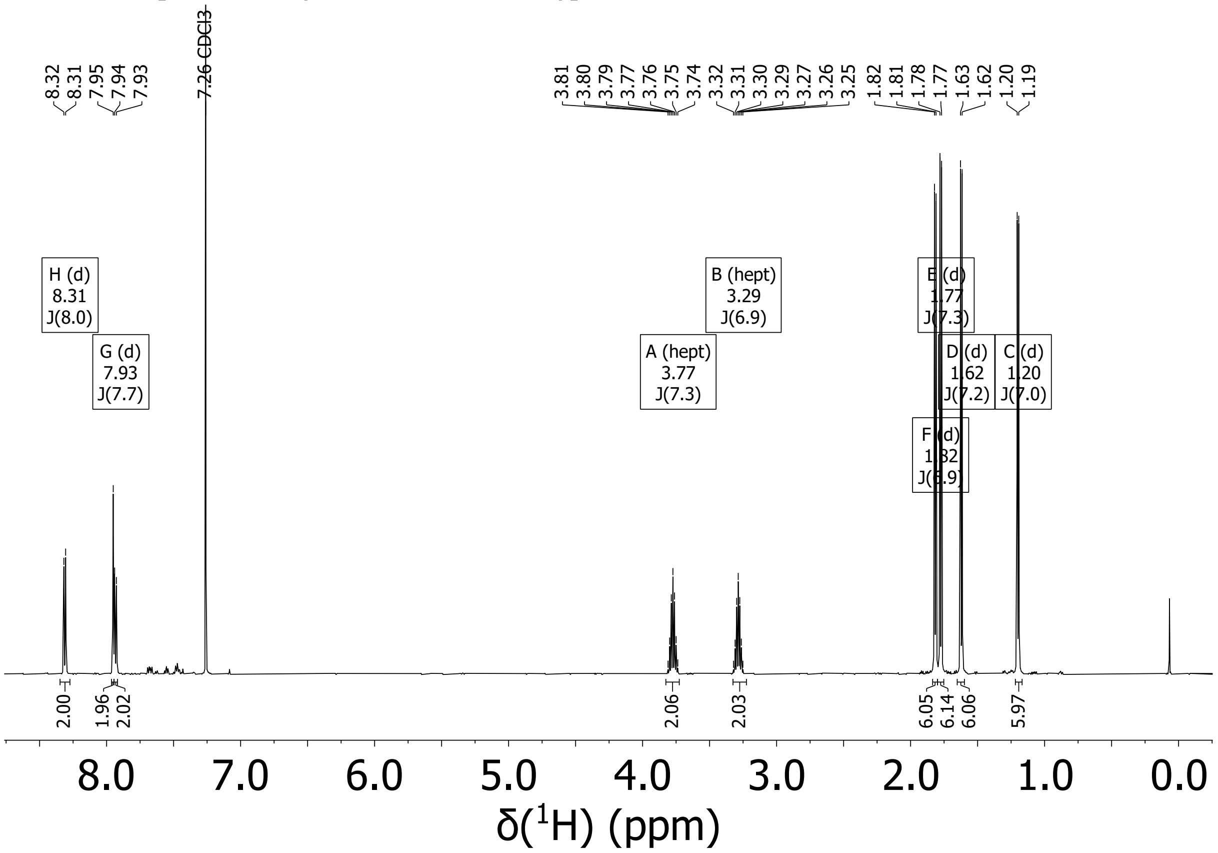

Figure S43. ^1H NMR spectrum of *mer*-[$Re^{V}NCl_2$(κ^4-*As*,*CC*,*As*-L^{iPr})] in $CDCl_3$.

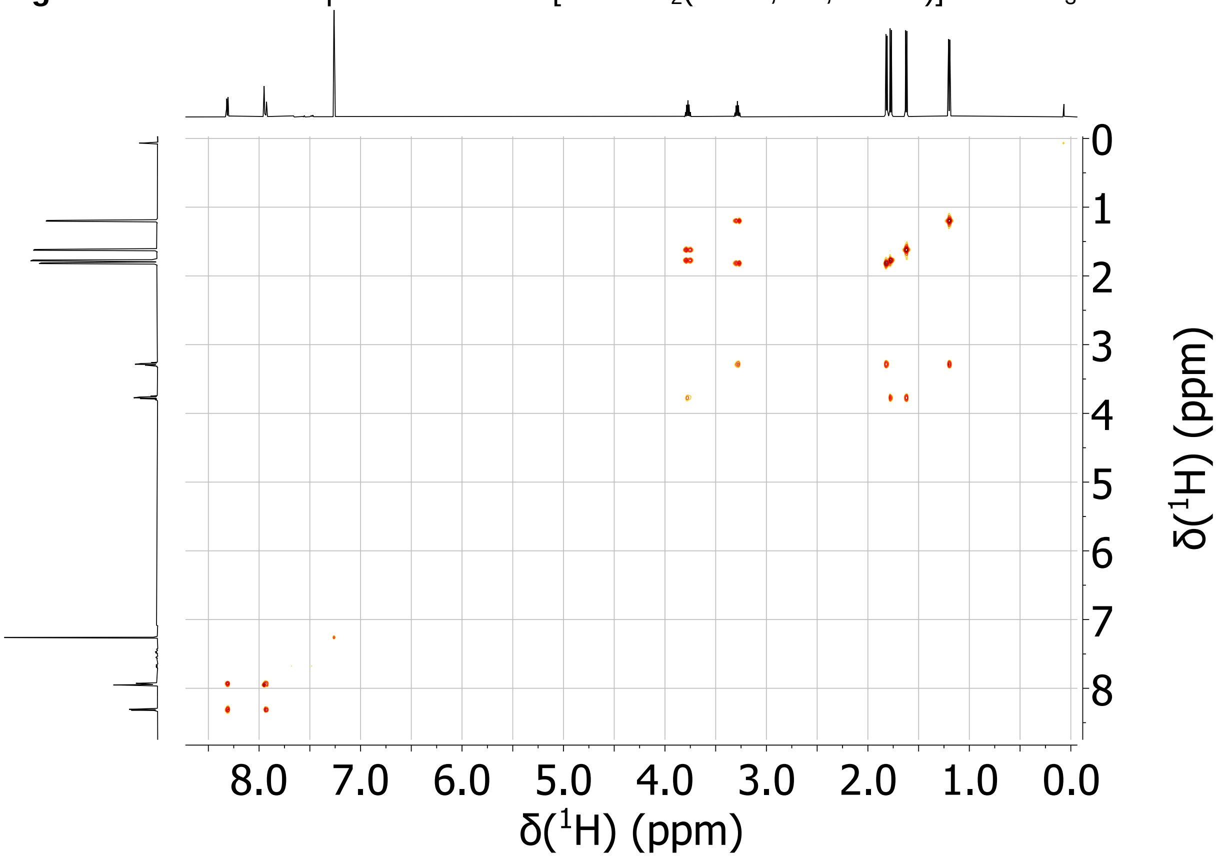

Figure S44. ^1H,^1H-COSY NMR spectrum of *mer*-[$Re^{V}NCl_2$(κ^4-*As*,*CC*,*As*-L^{iPr})] in $CDCl_3$.

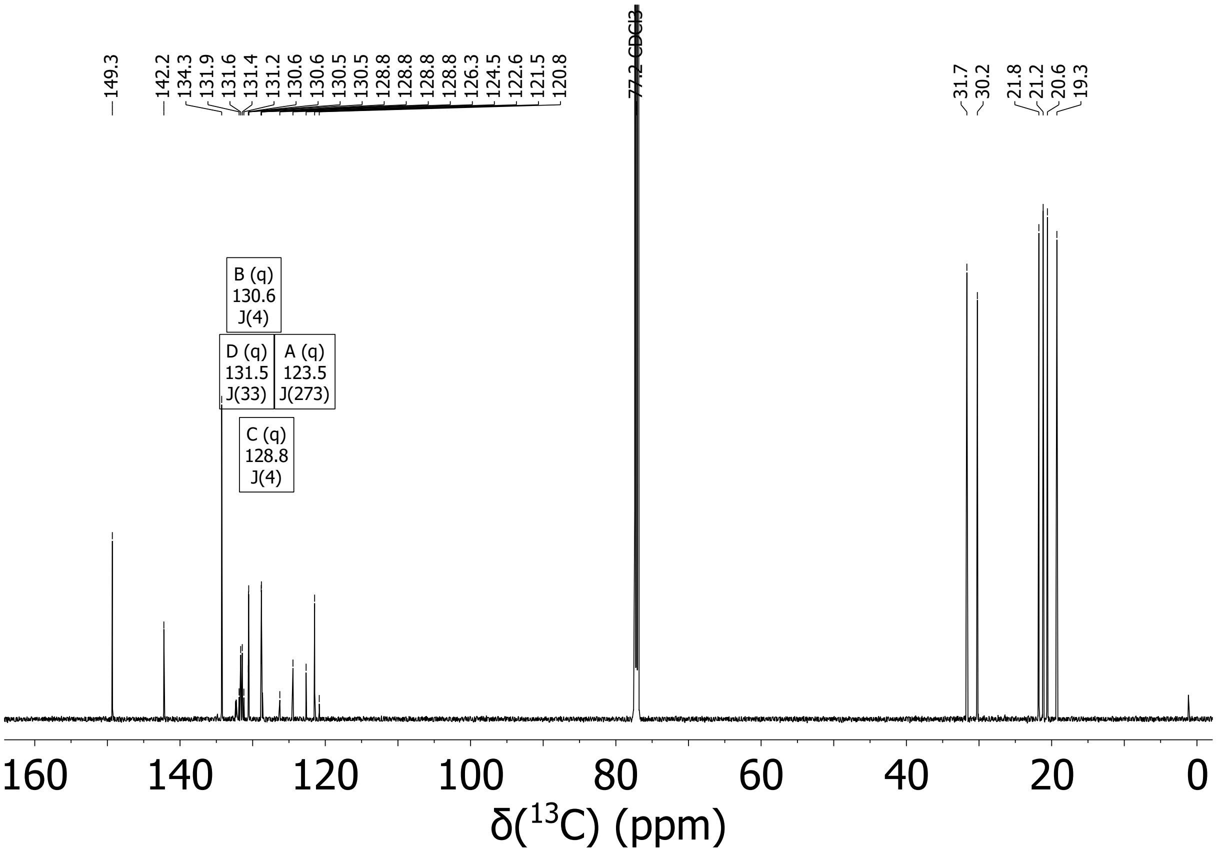

Figure S45. $^{13}C\{^1H\}$ NMR spectrum of *mer*-[Re^VNCl_2(κ^4-*As*,*CC*,*As*-L^{iPr})] in $CDCl_3$.

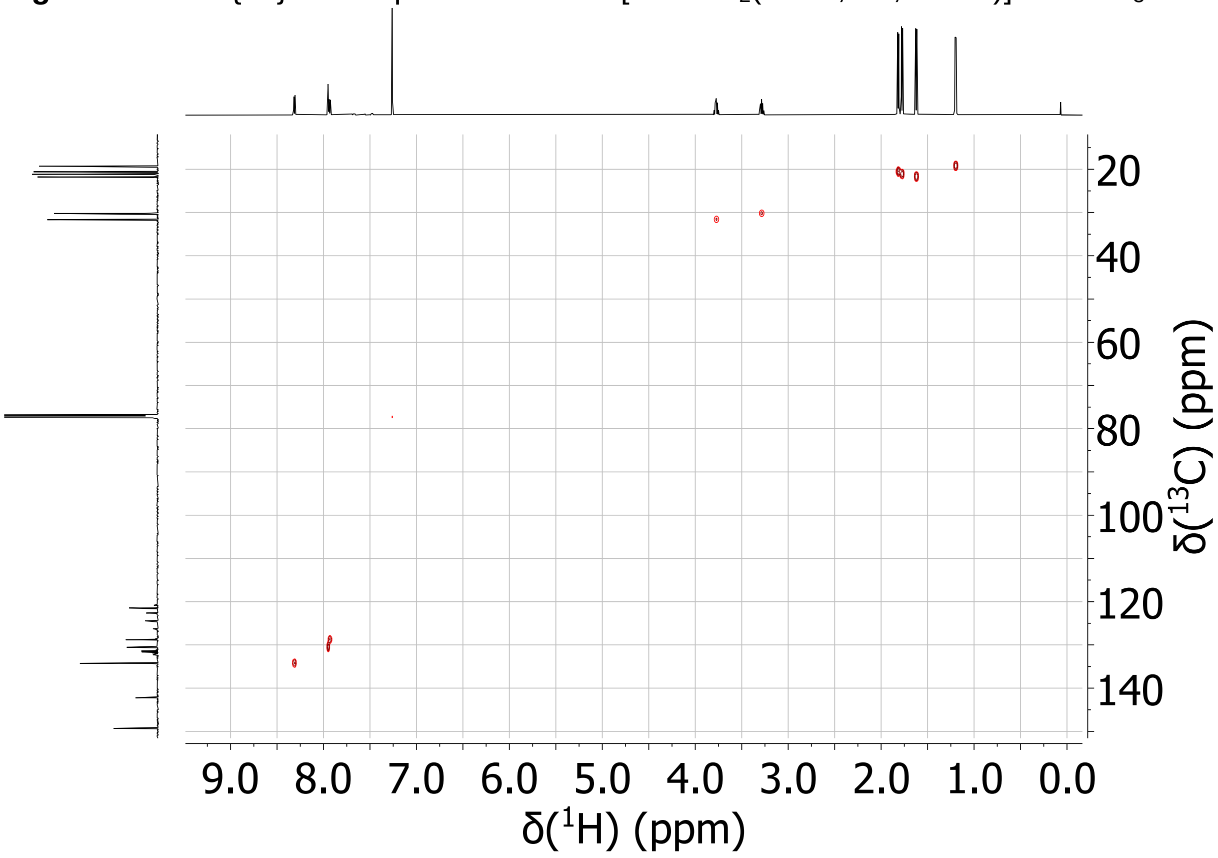

Figure S46. $^1H,^{13}C$-HSQC NMR spectrum of *mer*-[Re^VNCl_2(κ^4-*As*,*CC*,*As*-L^{iPr})] in $CDCl_3$.

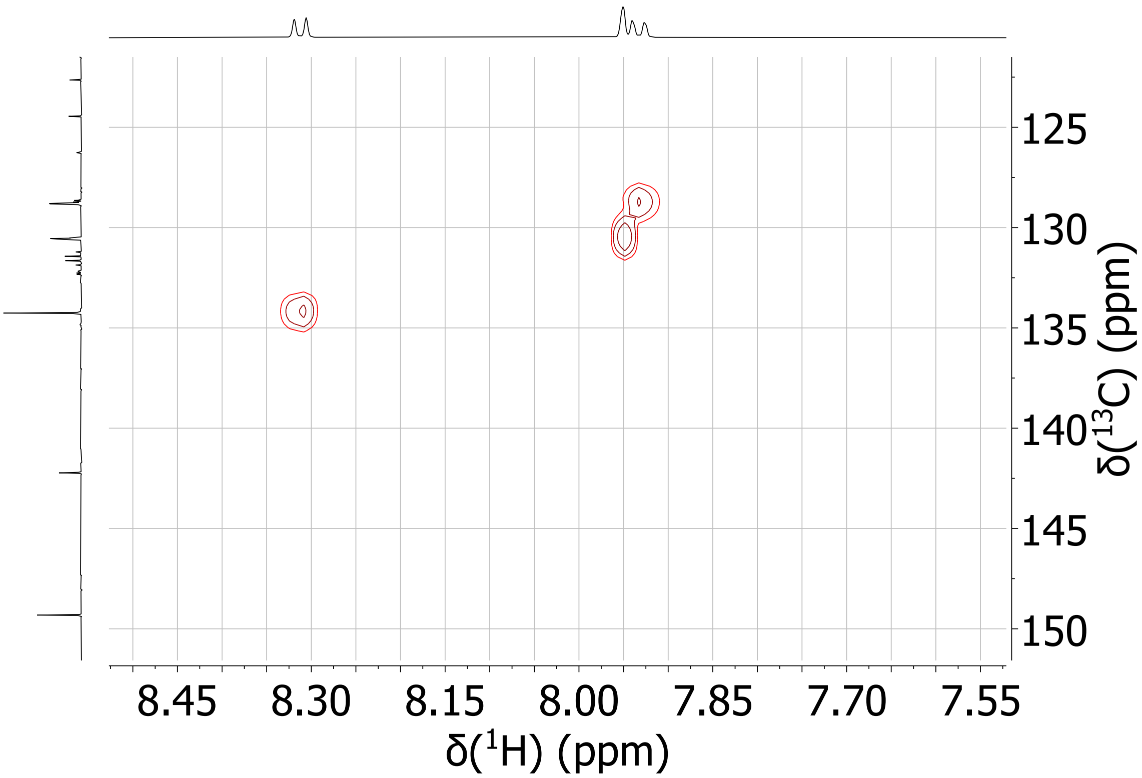

Figure S47. $^{1}H,^{13}C$-HSQC NMR spectrum of *mer*-[Re^{V}NCl$_2$(κ^4-*As*,*CC*,*As*-L^{iPr})] in CDCl$_3$. Zoom on the aromatic region.

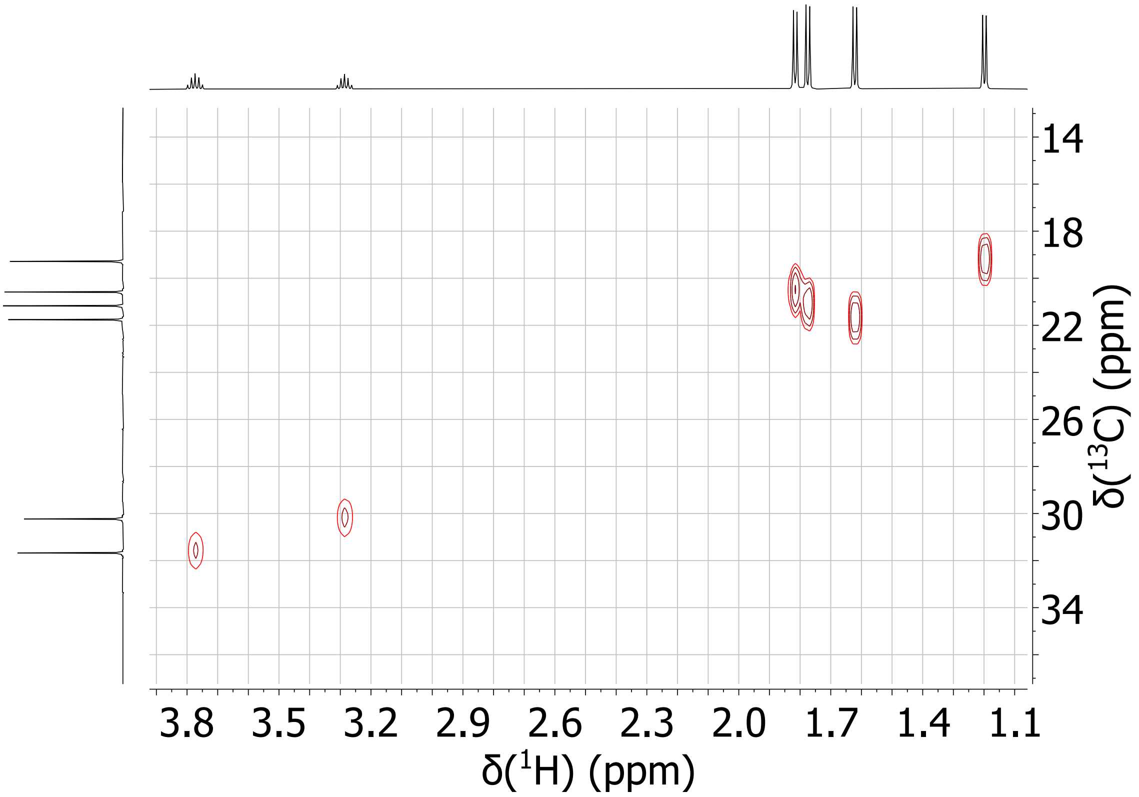

Figure S48. $^{1}H,^{13}C$-HSQC NMR spectrum of *mer*-[Re^{V}NCl$_2$(κ^4-*As*,*CC*,*As*-L^{iPr})] in CDCl$_3$. Zoom on the aliphatic region.

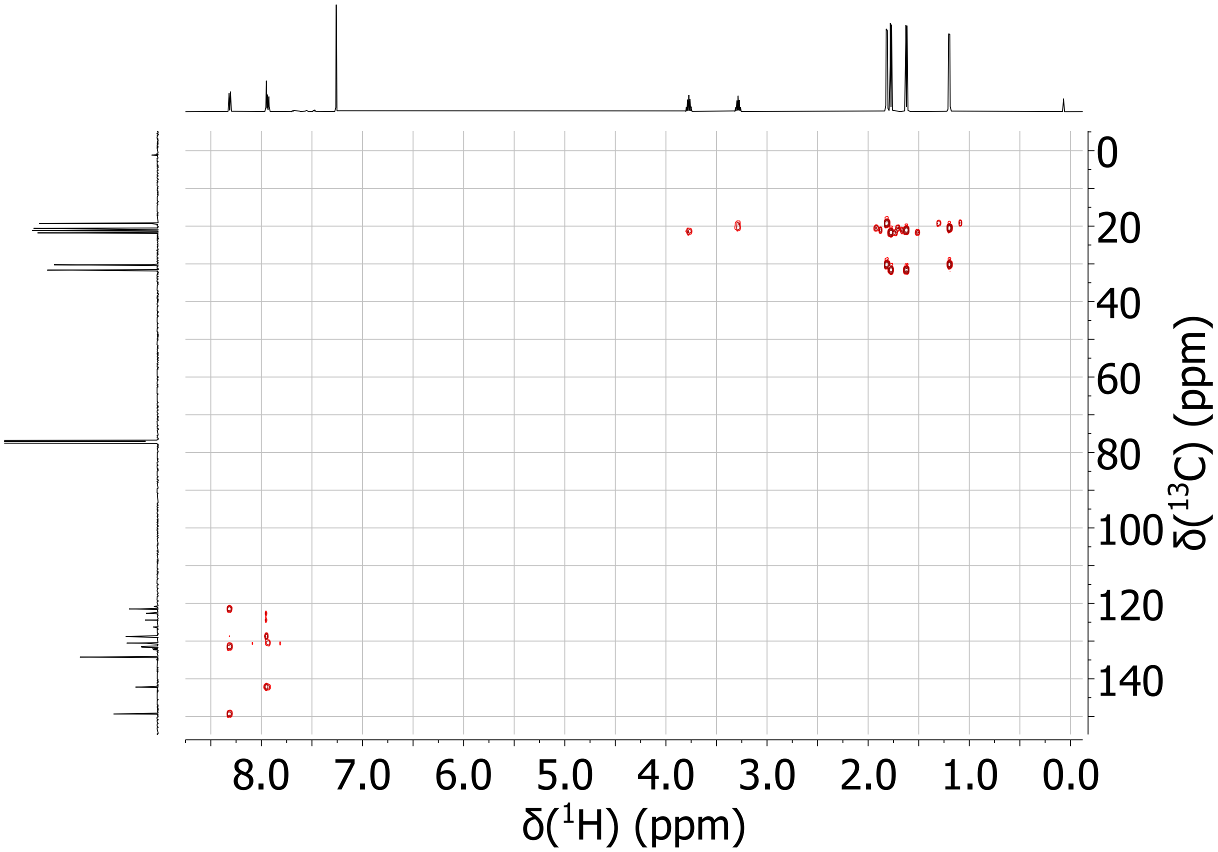

Figure S49. $^{1}H,^{13}C$-HMBC NMR spectrum of *mer*-[$Re^{V}NCl_2$(κ^4-*As*,*CC*,*As*-L^{iPr})] in $CDCl_3$.

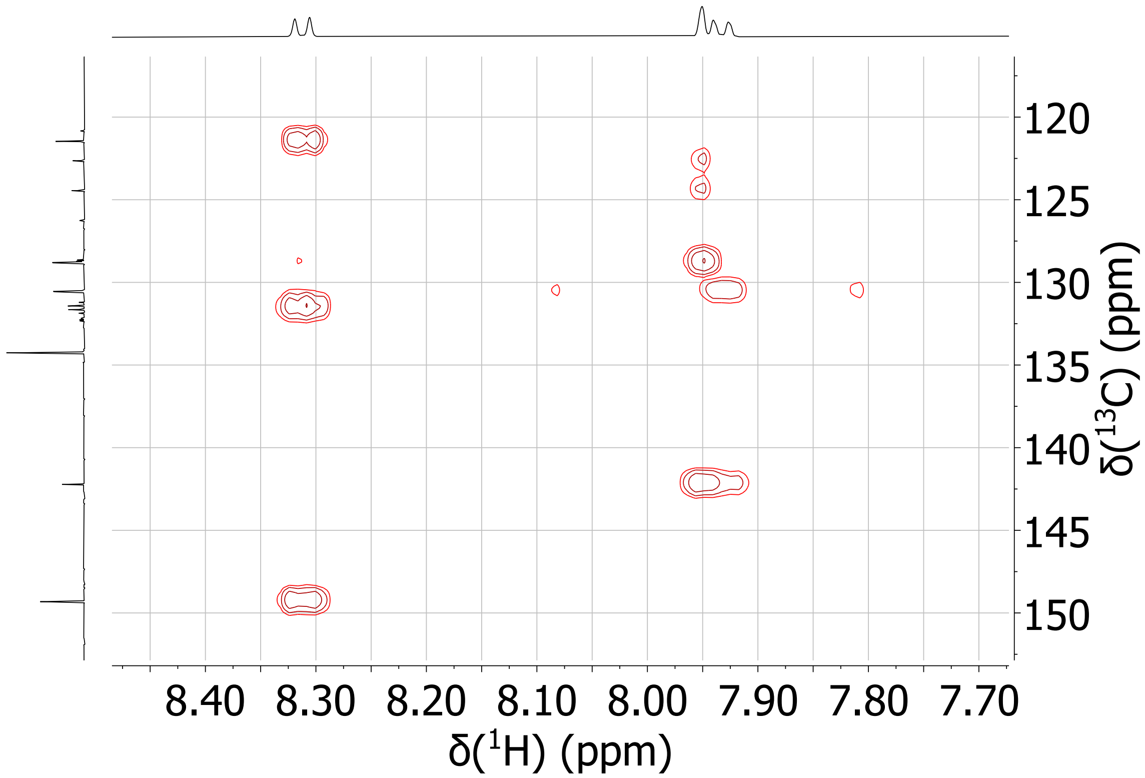

Figure S50. $^{1}H,^{13}C$-HMBC NMR spectrum of *mer*-[$Re^{V}NCl_2$(κ^4-*As*,*CC*,*As*-L^{iPr})] in $CDCl_3$. Zoom on the aromatic region.

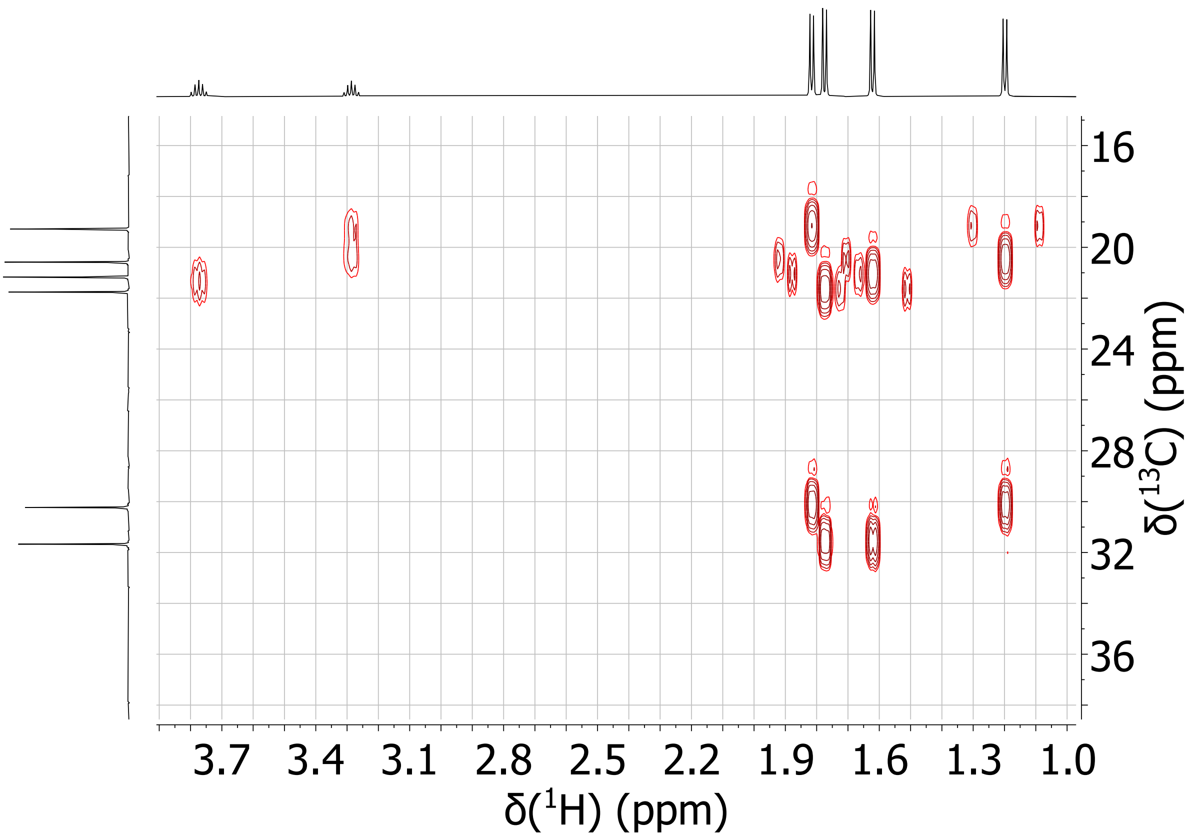

Figure S51. ^{1}H,^{13}C-HMBC NMR spectrum of *mer*-[Re^{V}NCl$_2$(κ^{4}-*As*,*CC*,*As*-L^{iPr})] in $CDCl_3$. Zoom on the aliphatic region.

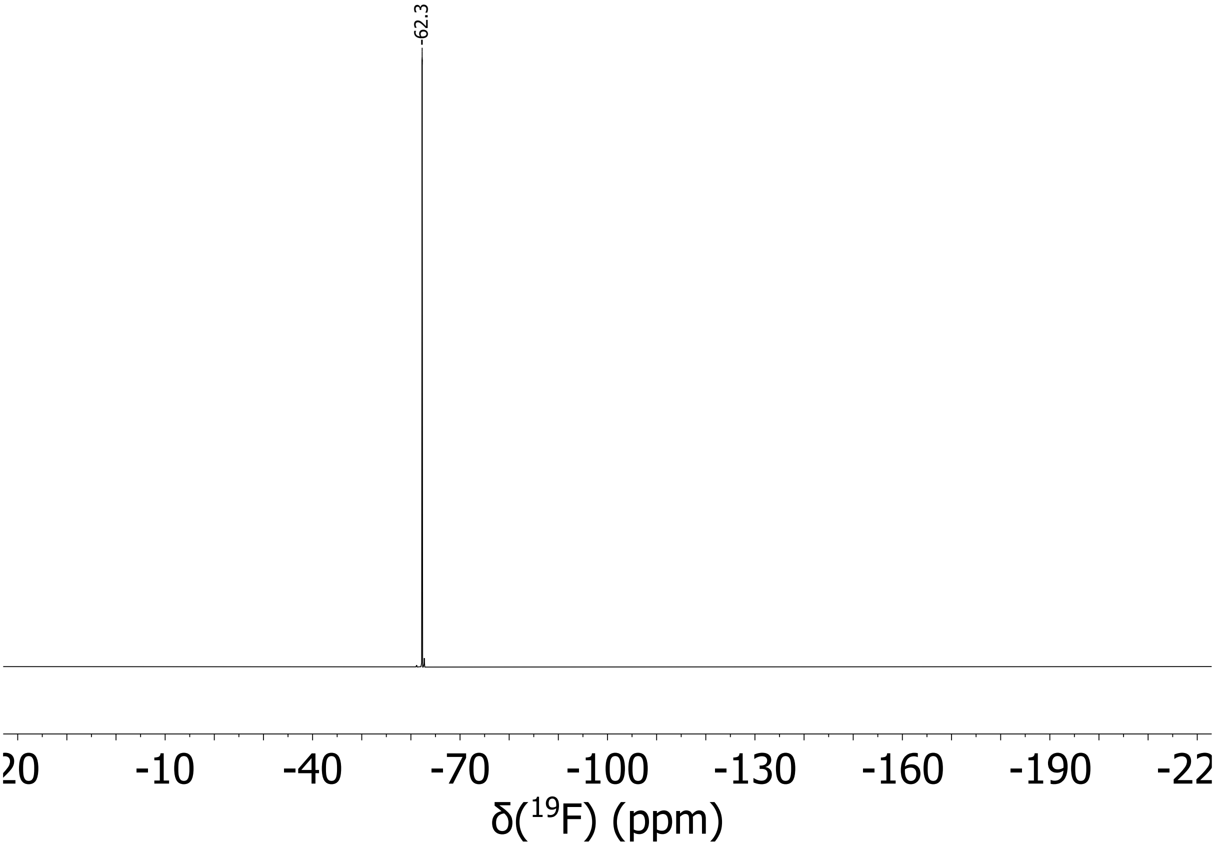

Figure S52. ^{19}F NMR spectrum of *mer*-[Re^{V}NCl$_2$(κ^{4}-*As*,*CC*,*As*-L^{iPr})] in $CDCl_3$.

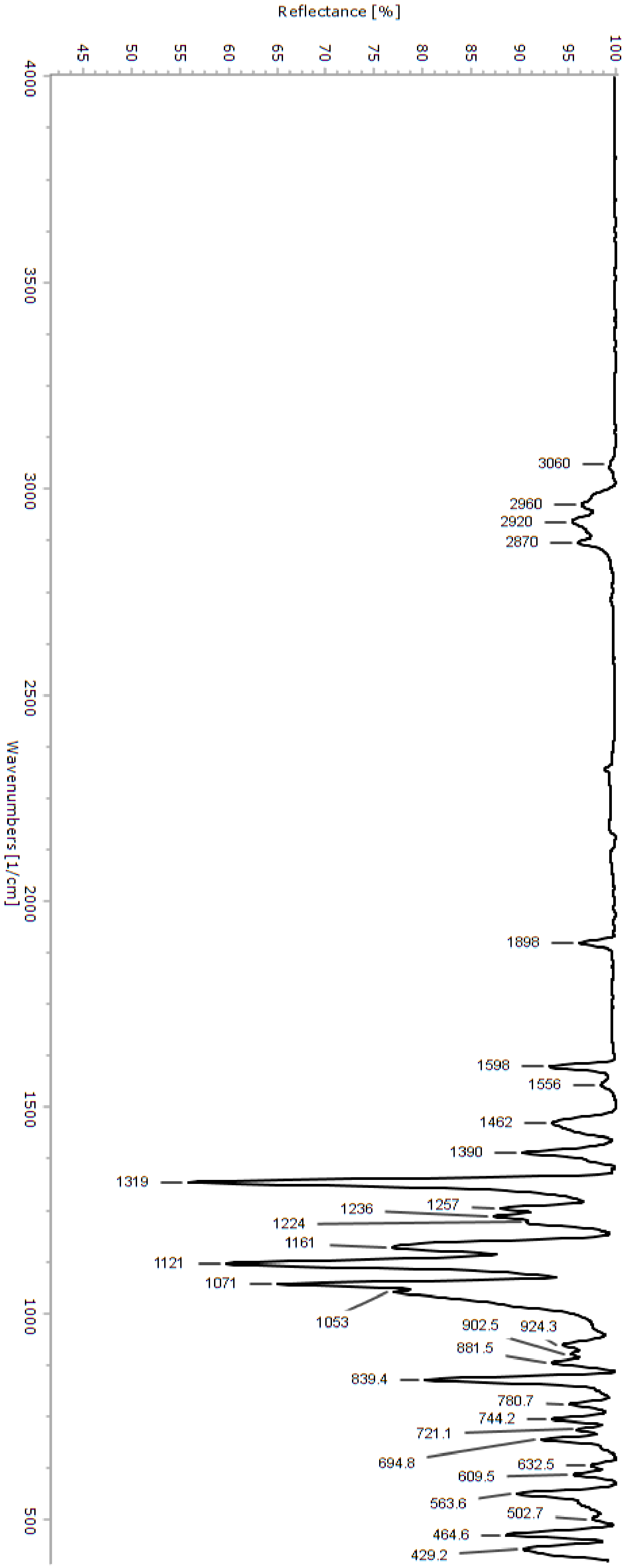

Figure S53. IR spectrum of *mer*-[$Re^{V}NCl_2$(κ^4-*As*,*CC*,*As*-L^{iPr})].

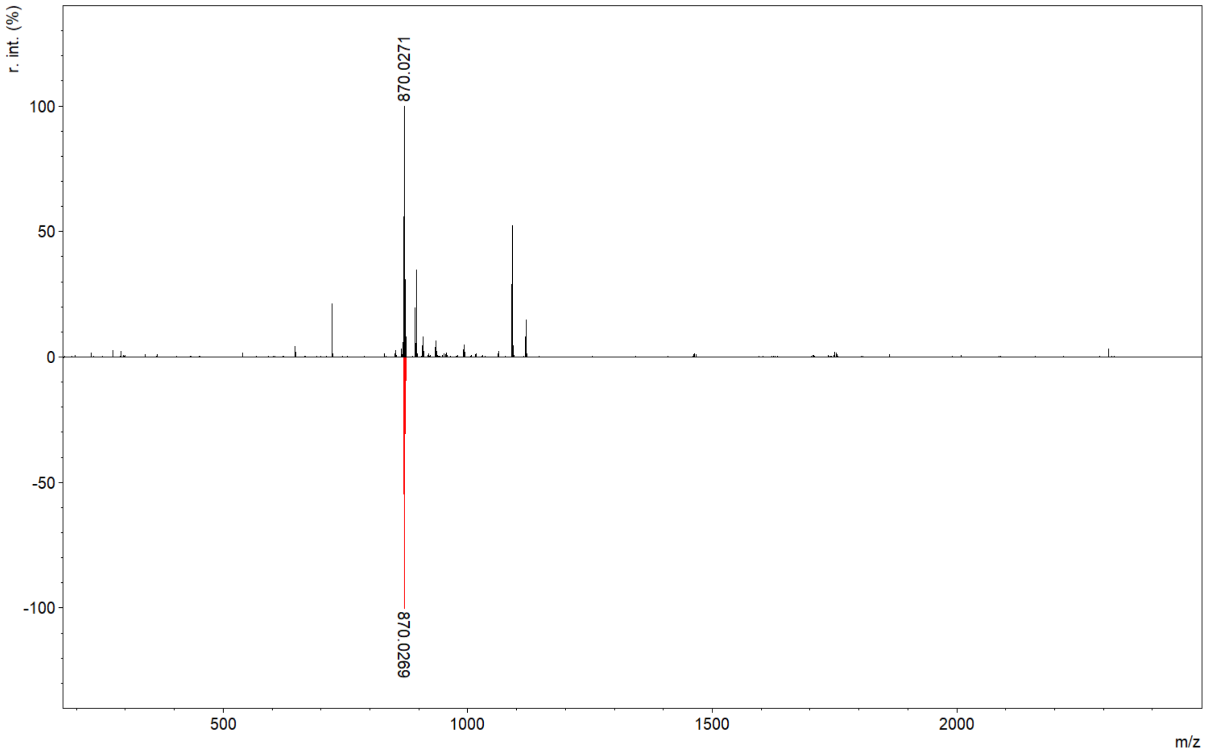

Figure S54. Full ESI$^+$ mass spectrum of *mer*-[Re^VNCl_2(κ^4-*As*,*CC*,*As*-L^{iPr})]. Experimental spectrum: top (black); simulated spectrum: bottom (red).

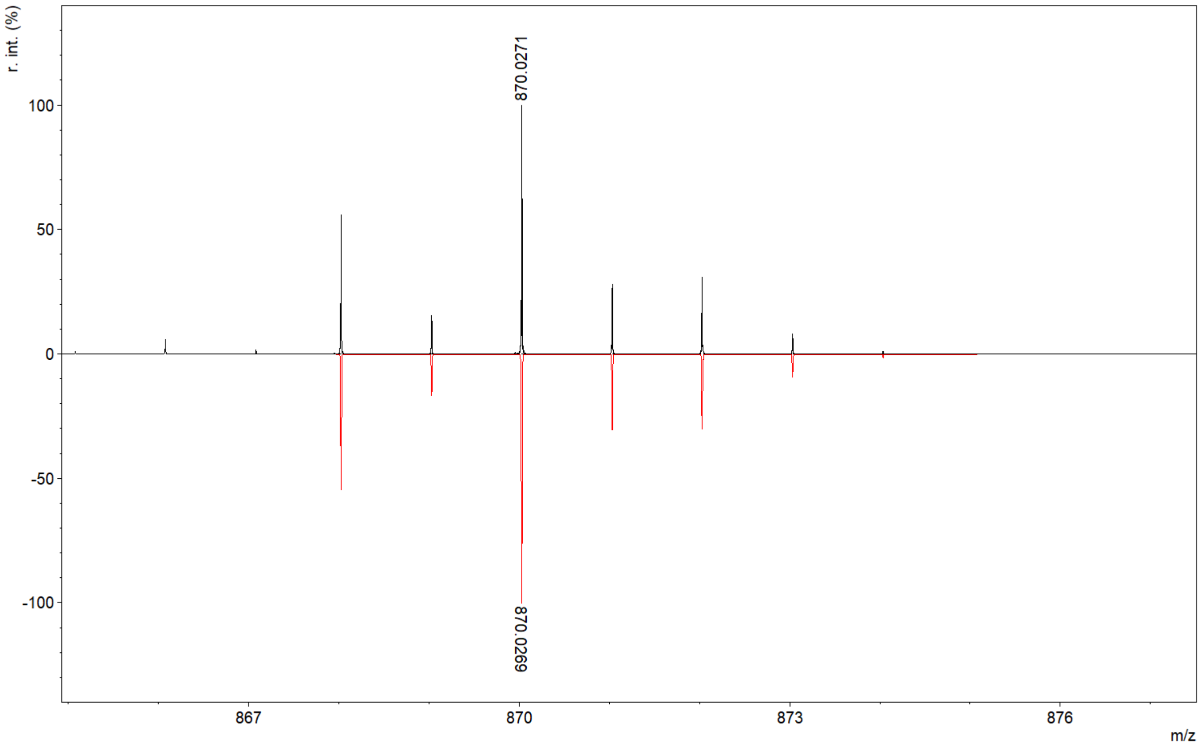

Figure S55. Zoom on the isotopic pattern of the [M-Cl]$^+$ ion of *mer*-[Re^VNCl_2(κ^4-*As*,*CC*,*As*-L^{iPr})] in the ESI$^+$ mass spectrum. Experimental spectrum: top (black); simulated spectrum: bottom (red).

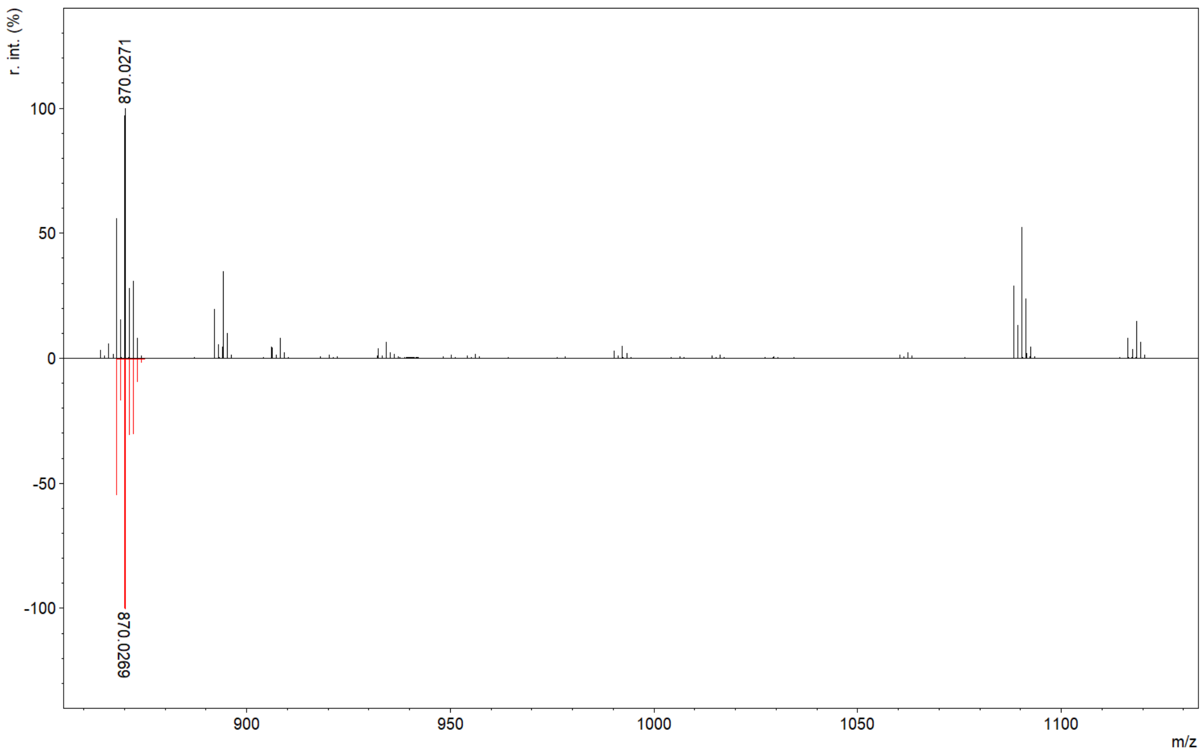

Figure S56. Zoom on additional, unidentified rhenium-containing species in the ESI$^+$ mass spectrum of *mer*-[$Re^V NCl_2$(κ^4-*As*,*CC*,*As*-L^{iPr})]. Experimental spectrum: top (black); simulated spectrum: bottom (red).

S3.7 *mer*-[Re^VCl_3(κ[4]-*As*,*CC*,*As*-L^{iPr})].

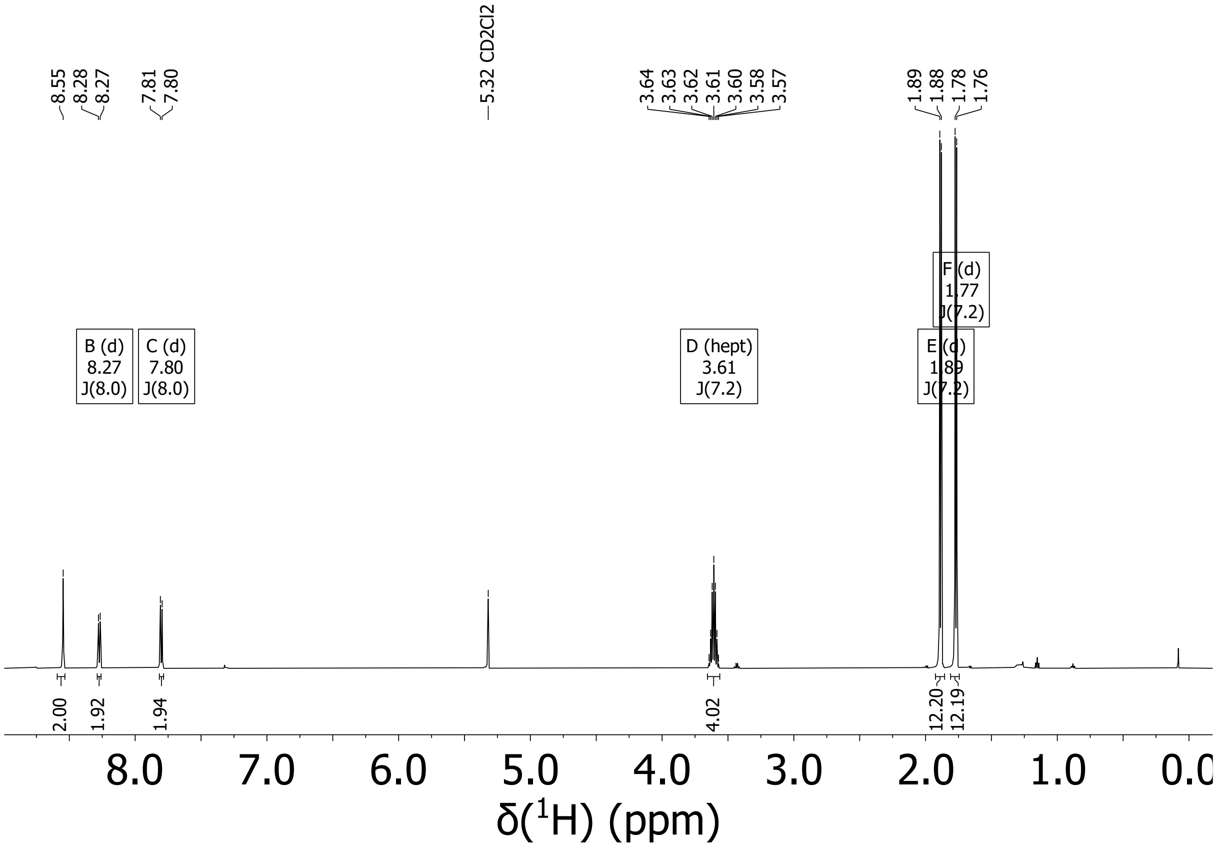

Figure S57. ^1H NMR spectrum of *mer*-[Re^VCl_3($κ^4$-*As*,*CC*,*As*-L^{iPr})] in CD_2Cl_2.

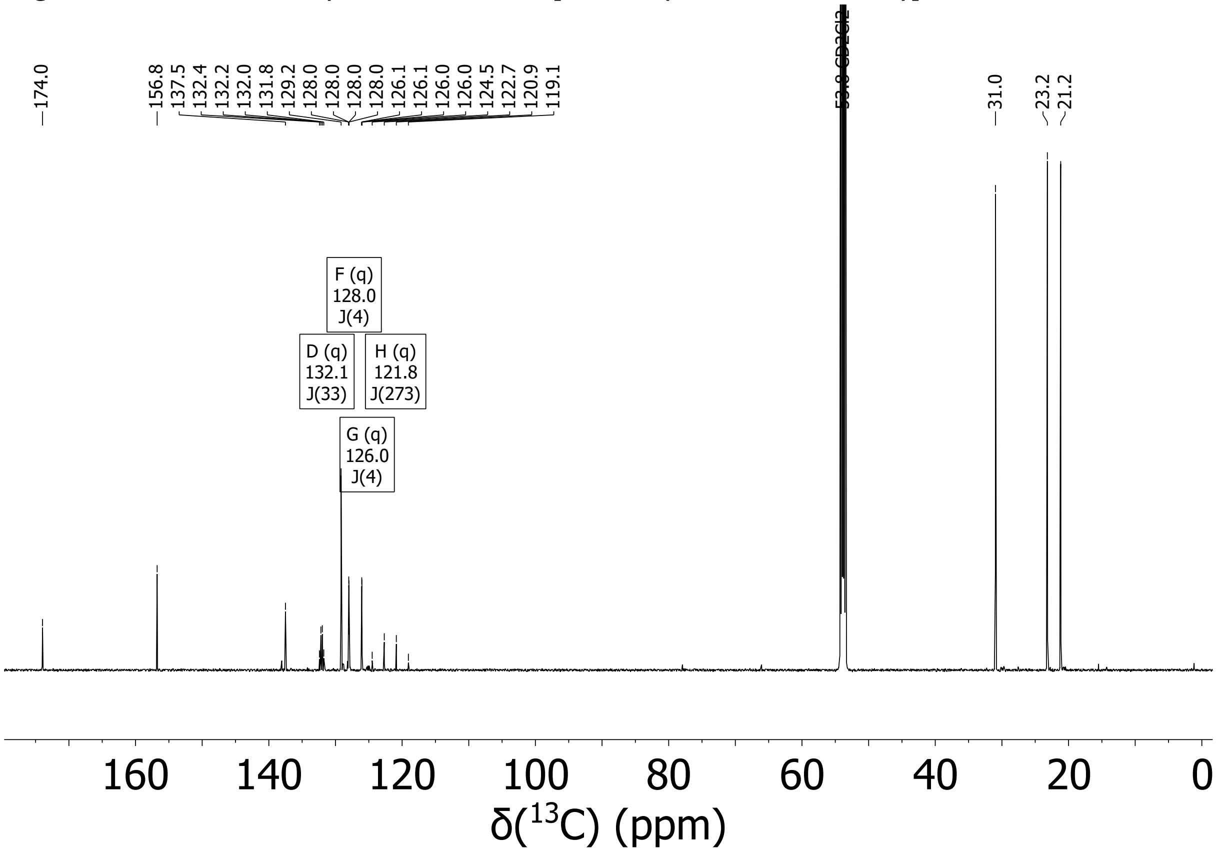

Figure S58. $^{13}C\{^1H\}$ NMR spectrum of *mer*-[Re^VCl_3($κ^4$-*As*,*CC*,*As*-L^{iPr})] in CD_2Cl_2.

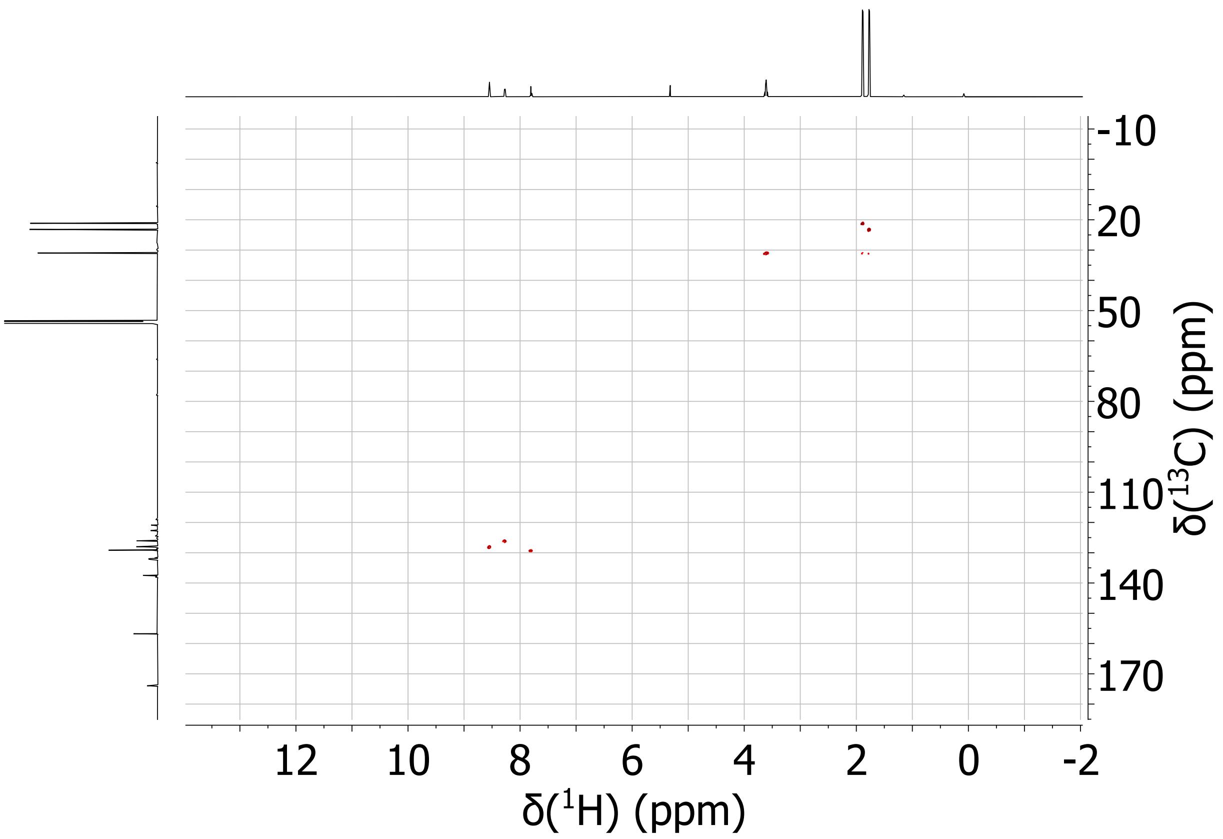

Figure S59. ^{1}H,^{13}C-HSQC NMR spectrum of *mer*-[Re^{V}Cl$_3$(κ^4-*As*,*CC*,*As*-L^{iPr})] in CD_2Cl_2.

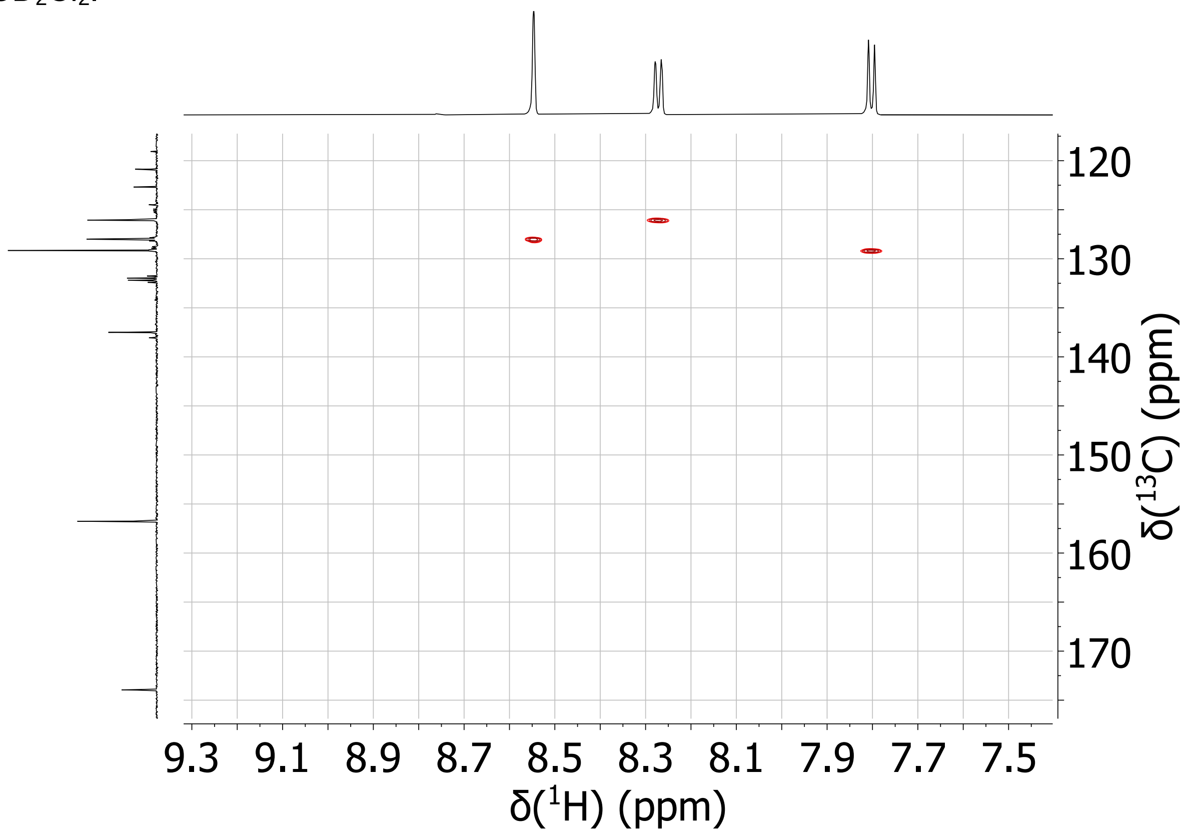

Figure S60. ^{1}H,^{13}C-HSQC NMR spectrum of *mer*-[Re^{V}Cl$_3$(κ^4-*As*,*CC*,*As*-L^{iPr})] in CD_2Cl_2. Zoom on the aromatic region.

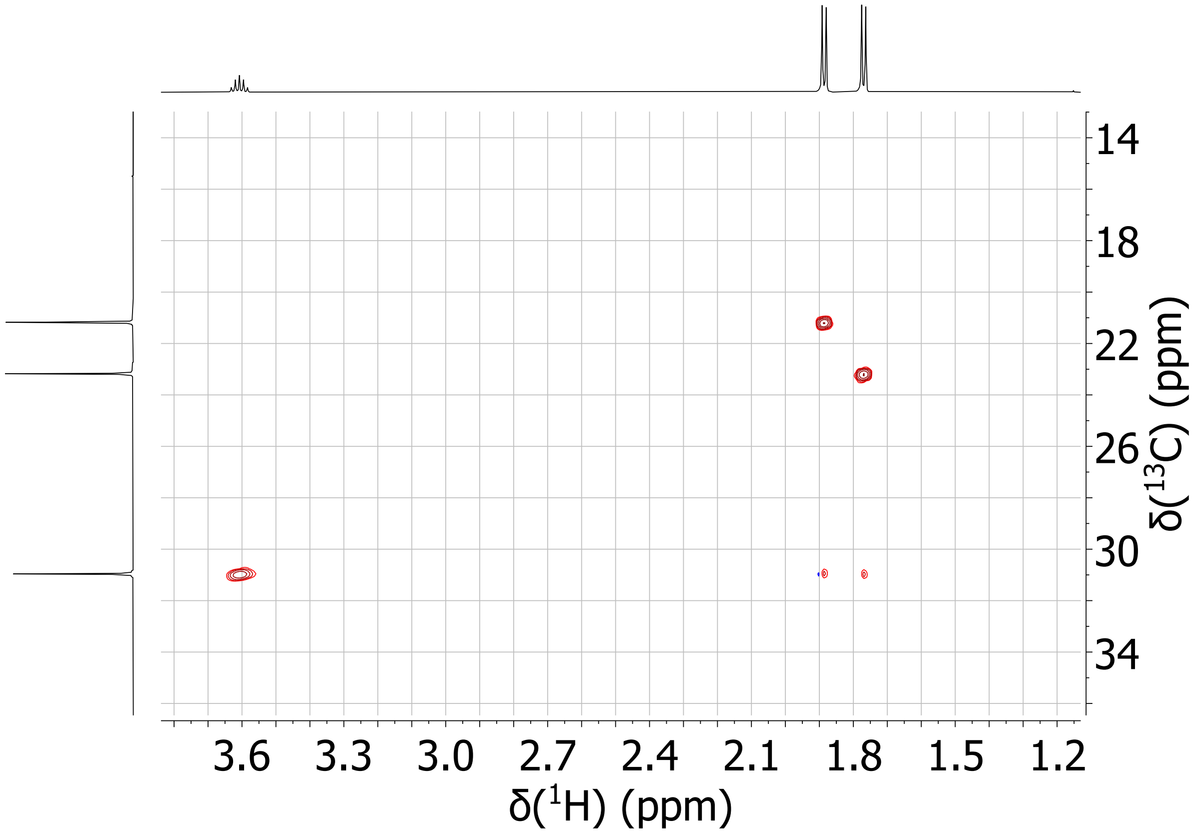

Figure S61. $^{1}H,^{13}C$-HSQC NMR spectrum of *mer*-[Re^{V}Cl$_3$(κ^4-*As*,*CC*,*As*-L^{iPr})] in CD_2Cl_2. Zoom on the aliphatic region.

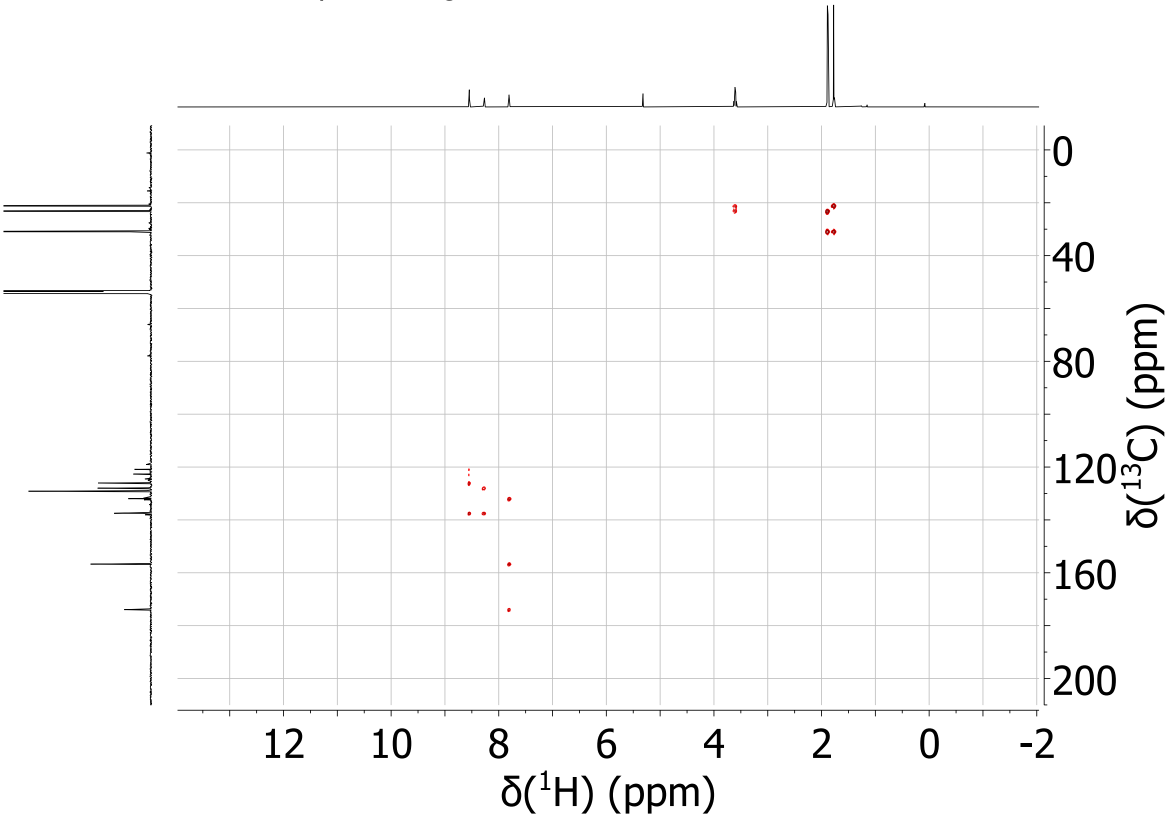

Figure S62. $^{1}H,^{13}C$-HMBC NMR spectrum of *mer*-[Re^{V}Cl$_3$(κ^4-*As*,*CC*,*As*-L^{iPr})] in CD_2Cl_2.

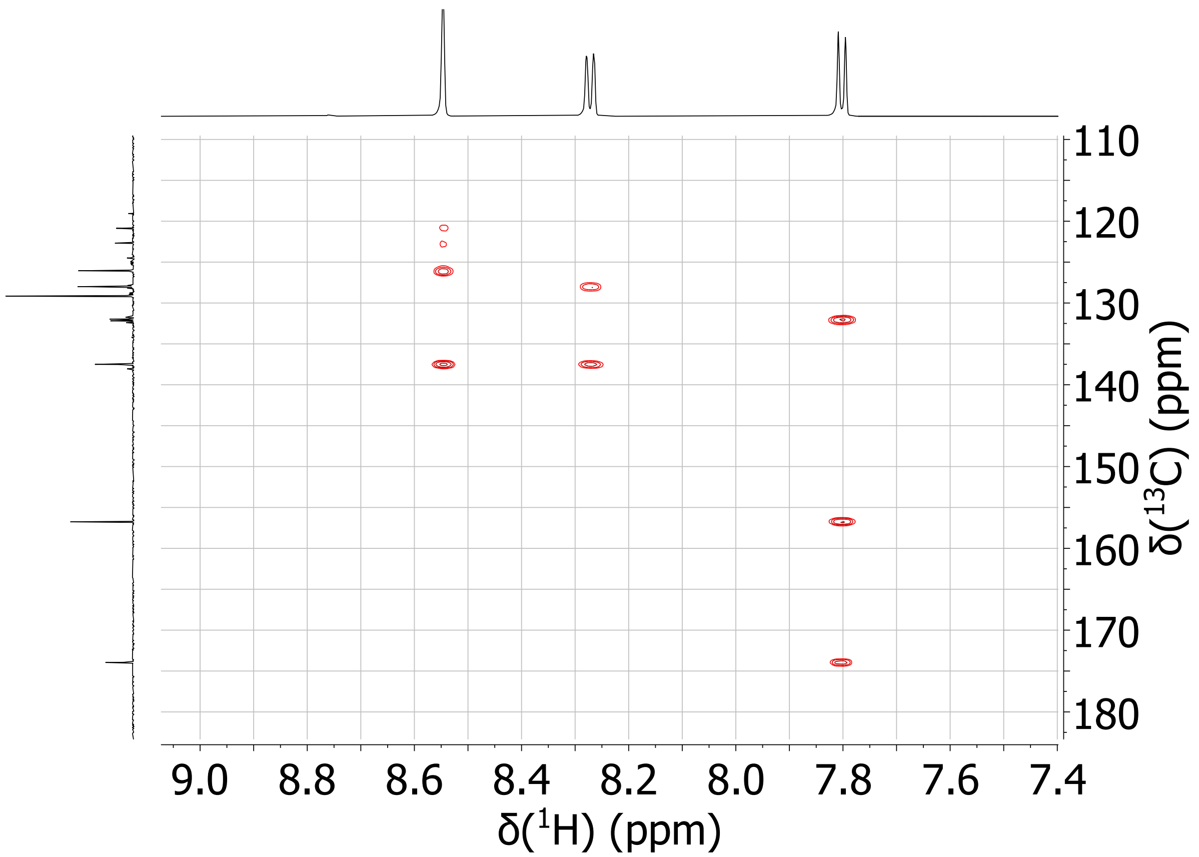

Figure S63. $^{1}H,^{13}C$-HMBC NMR spectrum of *mer*-[Re^{V}Cl$_3$(κ^4-*As*,*CC*,*As*-L^{iPr})] in CD$_2$Cl$_2$. Zoom on the aromatic region.

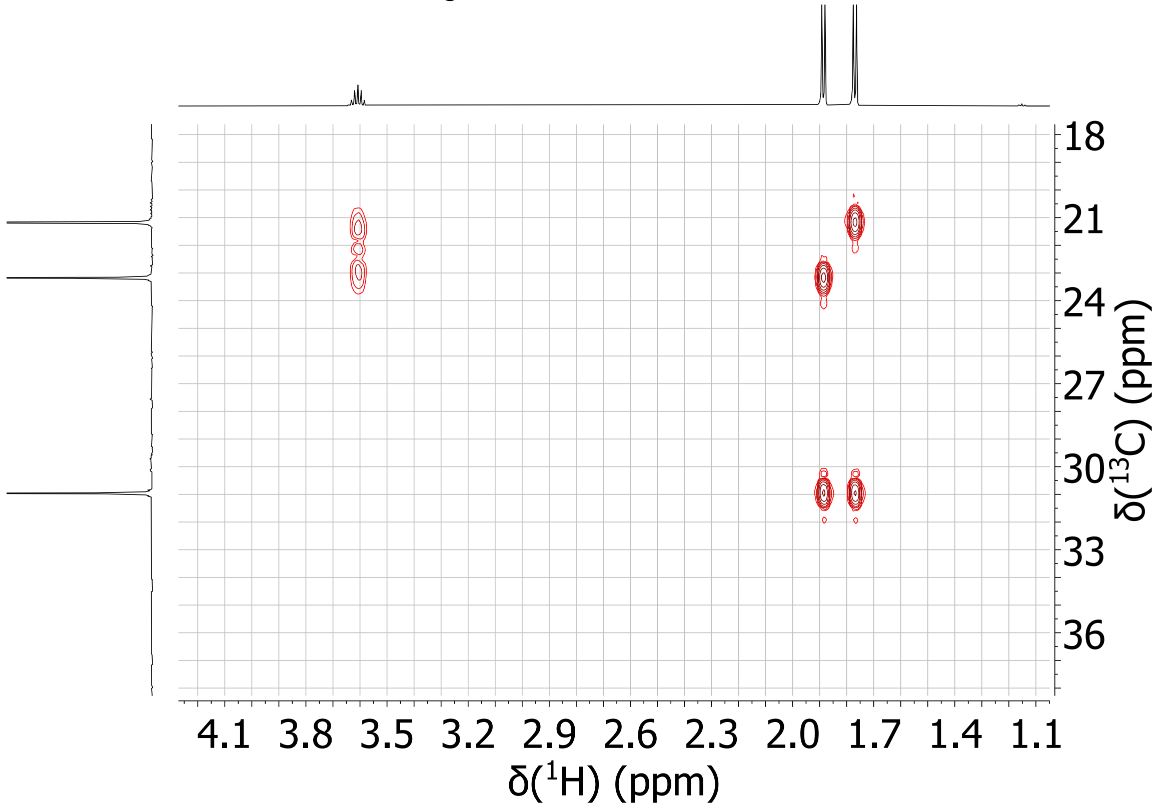

Figure S64. $^{1}H,^{13}C$-HMBC NMR spectrum of *mer*-[Re^{V}Cl$_3$(κ^4-*As*,*CC*,*As*-L^{iPr})] in CD$_2$Cl$_2$. Zoom on the aliphatic region.

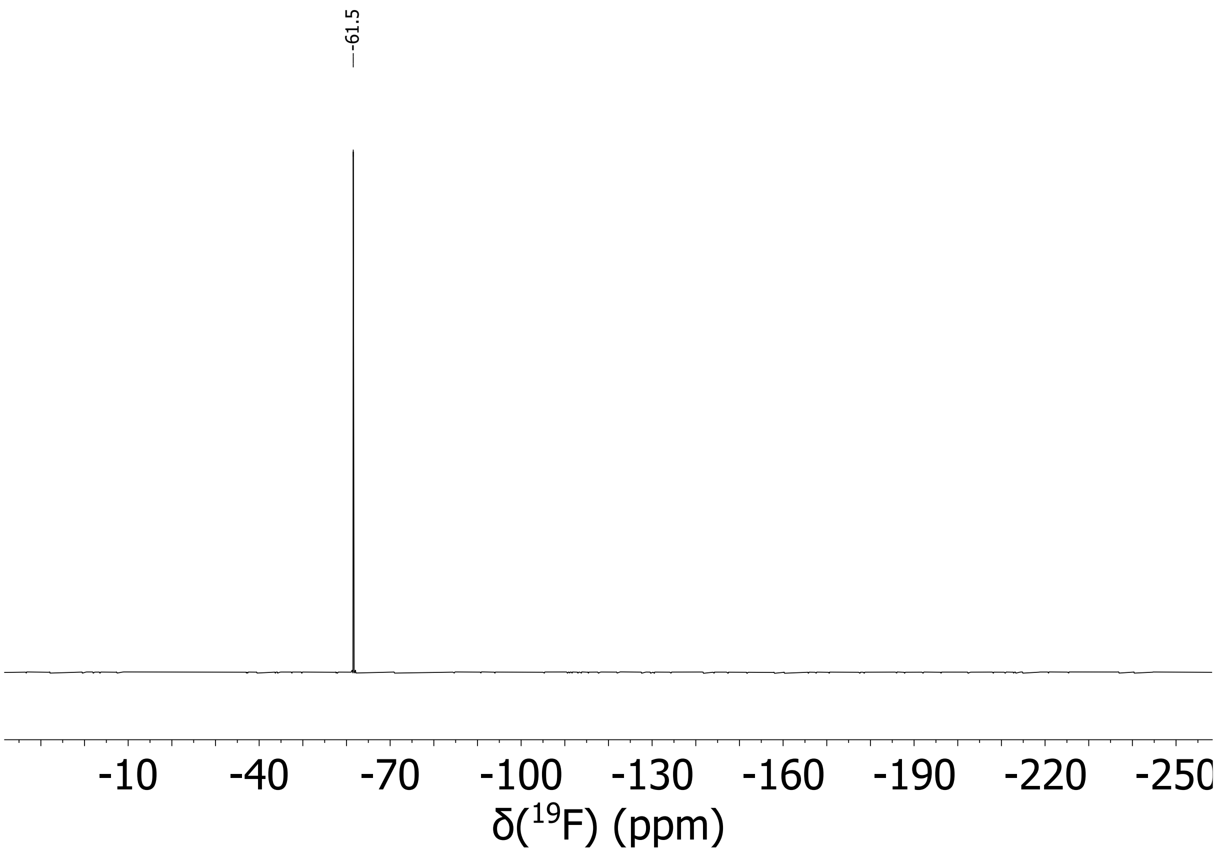

Figure S65. ^{19}F NMR spectrum of *mer*-[$Re^{V}Cl_3$(κ^4-*As*,*CC*,*As*-L^{iPr})] in CD_2Cl_2.

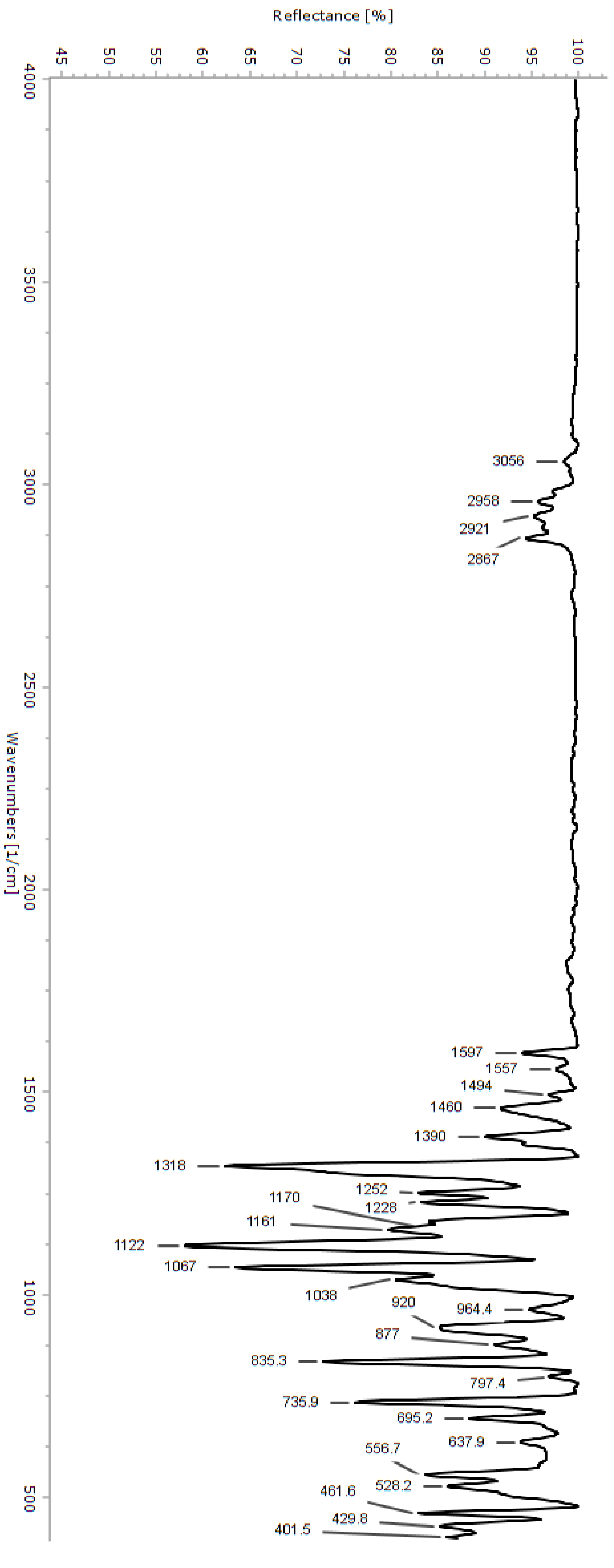

Figure S66. IR spectrum of *mer*-[$Re^{V}Cl_3$(κ^4-*As*,*CC*,*As*-L^{iPr})].

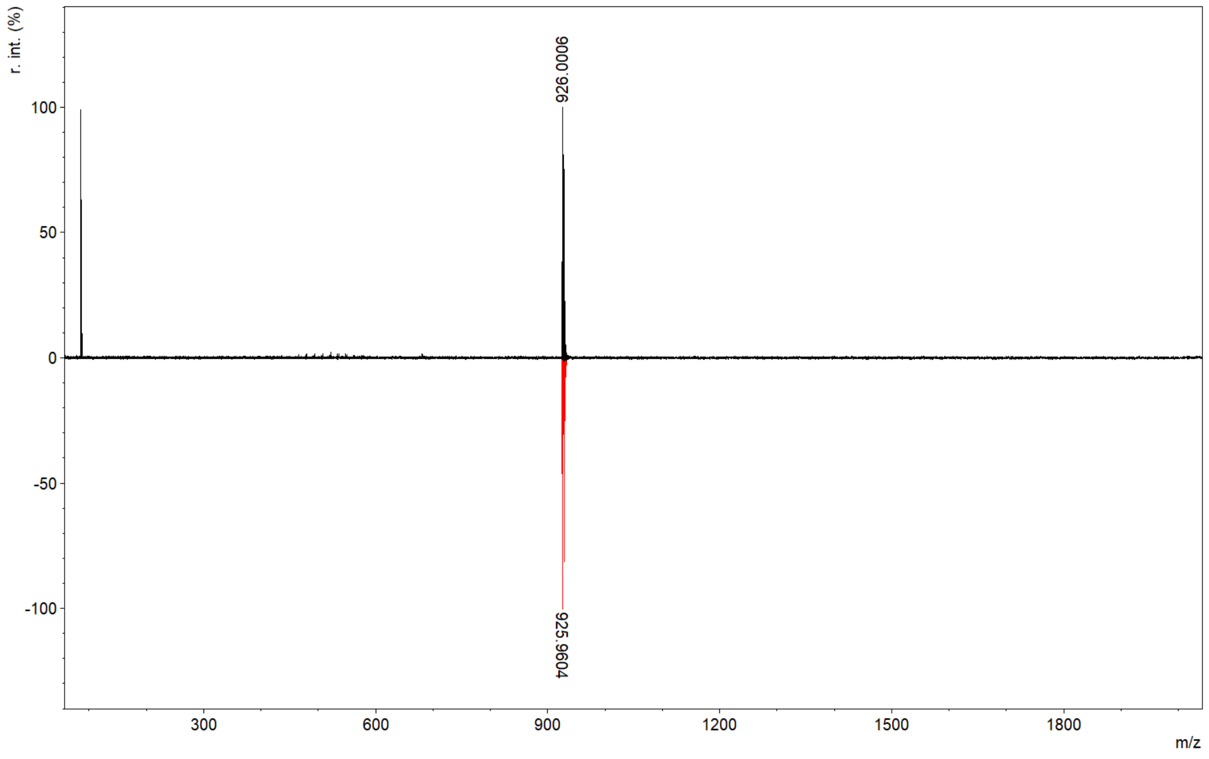

Figure S67. Full liquid injection field desorption ionization (LIFDI) mass spectrum of *mer*-[$Re^{V}Cl_3$(κ^4-*As*,*CC*,*As*-L^{iPr})] in positive ion mode. Experimental spectrum: top (black); simulated spectrum: bottom (red).

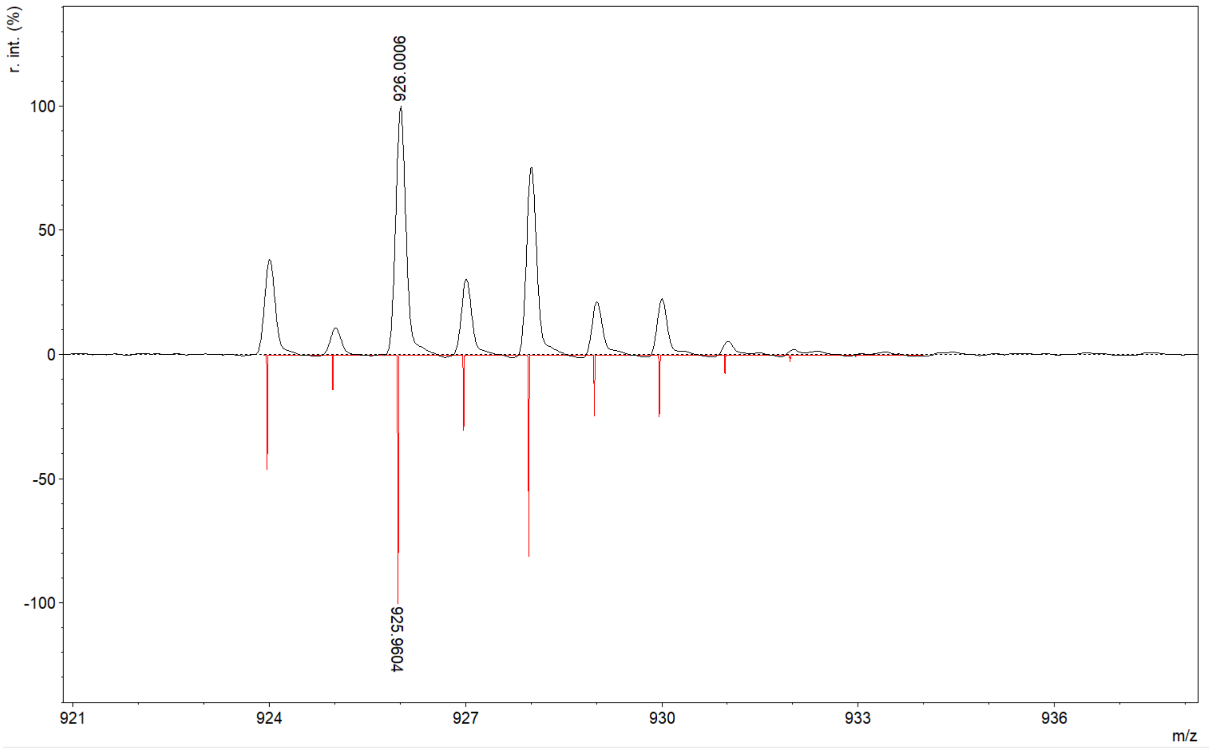

Figure S68. Zoom on the isotopic pattern of the $[M]^+$ ion of *mer*-[$Re^{V}Cl_3$(κ^4-*As*,*CC*,*As*-L^{iPr})] in the liquid injection field desorption ionization (LIFDI) mass spectrum in positive ion mode. Experimental spectrum: top (black); simulated spectrum: bottom (red).

S3.8 *mer*-[^{99m}Tc^{V}NCl$_2$(κ^4-*As*,*CC*,*As*-L^{iPr})].

The gradient elution system utilized mobile phase A (100% MeCN + 0.1% TFA) and mobile phase B (deionized H_2O + 0.1% TFA) at a flow rate of 0.8 mL/min, starting with 10% A/90% B changing within 30 minutes to 90% A/10% B.

Integration C:\Gina Data & Methods\Measurements\Forschung\Azura\9010-1090-30min_H2OMeCN Position 2\250428_2_

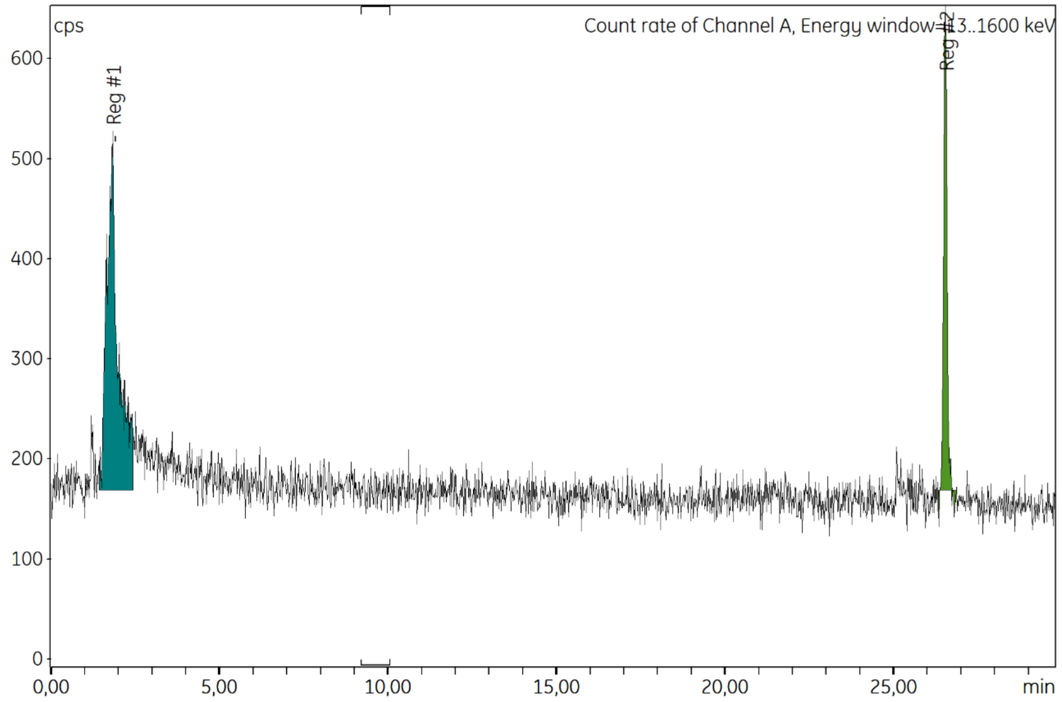

Figure S69. HPLC chromatogram of purified *mer*-[^{99m}Tc^{V}NCl$_2$(κ^4-As,CC,As-L^{iPr})] (Reg #2: 26.55 min; γ trace).

Integration C:\Gina Data & Methods\Measurements\Forschung\Azura\9010-1090-30min_H2OMeCN Position 2\250429_lig

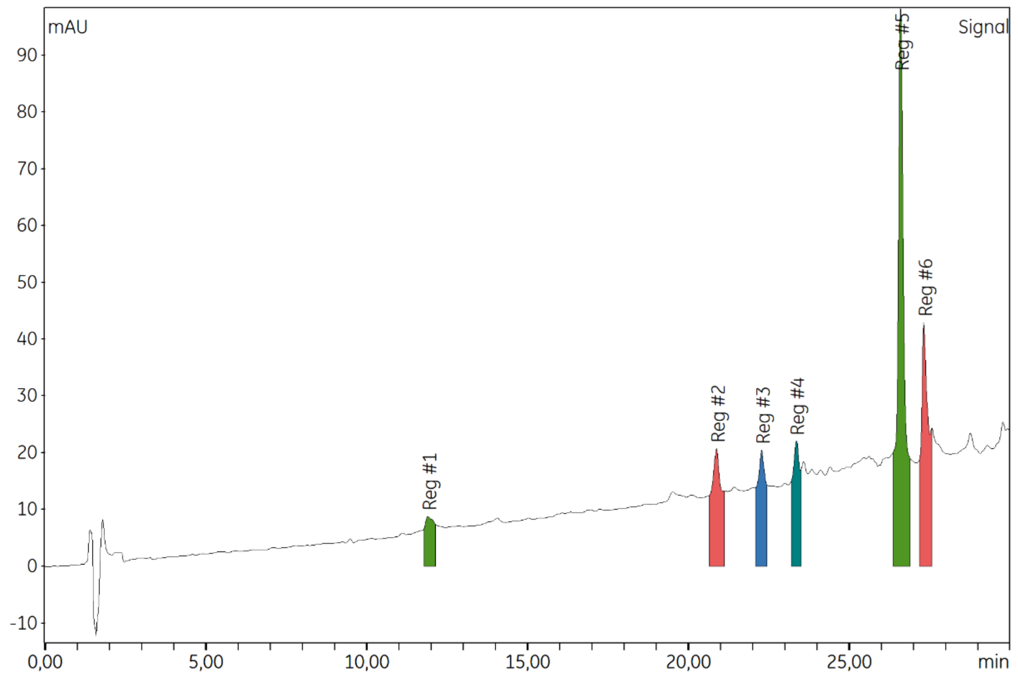

Figure S70. HPLC chromatogram of L^{iPr} (Reg #5: 26.62 min; UV trace).

Integration C:\Gina Data & Methods\Measurements\Forschung\Azura\9010-1090-30min_H2OMeCN Position 2\250428_Tc

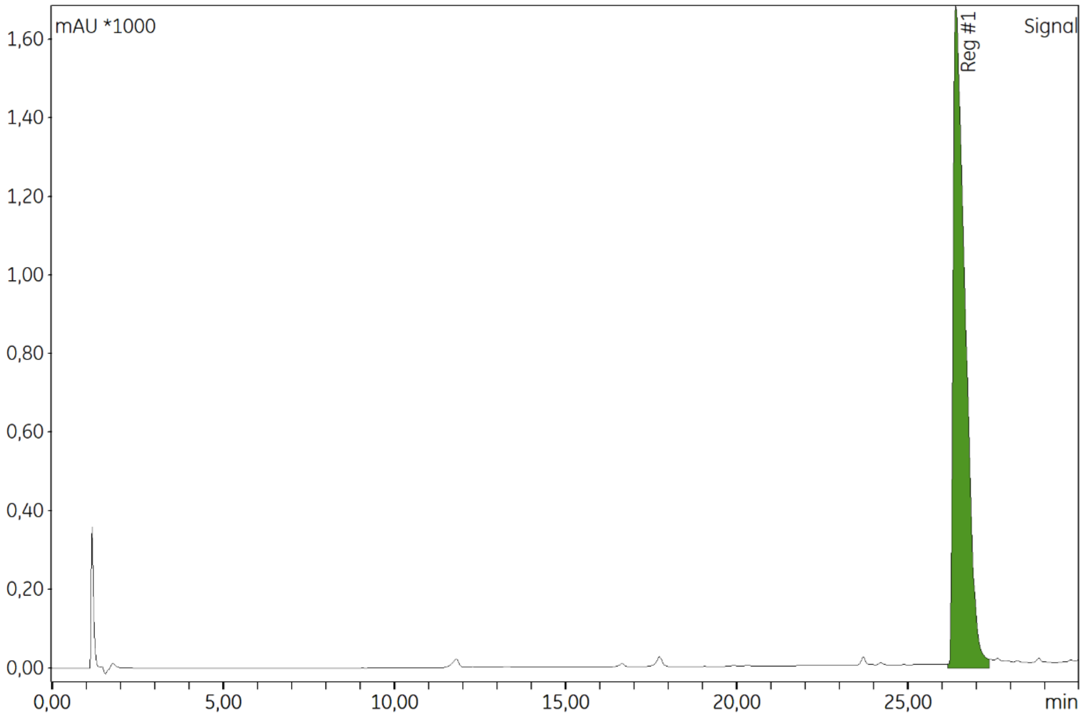

Figure S71. HPLC chromatogram of *mer*-[$Re^{V}NCl_2(\kappa^4$-As,CC,As-$L^{iPr})$] (Reg #1: 26.62 min; UV trace).

Integration C:\Gina Data & Methods\Measurements\Forschung\Azura\9010-1090-30min_H2OMeCN Position 2\250428_Tc

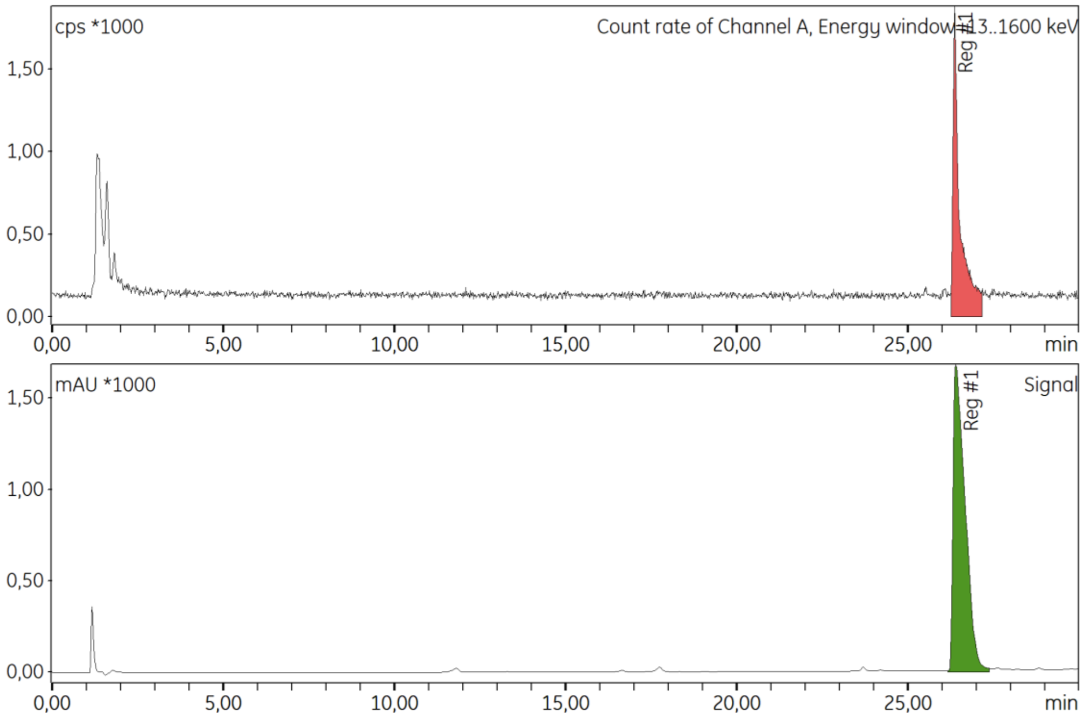

Figure S72. HPLC chromatogram of co-injected *mer*-[$M^{V}NCl_2(\kappa^4$-As,CC,As-$L^{iPr})$] (M = ^{99m}Tc, Re; top: γ trace; bottom: UV trace); solution of *mer*-[$Re^{V}NCl_2(\kappa^4$-As,CC,As-$L^{iPr})$] (1 mg) and crude *mer*-[$^{99m}Tc^{V}NCl_2(\kappa^4$-As,CC,As-$L^{iPr})$] (20 µL) in $NCCH_3$ (500 µL).

Part S4: Computational data

S4.1 Computational details: L^{iPr} complexes

DFT calculations were performed on the high-performance computing systems of the Freie Universität Berlin ZEDAT (Curta),[110] and of the state of Baden-Württemberg (bwFOR cluster JUSTUS) using the program package GAUSSIAN 16.[111] The gas phase geometry optimizations were performed using coordinates derived from the X-ray crystal structures using *GAUSSVIEW* and *Avogadro.*[112, 113] Frequency calculations following the geometry optimization were performed to validate the obtained geometries as energetic minima and showed no imaginary frequencies in all cases. The calculations were performed with the hybrid density functional B3LYP with Grimme dispersion and Becke-Johnson damping,[114] as implemented in GAUSSIAN.[115-117] The triple-ζ (pseudo)potential basis set def2-TZVP was used for all atoms.[118-120] The basis sets as well as the ECPs were obtained from the basis set exchange database.[121] NBO6.0 was used as included in GAUSSIAN. Further analyses were performed with the free multifunctional wavefunction analyzer *Multiwfn*.[122] Electron delocalization range function (EDR) and interaction region indicator (IRI) definitions can be found in references [123-125]. EDR provides a visual measure for electron delocalization, while IRI directly confirms regions of interaction between fragments as well as their extend. Topological features are interpreted according to the quantum theory of atoms in molecules (QTAIM). Oxidation states were be calculated by localized orbital bonding analysis (LOBA) for atoms and chemically sensitive fragments (for discussion about the validity of oxidation state calculations see: references [99], [100]).[98] The properties at the (3,-1) critical points allow an evaluation of the closed- or shared-shell nature of bonds and other bond-specific classifications such as covalency based on the electron density ρ(r), the ellipticity $\varepsilon(r) = [\lambda_1(r)/\lambda_2(r)]-1$ where λ_1(r) and λ_2(r) are the lowest and the second lowest eigenvalues of the Hessian matrix of ρ(r), the ratio values between the perpendicular and the parallel curvatures – a covalency index $\eta(r) = |\lambda_1(r)|/\lambda_3(r)$ with λ_1(r) and λ_3(r) as the lowest and the highest eigenvalues of the Hessian matrix of ρ(r), the Laplacian of the electron density ($\nabla^2\rho(r)$), the kinetic energy density ratio ($G(r)/\rho(r)$), and the total energy density ratio ($H(r)/\rho(r)$; $H(r) = G(r) + V(r)$).[93, 125-132] For typical closed-shell interactions (i.e. ionic bonds, hydrogen bonds, Van der Waals interactions), ρ_b is small, while $\nabla^2\rho_b$ is positive, H(r) is positive and $\eta(r) << 1$. In contrast for a shared-shell interaction (i.e. covalent bonds), ρ_b is large, H(r) is negative and $\eta(r) >> 1$, while $\nabla^2\rho_b$ is positive, if the bond is nonpolar and negative if the bond is considerably polarized (typically $\eta(r) \approx 1$).[126-133] The M-C bonds in the present study show significant polar, covalent character. The M-C bond is expectedly most covalent with a simultaneously significantly reduced ε (resulting from the reduced π-bond fraction for both M-C and C≡C bonds) for the 4e$^-$ donor complex *mer*-[$Re^{V}Cl_3$(*As*,*CC*,*As*-L^{iPr})]. In contrast, weaker and more π-heavy M-C and C≡C bonds are observed in the classic 2e$^-$ donor alkyne complexes of technetium, including *mer*-[$Tc^{III}Cl_3$(*As*,*CC*,*As*-L^{iPr})], and the rhenium nitrido complex. Spin densities were calculated using *Multiwfn* and visualized with the visual molecular dynamics (*VMD*) package.[134] Complete active space self-consistent field (CAS-SCF) calculations were performed in the Orca 6.1.1 program suite,[135-146] based on the optimized structures of *mer*-[$Re^{V}Cl_3$(*As*,*CC*,*As*-L^{iPr})] and *mer*-[$Tc^{III}Cl_3$(*As*,*CC*,*As*-L^{iPr})] (B3LYP/def2-TZVP). The active space was constructed from DFT-derived localized orbitals limited to π-type alkyne orbitals ($\pi_{\parallel}$, $\pi_{\parallel}$*, $\pi_{\perp}$, $\pi_{\perp}$*) and the five metal d orbitals, therefore yielding a CAS(8,9) configuration. To achieve computational feasibility other ligand contributions were omitted from the active space. Some external ligand orbital contamination was observed in the CAS orbitals, which could not be eliminated. IBOView was used for visualization.[147, 148]

Table S2. DFT-based free energy preferences for the potential electronic isomers of *mer*-[MCl_3(*As*,*CC*,*As*-L^{iPr})] (M = Mn, Tc, Re). The most stable electronic configuration in each respective case is bold; the differences of the minimum to the higher energy configurations is given.

		Mn		Tc		Re	
configuration; ox. state	multiplicity	Δ*G* [Hartree]	ΔΔ*G* [kJ/mol]	Δ*G* [Hartree]	ΔΔ*G* [kJ/mol]	Δ*G* [Hartree]	ΔΔ*G* [kJ/mol]
d^2; M^V	1	-8690.4535	+245	-7620.3699	+25	**-7617.8679**	
d^4-ls; M^{III}	3	-8690.5100	+96	**-7620.3794**		-7617.8668	+3
d^4-hs; M^{III}	5	**-8690.5467**		-7620.2951	+222	-7617.7834	+222

Table S3. CAS-SCF states of *mer*-[$ReCl_3$(*As*,*CC*,*As*-L^{iPr})]. The most stable electronic configuration in each respective case is bold; the differences of the minimum to the higher energy configurations is given.

**CAS-SCF STATES (Mult = 1)**	CAS-SCF STATES (Mult = 3)
**---**	---
**ROOT 0: *E*= -7602.0782822257 Eh**	ROOT 0: *E*= -7602.0661964618 Eh
**0.88432 [0]: 222200000**	0.90121 [0]: 222110000
**0.01434 [168]: 212110100**	0.01384 [424]: 202110200
**0.01229 [6]: 222020000**	0.01093 [805]: 121110011
**0.01091 [436]: 202200200**	0.00938 [86]: 220110020
**0.00959 [214]: 211210010**	0.00936 [145]: 212120000
**0.00909 [847]: 121200011**	0.00922 [1018]: 112110110
**0.00866 [1060]: 112200110**	0.00730 [154]: 212100200
**0.00815 [83]: 220200020**	0.00515 [207]: 211120010
**0.00499 [427]: 202220000**	0.00436 [6]: 222010100
**0.00437 [1054]: 112210001**	0.00415 [1765]: 022110020
**0.00413 [36]: 221100110**	0.00323 [23]: 221110010
**0.00357 [3]: 222100100**	
**0.00350 [1812]: 022200020**	

Table S4. CAS-SCF states of *mer*-[$TcCl_3$(*As*,*CC*,*As*-L^{iPr})]. The most stable electronic configuration in each respective case is bold; the differences of the minimum to the higher energy configurations is given.

CAS-SCF STATES (MULT= 1)	**CAS-SCF STATES (MULT= 3)**
---	**---**
ROOT 0: *E*= -7604.5872044568 Eh	**ROOT 0: *E*= -7604.6163148495 Eh**
0.82869 [0]: 222200000	**0.90400 [0]: 222110000**
0.06581 [6]: 222020000	**0.01689 [803]: 121110101**
0.01408 [845]: 121200101	**0.01548 [427]: 202110020**
0.01277 [439]: 202200020	**0.01244 [1018]: 112110110**
0.01243 [213]: 211210100	**0.00714 [1762]: 022110200**
0.01102 [1060]: 112200110	**0.00714 [83]: 220110200**
0.00706 [80]: 220200200	**0.00487 [22]: 221110100**
0.00580 [1809]: 022200200	**0.00402 [206]: 211120100**
0.00393 [1054]: 112210001	
0.00264 [27]: 221111000	
0.00262 [169]: 212110010	

Table S5. Exp. *vs.* calc. C≡C and M-C bond lengths in *mer*-[MX_3(κ^4-*As*,*CC*,*As*-L^{iPr})] (M = Tc, Re) (X_3 = NCl_2, NBr_2, N(CN)Cl for M = Tc and Cl_3, NCl_2 for M = Re). Most stable configuration for X_3 = Cl_3 is bold.

	Experimental [Å]		Calculated [Å]	
**core**	**M-C$_{avg.}$**	**C≡C**	**M-C$_{avg.}$**	**C≡C**
{$Tc^{V}NBr_2$}	2.194	1.250	2.181	1.258
{$Tc^{V}NCl_2$}	2.163	1.254	2.186	1.258
{Tc^{V}N(CN)Cl}	2.166	1.254	2.211	1.254
{$Tc^{III}Cl_3$} (s, **ls**, hs)	2.122	1.243	1.992, **2.141**, 2.164	1.303, **1.265**, 1.334
{$Re^{V}NCl_2$}	2.133	1.262	2.156	1.271
{$Re^{V}Cl_3$} (**s**, ls, hs)	1.990	1.309	**2.002**, 2.131, 2.159	**1.313**, 1.276, 1.346

Table S6. Properties at the bond critical points of some selected bonds including the electron density ρ_b, the Laplacian $\nabla^2\rho_b$, eigenvalues of the Hessian matrix λ_1, λ_2 and λ_3, the Lagrangian energy density *G*(*r*), potential energy density *V*(*r*) and total energy density *H*(*r*). For M-C bonds two values represent the two individual M-C (3,-1) critical points for *mer*-[MX_3(κ^4-*As*,*CC*,*As*-L^{iPr})] (M = Tc, Re) (X_3 = NCl_2, NBr_2, N(CN)Cl for M = Tc and Cl_3, NCl_2 for M = Re).

core	bond	ρ_b	$\nabla^2\rho_b$	λ_1	λ_2	λ_3	*G*(*r*)	*V*(*r*)	*H*(*r*)
{$Tc^{V}NBr_2$}	C≡C	0.389	-1.150	-0.740	-0.669	0.259	0.224	-0.735	-0.511
	M-C	0.096<br>0.096	0.159<br>0.162	-0.121<br>-0.120	-0.076<br>-0.074	0.356<br>0.355	0.071<br>0.071	-0.102<br>-0.102	-0.031<br>-0.031
{$Tc^{V}NCl_2$}	C≡C	0.388	-1.144	-0.736	-0.667	0.258	0.225	-0.736	-0.511
	M-C	0.096<br>0.095	0.157<br>0.160	-0.120<br>-0.119	-0.076<br>-0.074	0.353<br>0.353	0.07<br>0.07	-0.1<br>-0.1	-0.031<br>-0.03
{Tc^{V}N(CN)Cl}	C≡C	0.390	-1.143	-0.729	-0.668	0.254	0.229	-0.744	-0.515
	M-C	0.090<br>0.089	0.163<br>0.167	-0.11<br>-0.108	-0.067<br>-0.063	0.339<br>0.338	0.067<br>0.067	-0.094<br>-0.093	-0.026<br>-0.026
{$Tc^{III}Cl_3$}_ls	C≡C	0.383	-1.122	-0.718	-0.669	0.266	0.217	-0.714	-0.497
	M-C	0.104<br>0.104	0.167<br>0.168	-0.12<br>-0.12	-0.094<br>-0.094	0.382<br>0.382	0.079<br>0.079	-0.116<br>-0.116	-0.037<br>-0.037
{$Re^{V}NCl_2$}	C≡C	0.379	-1.095	-0.716	-0.650	0.271	0.212	-0.698	-0.486
	M-C	0.111<br>0.111	0.119<br>0.119	-0.140<br>-0.140	-0.100<br>-0.100	0.359<br>0.359	0.075<br>0.075	-0.123<br>-0.123	-0.048<br>-0.048
{$Re^{V}Cl_3$}_s	C≡C	0.351	-0.970	-0.645	-0.626	0.301	0.176	-0.419	-0.595
	M-C	0.152<br>0.152	0.162<br>0.162	-0.184<br>-0.184	-0.173<br>-0.173	0.519<br>0.519	0.12<br>0.12	-0.204<br>-0.204	-0.085<br>-0.085

Table S7. Property-derived descriptors at the bond critical points of some selected bonds including the ellipticity ε, covalency index η(r), the kinetic energy density ratio (G(r)/ρ(r)), and the total energy density ratio (H(r)/ρ_b(r); H(r) = G(r) + V(r)). For M-C bonds two values represent the two individual M-C (3,-1) critical points for *mer*-[MX_3(κ^4-*As*,*CC*,*As*-L^{iPr})] (M = Tc, Re) (X_3 = NCl_2, NBr_2, N(CN)Cl for M = Tc and Cl_3, NCl_2 for M = Re).

core	bond	$\varepsilon = \lambda_1/\lambda_2-1$	η(r) = $\lvert\lambda_1\rvert/\lambda_3$	H(r)/ρ_b(r)	G(r)/ρ_b(r)
{$Tc^V NBr_2$}	C≡C	0.106	2.855	0.575	-1.315
	M-C	0.593<br>0.626	0.339<br>0.337	0.735<br>0.742	-0.323<br>-0.321
{$Tc^V NCl_2$}	C≡C	0.103	2.849	0.578	-1.315
	M-C	0.579<br>0.612	0.34<br>0.338	0.73<br>0.737	-0.32<br>-0.318
{Tc^VN(CN)Cl}	C≡C	0.091	2.875	0.588	-1.322
	M-C	0.643<br>0.718	0.323<br>0.32	0.75<br>0.762	-0.295<br>-0.291
{$Tc^{III}Cl_3$}_ls	C≡C	0.074	2.705	0.565	-1.297
	M-C	0.283<br>0.284	0.315<br>0.315	0.756<br>0.756	-0.354<br>-0.354
{$Re^V NCl_2$}	C≡C	0.101	2.640	0.560	-1.283
	M-C	0.404<br>0.404	0.39<br>0.39	0.673<br>0.673	-0.428<br>-0.428
{$Re^V Cl_3$}_s	C≡C	0.030	2.144	0.502	-1.695
	M-C	0.062<br>0.062	0.354<br>0.354	0.79<br>0.79	-0.559<br>-0.559

Table S8. Oxidation states calculated by localized orbital bonding analysis (LOBA) in *mer*-[MX_3(κ^4-*As*,*CC*,*As*-L^{iPr})] (M = Tc, Re) (X_3 = NCl_2, NBr_2, N(CN)Cl for M = Tc and Cl_3, NCl_2 for M = Re).

core	M central ion	C≡C carbon atoms	MX_3-core fragment	L fragment
{$Tc^V NBr_2$}	3	0, -2	0	0
{$Tc^V NCl_2$}	3	0, -2	0	0
{Tc^VN(CN)Cl}	3	0, 0	0	0
{$Tc^{III}Cl_3$}_ls	3	1, -2	0	0
{$Re^V NCl_2$}	3	0, 0	0	0
{$Re^V Cl_3$}_s	5	-2, -2	2	-2

Table S9. Shape-measures for *mer*-[$ReCl_3$(κ^4-*As*,*CC*,*As*-L^{iPr})].

	**HP-6**	**PPY-6**	**OC-6**	**TPR-6**	**JPPY-6**		
CN 6	D6h: Hexagon	C5v: Pentagonal pyramid	Oh: Octahedron	D3h: Trigonal prism	C5v: Johnson pentagonal pyramid J2		
	33.586	28.447	0.654	16.99	31.688		
	**HP-7**	**HPY-7**	**PBPY-7**	**COC-7**	**CTPR-7**	**JPBPY-7**	**JETPY-7**
CN 7	D7h: Heptagon	C6v: Hexagonal pyramid	D5h: Pentagonal bipyramid	C3v: Capped octahedron	C2v: Capped trigonal prism	D5h: Johnson pentagonal bipyramid J13	C3v: Johnson elongated triangular pyramidJ7
	34.988	24.096	4.953	9.178	7.209	9.578	24.552

S4.2 *mer*-[$Tc^{V}NBr_2$(κ^4-*As*,*CC*,*As*-L^{iPr})].

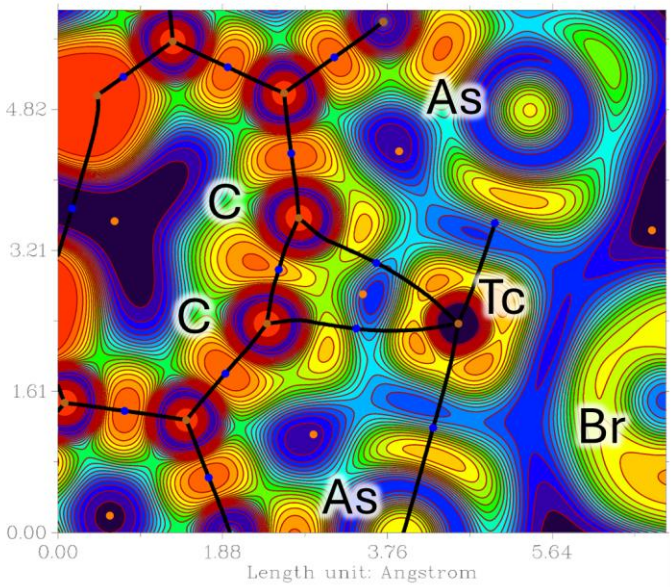

Figure S73. Electron localization function (ELF) map with topological features of ρ(r) ((3,-3) critical points: brown, (3,-1) critical points: blue, (3,+1) critical points: orange, bond paths between (3,-3)/(3,-1) critical points: black) for *mer*-[$Tc^{V}NBr_2$(κ^4-*As*,*CC*,*As*-L^{iPr})] cut through the M···C≡C plane.

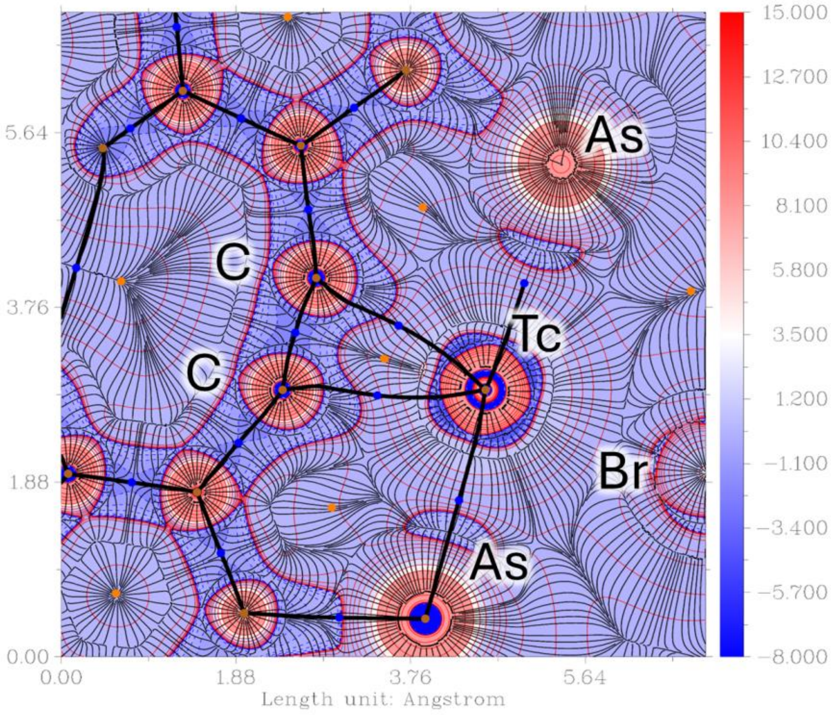

Figure S74. Laplacian of the electron density, $\nabla^2\rho(r)$, map with topological features of ρ(r) ((3,-3) critical points: brown, (3,-1) critical points: blue, (3,+1) critical points: orange, bond paths between (3,-3)/(3,-1) critical points: black) for *mer*-[$Tc^{V}NBr_2$(κ^4-*As*,*CC*,*As*-L^{iPr})] cut through the M···C≡C plane.

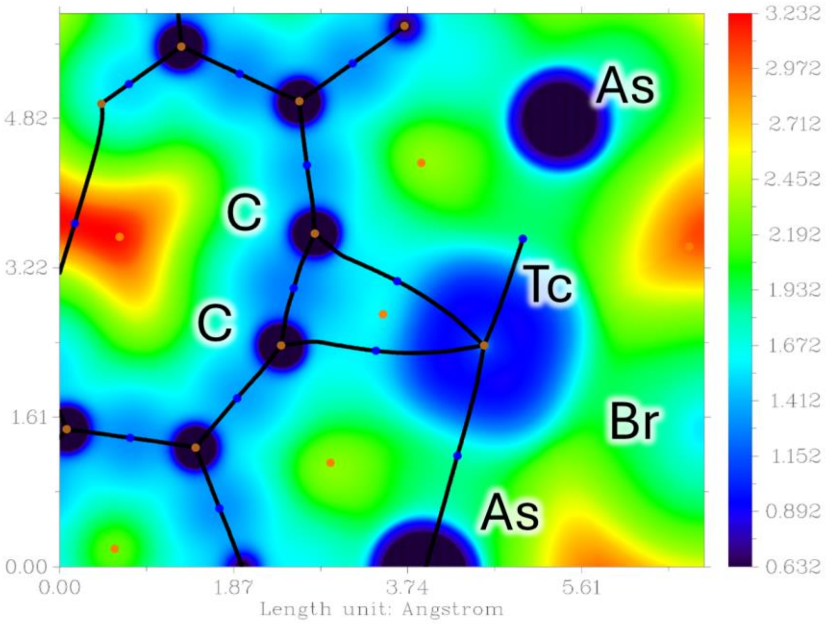

Figure S75. Electron delocalization range function (EDR) map with topological features of ρ(r) ((3,-3) critical points: brown, (3,-1) critical points: blue, (3,+1) critical points: orange, bond paths between (3,-3)/(3,-1) critical points: black) for *mer*-[$Tc^{V}NBr_2$(κ^4-*As*,*CC*,*As*-L^{iPr})] cut through the M···C≡C plane.

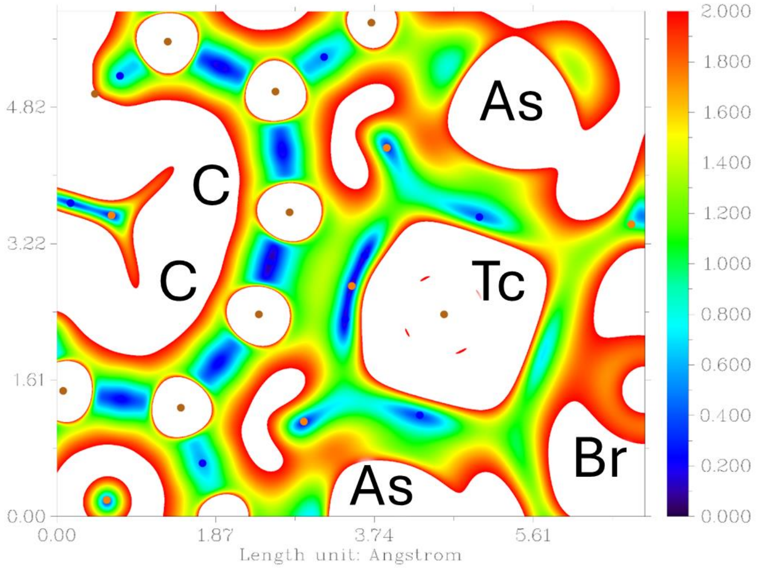

Figure S76. Interaction region indicator (IRI) map with topological features of ρ(r) ((3,-3) critical points: brown, (3,-1) critical points: blue, (3,+1) critical points: orange) for *mer*-[$Tc^{V}NBr_2$(κ^4-*As*,*CC*,*As*-L^{iPr})] cut through the M···C≡C plane.

S4.3 *mer*-[$Tc^{V}NCl_2$(κ^4-*As*,*CC*,*As*-L^{iPr})].

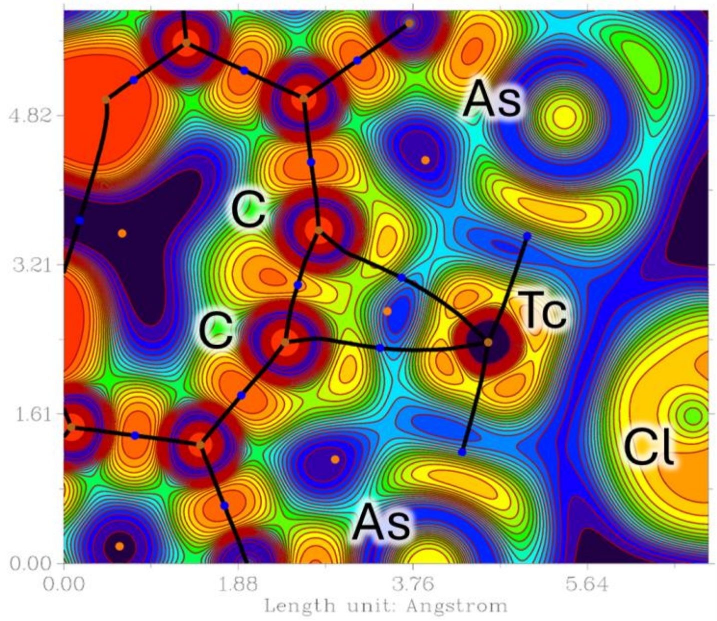

Figure S77. Electron localization function (ELF) map with topological features of ρ(r) ((3,-3) critical points: brown, (3,-1) critical points: blue, (3,+1) critical points: orange, bond paths between (3,-3)/(3,-1) critical points: black) for *mer*-[$Tc^{V}NCl_2$(κ^4-*As*,*CC*,*As*-L^{iPr})] cut through the M···C≡C plane.

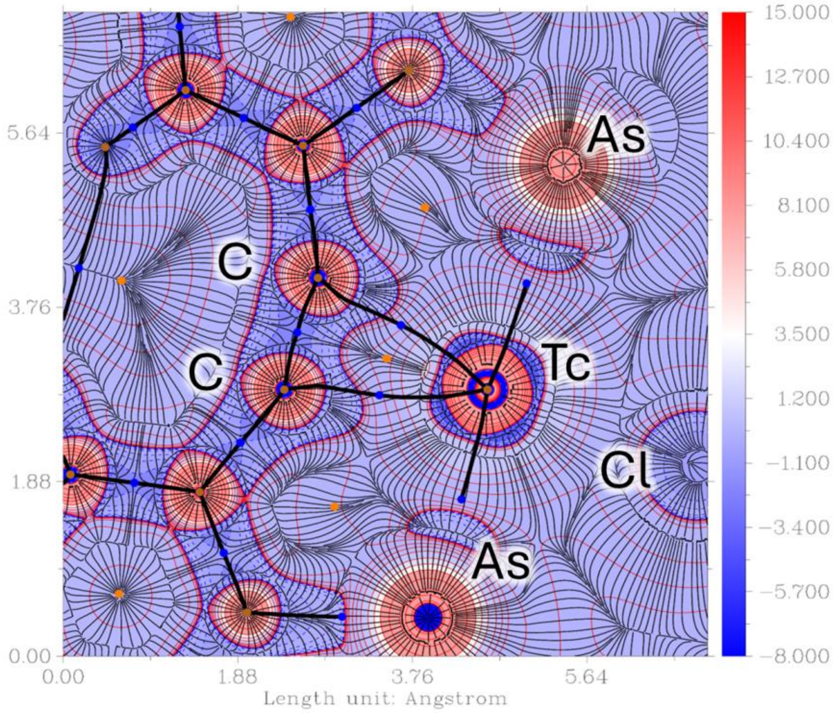

Figure S78. Laplacian of the electron density, $\nabla^2\rho(r)$, map with topological features of ρ(r) ((3,-3) critical points: brown, (3,-1) critical points: blue, (3,+1) critical points: orange, bond paths between (3,-3)/(3,-1) critical points: black) for *mer*-[$Tc^{V}NCl_2$(κ^4-*As*,*CC*,*As*-L^{iPr})] cut through the M···C≡C plane.

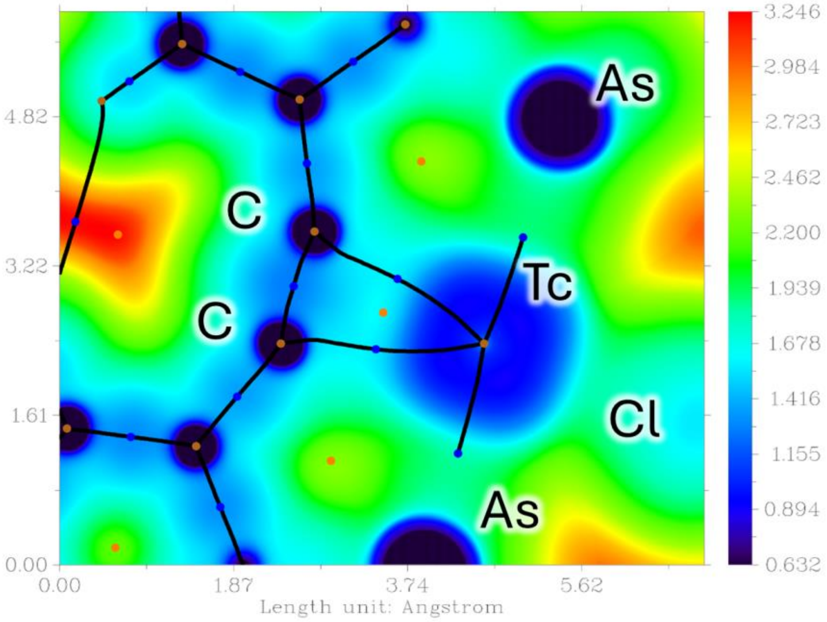

Figure S79. Electron delocalization range function (EDR) map with topological features of ρ(r) ((3,-3) critical points: brown, (3,-1) critical points: blue, (3,+1) critical points: orange, bond paths between (3,-3)/(3,-1) critical points: black) for *mer*-[$Tc^{V}NCl_2$(κ^4-*As*,*CC*,*As*-L^{iPr})] cut through the M···C≡C plane.

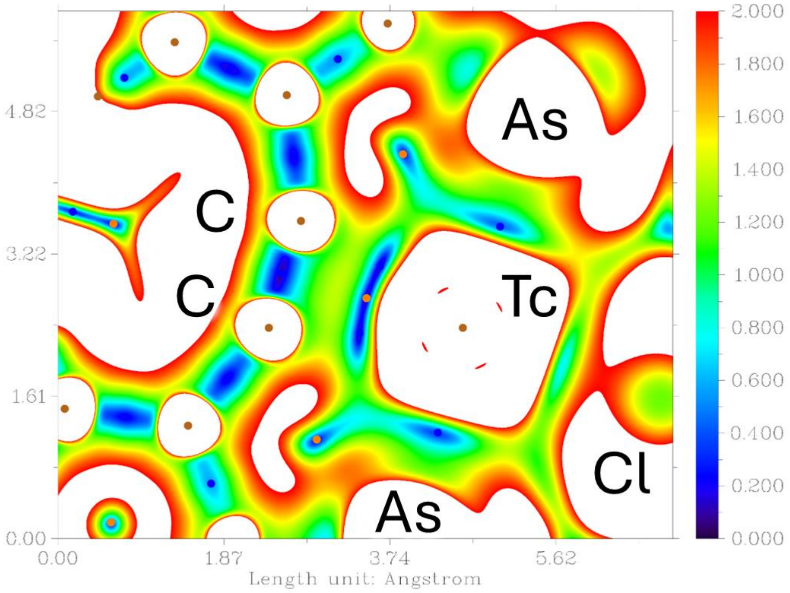

Figure S80. Interaction region indicator (IRI) map with topological features of ρ(r) ((3,-3) critical points: brown, (3,-1) critical points: blue, (3,+1) critical points: orange) for *mer*-[$Tc^{V}NCl_2$(κ^4-*As*,*CC*,*As*-L^{iPr})] cut through the M···C≡C plane.

S4.4 *cis,trans,mer*-[Tc^{V}N(CN)Cl(κ^{4}-*As*,*CC*,*As*-L^{iPr})].

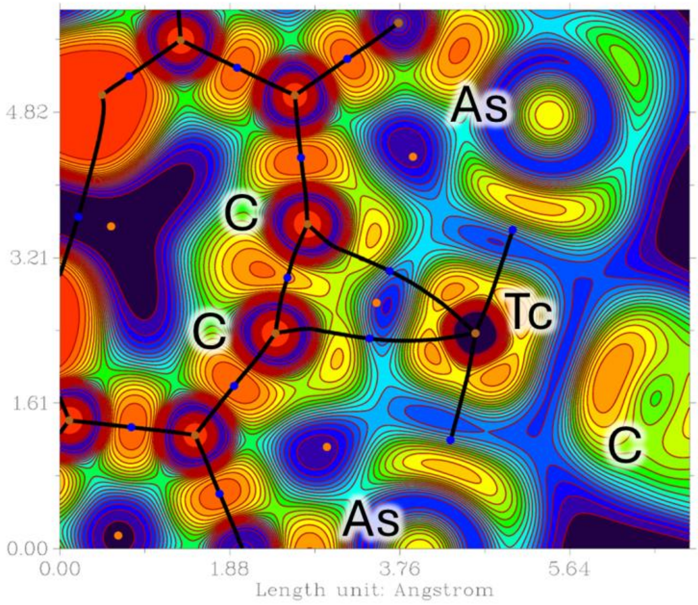

Figure S81. Electron localization function (ELF) map with topological features of ρ(r) ((3,-3) critical points: brown, (3,-1) critical points: blue, (3,+1) critical points: orange, bond paths between (3,-3)/(3,-1) critical points: black) for *cis,trans,mer*-[Tc^{V}N(CN)Cl(κ^{4}-*As*,*CC*,*As*-L^{iPr})] cut through the M···C≡C plane.

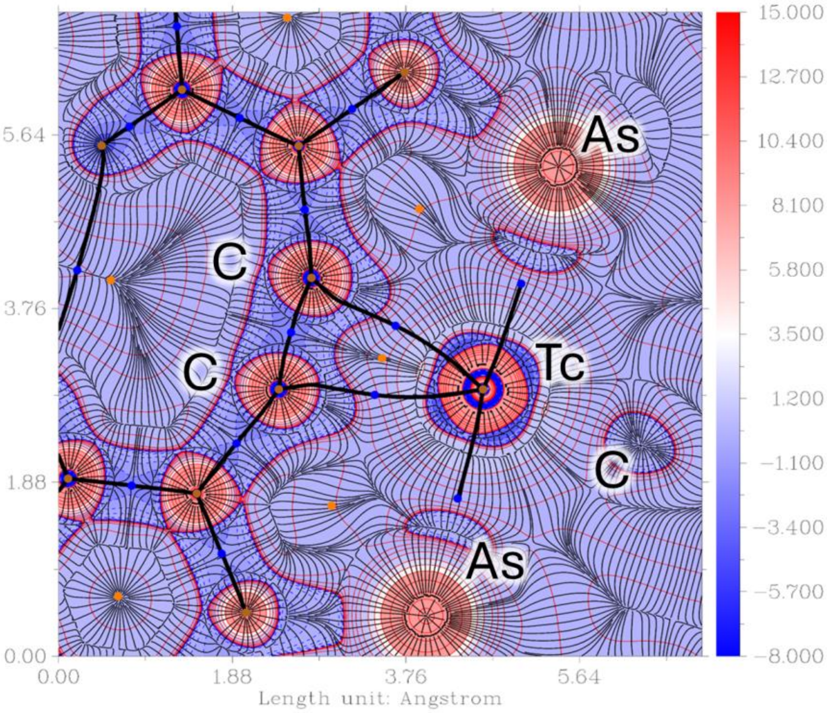

Figure S82. Laplacian of the electron density, $\nabla^2\rho(r)$, map with topological features of ρ(r) ((3,-3) critical points: brown, (3,-1) critical points: blue, (3,+1) critical points: orange, bond paths between (3,-3)/(3,-1) critical points: black) for *cis,trans,mer*-[Tc^{V}N(CN)Cl(κ^{4}-*As*,*CC*,*As*-L^{iPr})] cut through the M···C≡C plane.

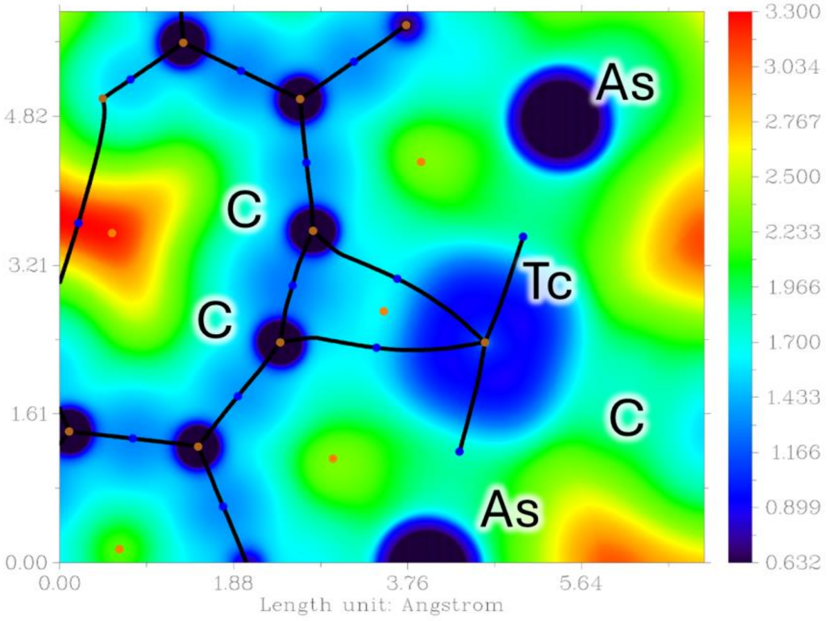

Figure S83. Electron delocalization range function (EDR) map with topological features of ρ(r) ((3,-3) critical points: brown, (3,-1) critical points: blue, (3,+1) critical points: orange, bond paths between (3,-3)/(3,-1) critical points: black) for *cis,trans,mer*-[Tc^{V}N(CN)Cl(κ^4-*As*,*CC*,*As*-L^{iPr})] cut through the M···C≡C plane.

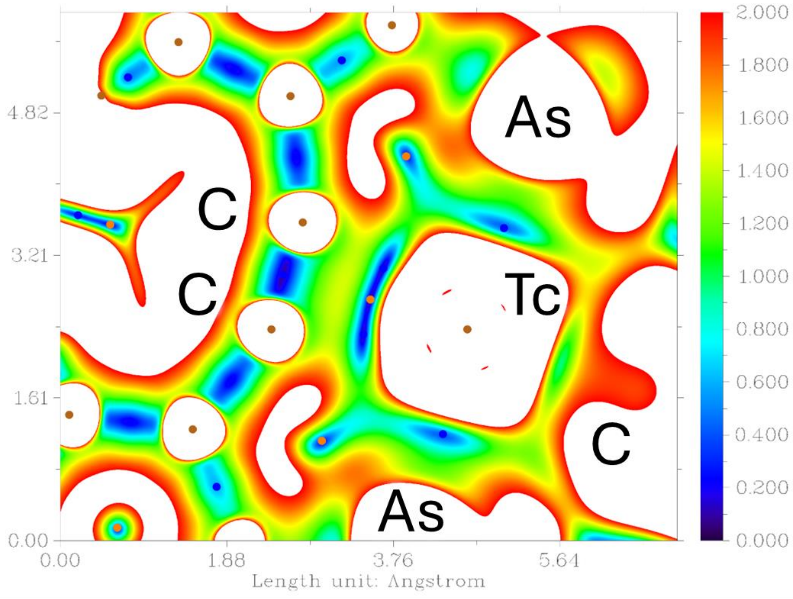

Figure S84. Interaction region indicator (IRI) map with topological features of ρ(r) ((3,-3) critical points: brown, (3,-1) critical points: blue, (3,+1) critical points: orange) for *cis,trans,mer*-[Tc^{V}N(CN)Cl(κ^4-*As*,*CC*,*As*-L^{iPr})] cut through the M···C≡C plane.

S4.5 *mer*-[Tc^{III}Cl$_3$(κ^4-*As*,*CC*,*As*-L^{iPr})]: singlet/d^2

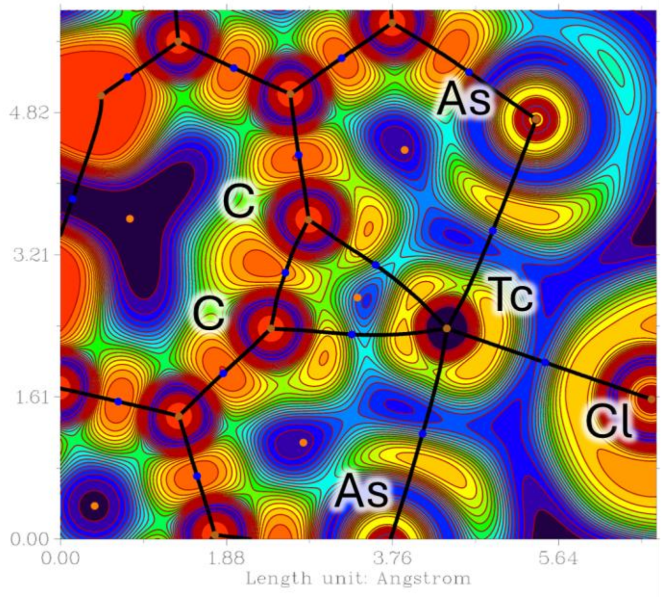

Figure S85. Electron localization function (ELF) map with topological features of ρ(r) ((3,-3) critical points: brown, (3,-1) critical points: blue, (3,+1) critical points: orange, bond paths between (3,-3)/(3,-1) critical points: black) for *mer*-[Tc^{III}Cl$_3$(κ^4-*As*,*CC*,*As*-L^{iPr})] cut through the M···C≡C plane.

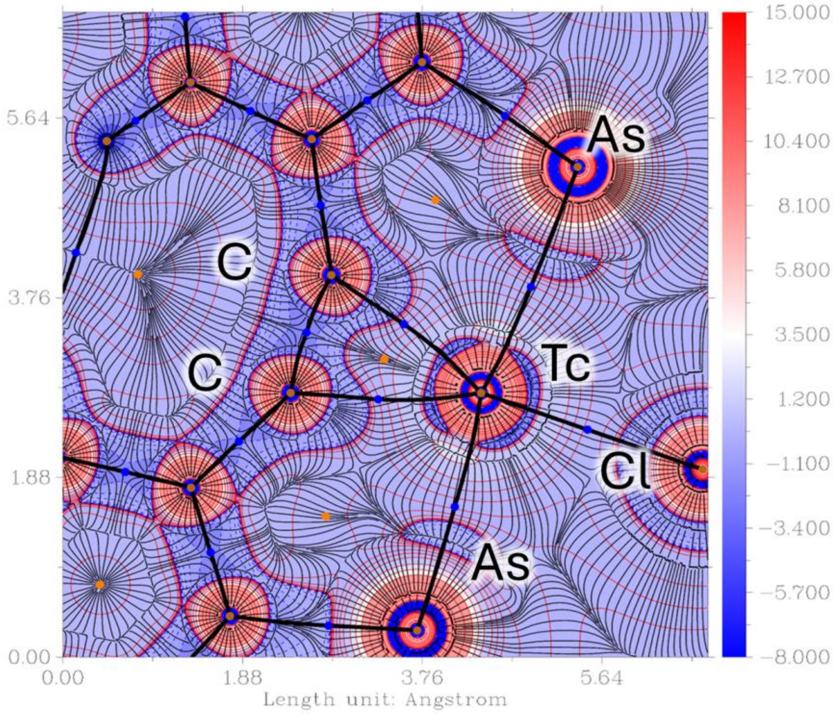

Figure S86. Laplacian of the electron density, $\nabla^2\rho(r)$, map with topological features of ρ(r) ((3,-3) critical points: brown, (3,-1) critical points: blue, (3,+1) critical points: orange, bond paths between (3,-3)/(3,-1) critical points: black) for *mer*-[Tc^{III}Cl$_3$(κ^4-*As*,*CC*,*As*-L^{iPr})] cut through the M···C≡C plane.

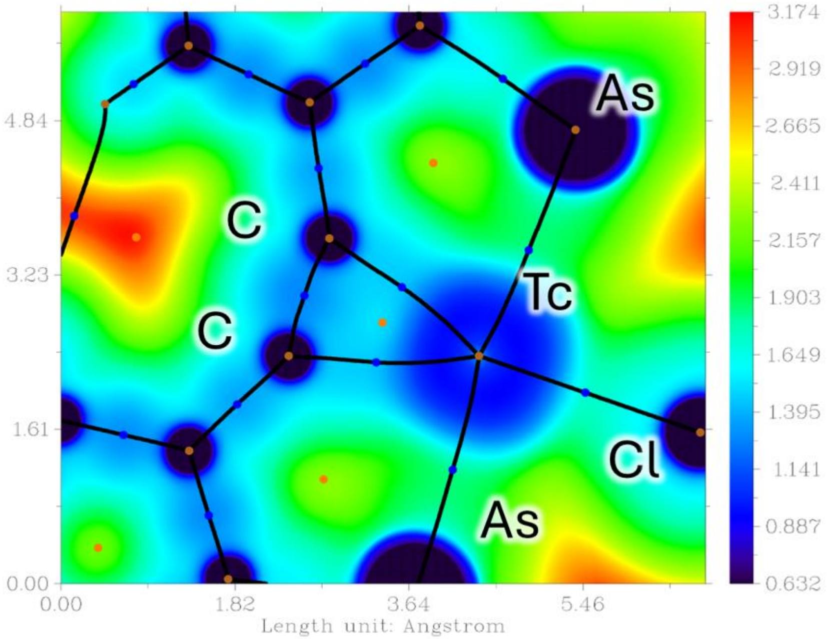

Figure S87. Electron delocalization range function (EDR) map with topological features of ρ(r) ((3,-3) critical points: brown, (3,-1) critical points: blue, (3,+1) critical points: orange, bond paths between (3,-3)/(3,-1) critical points: black) for *mer*-[$Tc^{III}Cl_3$(κ^4-*As*,*CC*,*As*-L^{iPr})] cut through the M···C≡C plane.

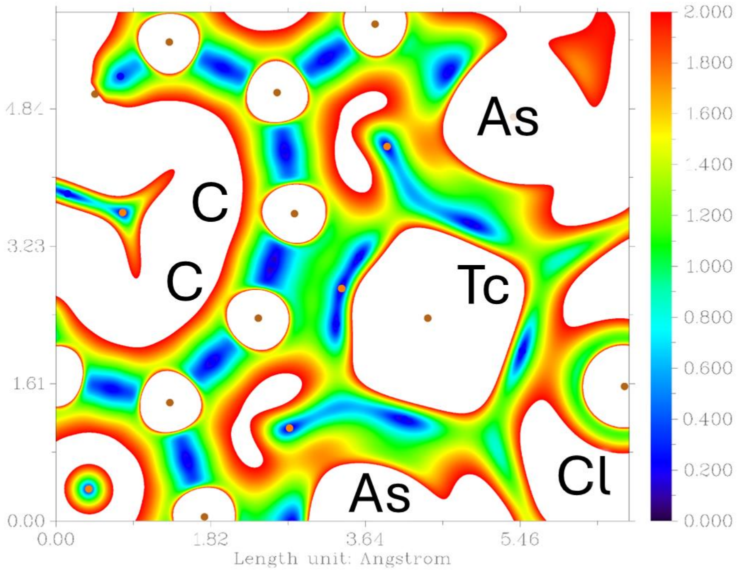

Figure S88. Interaction region indicator (IRI) map with topological features of ρ(r) ((3,-3) critical points: brown, (3,-1) critical points: blue, (3,+1) critical points: orange) for *mer*-[$Tc^{III}Cl_3$(κ^4-*As*,*CC*,*As*-L^{iPr})] cut through the M···C≡C plane.

S4.6 *mer*-[$Tc^{III}Cl_3$(κ^4-*As*,*CC*,*As*-L^{iPr})]: triplet/d^4-ls; energetically preferred

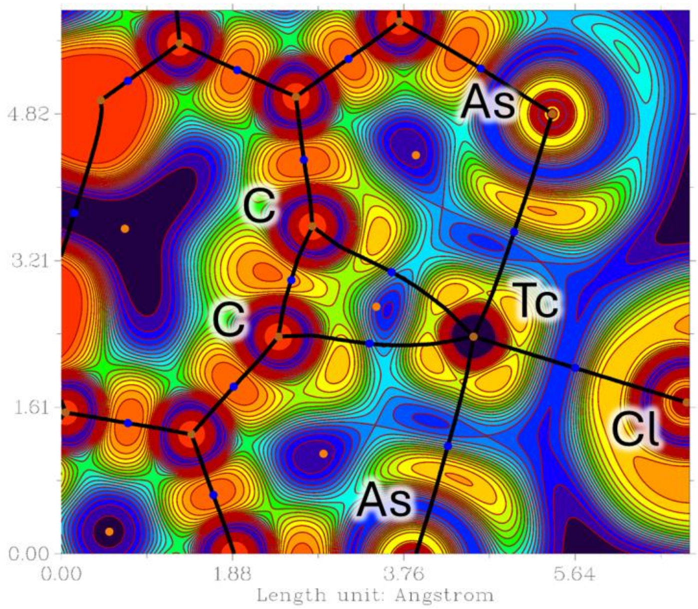

Figure S89. Electron localization function (ELF) map with topological features of ρ(r) ((3,-3) critical points: brown, (3,-1) critical points: blue, (3,+1) critical points: orange, bond paths between (3,-3)/(3,-1) critical points: black) for *mer*-[$Tc^{III}Cl_3$(κ^4-*As*,*CC*,*As*-L^{iPr})] cut through the M⋯C≡C plane.

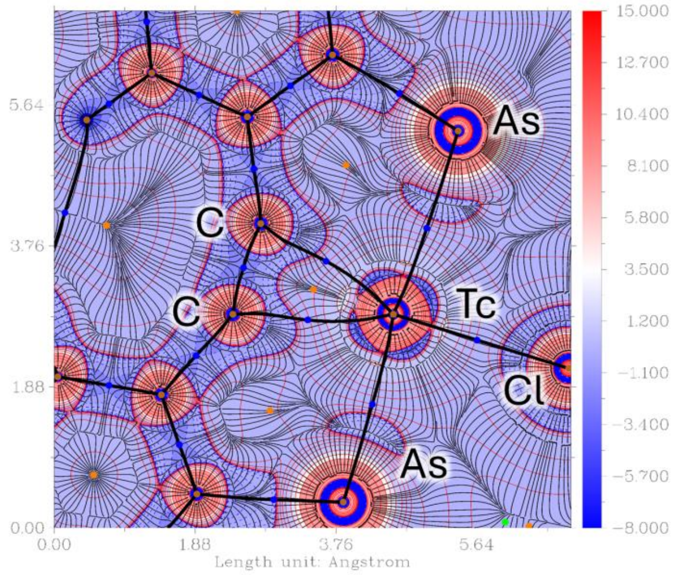

Figure S90. Laplacian of the electron density, $\nabla^2\rho(r)$, map with topological features of ρ(r) ((3,-3) critical points: brown, (3,-1) critical points: blue, (3,+1) critical points: orange, bond paths between (3,-3)/(3,-1) critical points: black) for *mer*-[$Tc^{III}Cl_3$(κ^4-*As*,*CC*,*As*-L^{iPr})] cut through the M⋯C≡C plane.

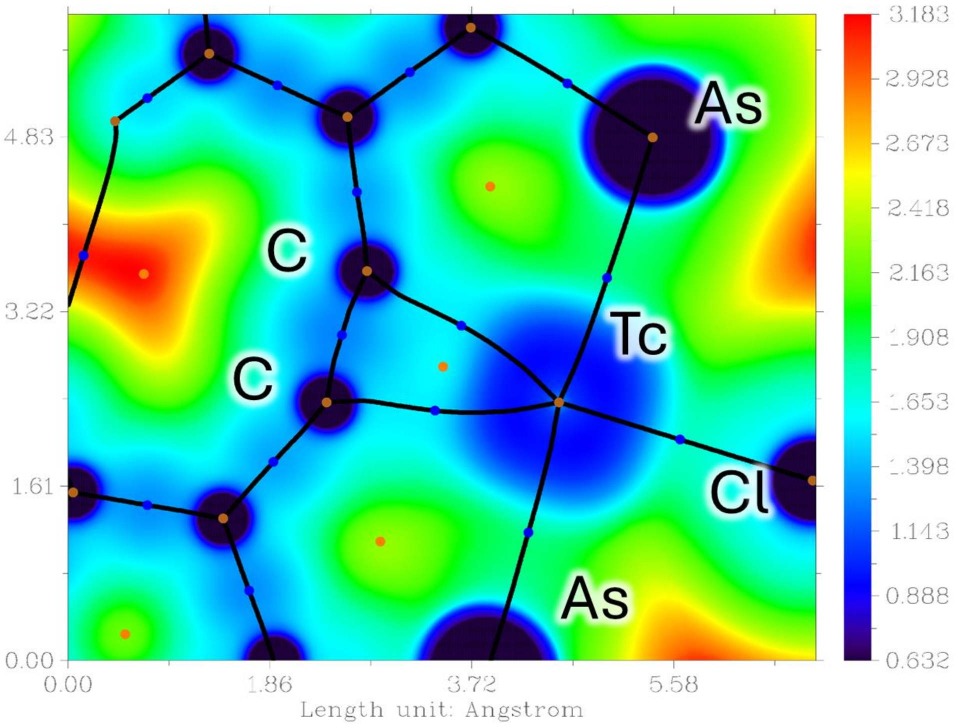

Figure S91. Electron delocalization range function (EDR) map with topological features of ρ(r) ((3,-3) critical points: brown, (3,-1) critical points: blue, (3,+1) critical points: orange, bond paths between (3,-3)/(3,-1) critical points: black) for *mer*-[$Tc^{III}Cl_3$(κ^4-*As*,*CC*,*As*-L^{iPr})] cut through the M···C≡C plane.

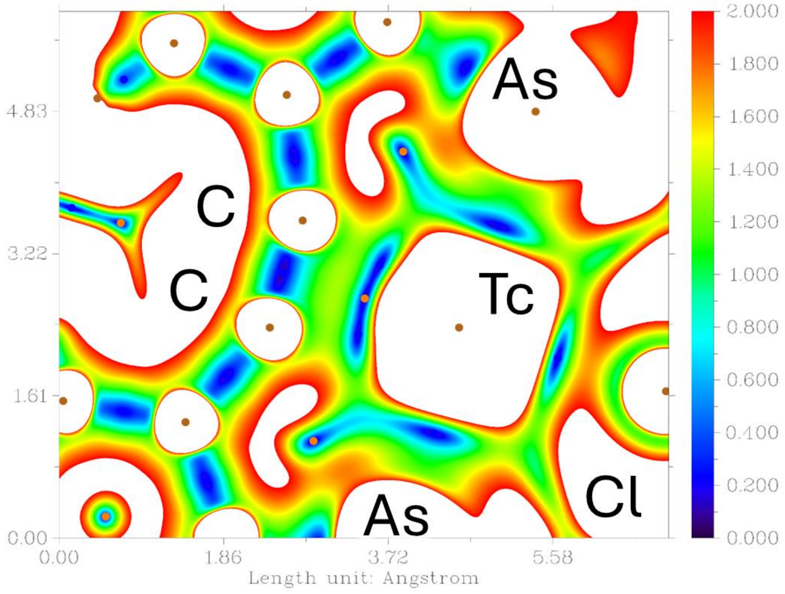

Figure S92. Interaction region indicator (IRI) map with topological features of ρ(r) ((3,-3) critical points: brown, (3,-1) critical points: blue, (3,+1) critical points: orange) for *mer*-[$Tc^{III}Cl_3$(κ^4-*As*,*CC*,*As*-L^{iPr})] cut through the M···C≡C plane.

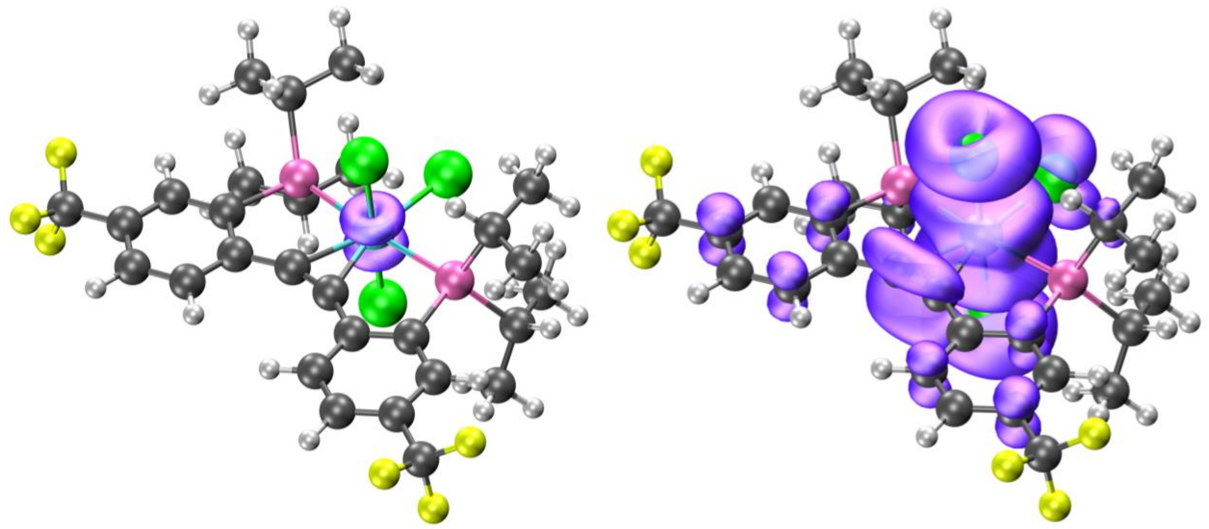

Figure S93. Spin density (left: isovalue of 0.04; right: isovalue of 0.0004) for *mer*-[$Tc^{III}Cl_3$(κ^4-*As*,*CC*,*As*-L^{iPr})].

S4.7 *mer*-[Tc^{III}Cl$_3$(κ^4-*As*,*CC*,*As*-L^{iPr})]: quintet/d^4-hs

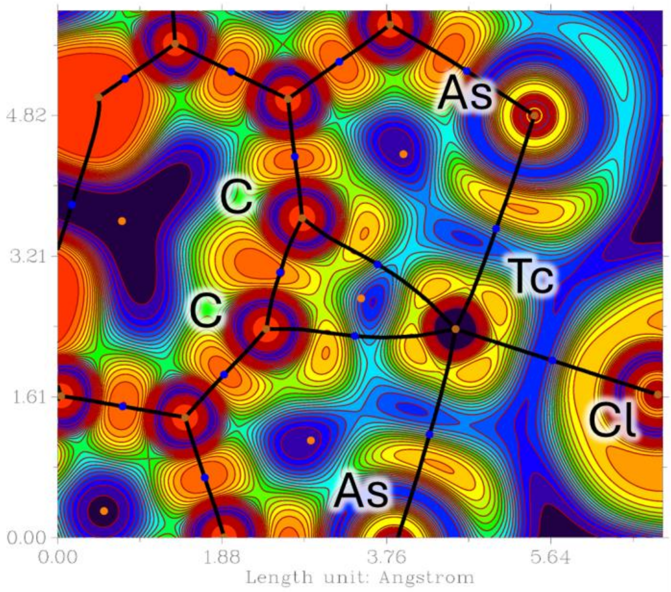

Figure S94. Electron localization function (ELF) map with topological features of ρ(r) ((3,-3) critical points: brown, (3,-1) critical points: blue, (3,+1) critical points: orange, bond paths between (3,-3)/(3,-1) critical points: black) for *mer*-[Tc^{III}Cl$_3$(κ^4-*As*,*CC*,*As*-L^{iPr})] cut through the M···C≡C plane.

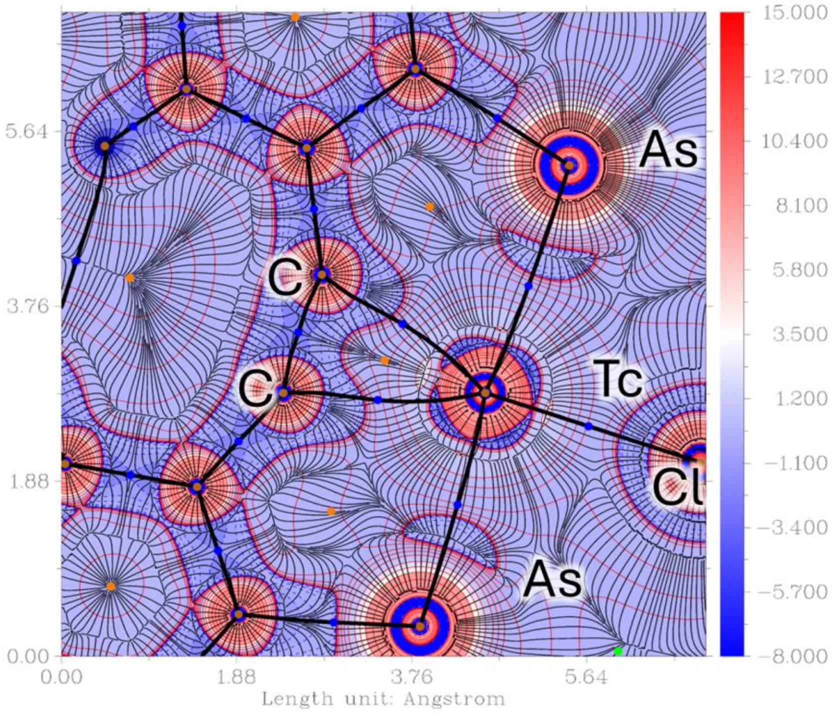

Figure S95. Laplacian of the electron density, $\nabla^2\rho(r)$, map with topological features of ρ(r) ((3,-3) critical points: brown, (3,-1) critical points: blue, (3,+1) critical points: orange, bond paths between (3,-3)/(3,-1) critical points: black) for *mer*-[Tc^{III}Cl$_3$(κ^4-*As*,*CC*,*As*-L^{iPr})] cut through the M···C≡C plane.

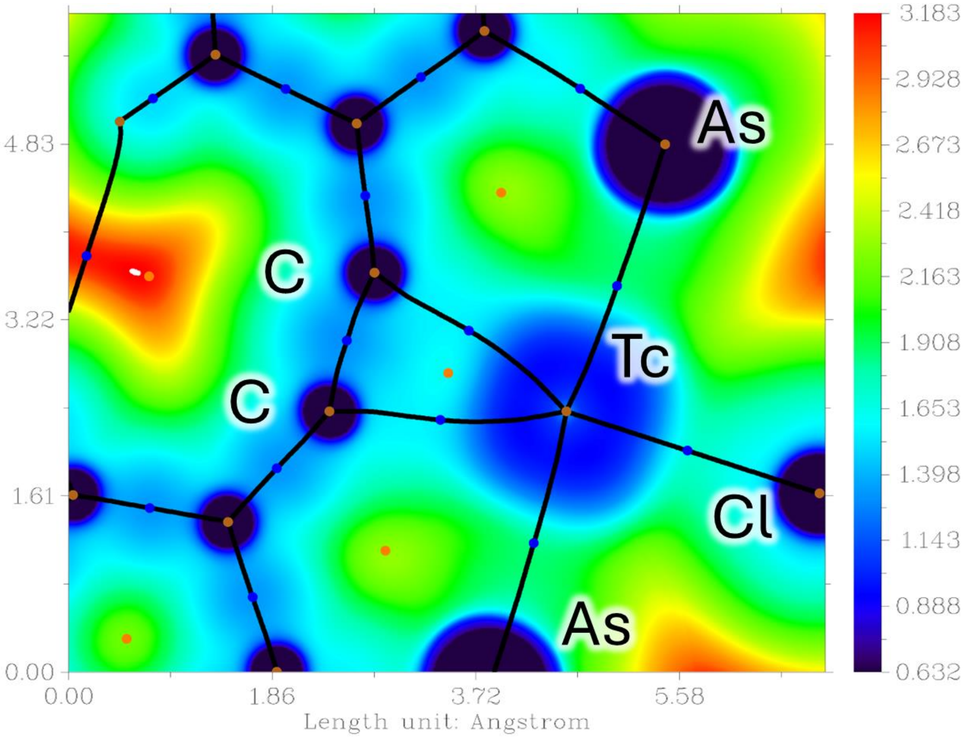

Figure S96. Electron delocalization range function (EDR) map with topological features of ρ(r) ((3,-3) critical points: brown, (3,-1) critical points: blue, (3,+1) critical points: orange, bond paths between (3,-3)/(3,-1) critical points: black) for *mer*-[$Tc^{III}Cl_3$(κ^4-*As*,*CC*,*As*-L^{iPr})] cut through the M···C≡C plane.

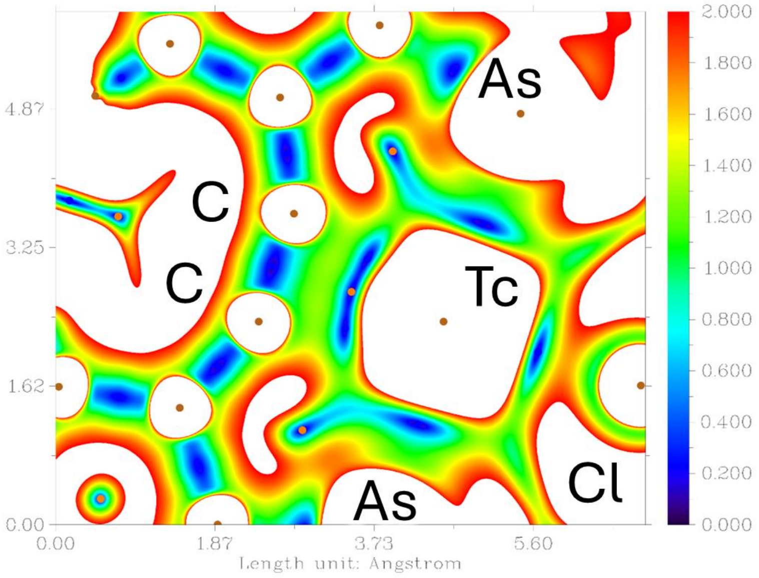

Figure S97. Interaction region indicator (IRI) map with topological features of ρ(r) ((3,-3) critical points: brown, (3,-1) critical points: blue, (3,+1) critical points: orange) for *mer*-[$Tc^{III}Cl_3$(κ^4-*As*,*CC*,*As*-L^{iPr})] cut through the M···C≡C plane.

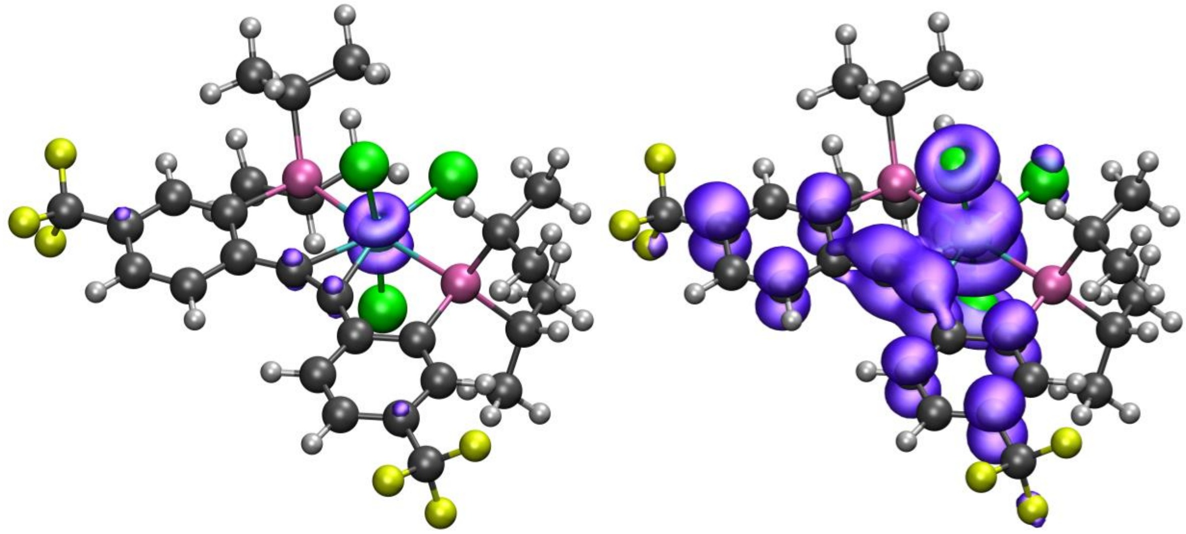

Figure S98. Spin density (left: isovalue of 0.04; right: isovalue of 0.0004) for *mer*-[$Tc^{III}Cl_3$(κ^4-*As*,*CC*,*As*-L^{iPr})].

S4.8 *mer*-[Re^{V}NCl$_2$(κ^4-*As*,*CC*,*As*-L^{iPr})].

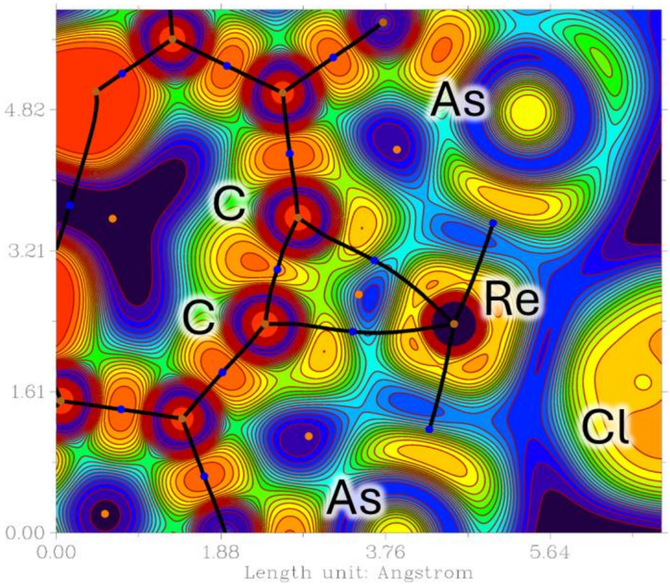

Figure S99. Electron localization function (ELF) map with topological features of ρ(r) ((3,-3) critical points: brown, (3,-1) critical points: blue, (3,+1) critical points: orange, bond paths between (3,-3)/(3,-1) critical points: black) for *mer*-[Re^{V}NCl$_2$(κ^4-*As*,*CC*,*As*-L^{iPr})] cut through the M···C≡C plane.

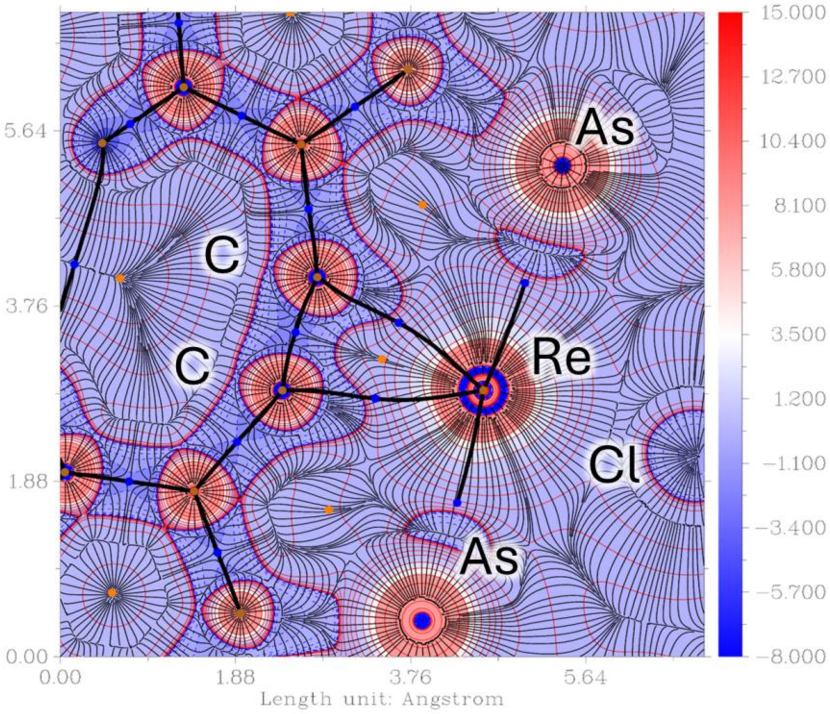

Figure S100. Laplacian of the electron density, $\nabla^2\rho(r)$, map with topological features of ρ(r) ((3,-3) critical points: brown, (3,-1) critical points: blue, (3,+1) critical points: orange, bond paths between (3,-3)/(3,-1) critical points: black) for *mer*-[Re^{V}NCl$_2$(κ^4-*As*,*CC*,*As*-L^{iPr})] cut through the M···C≡C plane.

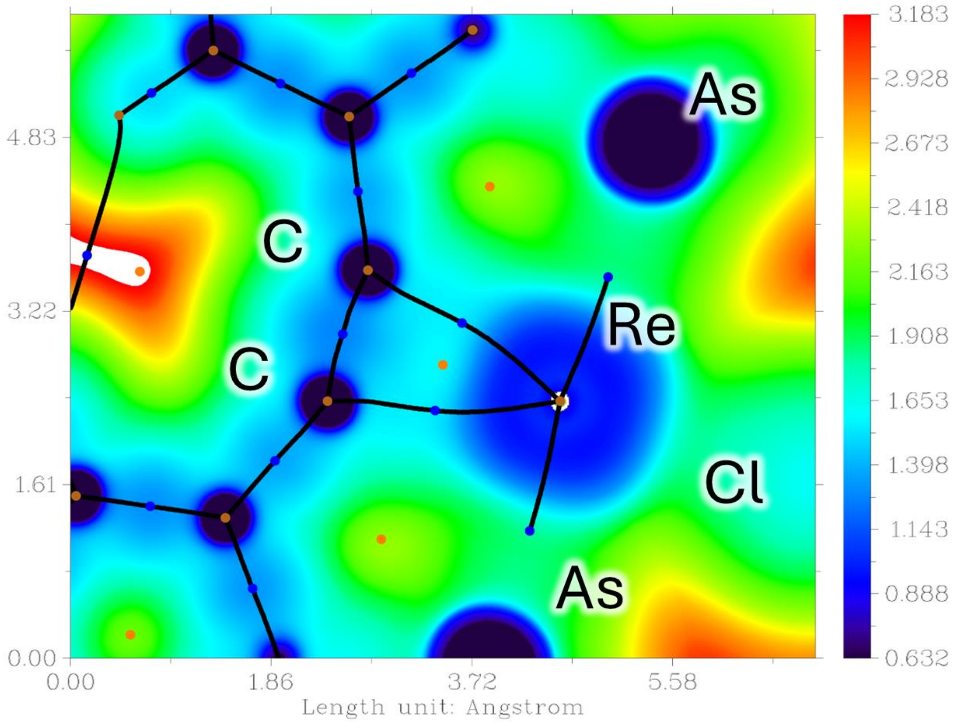

Figure S101. Electron delocalization range function (EDR) map with topological features of ρ(r) ((3,-3) critical points: brown, (3,-1) critical points: blue, (3,+1) critical points: orange, bond paths between (3,-3)/(3,-1) critical points: black) for *mer*-[$Re^{V}NCl_2$(κ^4-*As*,*CC*,*As*-L^{iPr})] cut through the M···C≡C plane.

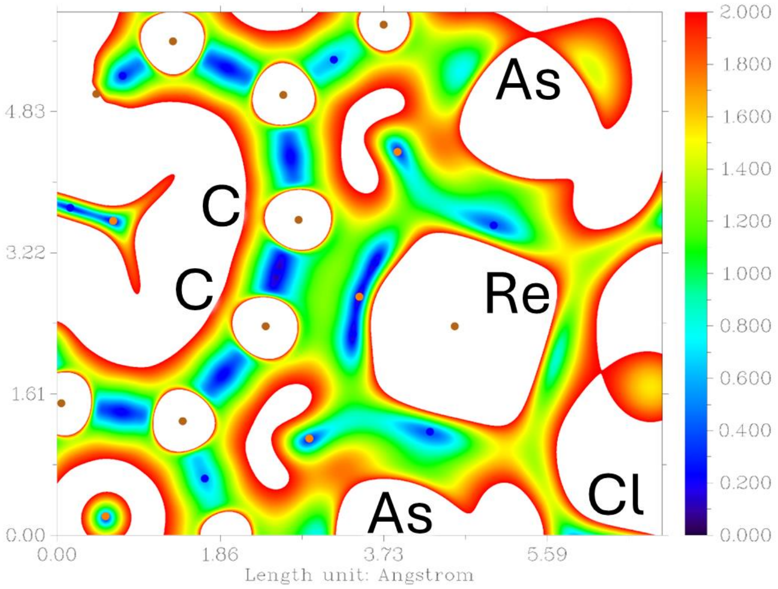

Figure S102. Interaction region indicator (IRI) map with topological features of ρ(r) ((3,-3) critical points: brown, (3,-1) critical points: blue, (3,+1) critical points: orange) for *mer*-[$Re^{V}NCl_2$(κ^4-*As*,*CC*,*As*-L^{iPr})] cut through the M···C≡C plane.

S4.9 *mer*-[$Re^{V}Cl_3$(κ^4-*As*,*CC*,*As*-L^{iPr})]. Singlet/d^2; energetically preferred

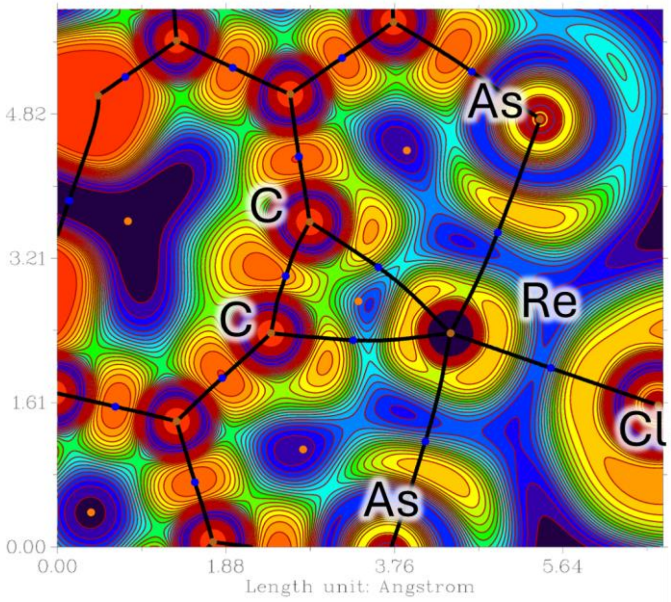

Figure S103. Electron localization function (ELF) map with topological features of ρ(r) ((3,-3) critical points: brown, (3,-1) critical points: blue, (3,+1) critical points: orange, bond paths between (3,-3)/(3,-1) critical points: black) for *mer*-[$Re^{V}Cl_3$(κ^4-*As*,*CC*,*As*-L^{iPr})] cut through the M···C≡C plane.

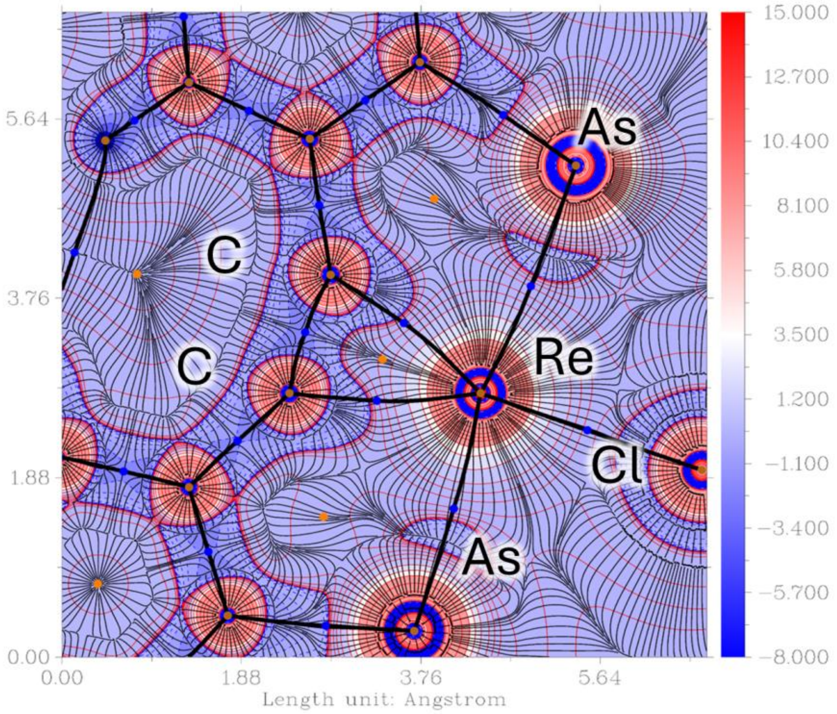

Figure S104. Laplacian of the electron density, $\nabla^2\rho(r)$, map with topological features of ρ(r) ((3,-3) critical points: brown, (3,-1) critical points: blue, (3,+1) critical points: orange, bond paths between (3,-3)/(3,-1) critical points: black) for *mer*-[$Re^{V}Cl_3$(κ^4-*As*,*CC*,*As*-L^{iPr})] cut through the M···C≡C plane.

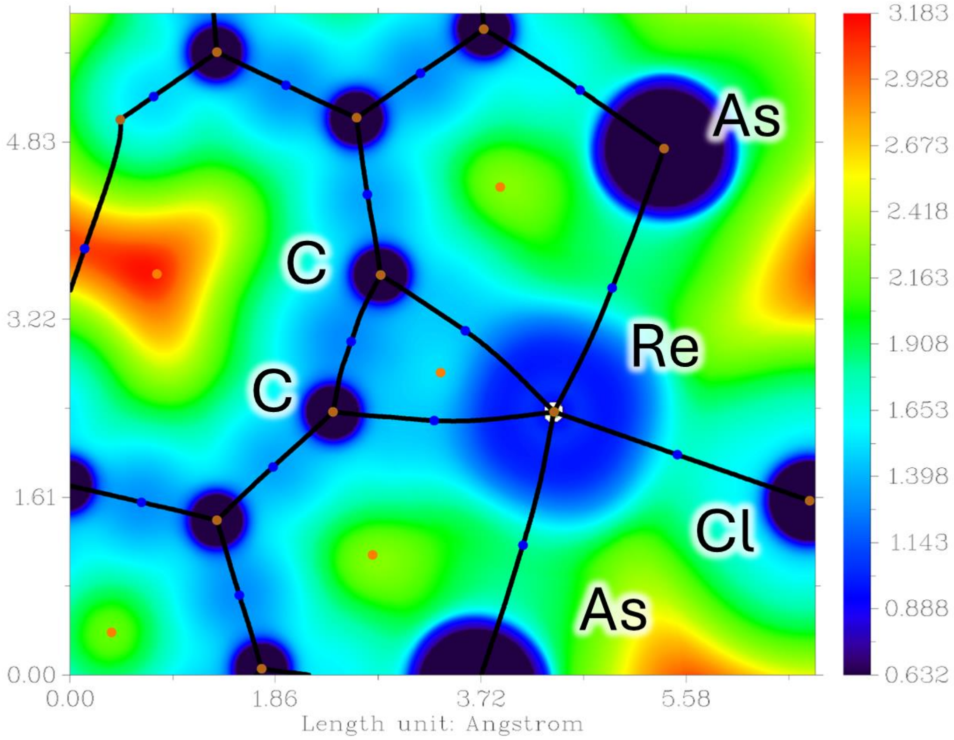

Figure S105. Electron delocalization range function (EDR) map with topological features of ρ(r) ((3,-3) critical points: brown, (3,-1) critical points: blue, (3,+1) critical points: orange, bond paths between (3,-3)/(3,-1) critical points: black) for *mer*-[$Re^{V}Cl_3$($κ^4$-*As*,*CC*,*As*-L^{iPr})] cut through the M⋯C≡C plane.

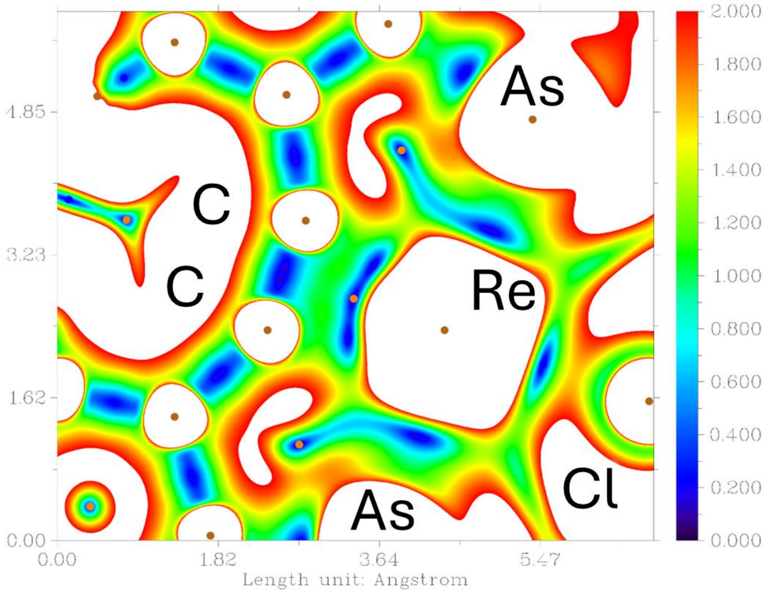

Figure S106. Interaction region indicator (IRI) map with topological features of ρ(r) ((3,-3) critical points: brown, (3,-1) critical points: blue, (3,+1) critical points: orange) for *mer*-[$Re^{V}Cl_3$($κ^4$-*As*,*CC*,*As*-L^{iPr})] cut through the M⋯C≡C plane.

S4.10 *mer*-[$Re^{V}Cl_3$(κ^4-*As*,*CC*,*As*-L^{iPr})]. Triplet/d^4-ls

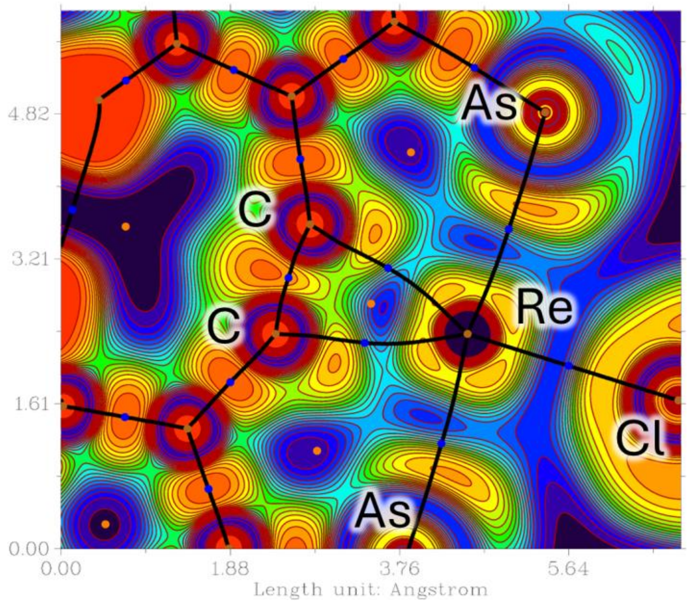

Figure S107. Electron localization function (ELF) map with topological features of ρ(r) ((3,-3) critical points: brown, (3,-1) critical points: blue, (3,+1) critical points: orange, bond paths between (3,-3)/(3,-1) critical points: black) for *mer*-[$Re^{V}Cl_3$(κ^4-*As*,*CC*,*As*-L^{iPr})] cut through the M···C≡C plane.

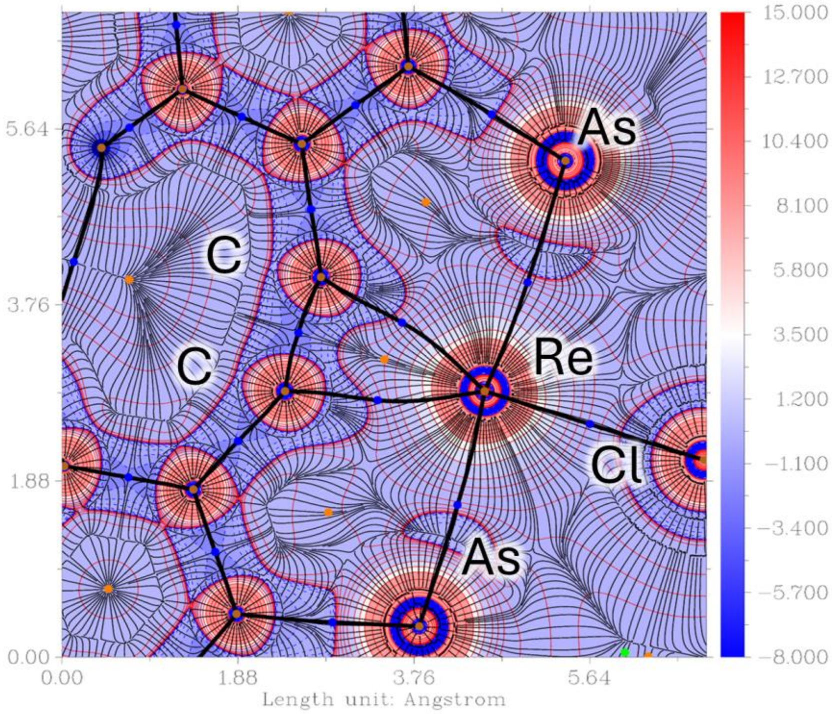

Figure S108. Laplacian of the electron density, $\nabla^2\rho(r)$, map with topological features of ρ(r) ((3,-3) critical points: brown, (3,-1) critical points: blue, (3,+1) critical points: orange, bond paths between (3,-3)/(3,-1) critical points: black) for *mer*-[$Re^{V}Cl_3$(κ^4-*As*,*CC*,*As*-L^{iPr})] cut through the M···C≡C plane.

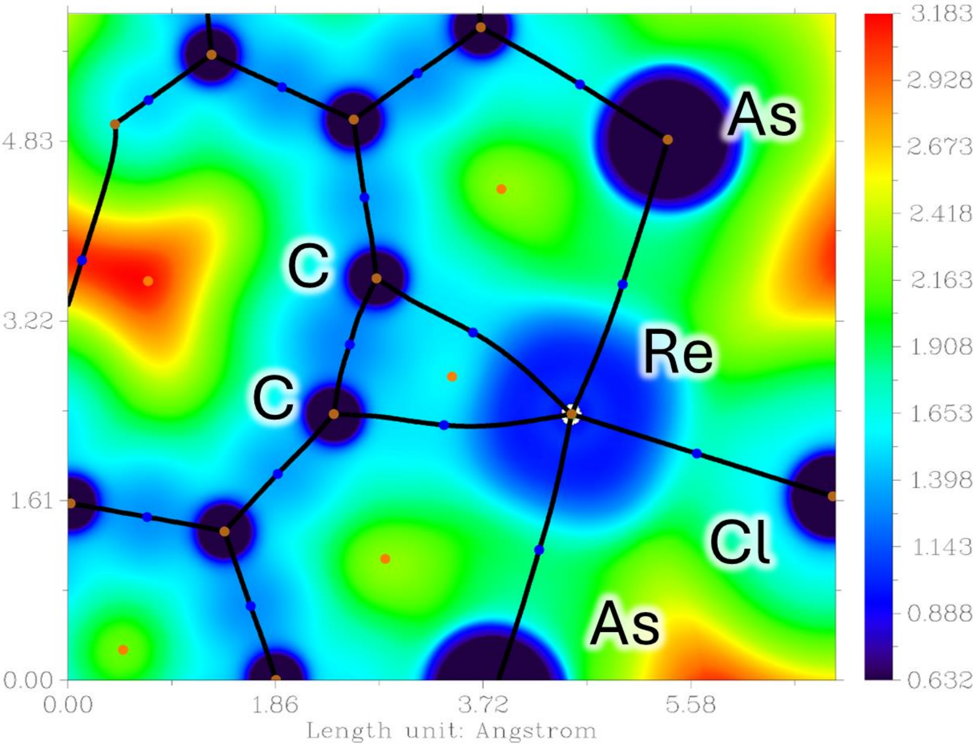

Figure S109. Electron delocalization range function (EDR) map with topological features of ρ(r) ((3,-3) critical points: brown, (3,-1) critical points: blue, (3,+1) critical points: orange, bond paths between (3,-3)/(3,-1) critical points: black) for *mer*-[$Re^{V}Cl_3$(κ[4]-*As*,*CC*,*As*-L[*i*Pr])] cut through the M⋯C≡C plane.

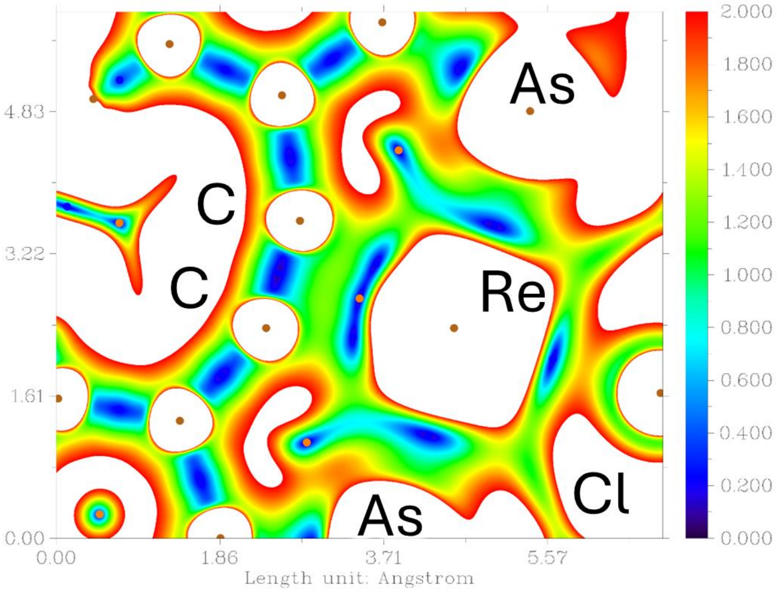

Figure S110. Interaction region indicator (IRI) map with topological features of ρ(r) ((3,-3) critical points: brown, (3,-1) critical points: blue, (3,+1) critical points: orange) for *mer*-[$Re^{V}Cl_3$(κ[4]-*As*,*CC*,*As*-L[*i*Pr])] cut through the M⋯C≡C plane.

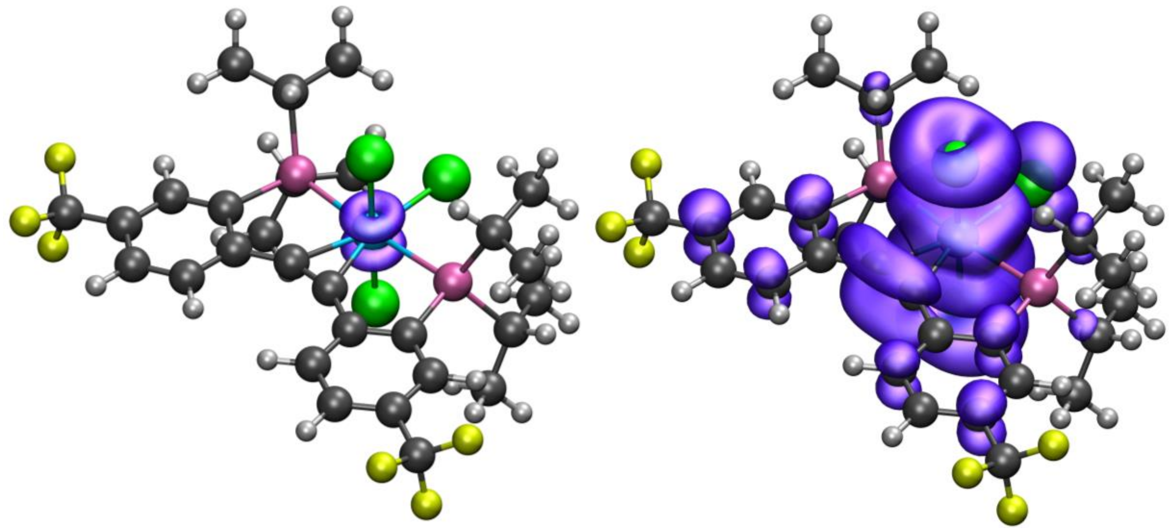

Figure S111. Spin density (left: isovalue of 0.04; right: isovalue of 0.0004) for *mer*-[$Re^{V}Cl_3$(κ^4-*As*,*CC*,*As*-L^{iPr})].

S4.11 *mer*-[$Re^{V}Cl_3$(κ^4-*As*,*CC*,*As*-L^{iPr})]. Triplet/d^4-ls

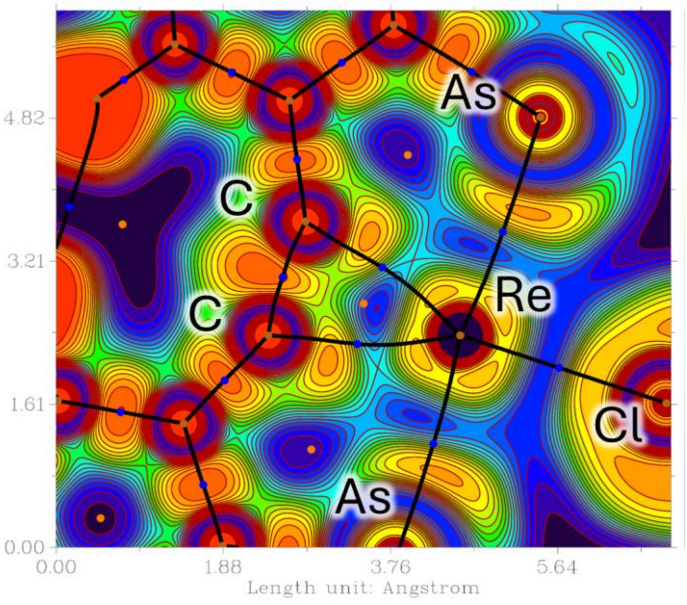

Figure S112. Electron localization function (ELF) map with topological features of ρ(r) ((3,-3) critical points: brown, (3,-1) critical points: blue, (3,+1) critical points: orange, bond paths between (3,-3)/(3,-1) critical points: black) for *mer*-[$Re^{V}Cl_3$(κ^4-*As*,*CC*,*As*-L^{iPr})] cut through the M···C≡C plane.

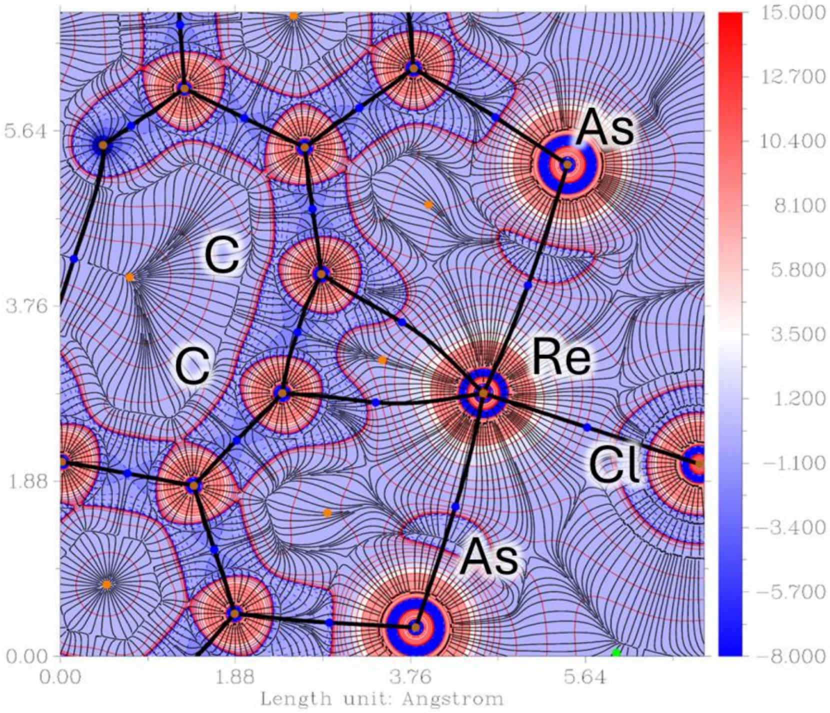

Figure S113. Laplacian of the electron density, $\nabla^2\rho(r)$, map with topological features of ρ(r) ((3,-3) critical points: brown, (3,-1) critical points: blue, (3,+1) critical points: orange, bond paths between (3,-3)/(3,-1) critical points: black) for *mer*-[$Re^{V}Cl_3$(κ^4-*As*,*CC*,*As*-L^{iPr})] cut through the M···C≡C plane.

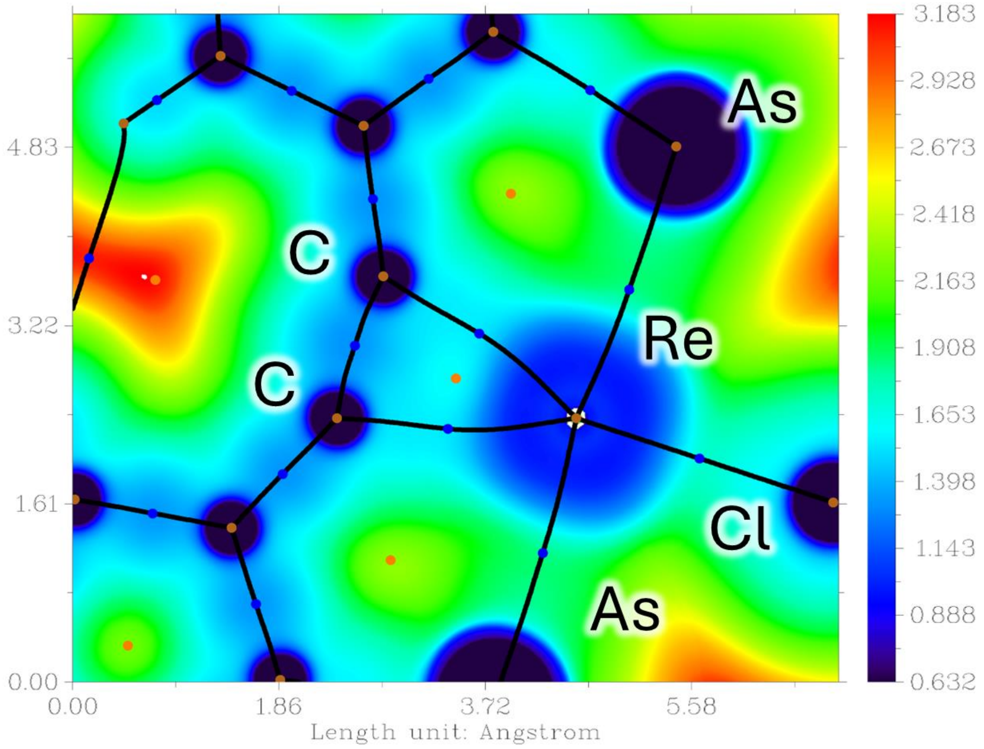

Figure S114. Electron delocalization range function (EDR) map with topological features of ρ(r) ((3,-3) critical points: brown, (3,-1) critical points: blue, (3,+1) critical points: orange, bond paths between (3,-3)/(3,-1) critical points: black) for *mer*-[$Re^{V}Cl_3$(κ^4-*As*,*CC*,*As*-L^{iPr})] cut through the M⋯C≡C plane.

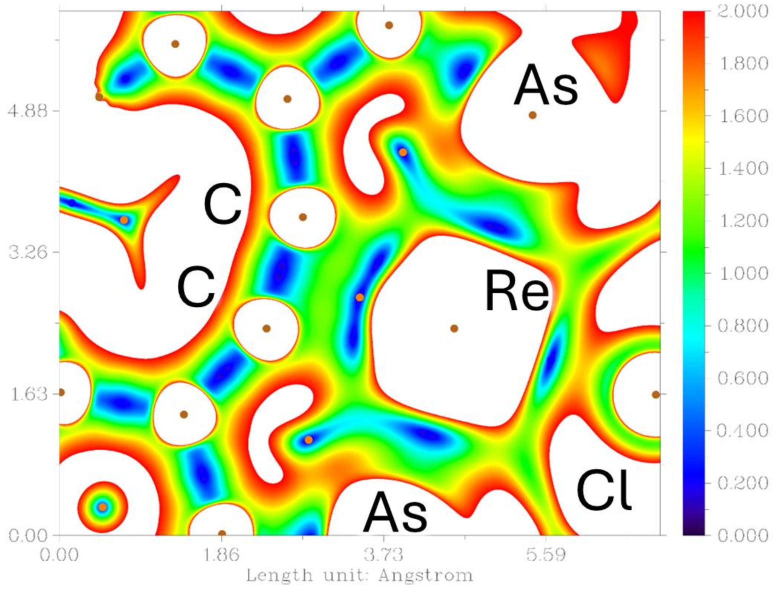

Figure S115. Interaction region indicator (IRI) map with topological features of ρ(r) ((3,-3) critical points: brown, (3,-1) critical points: blue, (3,+1) critical points: orange) for *mer*-[$Re^{V}Cl_3$(κ^4-*As*,*CC*,*As*-L^{iPr})] cut through the M⋯C≡C plane.

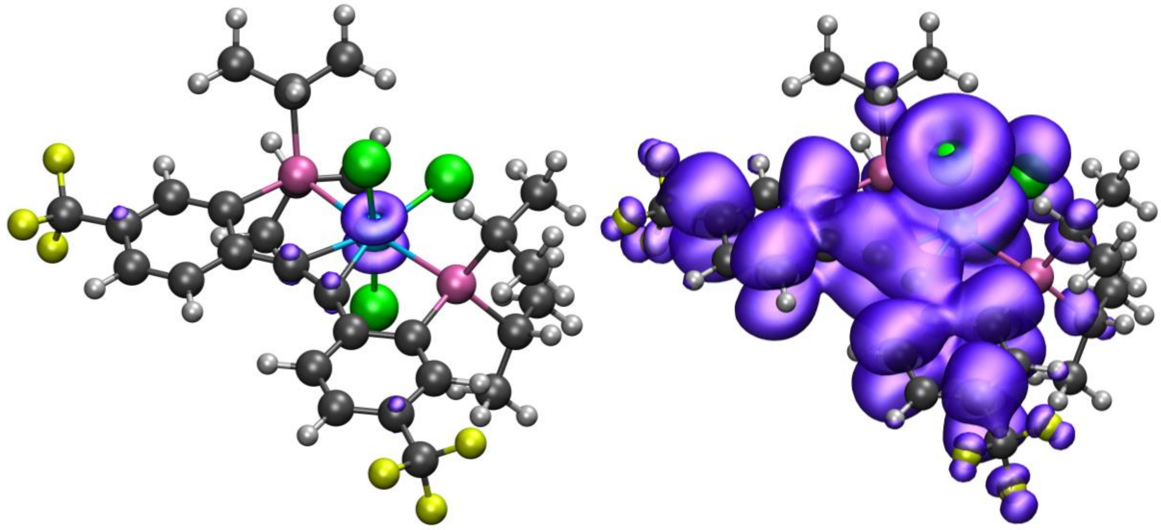

Figure S116. Spin density (left: isovalue of 0.04; right: isovalue of 0.0004) for *mer*-[$Re^{V}Cl_3$(κ^4-*As*,*CC*,*As*-L^{iPr})].

S4.12 *mer*-$[Mn^{III}Cl_3(\kappa^4\text{-}As,CC,As\text{-}L^{iPr})]$: singlet/d^2

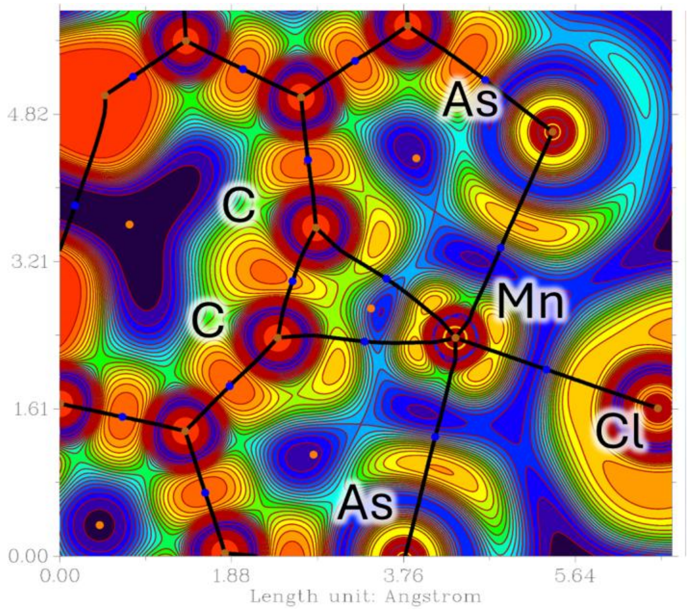

Figure S117. Electron localization function (ELF) map with topological features of ρ(r) ((3,-3) critical points: brown, (3,-1) critical points: blue, (3,+1) critical points: orange, bond paths between (3,-3)/(3,-1) critical points: black) for *mer*-$[Mn^{III}Cl_3(\kappa^4\text{-}As,CC,As\text{-}L^{iPr})]$ cut through the M···C≡C plane.

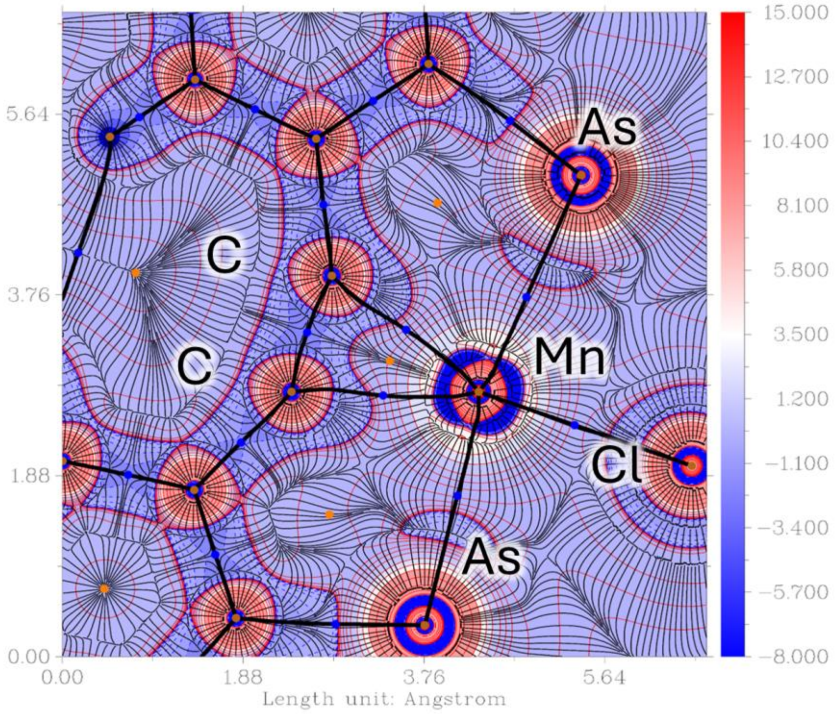

Figure S118. Laplacian of the electron density, $\nabla^2\rho(r)$, map with topological features of ρ(r) ((3,-3) critical points: brown, (3,-1) critical points: blue, (3,+1) critical points: orange, bond paths between (3,-3)/(3,-1) critical points: black) for *mer*-$[Mn^{III}Cl_3(\kappa^4\text{-}As,CC,As\text{-}L^{iPr})]$ cut through the M···C≡C plane.

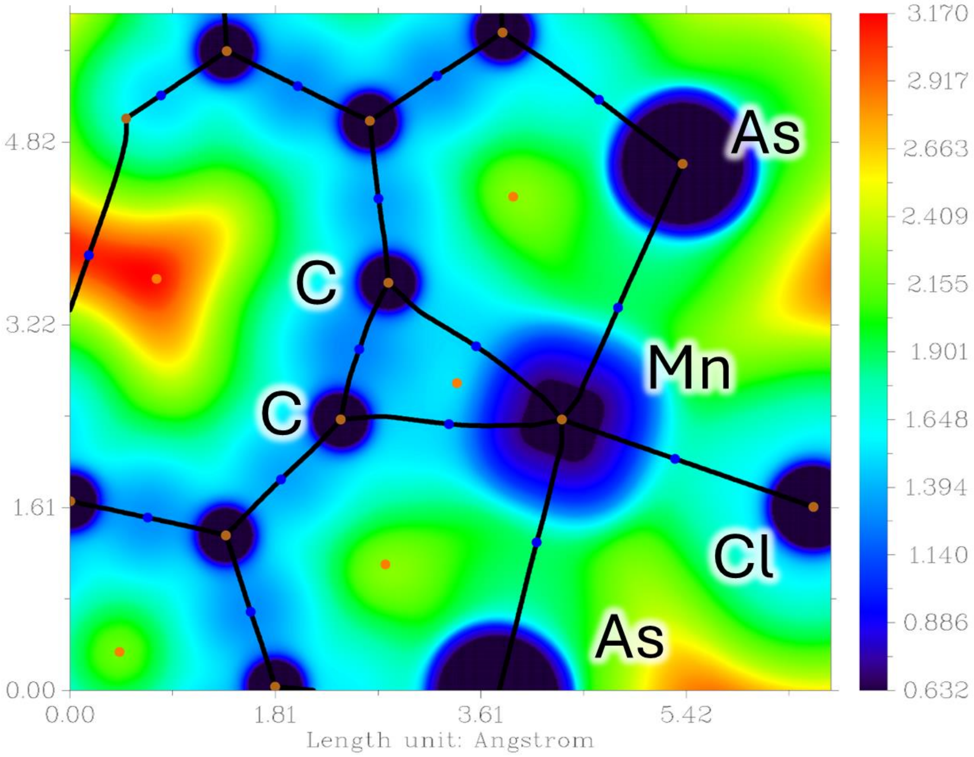

Figure S119. Electron delocalization range function (EDR) map with topological features of ρ(r) ((3,-3) critical points: brown, (3,-1) critical points: blue, (3,+1) critical points: orange, bond paths between (3,-3)/(3,-1) critical points: black) for *mer*-[$Mn^{III}Cl_3$(κ[4]-*As*,*CC*,*As*-L^{iPr})] cut through the M···C≡C plane.

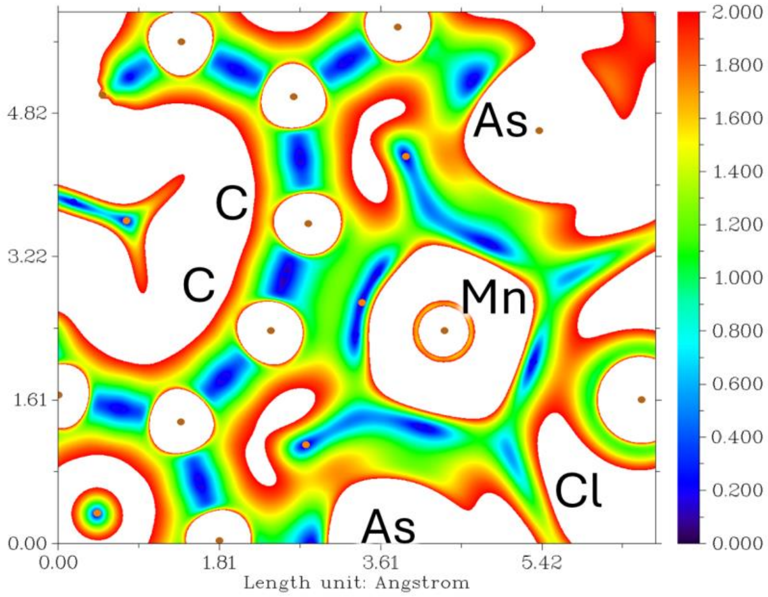

Figure S120. Interaction region indicator (IRI) map with topological features of ρ(r) ((3,-3) critical points: brown, (3,-1) critical points: blue, (3,+1) critical points: orange) for *mer*-[$Mn^{III}Cl_3$(κ[4]-*As*,*CC*,*As*-L^{iPr})] cut through the M···C≡C plane.

S4.13 *mer*-[$Mn^{III}Cl_3$(κ^4-*As*,*CC*,*As*-L^{iPr})]: triplet/d^4-ls

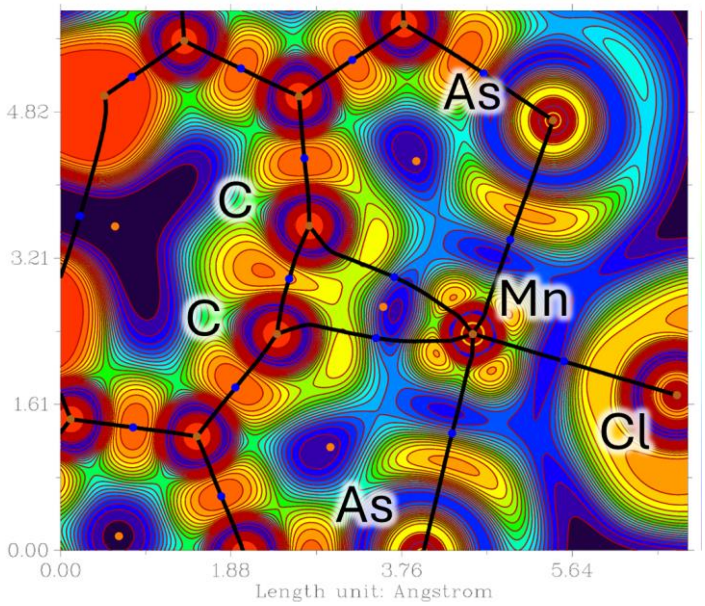

Figure S121. Electron localization function (ELF) map with topological features of ρ(r) ((3,-3) critical points: brown, (3,-1) critical points: blue, (3,+1) critical points: orange, bond paths between (3,-3)/(3,-1) critical points: black) for *mer*-[$Mn^{III}Cl_3$(κ^4-*As*,*CC*,*As*-L^{iPr})] cut through the M···C≡C plane.

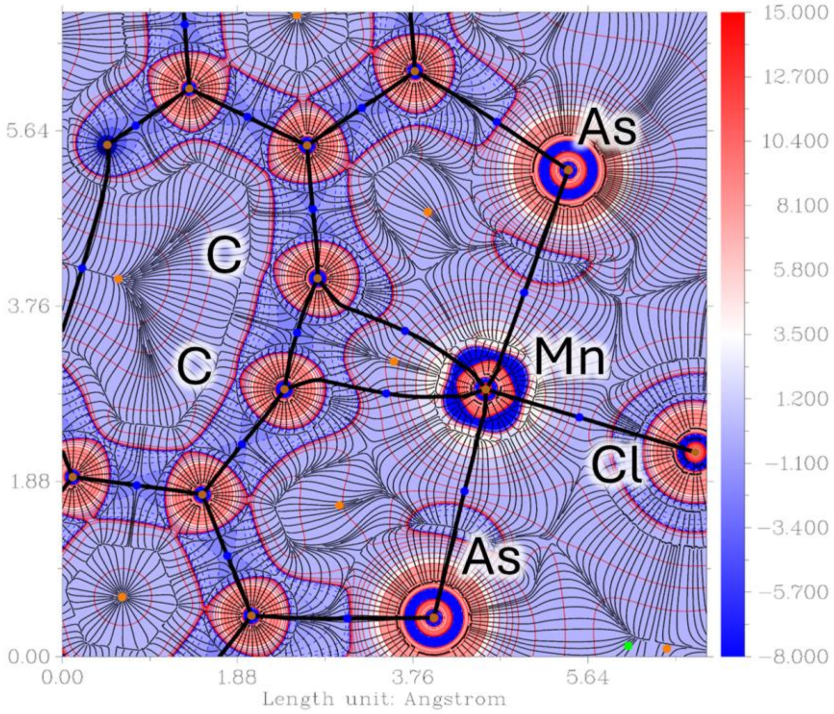

Figure S122. Laplacian of the electron density, $\nabla^2\rho(r)$, map with topological features of ρ(r) ((3,-3) critical points: brown, (3,-1) critical points: blue, (3,+1) critical points: orange, bond paths between (3,-3)/(3,-1) critical points: black) for *mer*-[$Mn^{III}Cl_3$(κ^4-*As*,*CC*,*As*-L^{iPr})] cut through the M···C≡C plane.

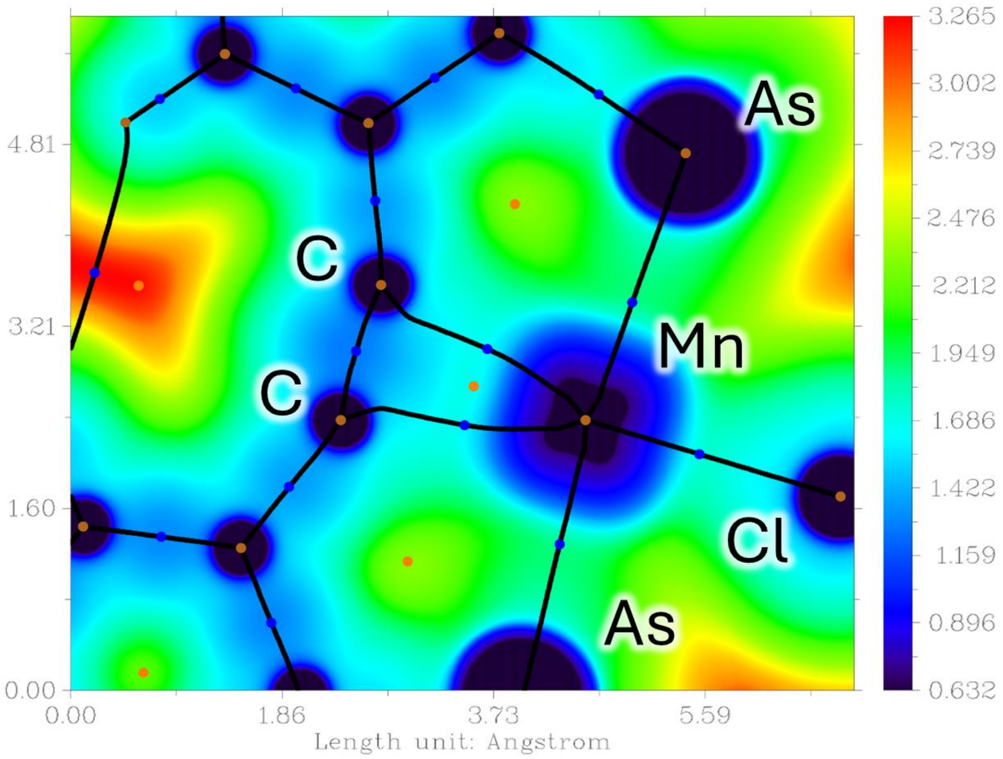

Figure S123. Electron delocalization range function (EDR) map with topological features of ρ(r) ((3,-3) critical points: brown, (3,-1) critical points: blue, (3,+1) critical points: orange, bond paths between (3,-3)/(3,-1) critical points: black) for *mer*-[$Mn^{III}Cl_3$(κ[4]-*As*,*CC*,*As*-L^{iPr})] cut through the M···C≡C plane.

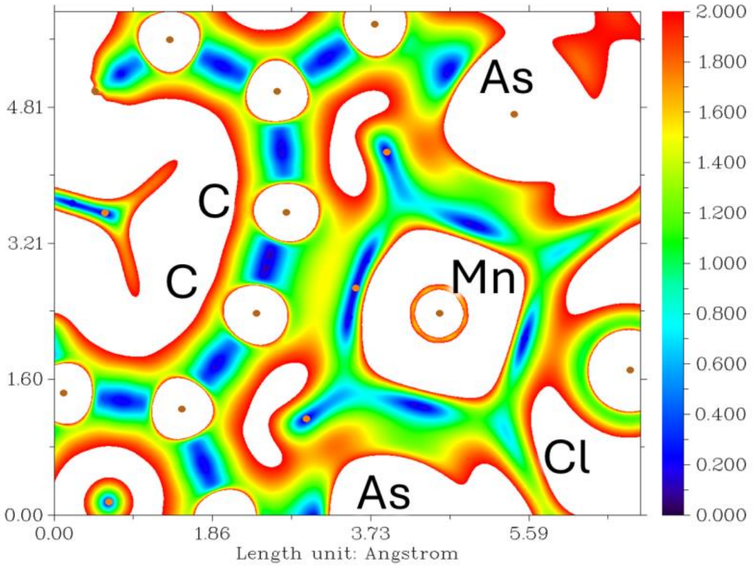

Figure S124. Interaction region indicator (IRI) map with topological features of ρ(r) ((3,-3) critical points: brown, (3,-1) critical points: blue, (3,+1) critical points: orange) for *mer*-[$Mn^{III}Cl_3$(κ[4]-*As*,*CC*,*As*-L^{iPr})] cut through the M···C≡C plane.

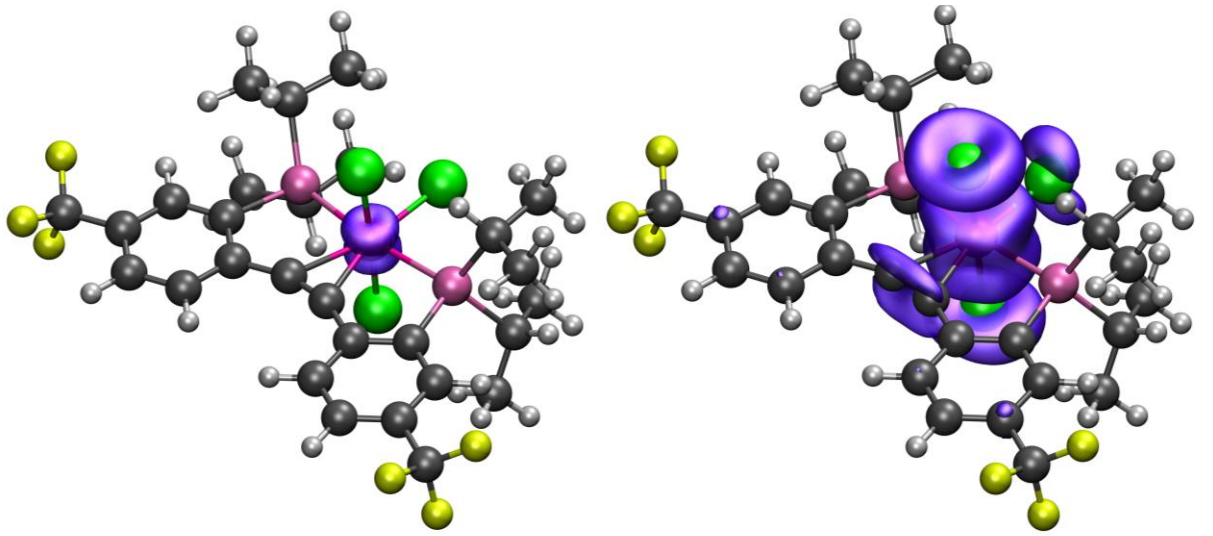

Figure S125. Spin density (left: isovalue of 0.04; right: isovalue of 0.0004) for *mer*-[$Mn^{III}Cl_3$(κ^4-*As*,*CC*,*As*-L^{iPr})].

S4.14 *mer*-[$Mn^{III}Cl_3$(κ^2-*As*,*CC*,*As*-L^{iPr})]: quintet/d^4-hs; energetically preferred

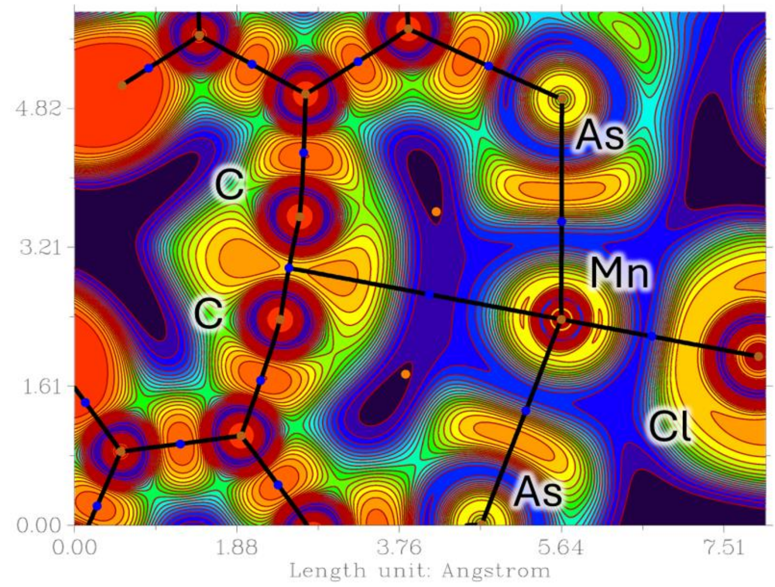

Figure S126. Electron localization function (ELF) map with topological features of ρ(r) ((3,-3) critical points: brown, (3,-1) critical points: blue, (3,+1) critical points: orange, bond paths between (3,-3)/(3,-1) critical points: black) for *mer*-[$Mn^{III}Cl_3$(κ^2-*As*,*CC*,*As*-L^{iPr})] cut through the M···C≡C plane.

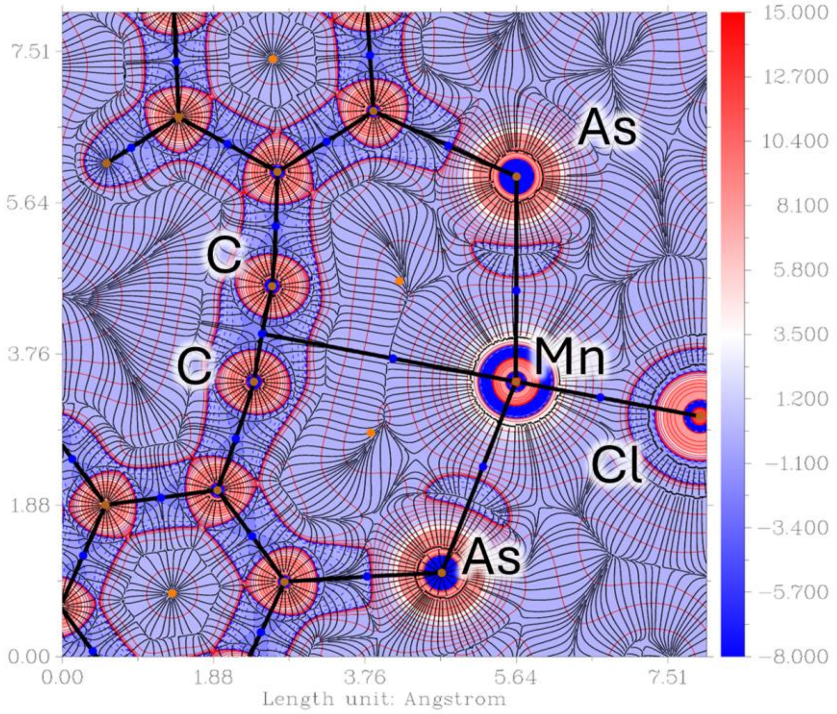

Figure S127. Laplacian of the electron density, $\nabla^2\rho(r)$, map with topological features of ρ(r) ((3,-3) critical points: brown, (3,-1) critical points: blue, (3,+1) critical points: orange, bond paths between (3,-3)/(3,-1) critical points: black) for *mer*-[$Mn^{III}Cl_3$(κ^2-*As*,*CC*,*As*-L^{iPr})] cut through the M···C≡C plane.

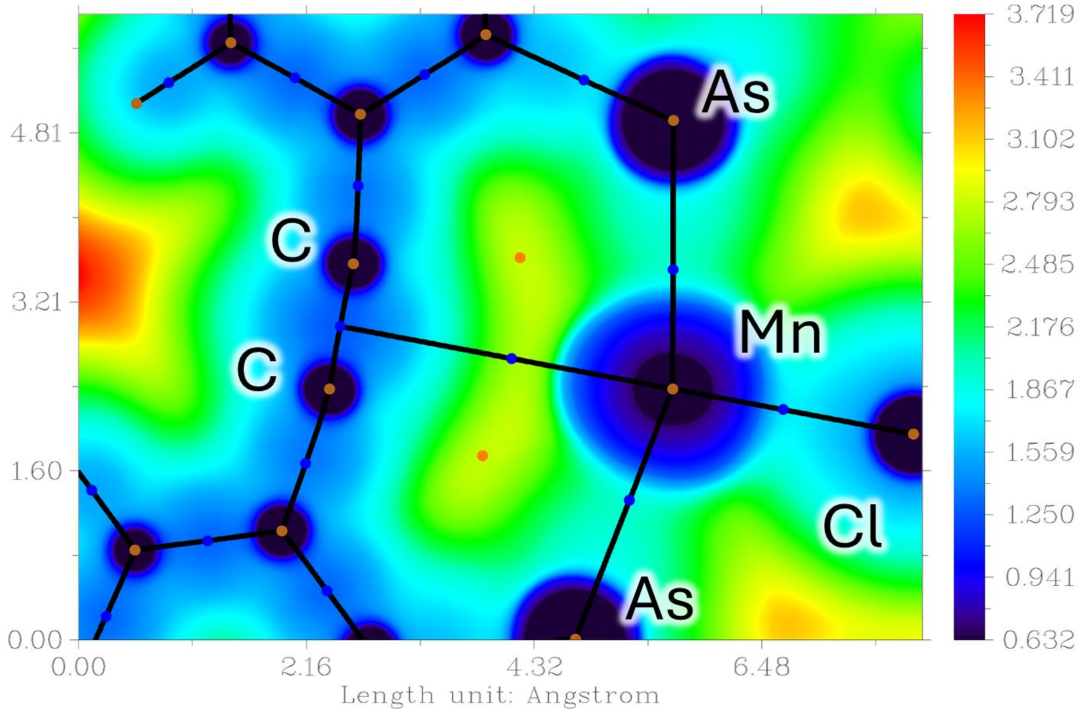

Figure S128. Electron delocalization range function (EDR) map with topological features of ρ(r) ((3,-3) critical points: brown, (3,-1) critical points: blue, (3,+1) critical points: orange, bond paths between (3,-3)/(3,-1) critical points: black) for *mer*-[$Mn^{III}Cl_3$(κ[2]-*As*,*CC*,*As*-L[*i*Pr])] cut through the M···C≡C plane.

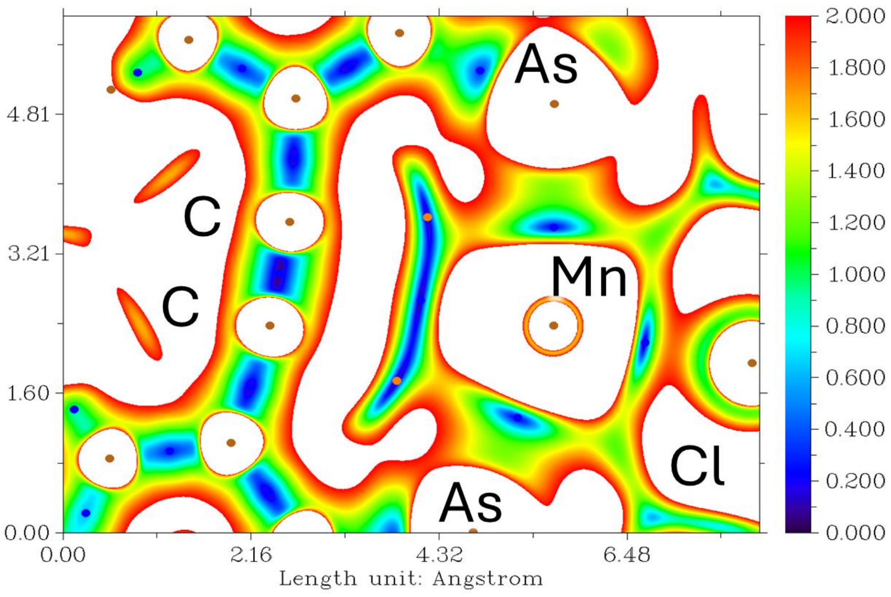

Figure S129. Interaction region indicator (IRI) map with topological features of ρ(r) ((3,-3) critical points: brown, (3,-1) critical points: blue, (3,+1) critical points: orange) for *mer*-[$Mn^{III}Cl_3$(κ[2]-*As*,*CC*,*As*-L[*i*Pr])] cut through the M···C≡C plane.

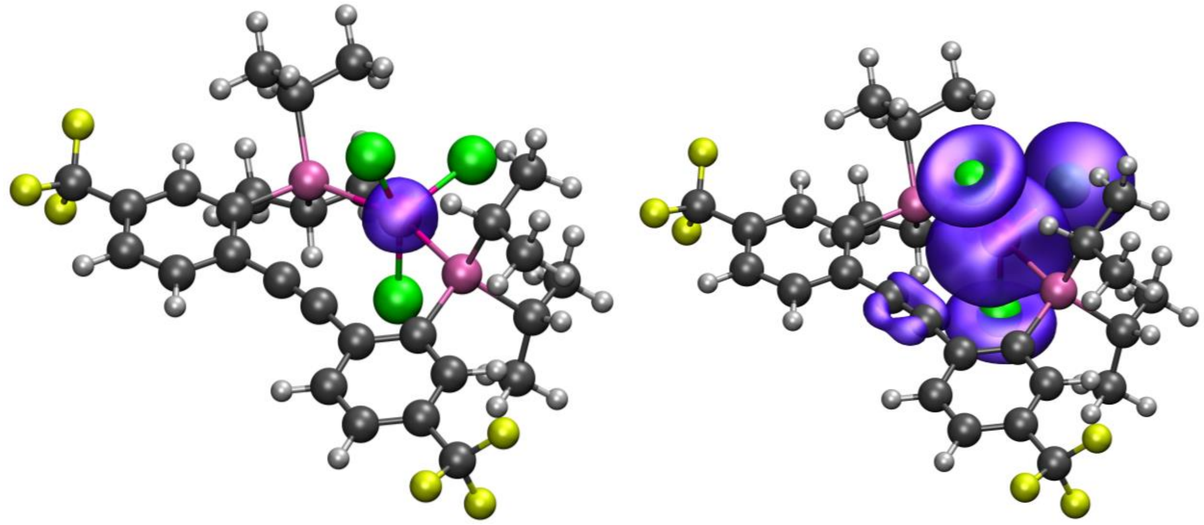

Figure S130. Spin density (left: isovalue of 0.04; right: isovalue of 0.0004) for *mer*-[$Mn^{III}Cl_3$(κ^2-*As*,*CC*,*As*-L^{iPr})].

S4.15 Computational details: alkyne vs. vinylidene carbene coordination for zero valent d-block elements

DFT calculations were performed on the high-performance computing systems of the Freie Universität Berlin ZEDAT (Curta),[110] and of the state of Baden-Württemberg (bwFOR cluster JUSTUS) using the program package GAUSSIAN 16.[111] The gas phase geometry optimizations were performed using coordinates modeled using GAUSSVIEW and Avogadro involving an implicit polarizable continuum model with integral equation formalism (IEF-PCM) for the solvent tetrahydrofurane.[112, 113] Frequency calculations following the geometry optimization were performed to validate the obtained geometries as energetic minima and showed no imaginary frequencies in all cases. The calculations were performed with the hybrid density functional B3LYP with Grimme dispersion and Becke-Johnson damping,[114] as implemented in GAUSSIAN.[115-117] The quadruple-ζ (pseudo)potential basis set def2-QZVPP was used for all atoms.[118, 149, 150] In some cases, preoptimization using smaller basis sets was required due to SCF convergence issues. The basis sets as well as the ECPs were obtained from the basis set exchange database.[121] Further analyses were performed with the free multifunctional wavefunction analyzer *Multiwfn*.[122]

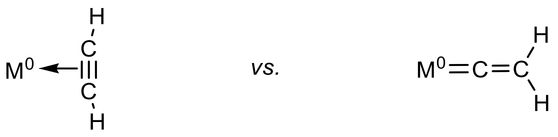

Figure S131. Considered geometries for hypothetical [M^0···(HC≡CH)] π model complexes and [M^0=C=CH$_2$] vinylidene model complexes (groups 3-12; periods 4-6).

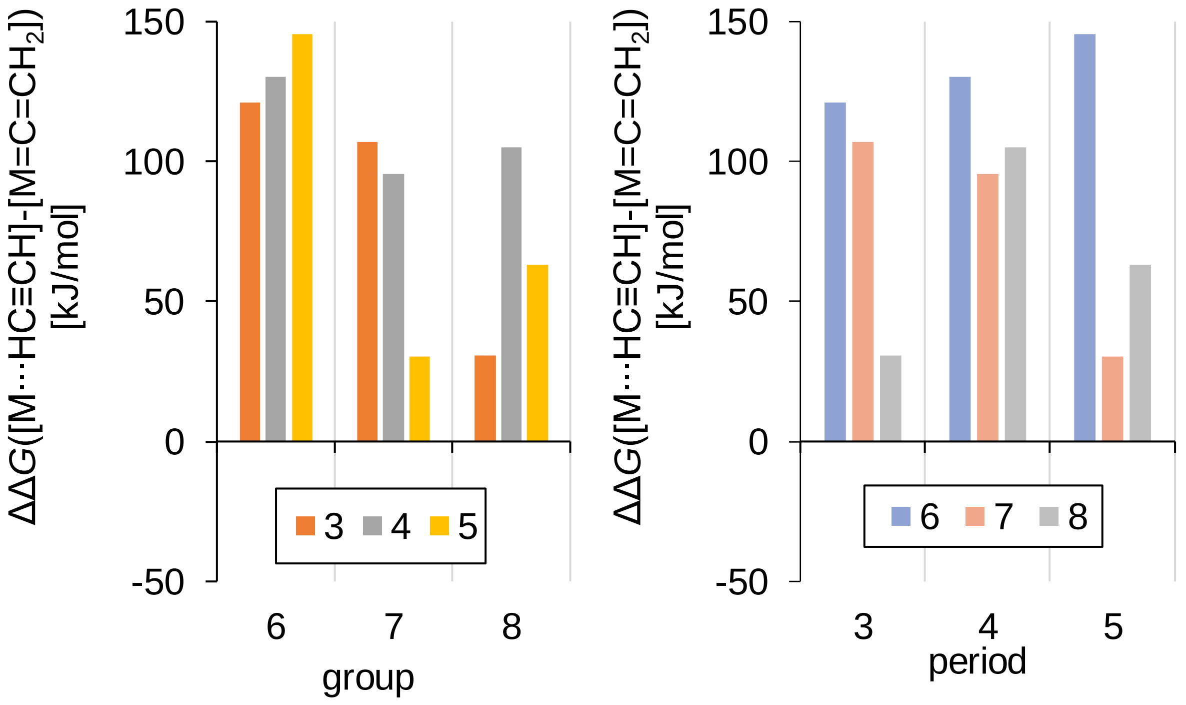

Figure S132. Free energy differences between hypothetical [M^0···(HC≡CH)] π model complexes and the isomeric [M^0=C=CH$_2$] vinylidene model complexes for group 6, 7 and 8 elements (periods 4-6); left: sorted by group; right: sorted by period.

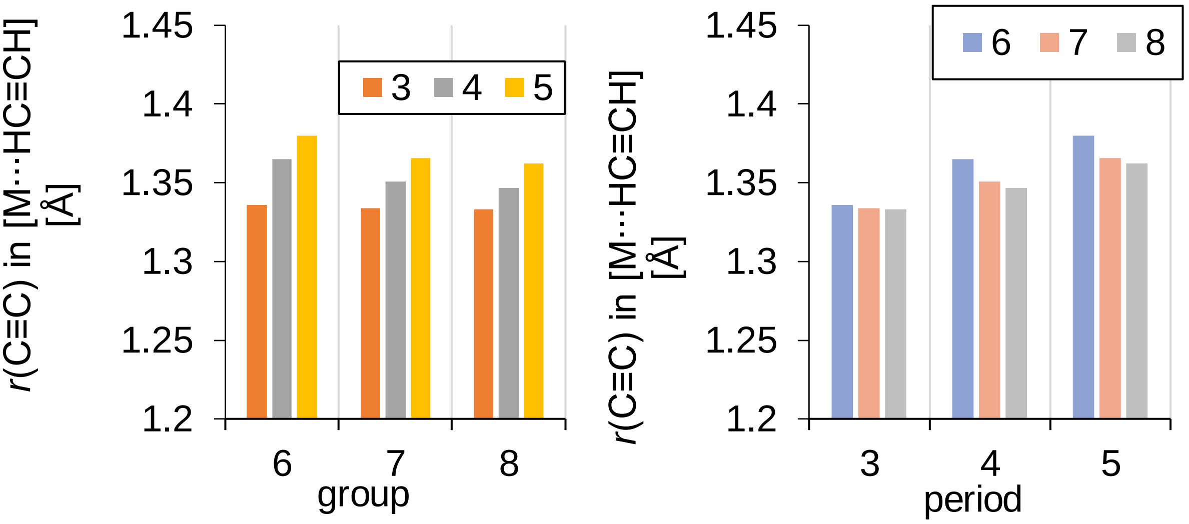

Figure S133. Overview over groups 6, 7 and 8 (periods 4-6) C≡C bond lengths in hypothetical [M^0···(HC≡CH)] π model complexes (left: sorted by group; right: sorted by period).

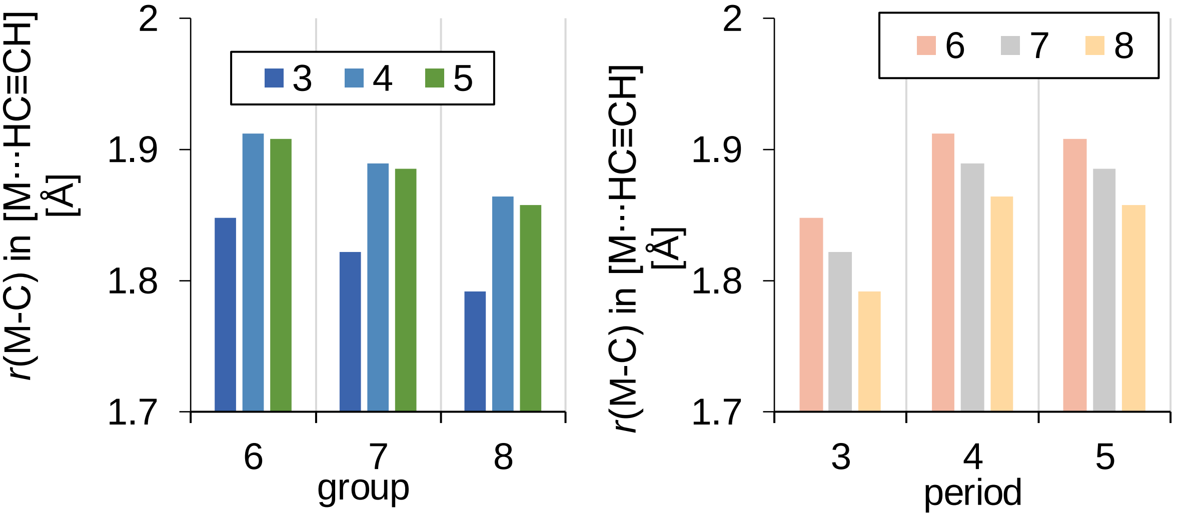

Figure S134. Overview over groups 6, 7 and 8 (periods 4-6) M-C bond lengths in hypothetical [M^0···(HC≡CH)] π model complexes (left: sorted by group; right: sorted by period).

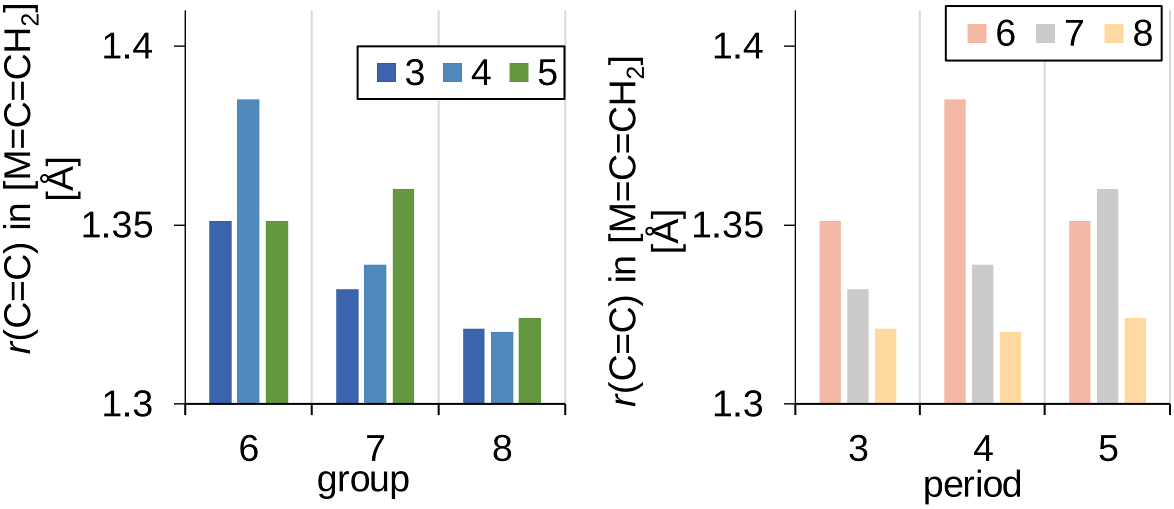

Figure S135. Overview over groups 6, 7 and 8 (periods 4-6) C=C bond lengths in hypothetical [M^0=C=CH_2] vinylidene model complexes (left: sorted by group; right: sorted by period).

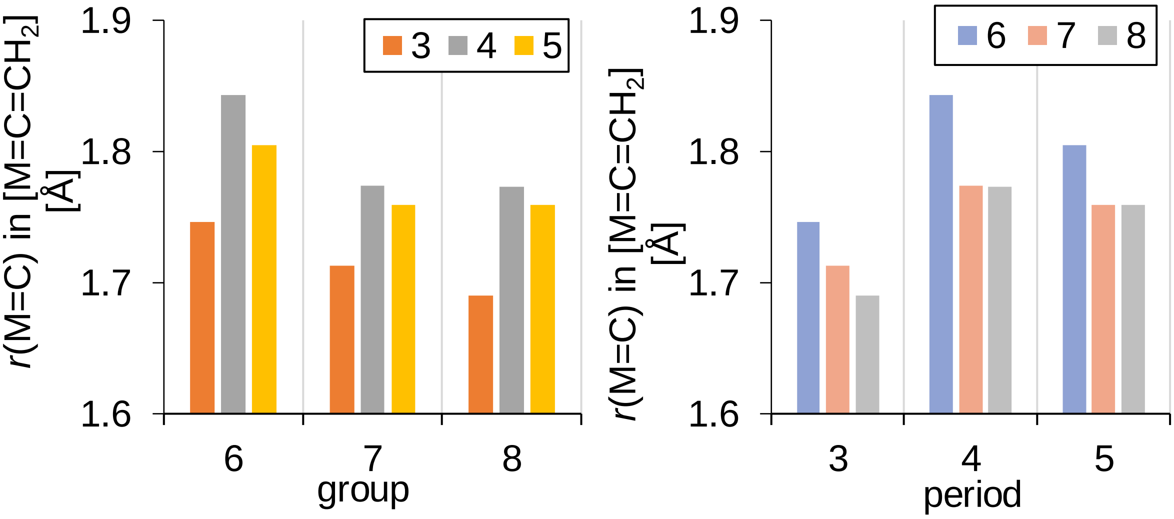

Figure S136. Overview over groups 6, 7 and 8 (periods 4-6) M=C bond lengths in hypothetical [M^0=C=CH_2] vinylidene model complexes (left: sorted by group; right: sorted by period).

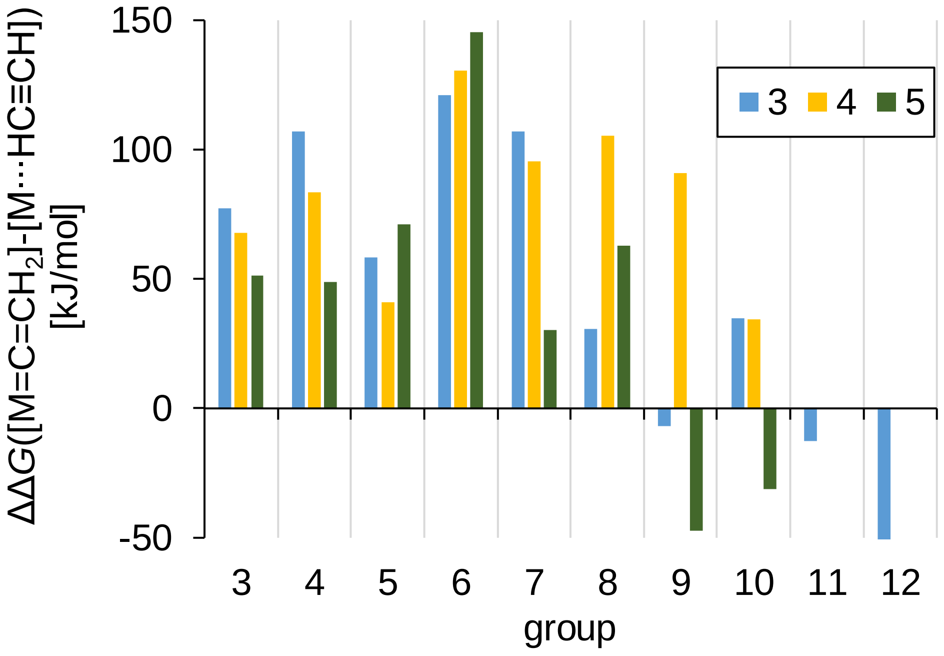

Figure S137. Free energy differences between hypothetical [M^0···(HC≡CH)] π model complexes and the isomeric [M^0=C=CH_2] vinylidene model complexes for d-element (groups 3-12; periods 4-6); sorted by groups.

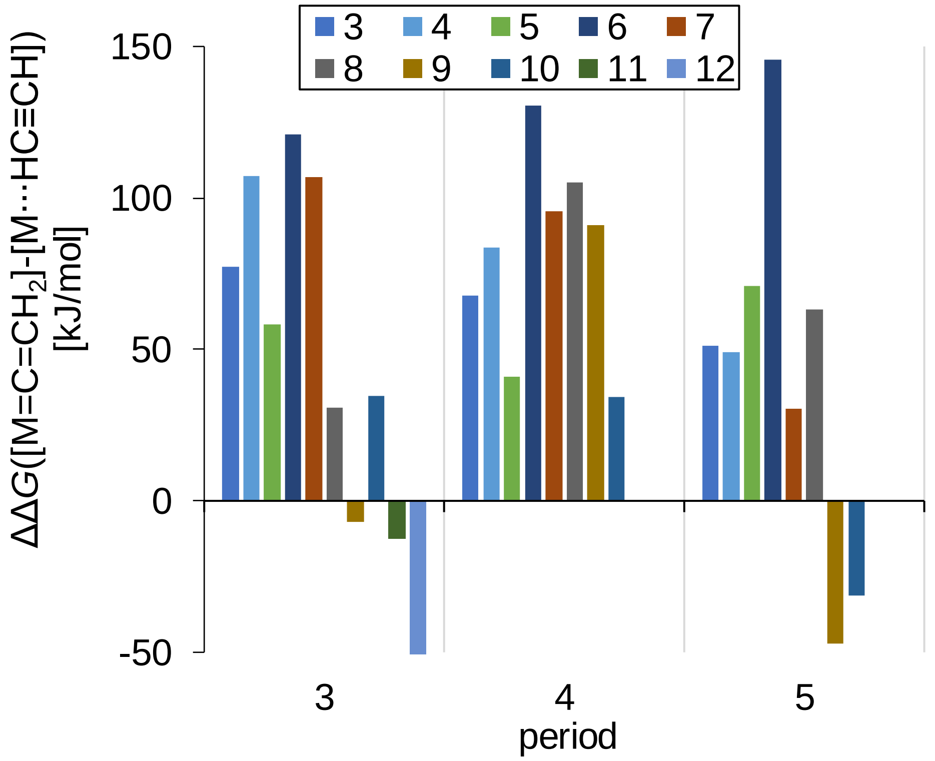

Figure S138. Free energy differences between hypothetical [M^0···(HC≡CH)] π model complexes and the isomeric [M^0=C=CH_2] vinylidene model complexes for d-element (groups 3-12; periods 4-6); sorted by periods.

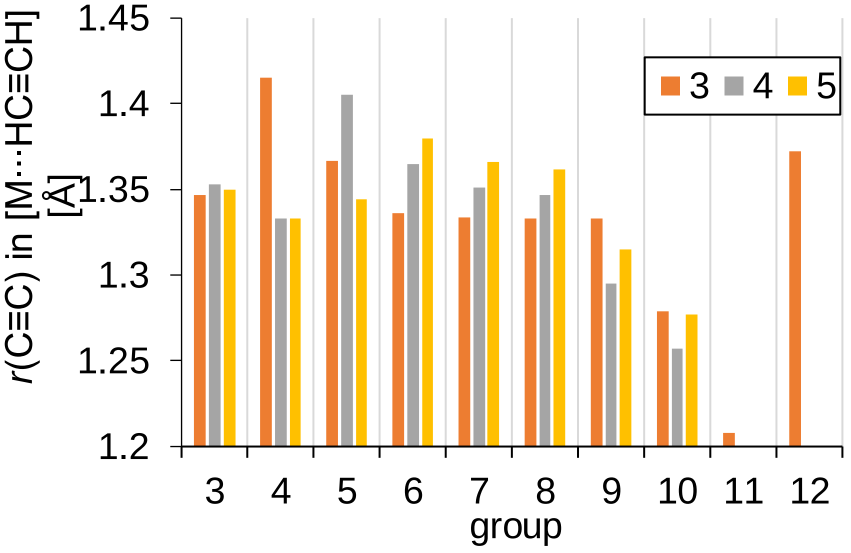

Figure S139. Overview over d-element (groups 3-12; periods 4-6) M-C bond lengths in hypothetical [M^0···(HC≡CH)] π model complexes; sorted by groups. Note that for Ag, Au, Cd and Hg dissociation of the alkyne from the metal was observed.

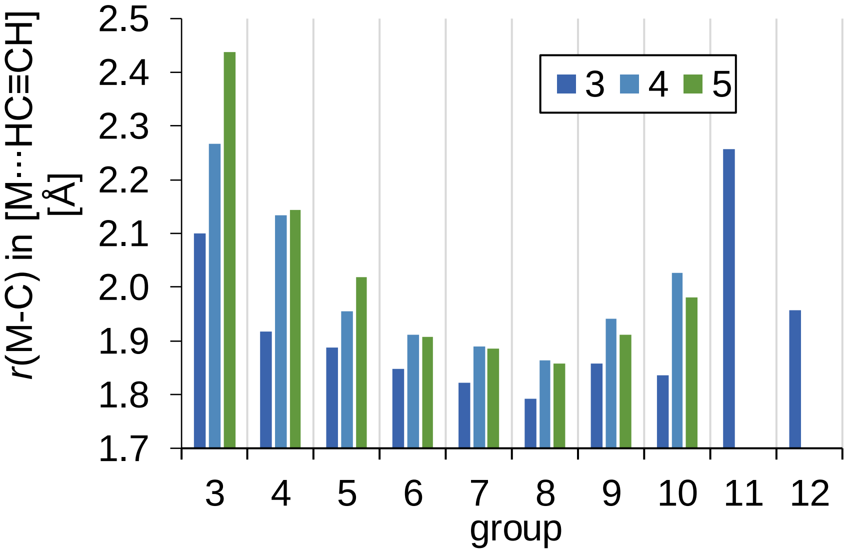

Figure S140. Overview over d-element (groups 3-12; periods 4-6) C≡C bond lengths in hypothetical [M^0···(HC≡CH)] π model complexes; sorted by groups. Note that for Ag, Au, Cd and Hg dissociation of the alkyne from the metal was observed.

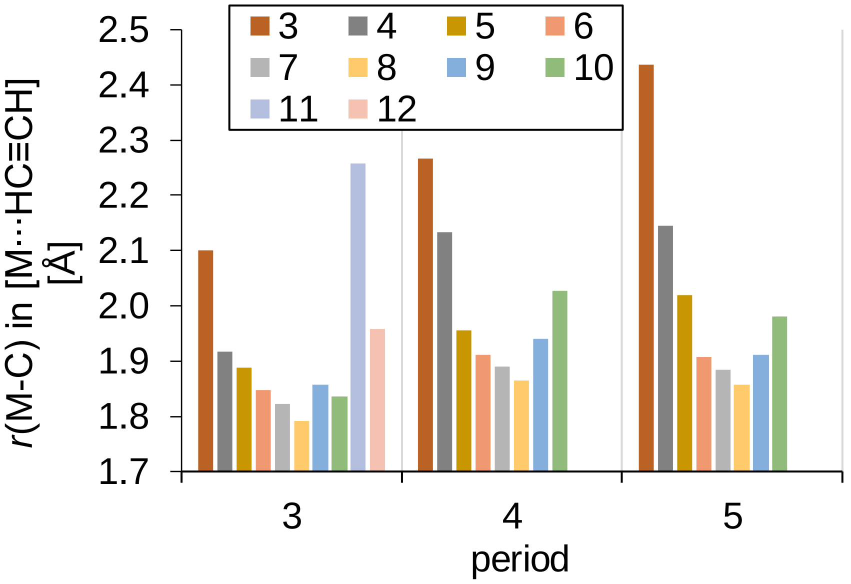

Figure S141. Overview over d-element (groups 3-12; periods 4-6) C≡C bond lengths in hypothetical [M^0···(HC≡CH)] π model complexes; sorted by periods. Note that for Ag, Au, Zn, Cd and Hg dissociation of the alkyne from the metal was observed.

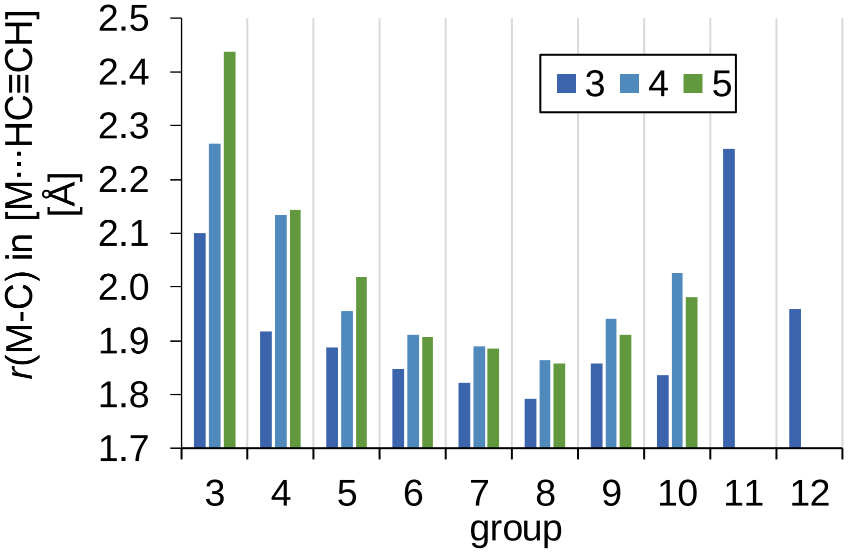

Figure S142. Overview over d-element (groups 3-12; periods 4-6) M-C bond lengths in hypothetical [M^0···(HC≡CH)] π model complexes; sorted by periods. Note that for Ag, Au, Zn, Cd and Hg dissociation of the alkyne from the metal was observed.

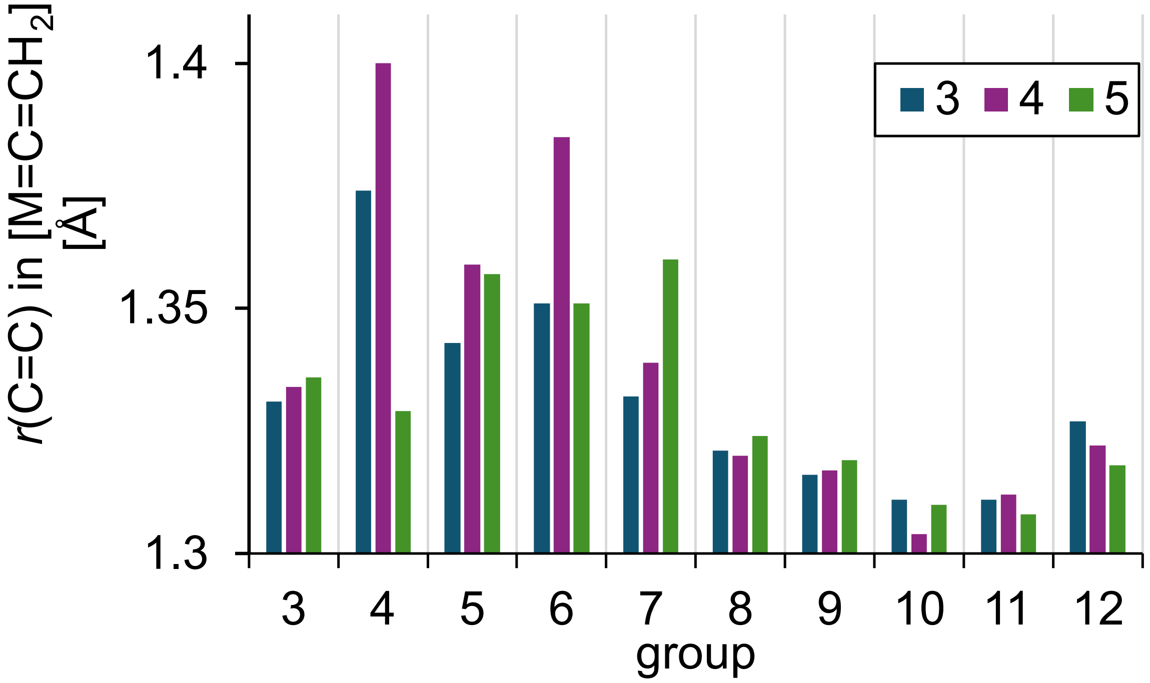

Figure S143. Overview over d-element (groups 3-12; periods 4-6) C=C bond lengths in hypothetical [M^0=C=CH_2] vinylidene model complexes; sorted by groups.

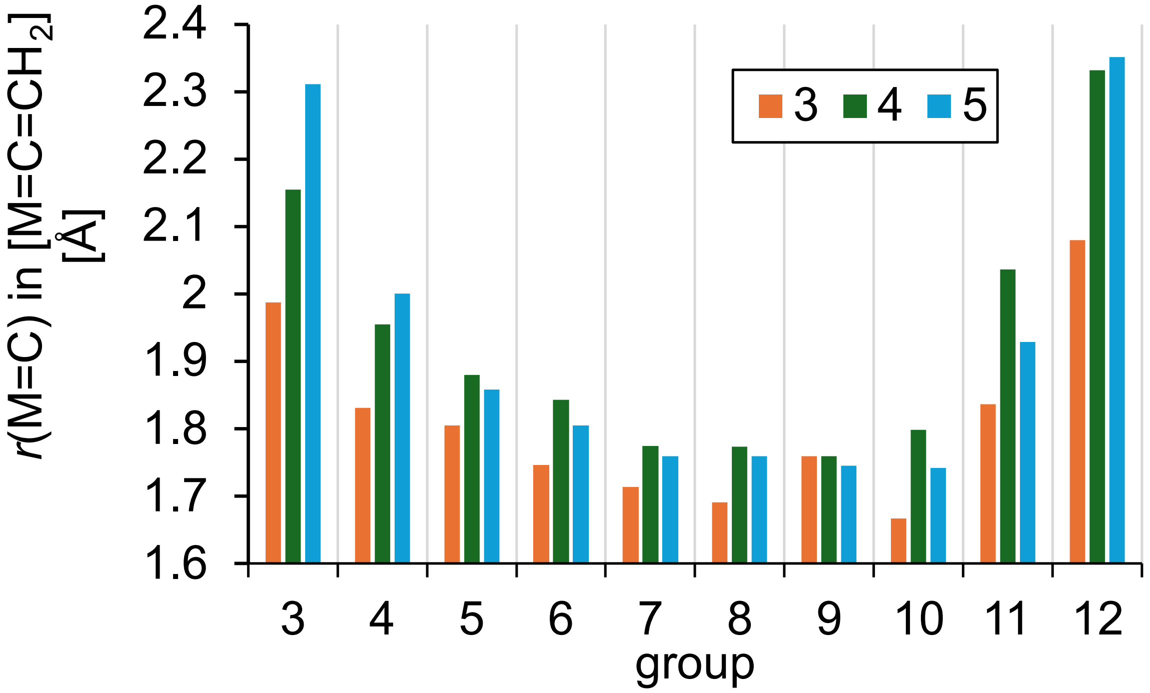

Figure S144. Overview over d-element (groups 3-12; periods 4-6) M=C bond lengths in hypothetical [M^0=C=CH_2] vinylidene model complexes; sorted by groups.

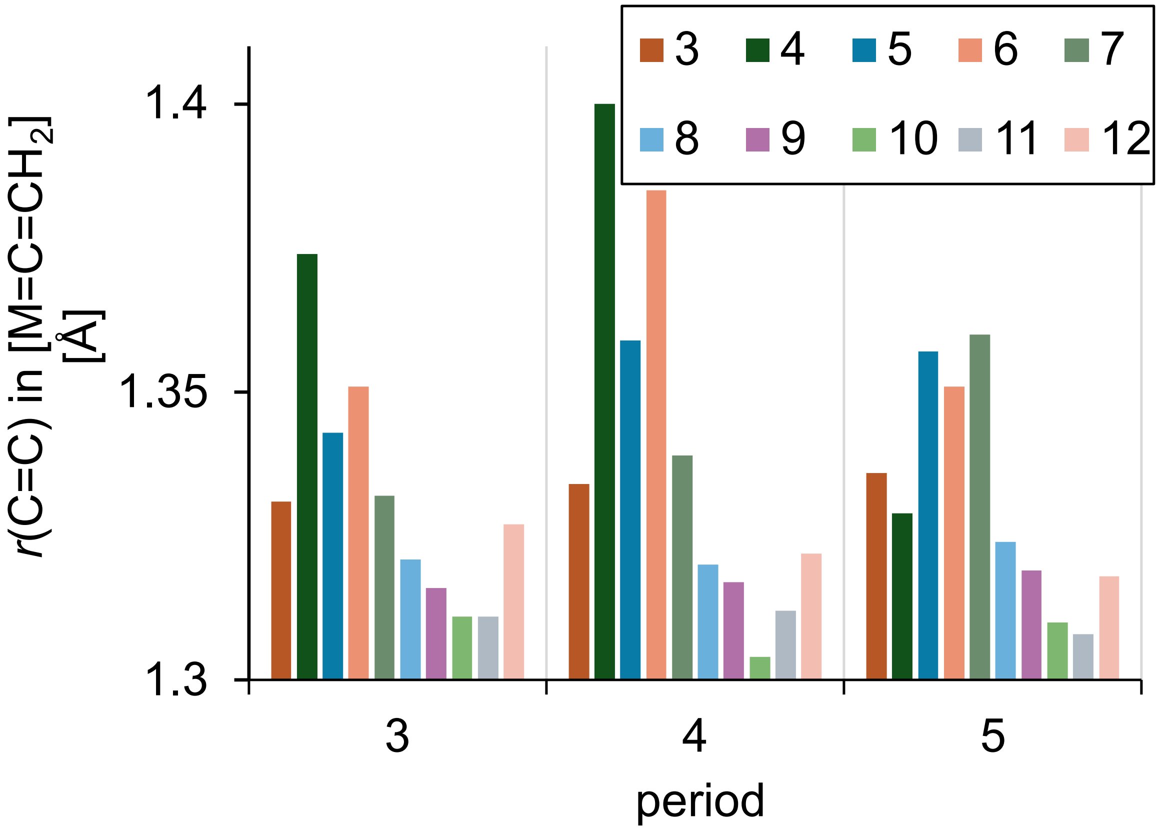

Figure S145. Overview over d-element (groups 3-12; periods 4-6) C=C bond lengths in hypothetical [M^0=C=CH_2] vinylidene model complexes; sorted by periods. Note that for Cu, Ag and Au a slightly bent and for Zn, Cd and Hg a near 90° bent was found.

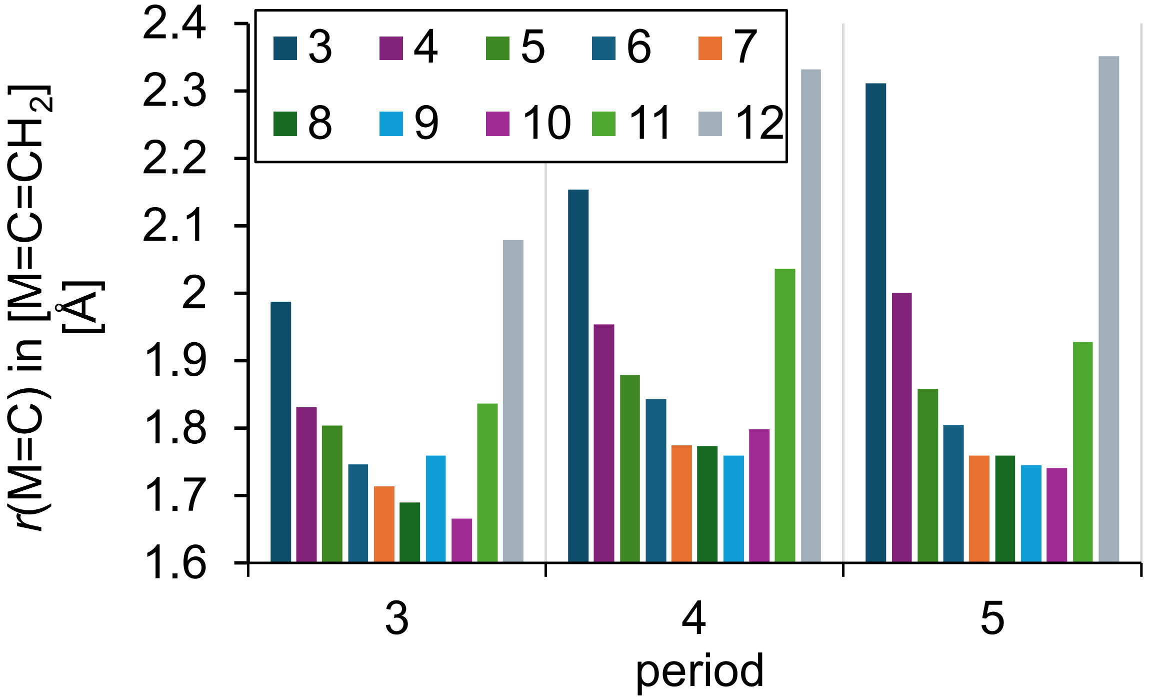

Figure S146. Overview over d-element (groups 3-12; periods 4-6) M=C bond lengths in hypothetical [M^0=C=CH_2] vinylidene model complexes; sorted by periods. Note that for Cu, Ag and Au a slightly bent and for Zn, Cd and Hg a near 90° bent was found.

S4.16 Computational details: alkyne vs. vinylidene carbene coordination for isostructural $[M\cdots(HC{\equiv}CH)N(Cl)_2(AsH_3)_2]^{+,0,-}$ and $[M{=}(C{=}CH_2)N(Cl)_2(AsH_3)_2]^{+,0,-}$ model complexes of groups 6, 7 and 8

DFT calculations were performed on the high-performance computing systems of the Freie Universität Berlin ZEDAT (Curta),[110] and of the state of Baden-Württemberg (bwFOR cluster JUSTUS) using the program package GAUSSIAN 16.[111] The gas phase geometry optimizations were performed using coordinates modeled using GAUSSVIEW and Avogadro involving an implicit polarizable continuum model with integral equation formalism (IEF-PCM) for the solvent tetrahydrofurane.[112, 113] Frequency calculations following the geometry optimization were performed to validate the obtained geometries as energetic minima and showed no imaginary frequencies in all cases (exception: for $[Cr\cdots(HC{\equiv}CH)N(Cl)_2(AsH_3)_2]^-$ a neglectable imaginary frequency was tolerated). For Fe dissociation into $[FeN(Cl)_2(AsH_3)_2]^-$ & HC≡CH instead of a side-on complex was found. The calculations were performed with the hybrid density functional B3LYP with Grimme dispersion and Becke-Johnson damping,[114] as implemented in GAUSSIAN.[115-117] The quadruple-ζ (pseudo)potential basis set def2-QZVPP was used for all atoms.[118, 149, 150] In some cases, preoptimization using smaller basis sets was required due to SCF convergence issues. The basis sets as well as the ECPs were obtained from the basis set exchange database.[121] Further analyses were performed with the free multifunctional wavefunction analyzer *Multiwfn*.[122]

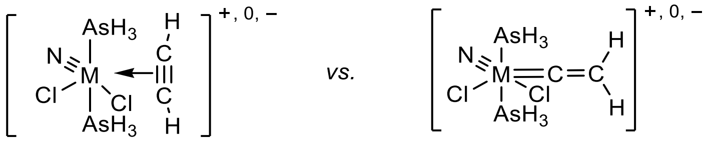

Figure S147. Considered geometries for hypothetical $[M\cdots(HC{\equiv}CH)N(Cl)_2(AsH_3)_2]^{+,0,-}$ π model complexes and $[M{=}(C{=}CH_2)N(Cl)_2(AsH_3)_2]^{+,0,-}$ vinylidene model complexes (groups 6-8; periods 4-6).

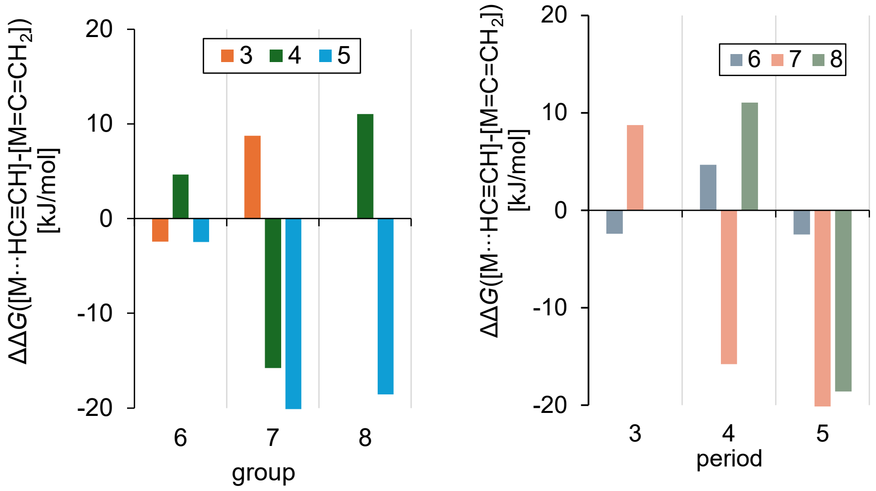

Figure S148. Free energy differences between hypothetical $[M\cdots(HC{\equiv}CH)N(Cl)_2(AsH_3)_2]^{+,0,-}$ π model complexes and $[M{=}(C{=}CH_2)N(Cl)_2(AsH_3)_2]^{+,0,-}$ vinylidene model complexes for group 6, 7 and 8 elements (periods 4-6); left: sorted by group; right: sorted by period. Note that for Fe dissociation of the alkyne in the potential side-on compound was observed and therefore no data are provided.

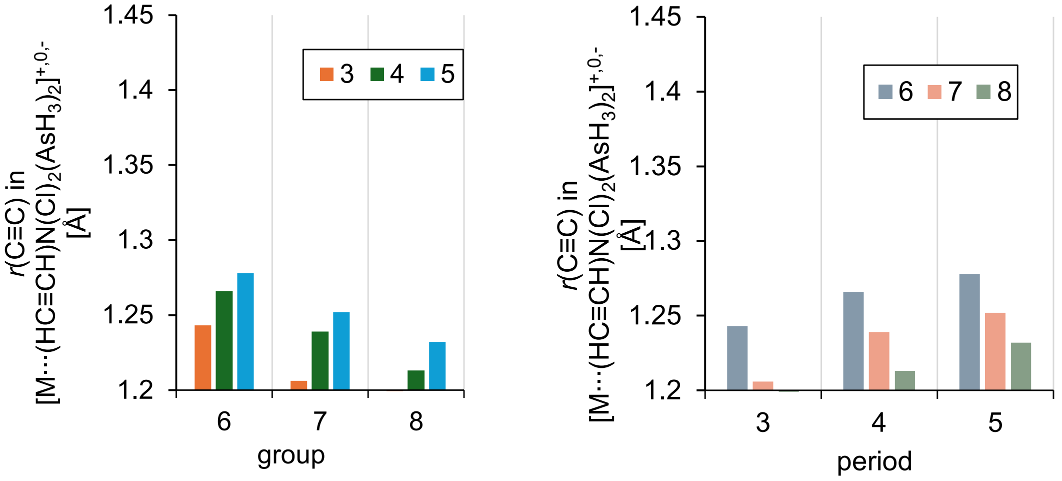

Figure S149. Overview over groups 6, 7 and 8 (periods 4-6) M-C bond lengths in hypothetical $[M\cdots(HC{\equiv}CH)N(Cl)_2(AsH_3)_2]^{+,0,-}$ π model complexes (left: sorted by group; right: sorted by period). Note that for Fe dissociation of the alkyne was observed and therefore no data are provided.

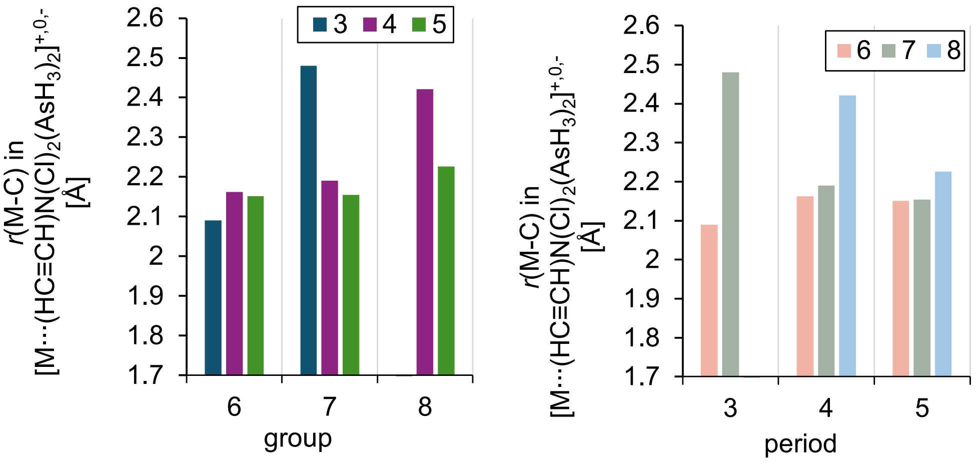

Figure S150. Overview over groups 6, 7 and 8 (periods 4-6) C≡C bond lengths in hypothetical $[M\cdots(HC{\equiv}CH)N(Cl)_2(AsH_3)_2]^{+,0,-}$ π model complexes (left: sorted by group; right: sorted by period). Note that for Fe dissociation of the alkyne was observed and therefore no data are provided.

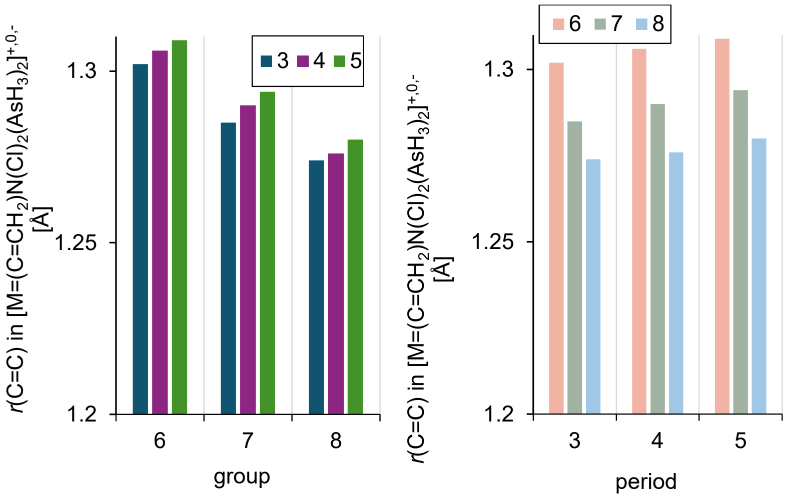

Figure S151. Overview over groups 6, 7 and 8 (periods 4-6) C=C bond lengths in hypothetical $[M{=}(C{=}CH_2)N(Cl)_2(AsH_3)_2]^{+,0,-}$ vinylidene model complexes (left: sorted by group; right: sorted by period).

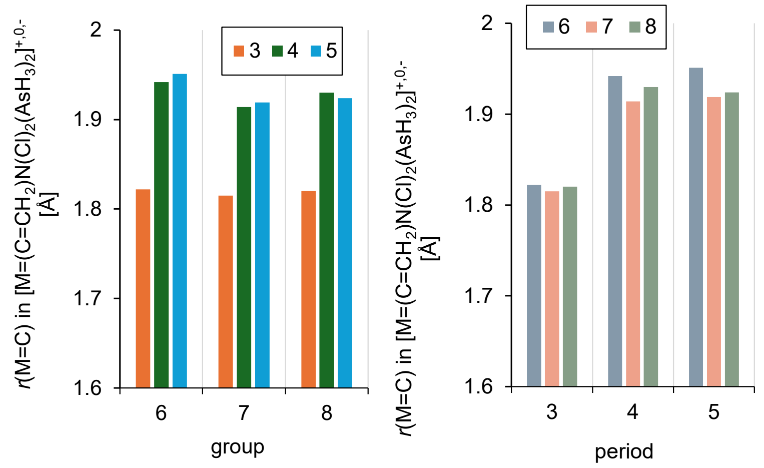

Figure S152. Overview over groups 6, 7 and 8 (periods 4-6) M=C bond lengths in hypothetical $[M{=}(C{=}CH_2)N(Cl)_2(AsH_3)_2]^{+,0,-}$ vinylidene model complexes (left: sorted by group; right: sorted by period).

Part S5: Additional figures and explanatory text

S5.1 Literature survey data on alkyne complexes of the d-block elements.

Transition metal alkyne complexes are ubiquitous in modern coordination and organometallic chemistry due to their importance as catalytic intermediates e.g. in copper-catalyzed azide-alkyne click-, oligomerization or (semi-)hydrogenation reactions. They have also attracted the fundamental interest of chemists due to their intriguing metal-ligand π-bonds that allow for the donation of a varying number of electrons depending on the number of π-bonds engaged in the metal-ligand interaction (i.e. $2e^-$ or $4e^-$). In the case of a side-on coordination of the alkyne, the higher the degree of donation into the unoccupied π* orbitals of the C-C triple bond, the smaller the angle between the two residues on the alkyne becomes, nearing a metallacyclopropene structure according to the Dewar–Chatt–Duncanson model. Especially terminal alkynes can undergo rearrangements to vinylidene carbenes; Fisher carbenes that may represent useful intermediates themselves. They may also act as bridging donor ligands, where each π-bond coordinates to a different metal center ($2e^-$ + $2e^-$).

Several striking differences between the organometallic chemistry of technetium and its group neighbor have been observed in the past.[1, 37] The most prominent regards the non-existence of any [$TcO_3(Cp^R)$] (Cp^R = unspecified cyclopentadienyl ligand), which eludes preparation to date, while [$ReO_3(Cp^R)$] is a widely used organometallic catalyst class and resembles an entry-point into a rich and diverse rhenium cyclopentadienyl chemistry.[1, 37] Similarly, the non-existence of alkyne complexes of technetium has puzzled chemists for decades due to the central position of technetium in the periodic table and the rich (structural) chemistry and reactivity of alkynes with the other d-block elements.[1-6] A complete analysis of reported structural data on crystalline d-block alkyne complexes surveyed from the Cambridge structural database,[33, 34] indicating technetium alkyne complexes as an equally elusive substance class is shown in Figure S141.

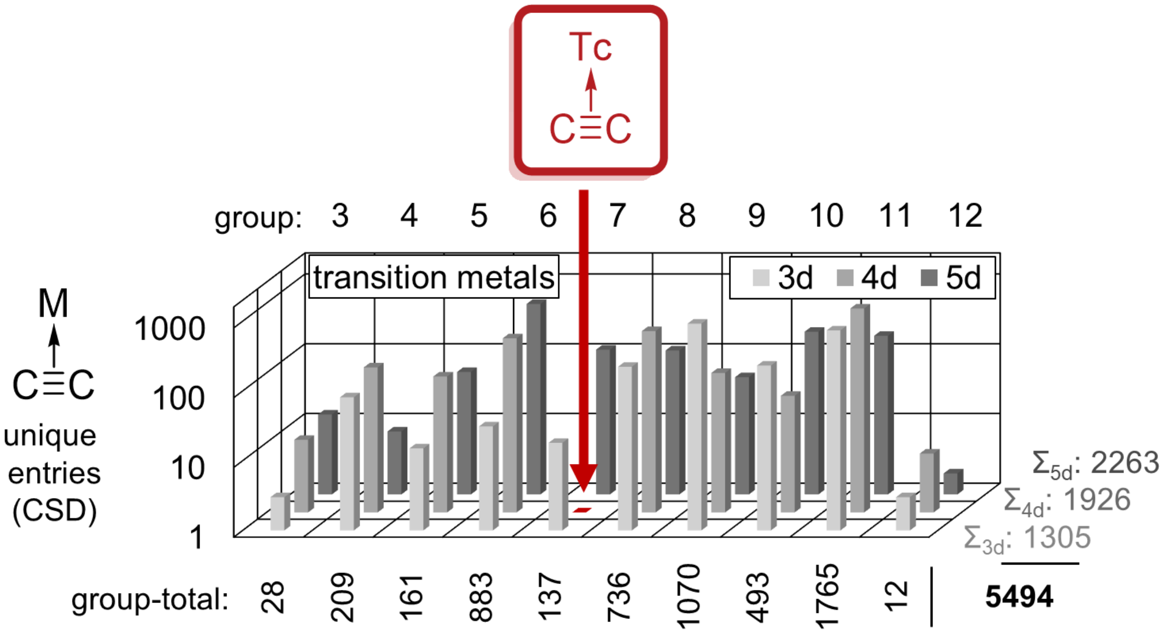

Figure S153. Literature survey of d-block alkyne complexes (groups 3-12; periods 4-6).

S5.2 C≡C bond lengths in structurally characterized *E*,(*CC*),*E* (E = P, As) complexes.[72-85]

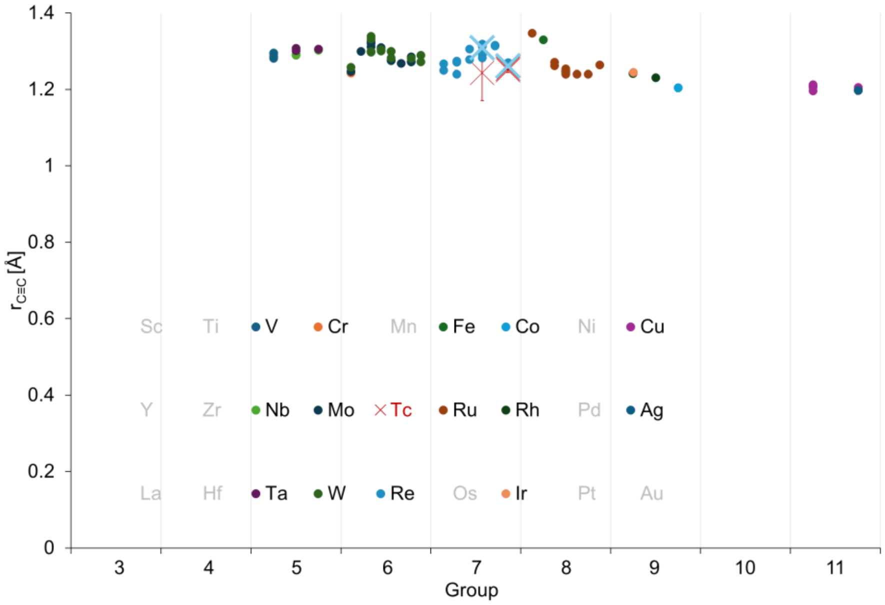

Figure S154. Overview over d-element (groups 3-11; periods 4-6) C≡C bond lengths in structurally characterized *E*,(*CC*),*E* (E = P, As) complexes with Tc/Re complexes of this study highlighted (depicted by X). Error bars are provided around (averaged if multiple data were available) bond lengths as minimum and maximum encountered values ± estimated standard deviation. Note that Group 3, 4, 9 and 10 complexes frequently undergo ligand cyclization.

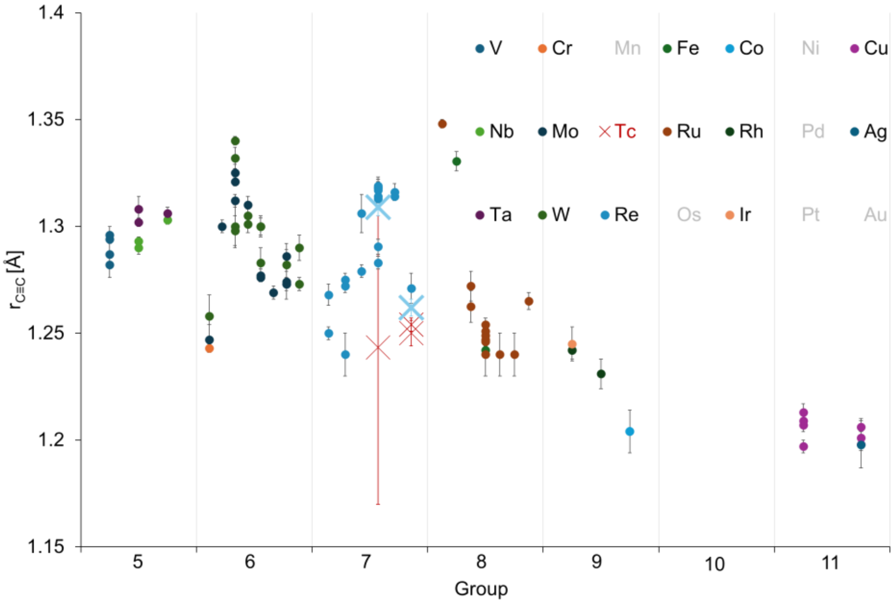

Figure S155. Close-up of group 5 to 11 (periods 4-6) d-element C≡C distance in *E*,(*CC*),*E* (E = P, As) complexes with Tc/Re complexes of this study highlighted (depicted by X). Error bars are provided around (averaged if multiple data were available) bond lengths as minimum and maximum encountered values ± estimated standard deviation. Note that Group 3, 4, 9 and 10 complexes frequently undergo ligand cyclization.

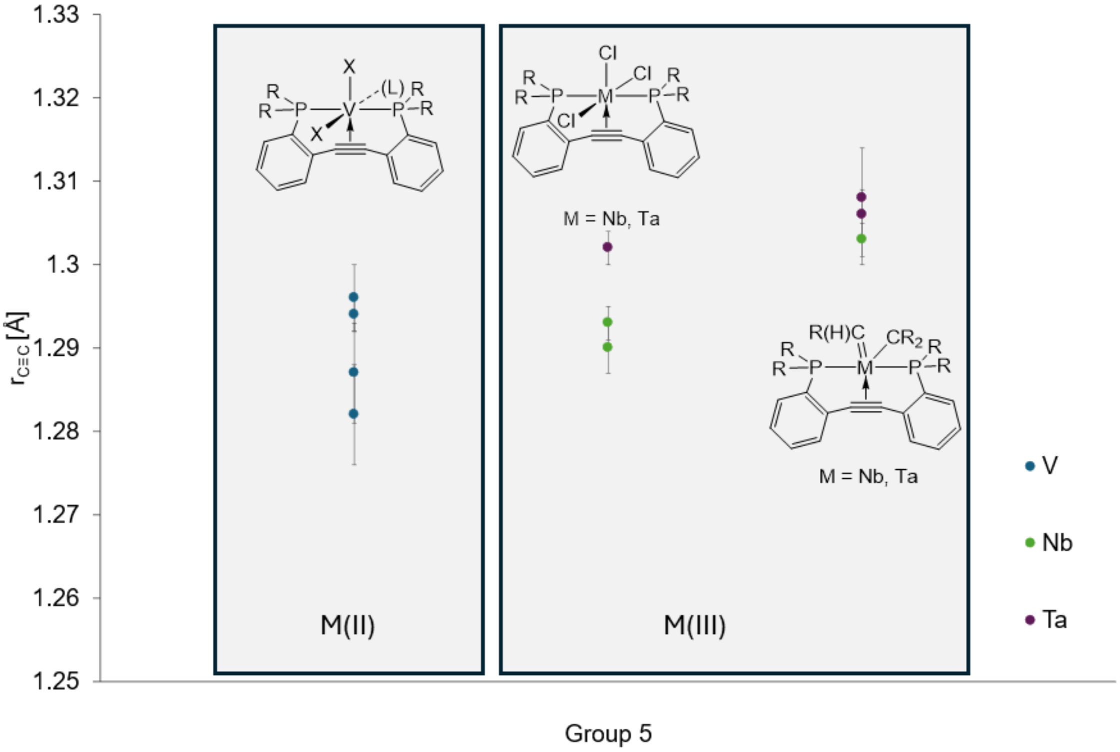

Figure S156. Close-up of group 5 (periods 4-6) d-element M-$C_{C≡C}$ distance in *E*,(*CC*),*E* (E = P, As) complexes. Error bars are provided around (averaged if multiple data were available) bond lengths as minimum and maximum encountered values ± estimated standard deviation.

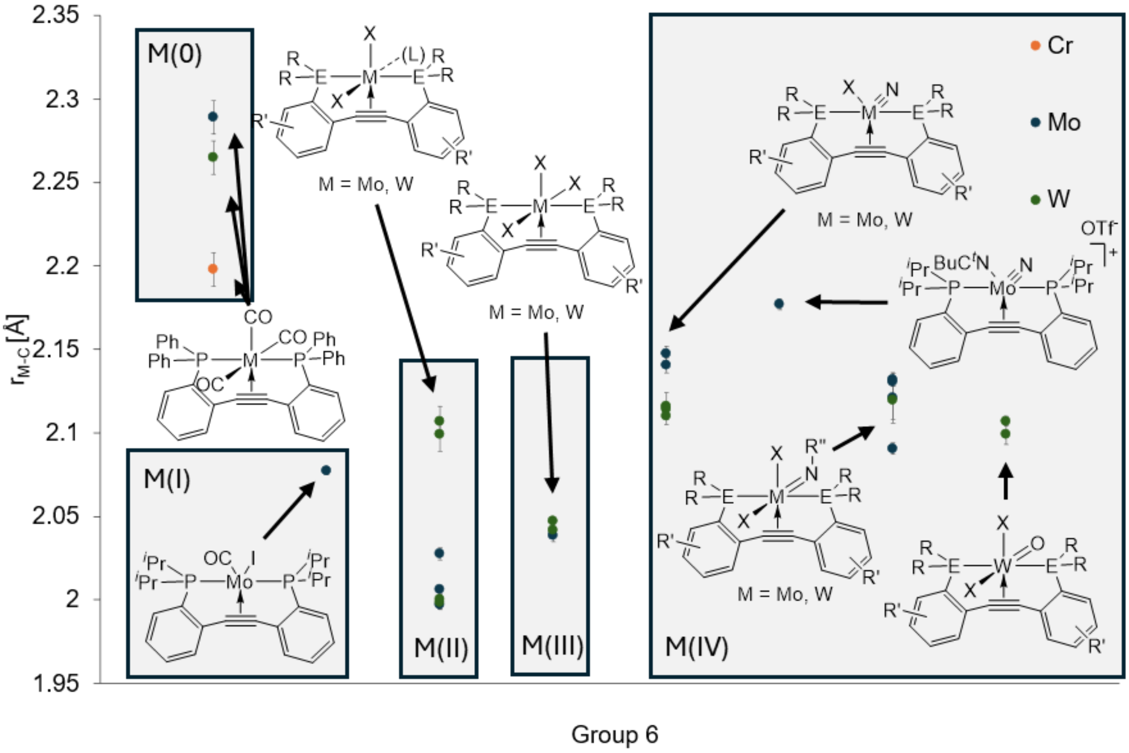

Figure S157. Close-up of group 6 (periods 4-6) d-element M-$C_{C≡C}$ distance in *E*,(*CC*),*E* (E = P, As) complexes. Error bars are provided around (averaged if multiple data were available) bond lengths as minimum and maximum encountered values ± estimated standard deviation.

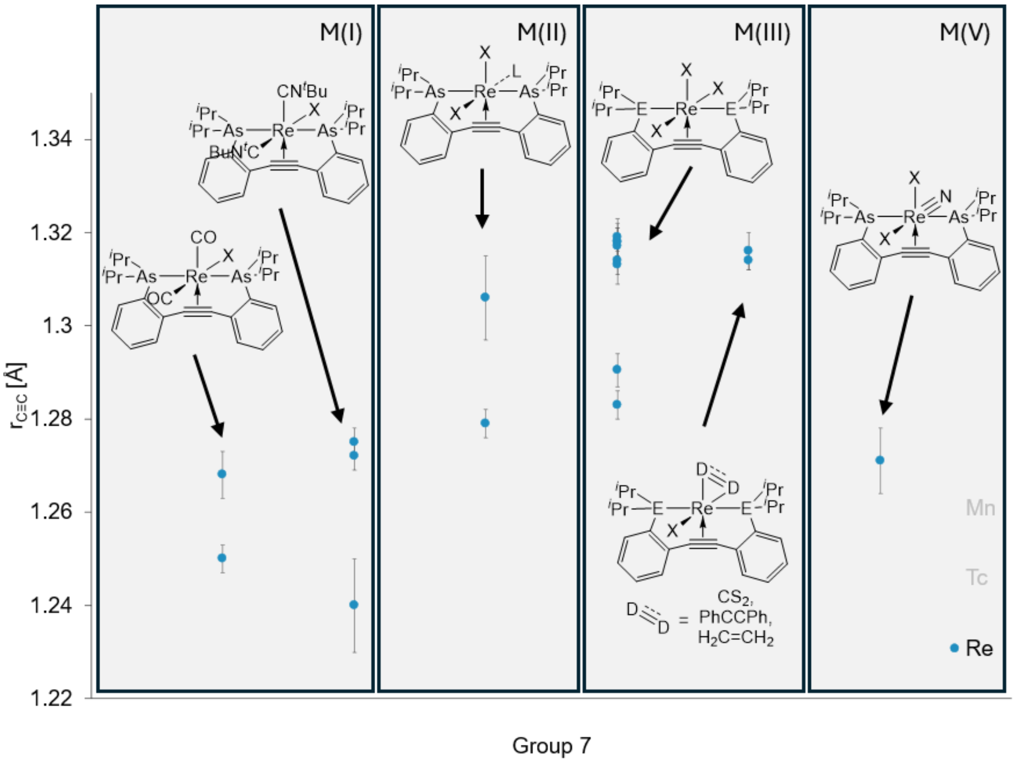

Figure S158. Close-up of group 7 (periods 4-6) d-element M-$C_{C≡C}$ distance in *E*,(*CC*),*E* (E = P, As) complexes. Error bars are provided around (averaged if multiple data were available) bond lengths as minimum and maximum encountered values ± estimated standard deviation.

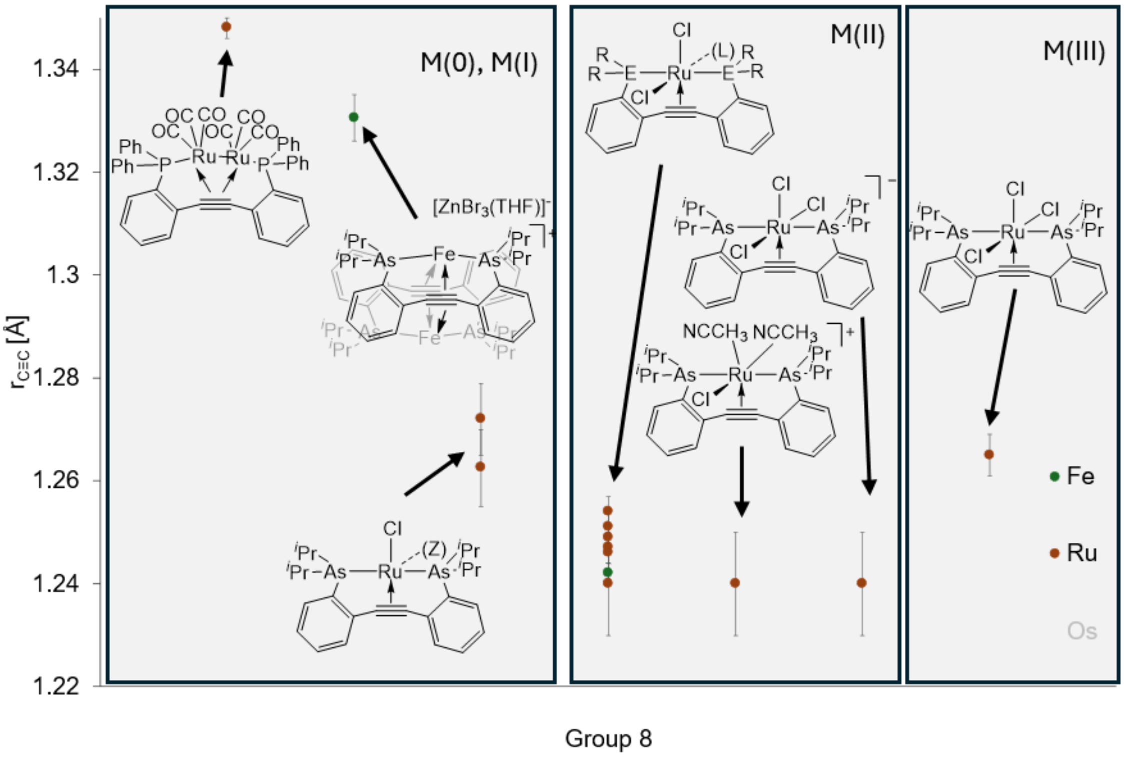

Figure S159. Close-up of group 8 (periods 4-6) d-element M-$C_{C≡C}$ distance in *E*,(*CC*),*E* (E = P, As) complexes. Error bars are provided around (averaged if multiple data were available) bond lengths as minimum and maximum encountered values ± estimated standard deviation.

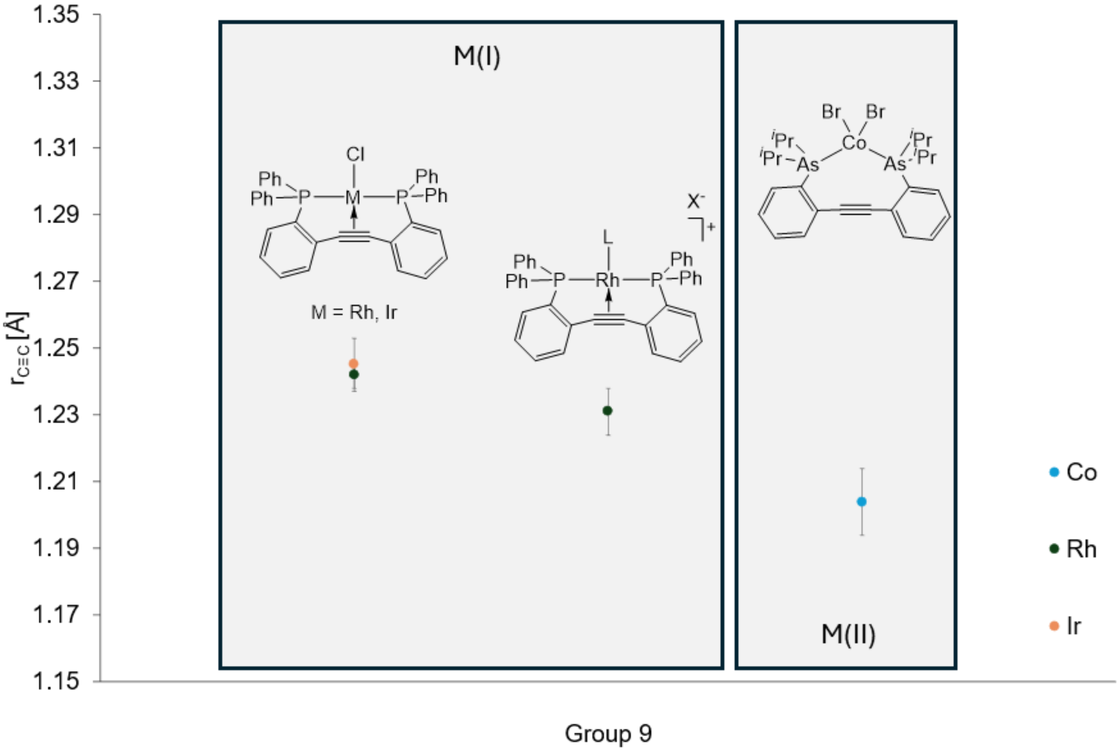

Figure S160. Close-up of group 9 (periods 4-6) d-element M-$C_{C≡C}$ distance in *E*,(*CC*),*E* (E = P, As) complexes. Error bars are provided around (averaged if multiple data were available) bond lengths as minimum and maximum encountered values ± estimated standard deviation. Note that Co complexes frequently undergo ligand cyclization.

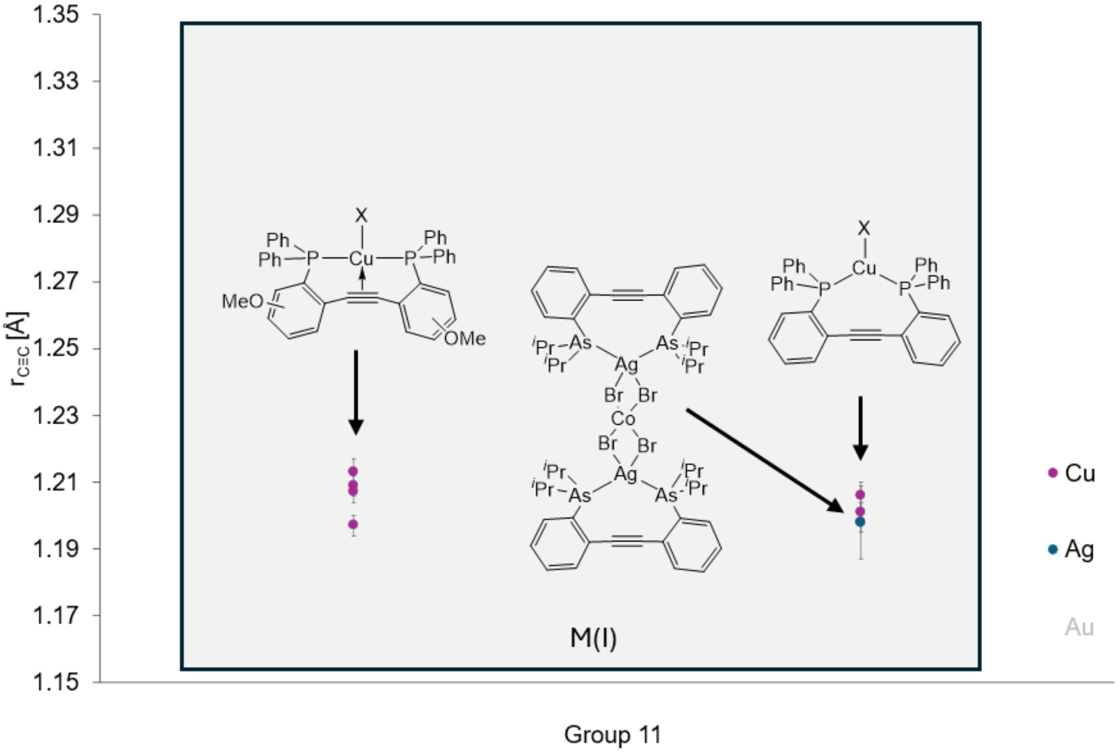

Figure S161. Close-up of group 11 (periods 4-6) d-element M-$C_{C≡C}$ distance in *E*,(*CC*),*E* (E = P, As) complexes. Error bars are provided around (averaged if multiple data were available) bond lengths as minimum and maximum encountered values ± estimated standard deviation.

S5.3 M-C$_{C≡C}$ bond lengths in structurally characterized *E*,(*CC*),*E* (E = P, As) complexes.[72-85]

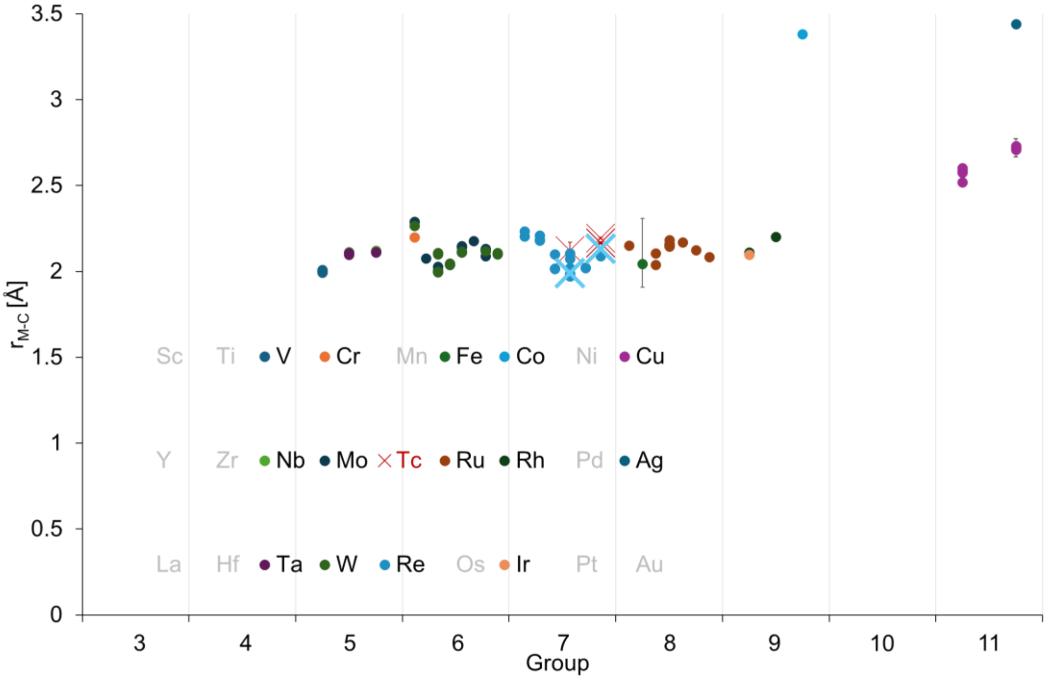

Figure S162. Overview over d-element (groups 3-11; periods 4-6) M-C$_{C≡C}$ bond lengths in structurally characterized *E*,(*CC*),*E* (E = P, As) complexes with Tc/Re complexes of this study highlighted (depicted by X). Error bars are provided around (averaged if multiple data were available) bond lengths as minimum and maximum encountered values ± estimated standard deviation. Note that Group 3, 4, 9 and 10 complexes frequently undergo ligand cyclization.

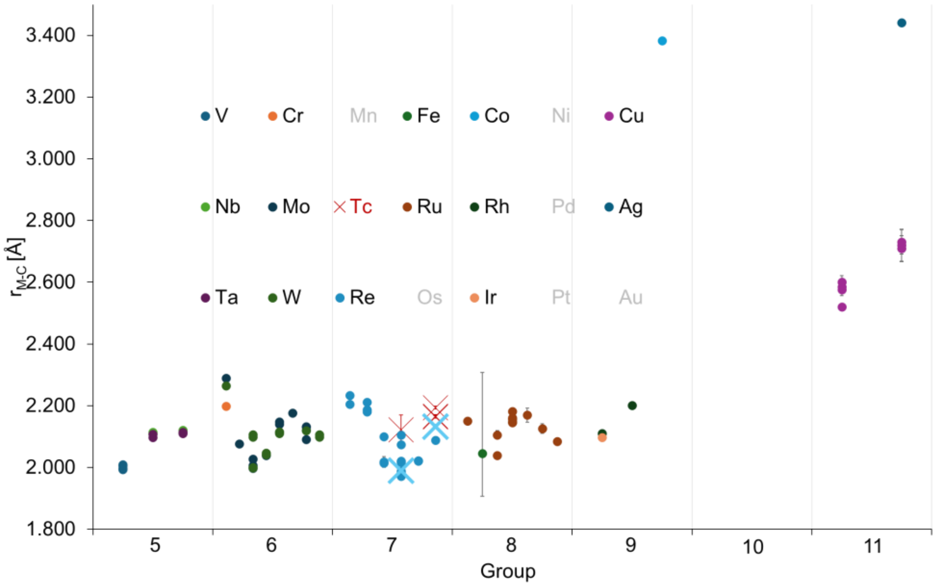

Figure S163. Close-up of group 5 to 11 (periods 4-6) d-element M-C$_{C≡C}$ distance in *E*,(*CC*),*E* (E = P, As) complexes with Tc/Re complexes of this study highlighted (depicted by X). Error bars are provided around (averaged if multiple data were available) bond lengths as minimum and maximum encountered values ± estimated standard deviation. Note that Group 3, 4, 9 and 10 complexes frequently undergo ligand cyclization.

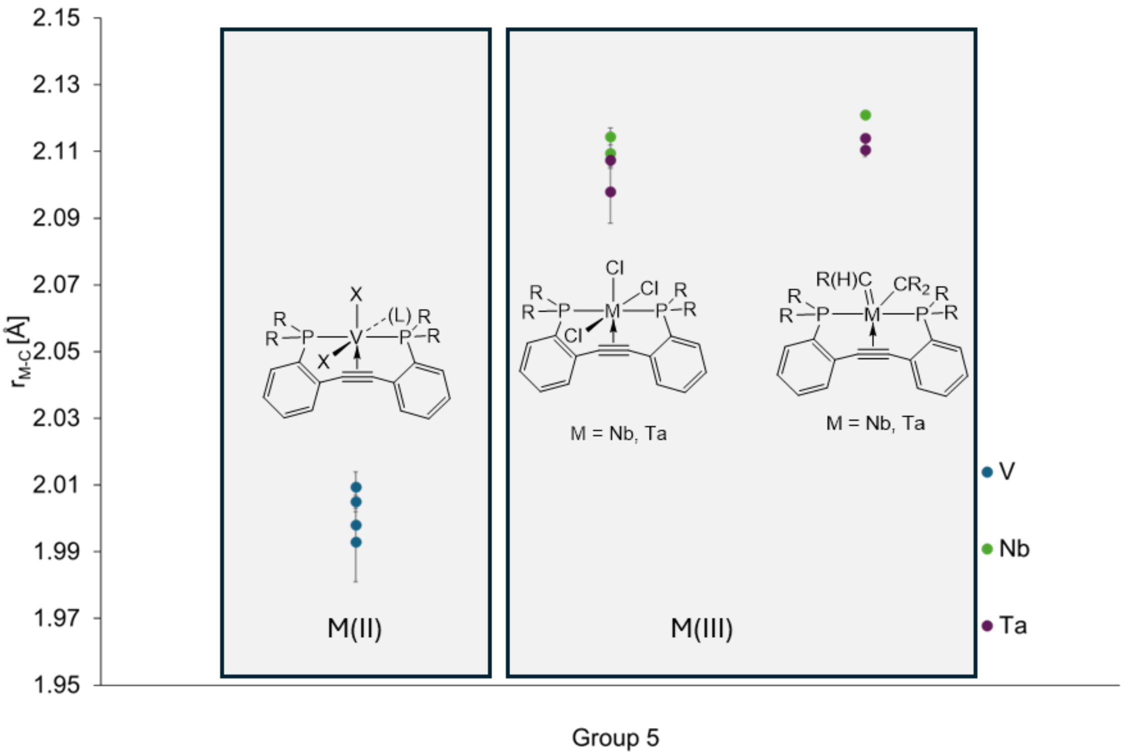

Figure S164. Close-up of group 5 (periods 4-6) d-element M-$C_{C≡C}$ distance in *E*,(*CC*),*E* (E = P, As) complexes. Error bars are provided around (averaged if multiple data were available) bond lengths as minimum and maximum encountered values ± estimated standard deviation.

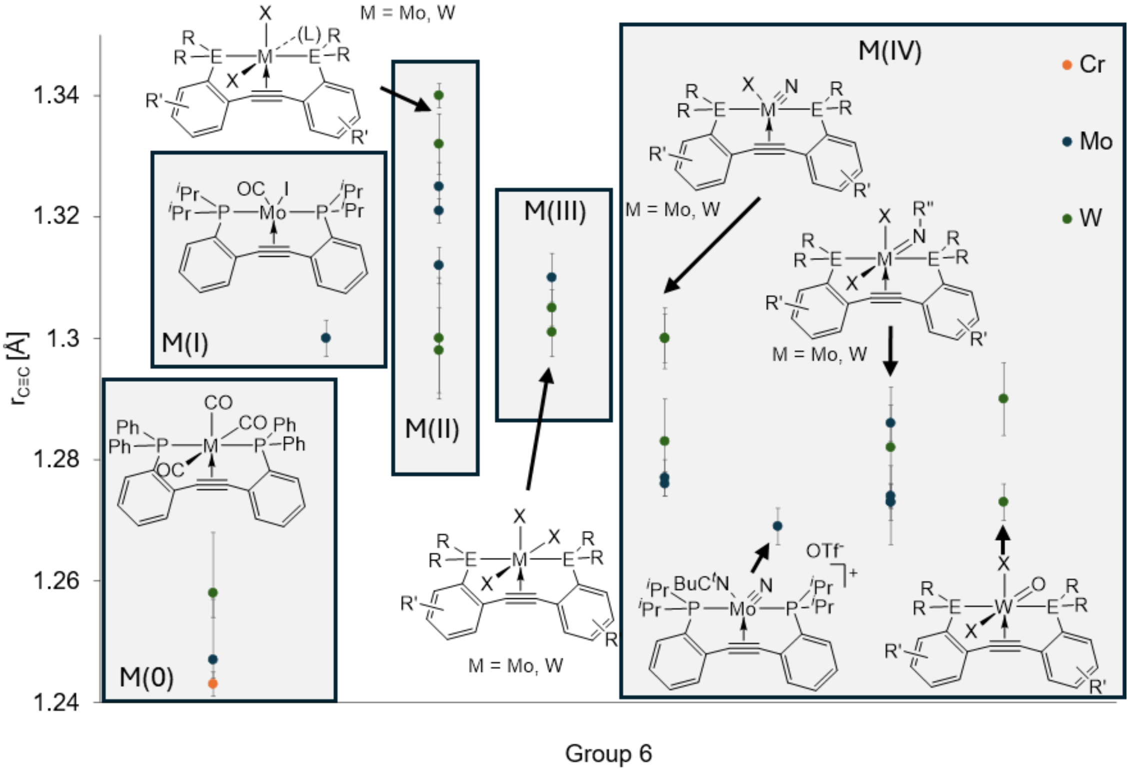

Figure S165. Close-up of group 6 (periods 4-6) d-element M-$C_{C≡C}$ distance in *E*,(*CC*),*E* (E = P, As) complexes. Error bars are provided around (averaged if multiple data were available) bond lengths as minimum and maximum encountered values ± estimated standard deviation.

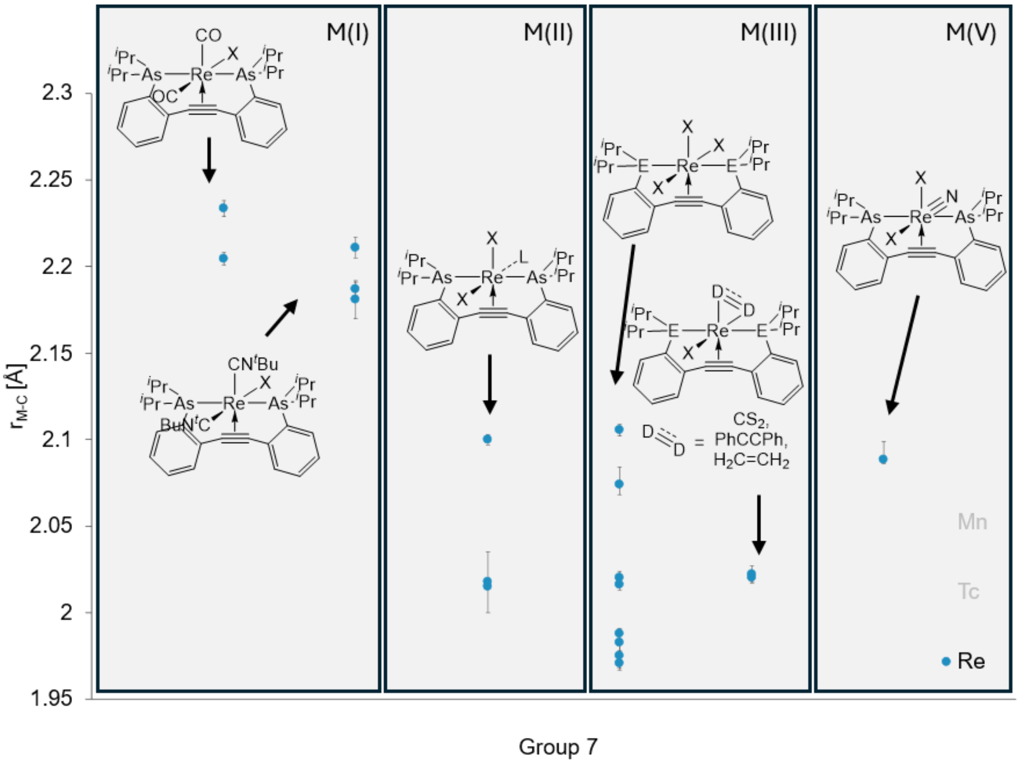

Figure S166. Close-up of group 7 (periods 4-6) d-element M-$C_{C≡C}$ distance in *E*,(*CC*),*E* (E = P, As) complexes. Error bars are provided around (averaged if multiple data were available) bond lengths as minimum and maximum encountered values ± estimated standard deviation.

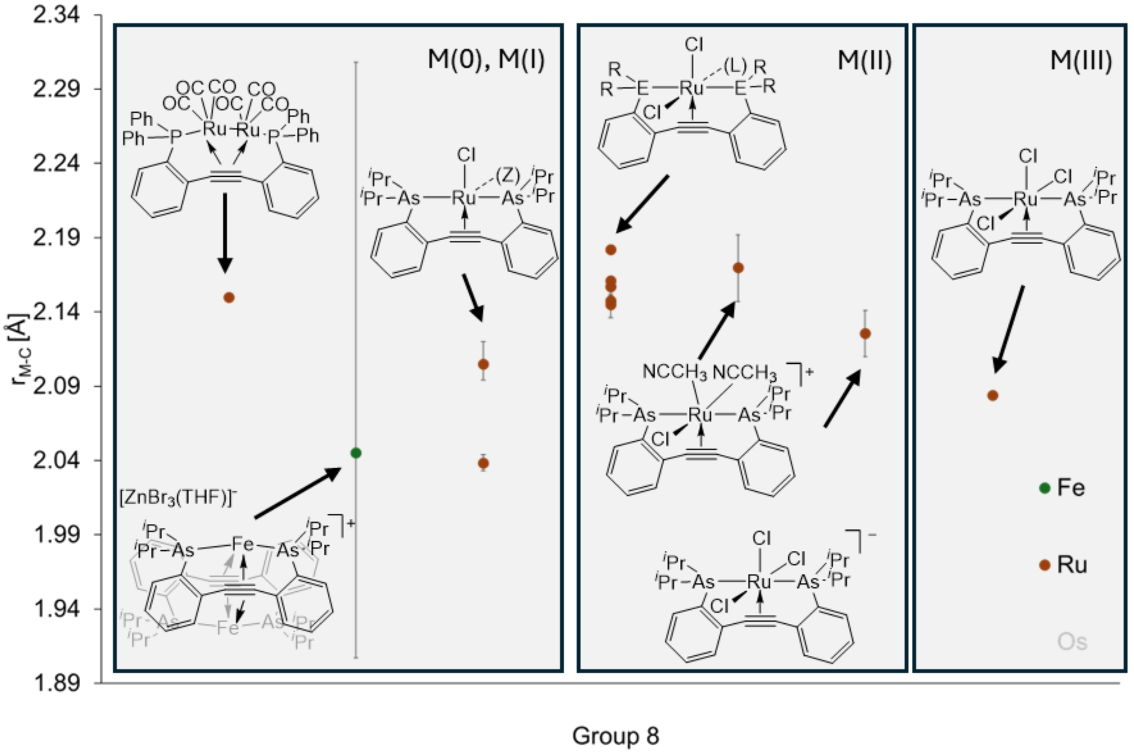

Figure S167. Close-up of group 8 (periods 4-6) d-element M-$C_{C≡C}$ distance in *E*,(*CC*),*E* (E = P, As) complexes. Error bars are provided around (averaged if multiple data were available) bond lengths as minimum and maximum encountered values ± estimated standard deviation.

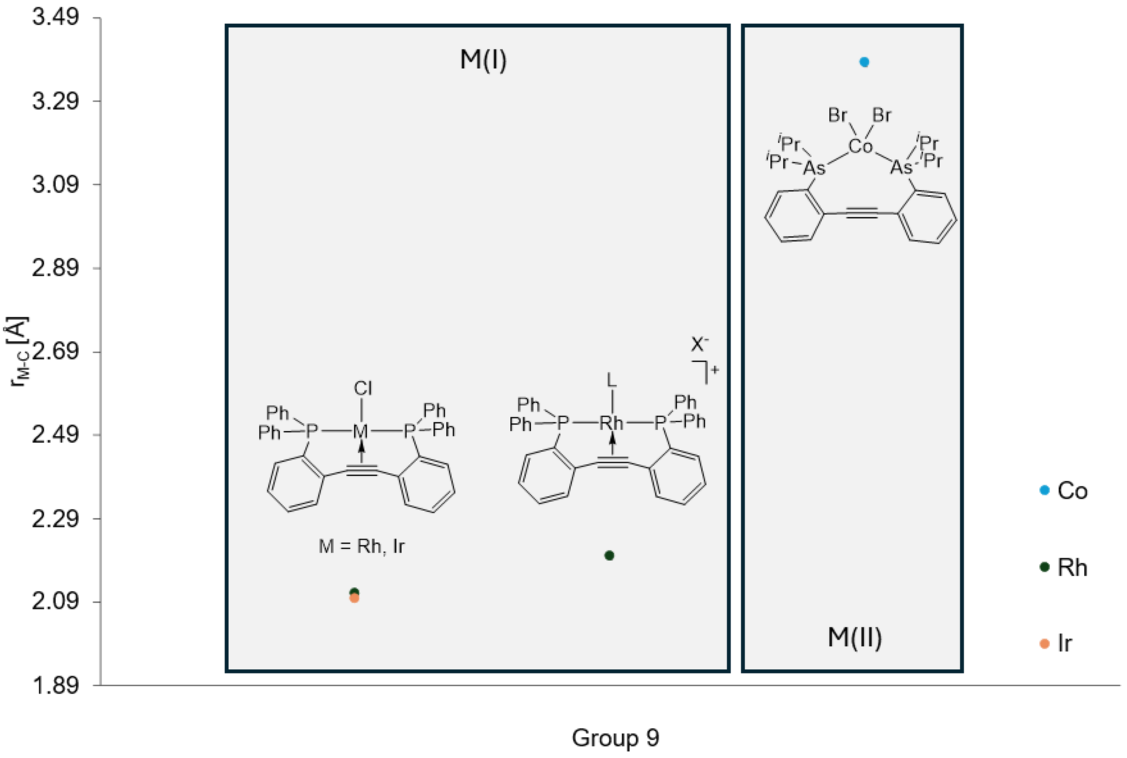

Figure S168. Close-up of group 9 (periods 4-6) d-element M-$C_{C≡C}$ distance in *E*,(*CC*),*E* (E = P, As) complexes. Error bars are provided around (averaged if multiple data were available) bond lengths as minimum and maximum encountered values ± estimated standard deviation. Note that Co complexes frequently undergo ligand cyclization.

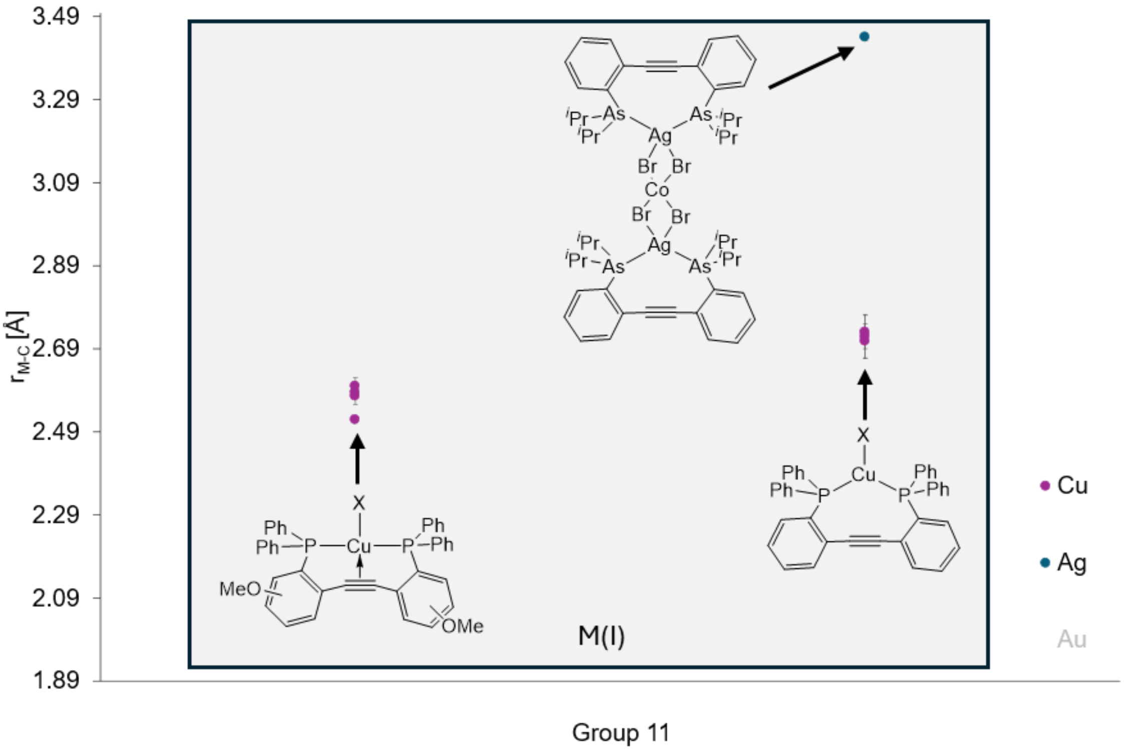

Figure S169. Close-up of group 11 (periods 4-6) d-element M-$C_{C≡C}$ distance in *E*,(*CC*),*E* (E = P, As) complexes. Error bars are provided around (averaged if multiple data were available) bond lengths as minimum and maximum encountered values ± estimated standard deviation.

S5.4 ^{13}C NMR chemical shifts in *E*,*CC*,*E* (E = P, As) complexes.[72-85]

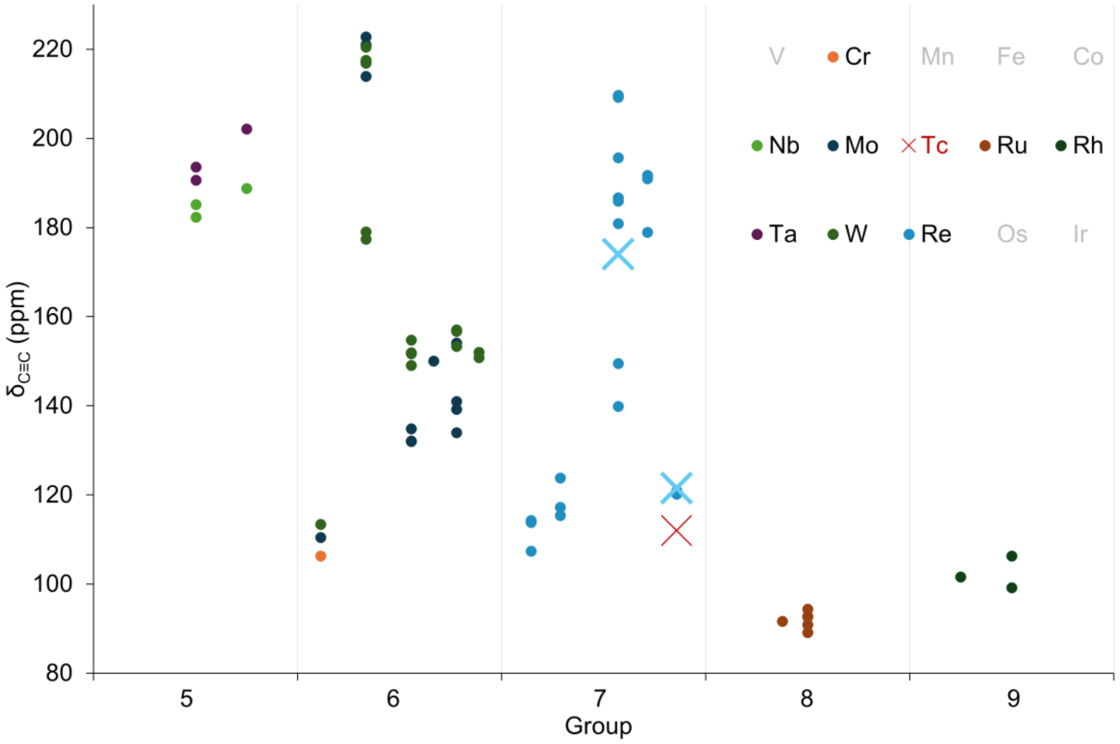

Figure S170. Overview over reported ^{13}C NMR chemical shifts for d-element (groups 5-9; periods 4-6) C≡C bond lengths in structurally characterized *E*,(*CC*),*E* (E = P, As) complexes with the Tc & Re complexes of this study highlighted (depicted by X).

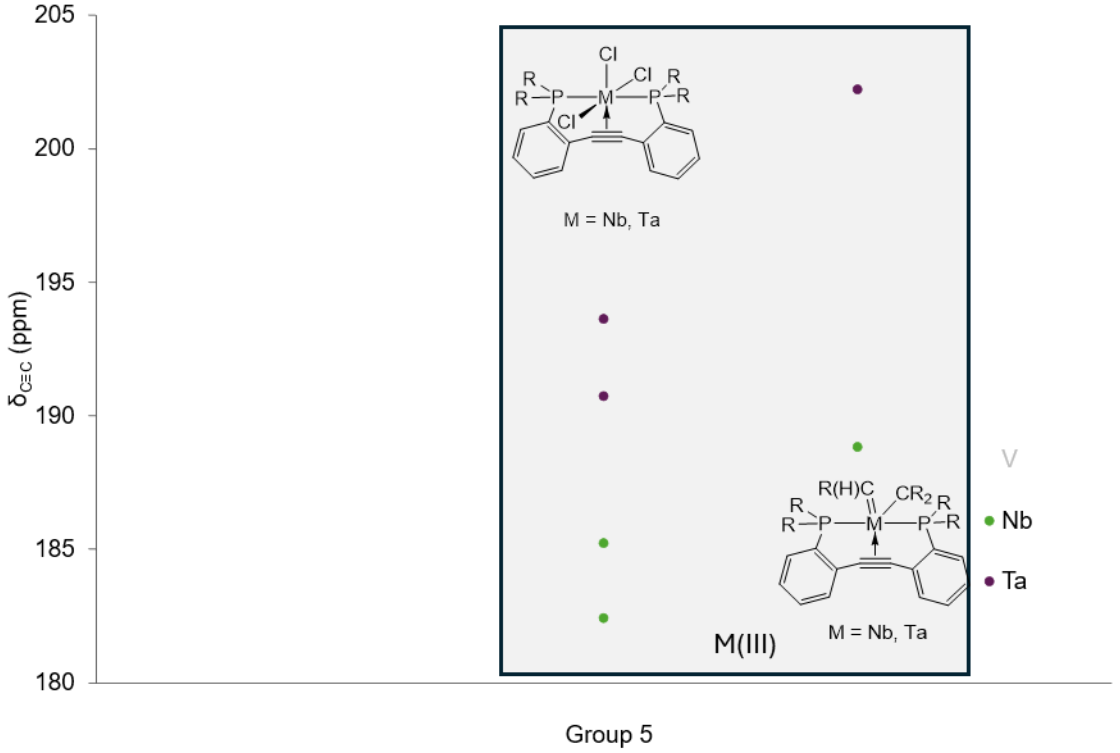

Figure S171. Close-up of reported ^{13}C NMR chemical shifts for group 5 (periods 4-6).

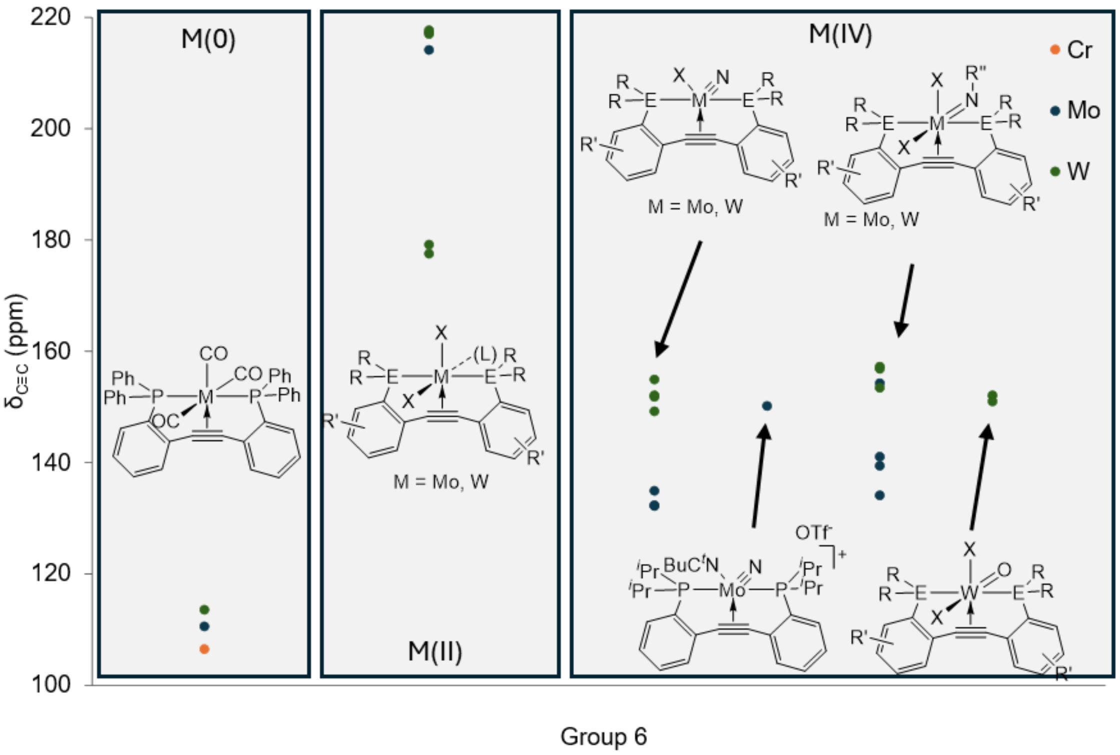

Figure S172. Close-up of reported ^{13}C NMR chemical shifts for group 6 (periods 4-6).

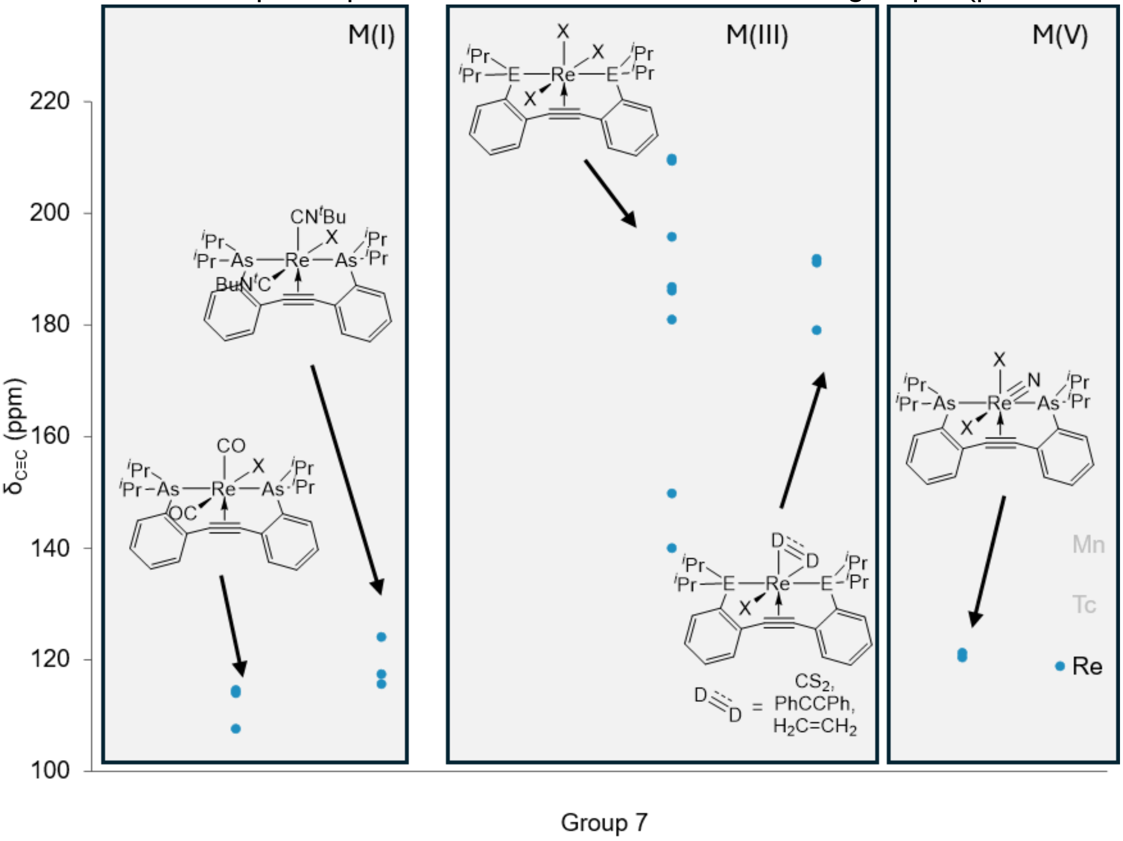

Figure S173. Close-up of reported ^{13}C NMR chemical shifts for group 7 (periods 4-6).

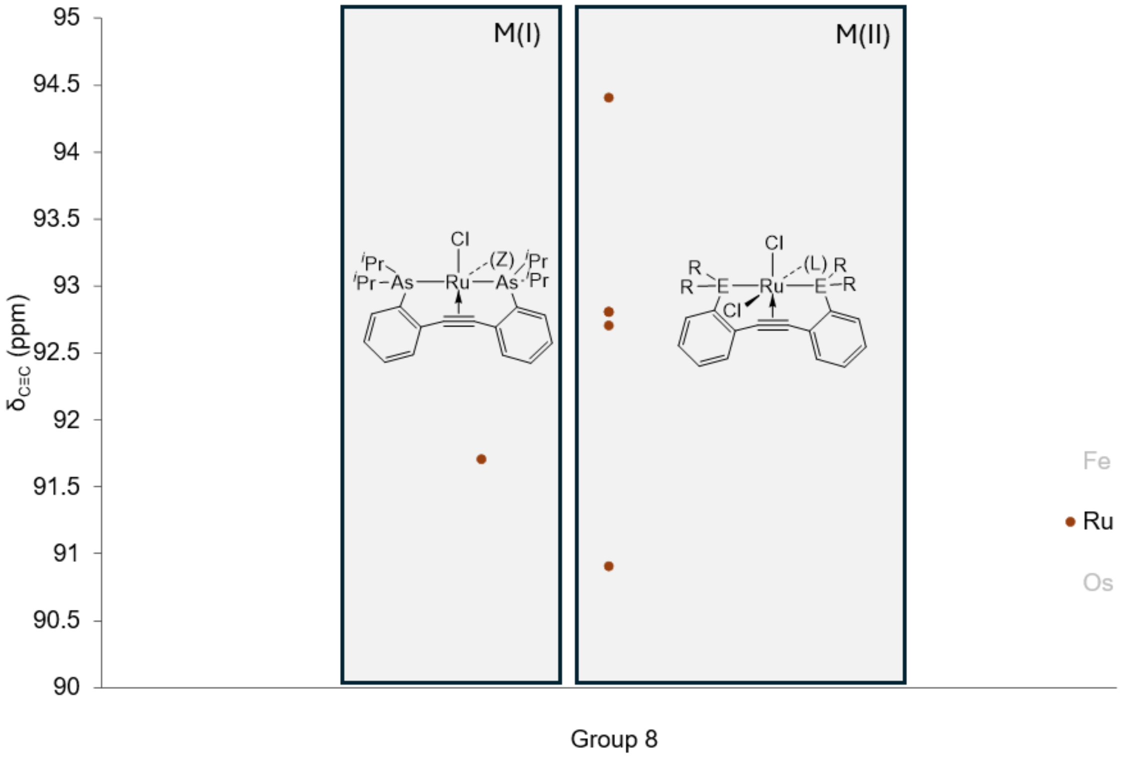

Figure S174. Close-up of reported ^{13}C NMR chemical shifts for group 8 (periods 4-6).

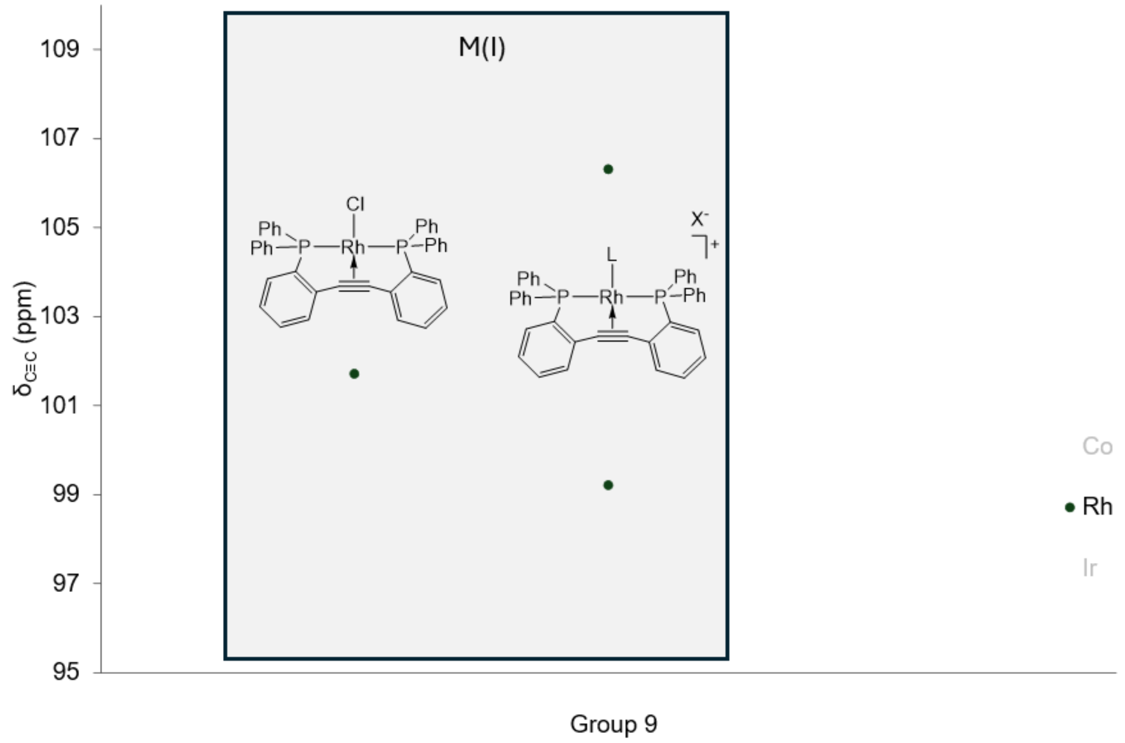

Figure S175. Close-up of reported ^{13}C NMR chemical shifts for group 9 (periods 4-6).

S5.5 $OPPh_3$ during reactions of [$Tc^{III}Cl_3(PPh_3)_2(NCCH_3)$].

Single crystals of *mer*-[$Tc^{III}Cl_3$(κ^4-*As*,*CC*,*As*-L^{iPr})]·$OPPh_3$ suitable for X-ray diffraction were obtained directly from the technetium-containing reaction mixture upon slow evaporation in air. The product cannot be separated from the phosphine oxide by-product without significant loss in yield and a complete removal of the phosphine oxide could not be achieved. Instead, quantitative yields of the technetium product can be isolated, when the reaction is performed under inert atmosphere avoiding inadvertent oxidation of the phosphine. The released PPh_3 and excess free ligand are easily removed from the product by washing of the solid residue with unpolar solvents (e.g., pentane) as confirmed by the absence of $^{31}P\{^1H\}$ resonances in the NMR spectra and the lack of P-C vibrational band patterns in the infrared spectra.

S5.6 Reactivity differences between *mer*-[MCl_3(κ^4-*As*,*CC*,*As*-L^{iPr})] (M = Tc, Re).

The proposed electronic differences between technetium and rhenium in *mer*-[MCl_3(*As*,*CC*,*As*-L^{iPr})] have consequences for the reactivity of the respective complexes. Explicitly, ligand exchange procedures that are generally successful for the (non-fluorinated) rhenium complexes, e.g., with $NaHBEt_3$ or AgOTf or reduction with coordination of neutral ligands, e.g., CO or CN^{t}Bu do not proceed for technetium and *mer*-[$Tc^{III}Cl_3$(*As*,*CC*,*As*-L^{iPr})] can be recovered unchanged from such attempts. Similarly, halide abstraction from *mer*-[Re^VNCl_2(*As*,*CC*,*As*-L^{iPr})] using AgOTf led to the exchange of both chlorido ligands under formation of *mer*-[$Re^VN(OTf)_2$(*As*,*CC*,*As*-L^{iPr})], while only a single halide exchange product was observed when reacting with Me_3Si-CN.

S5.7 Attempted preparation of [$MnCl_3$(κ^4-*As*,*CC*,*As*-L^{iPr})].

Attempted reactions using the recently developed manganese(III) starting material [$MnCl_3(OPPh_3)_2$] led to intractable decomposition already at room temperature – likely initiated by metal-induced halogenation of the triple bond and parallel reduction of Mn. Similar observations were made in toluene and chlorinated solvents.

S5.8 Attempted preparation of [$Tc(CO)_3$(κ^4-*As*,*CC*,*As*-L^{iPr})]$^+$.

Judging from the soft nature of the arsenic and alkyne moieties, reactions with the common class of *fac*-$\{Tc(CO)_3\}^+$ starting materials were initially attempted. No reaction with the alkyne unit was observed (recovery of the starting materials from toluene or THF) although minor resonances consistent with multiple As-coordinated species (likely attributed to clustering) were observed after boiling in toluene. This can be understood by a high isomerization barrier to the unfavorable *mer* orientation of the tricarbonyl unit with the desired pincer coordination as has been observed before, where the addition of halide scavengers was required.[6] Even the addition of halide scavengers instead led to the formation of a silver complex instead of the coordination to technetium in THF.

S5.9 Attempted preparation of [$TcCl_3$(κ^4-*As*,*CC*,*As*-L^{tBu})].

Initial experiments with the sterically encumbered *tert*-butyl substituted potential *As*,*CC*,*As* ligand L^{tBu} and [$Tc^{III}Cl_3(PPh_3)_2(NCCH_3)$] in boiling toluene or CH_2Cl_2 at room temperature led to cyclization of the arsine-substituted alkyne and intractable

decomposition of the technetium starting material – a similar reaction has previously been observed for phosphine-based potential *P*,*CC*,*P* ligands. Some crystals of the cyclic reaction product precipitated from the reaction mixture upon cooling and the structure was verified by X-ray diffraction.

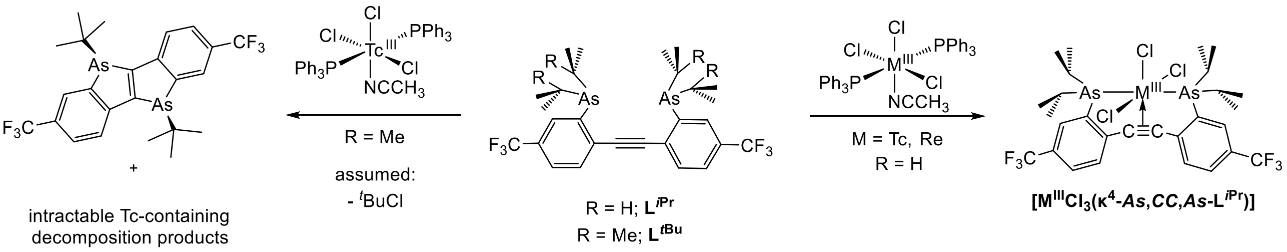

Figure S176. Reaction of L^{tBu} and L^{iPr} with isostructural technetium(III) and rhenium(III) starting materials.

S5.10 Attempted preparation of [TcN(NCS)Cl(κ^4-*As*,*CC*,*As*-L^{iPr})] & [TcN(N_3)Br(κ^4-*As*,*CC*,*As*-L^{iPr})].

The assumed initial reaction product of *mer*-[$Tc^{V}NCl_2$(κ^4-*As*,*CC*,*As*-L^{iPr})] with Me_3Si-SCN was not isolated due to the high solubility of the product. During crystallization attempts spanning some weeks, inadvertent oxidation of the arsine groups led to the precipitation of a crystalline double-salt containing the oxidized cation ($^{i}Pr_2As(OH)C_6(CF_3)H_3$-CC-$C_6H_3(CF_3)As(OH)^{i}Pr_2)^+$, [$Tc^{V}N(NCS)_4(THF)]^{2-}$ and Cl^-. Contrarily, a reaction of [$Tc^{V}NCl_2$(κ^4-*As*,*CC*,*As*-L^{iPr})] with Me_3Si-N_3 did not proceed and the starting material was recovered even after prolonged heating in toluene.

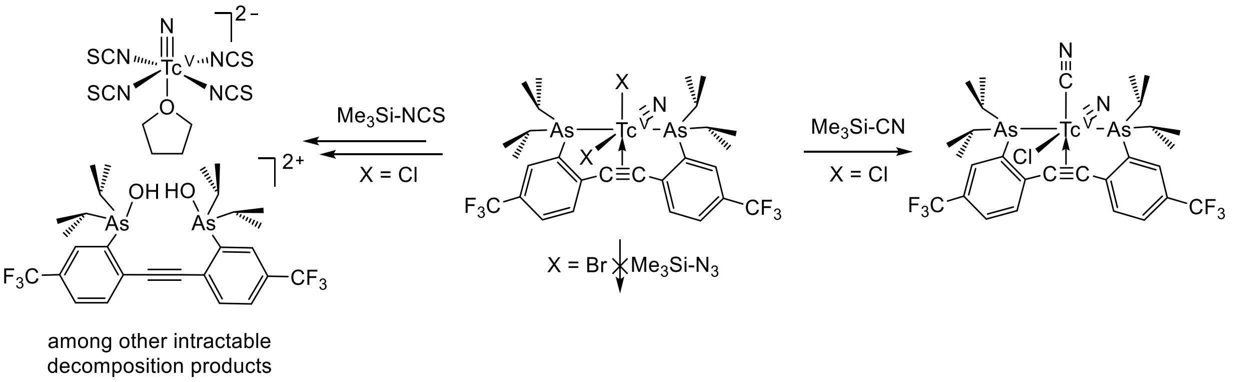

Figure S177. Reactions of *mer*-[$Tc^{V}NX_2$(κ^4-*As*,*CC*,*As*-L^{iPr})] (X = Cl, Br).

S5.11 Overview over attempted reactions of alkynes with technetium starting materials.

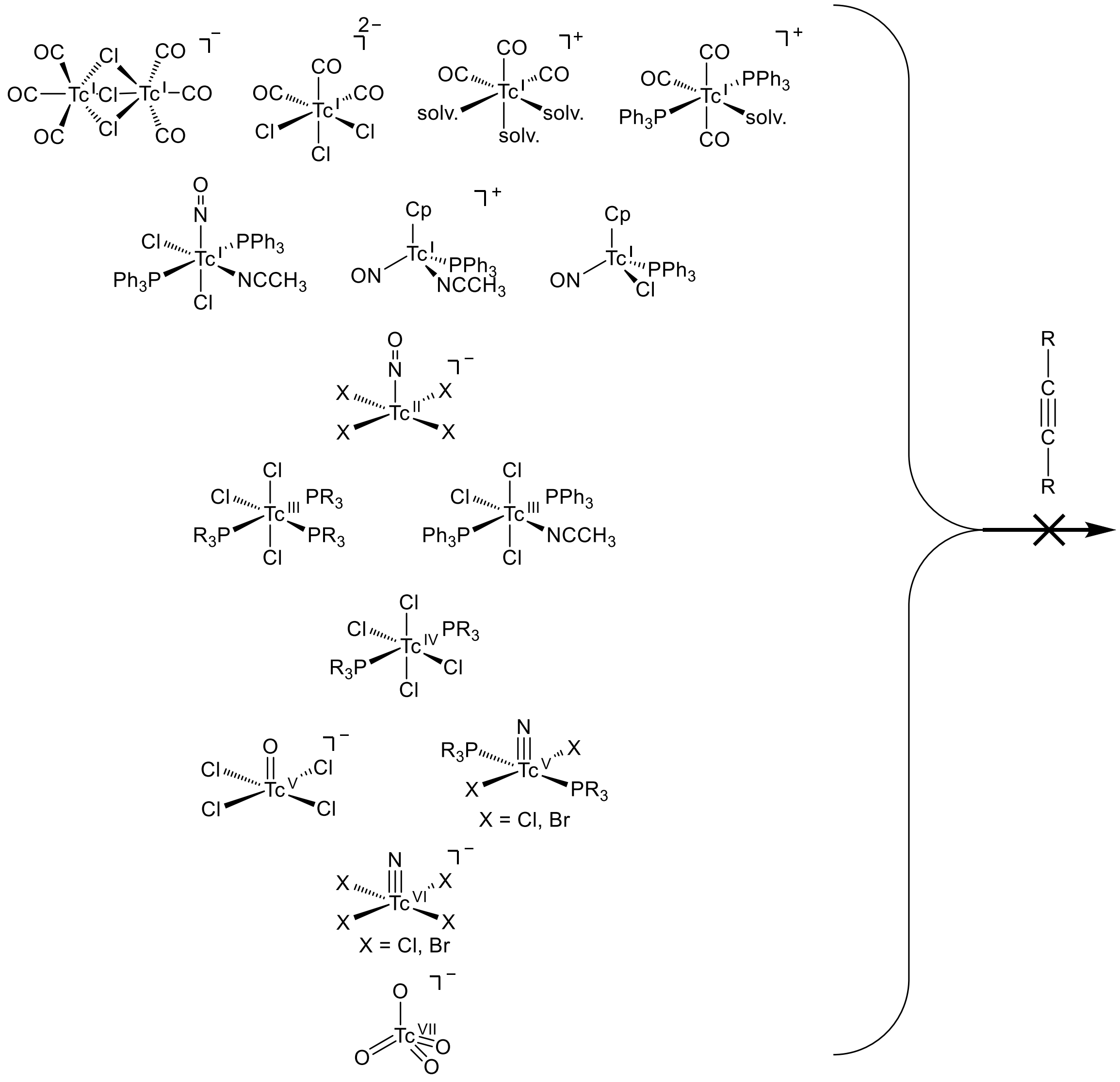

Figure S178. Selection of attempted reactions of technetium starting materials with R–C≡C–R to form complexes containing side-on coordinating η^2-R–C≡C–R ligands.[1]

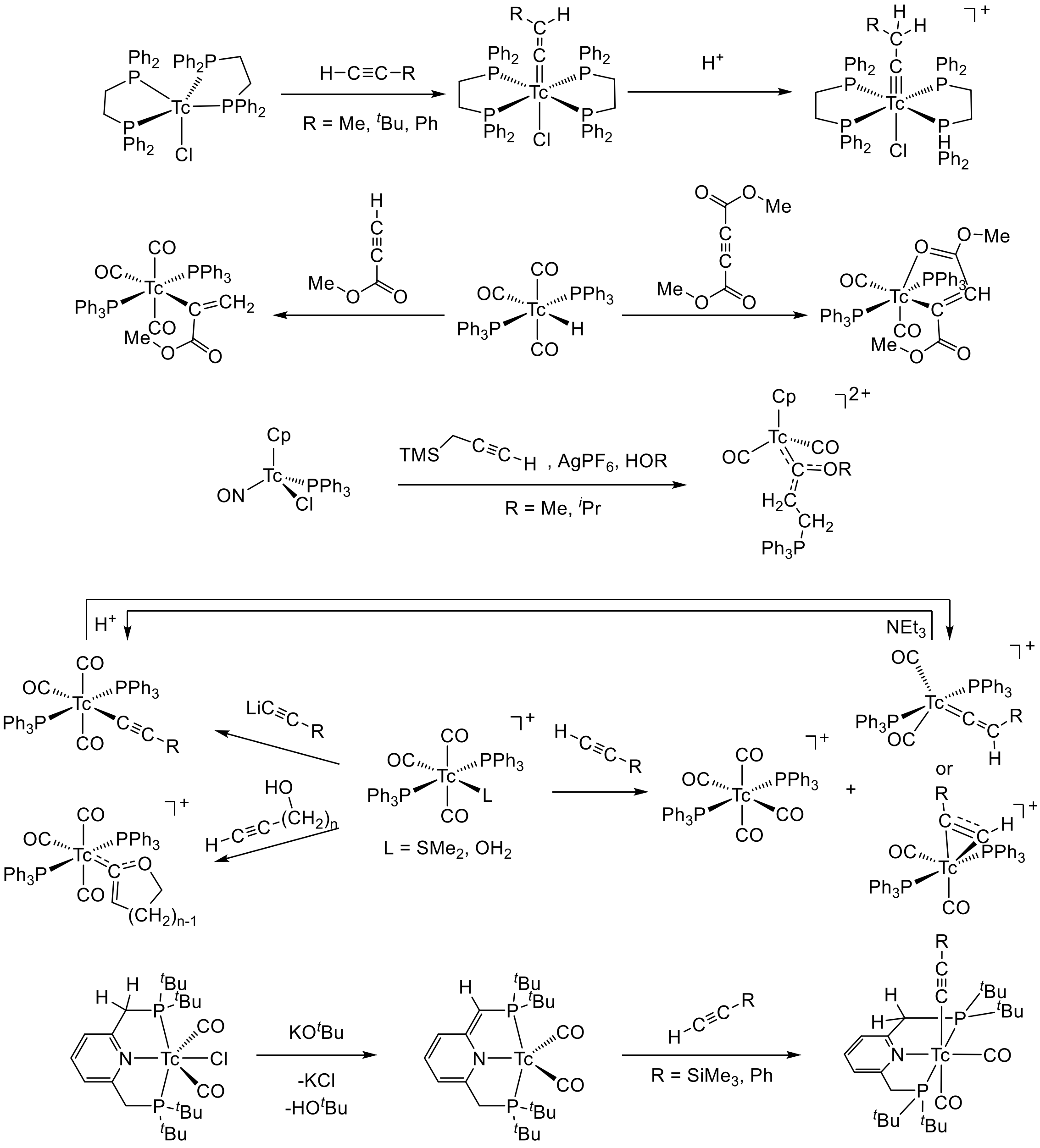

Figure S179. Reactions of technetium starting materials with R–C≡C–R.[1]

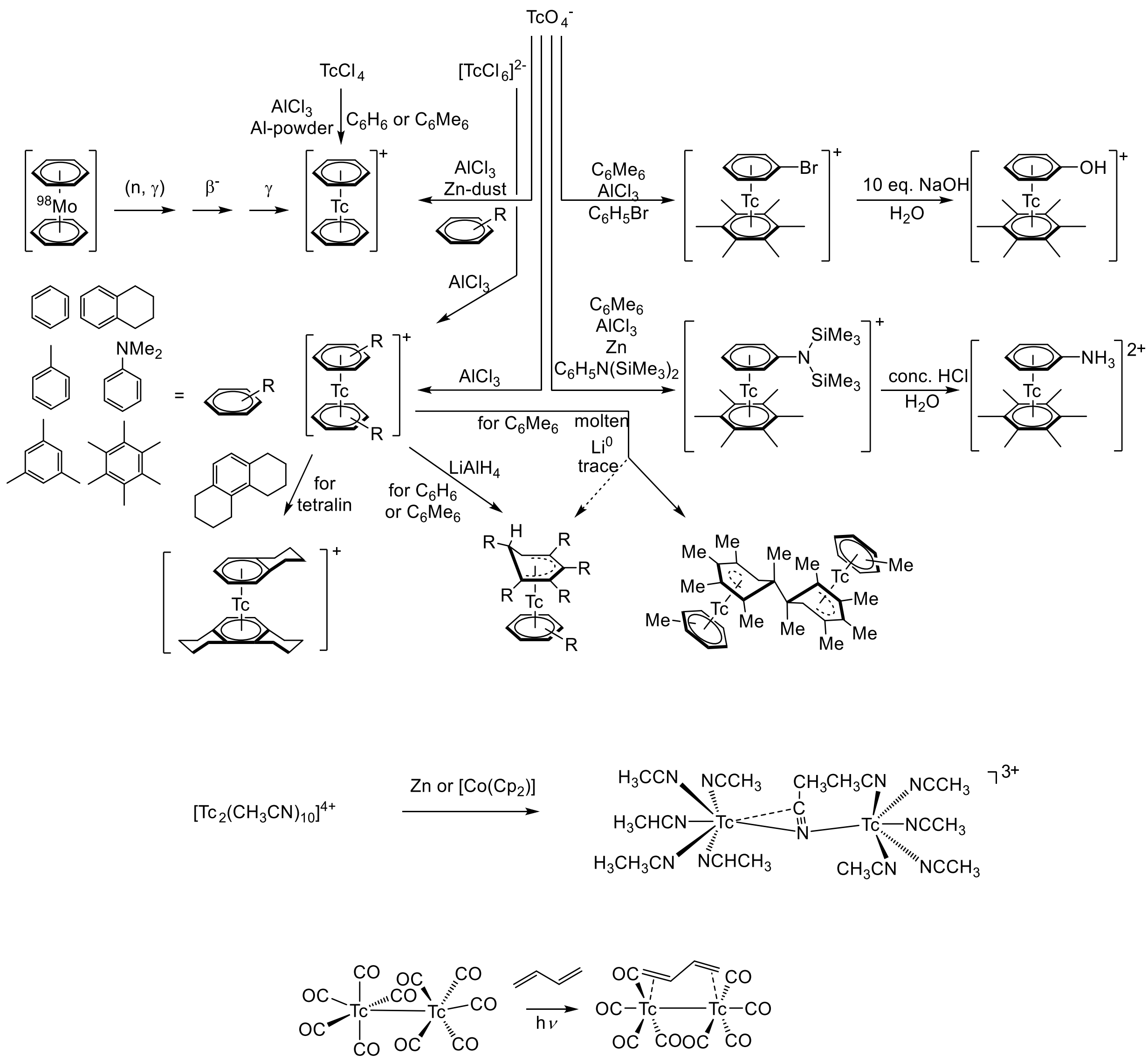

Figure S180. Non-cyclopentadienyl π-complexes of technetium.[1]

Part S6: References

[1] M. Roca Jungfer, M. L. Besmer, Structural organometallic chemistry of technetium. *Adv. Organomet. Chem.* **81**, 271–328 (2024).

[2] A. K. Burrell, J. C. Bryan, G. J. Kubas, Technetium–carbon multiple bonds: synthesis and structure of Tc(=C=CHPh)Cl(dppe)$_2$ and [Tc(=C–CH$_2$–t-Bu)Cl(dppe)$_2$]$^+$. *Organometallics* **13**, 1067–1069 (1994).

[3] A. Abdulkader, A. Hagenbach, U. Abram, [Tc(NO)Cl(Cp)(PPh$_3$)]—A technetium(I) compound with an unexpected synthetic potential. *Eur. J. Inorg. Chem.* **2021**, 3812–3818 (2021).

[4] M. Roca Jungfer, U. Abram, Unlocking air- and water-stable technetium acetylides and other organometallic complexes. *Inorg. Chem.* **61**, 7765–7779 (2022).

[5] M. J. Ernst, M. Roca Jungfer, U. Abram, Reactions of Tc^I(NO) and Tc^{V}N complexes with alkynes and alkynides. *Organometallics* **41**, 2011–2021 (2022).

[6] M. L. Besmer, H. Braband, T. Fox, B. Spingler, A. P. Sattelberger, R. Alberto, Binding small molecules to a cis-dicarbonyl ^{99}Tc^I-PNP complex via metal–ligand cooperativity. *Inorg. Chem.* **62**, 10727–10735 (2023).

[7] J. K. Felixberger, J. G. Kuchler, E. Herdtweck, R. A. Paciello, W. A. Herrmann, Alkyne coordination to organorhenium oxides with rhenium in high oxidation states. *Angew. Chem. Int. Ed.* **27**, 946–948 (1988).

[8] W. A. Herrmann, R. A. Fischer, E. Herdtweck, Alkyne complexes of rhenium in intermediate oxidation states: synthesis and molecular structure of a rhenium(V) allenylidene complex. *Angew. Chem. Int. Ed.* **26**, 12–13 (1987).

[9] J. J. Kowalczyk, A. M. Arif, J. A. Gladysz, Synthesis, structure, and reactivity of chiral rhenium alkyne complexes of the formula [(η^5-C$_5$H$_5$)Re(NO)(PPh$_3$)(RC≡CR′)]$^+$BF$_4^-$. *Organometallics* **10**, 1079–1088 (1991).

[10] A. M. Santos, C. C. Romão, F. E. Kühn, (η^2-Alkyne)methyl(dioxo)rhenium complexes as aldehyde-olefination catalysts. *J. Am. Chem. Soc.* **125**, 2414–2415 (2003).

[11] K. G. Caulton, Coordination chemistry of the manganese and rhenium fragments (C$_5$H$_5$)M(CO)$_2$. *Coord. Chem. Rev.* **38**, 1–43 (1981).

[12] J. M. Mayer, T. H. Tulip, J. C. Calabrese, E. Valencia, Associative ligand substitution reactions of low-valent rhenium-oxo compounds: crystal and molecular structures of [ReO(MeC≡CMe)$_2$L]SbF$_6$ (L = pyridine, 4,4′-dimethyl-2,2′-bipyridine). *J. Am. Chem. Soc.* **109**, 157–163 (1987).

[13] J. K. Bera, P. E. Fanwick, R. A. Walton, Decarboxylation reactions of alkyne-carboxylic acids as a route to multiply bonded [Re$_2$]$^{n+}$ μ-alkyne and μ-carbyne complexes. *Dalton Trans.* **2001**, 109–110 (2001).

[14] R. D. Adams, P. Dhull, M. Kaushal, M. D. Smith, The activation and transformations of vinyl acetate at a dirhenium carbonyl center. *J. Organomet. Chem.* **902**, 120969 (2019).

[15] D. M. Hoffman, J. C. Huffman, D. Lappas, D. A. Wierda, Alkyne reactions with rhenium(V) oxo alkyl phosphine complexes—phosphine displacement versus apparent Re–P insertion. *Organometallics* **12**, 4312–4320 (1993).

[16] M. Ganesan, K. Y. Shih, P. E. Fanwick, R. A. Walton, Complexation of the triply bonded dirhenium(II) complex Re$_2$Cl$_4$(μ-dppm)$_2$ (dppm = Ph$_2$PCH$_2$PPh$_2$) by up to three acetylene molecules. *Inorg. Chem.* **42**, 1241–1247 (2003).

[17] E. Spaltenstein, T. K. G. Erikson, S. C. Critchlow, J. M. Mayer, Low-valent rhenium-oxo alkyl and -oxo hydride complexes: the stabilizing influence of the oxo ligand. *J. Am. Chem. Soc.* **111**, 617–623 (1989).

[18] D. S. Williams, R. R. Schrock, Synthesis and reactivity of a series of analogous rhenium tris(imido), bis(imido) alkyne, and imido bis(alkyne) complexes. *Organometallics* **12**, 1148–1161 (1993).

[19] T. R. Cundari, R. R. Conry, E. Spaltenstein, S. C. Critchlow, K. A. Hall, S. K. Tahmassebi, J. M. Mayer, Rhenium-oxo-bis(acetylene) anions: structure, properties, and electronic structure. Comparison of Re–O bonding with that in other rhenium-oxo complexes. *Organometallics* **13**, 322–331 (1994).

[20] S. K. Tahmassebi, W. S. McNeil, J. M. Mayer, Hydroxide-, amide-, and sulfhydryl-rhenium(I) tris(alkyne) complexes: rearrangements to rhenium(III) bis(alkyne) oxo and nitrido compounds. *Organometallics* **16**, 5342–5353 (1997).

[21] Y. Han, C. J. Harlan, P. Stoessel, B. J. Frost, J. R. Norton, S. Miller, B. Bridgewater, Q. Xu, Rhenium oxo complexes of a chelating diyne ligand: synthesis and study of the kinetics of protonation. *Inorg. Chem.* **40**, 2942–2952 (2001).

[22] J. H. Jung, D. M. Hoffman, T. R. Lee, Synthesis, X-ray crystallographic, and reactivity studies of rhenium(V) alkyne complexes. *J. Organomet. Chem.* **599**, 112–122 (2000).

[23] H. W. Swidersky, O. Kindel, F. Weller, K. Dehnicke, Die Kristallstrukturen von $[ReCl_4(PhC{\equiv}CPh)]_2{\cdot}2CH_2Cl_2$ und von $PPh_4[ReOCl_4]$. *Z. Anorg. Allg. Chem.* **580**, 18–26 (1990).

[24] T. Weidmann, V. Weinrich, B. Wagner, C. Robl, W. Beck, Dirhenioethin $(OC)_5Re{-}C{\equiv}C{-}Re(CO)_5$ als Baustein zum Aufbau von Carbonyl-Metall-Clusterverbindungen. *Chem. Ber.* **124**, 1363–1368 (1991).

[25] S. Komiya, A. Baba, Synthesis and structure of hydrido bis(ethylene) and hydrido dinitrogen complexes of rhenium(I) having dimethylphenylphosphine ligands. *Organometallics* **10**, 3105–3110 (1991).

[26] F. W. V. Einstein, K. G. Tyers, D. Sutton, Characterization of the two-electron η^2-alkyne ligand in cyclopentadienyldicarbonyl(alkyne)rhenium complexes. X-ray structure of $Re(\eta^5\text{-}C_5H_5)(\eta^2\text{-}C_2Ph_2)(CO)_2$. *Organometallics* **4**, 489–493 (1985).

[27] R. R. Conry, J. M. Mayer, Rhenium(I) tris(acetylene) complexes: $Re(OR')(RC{\equiv}CR)_3$ and $[Re(L)(RC{\equiv}CR)_3]OTf$. *Organometallics* **12**, 3179–3186 (1993).

[28] S. K. Tahmassebi, J. M. Mayer, Synthesis and reactivity of rhenium(III) sulfido bis(acetylene) iodide complexes. *Organometallics* **14**, 1039–1043 (1995).

[29] B. Dudle, O. Blacque, H. Berke, Ethylene reactions of a $[ReH(\eta^2\text{-}BH_4)(NO)(PPh_3)_2]$ complex: reductive elimination of ethane and oxidative coupling to butadiene. *Organometallics* **31**, 1832–1839 (2012).

[30] E. Valencia, B. D. Santarsiero, S. J. Geib, A. L. Rheingold, J. M. Mayer, Synthesis and characterization of symmetrical and unsymmetrical low-valent rhenium-oxo dimers, $Re_2O_2(RC{\equiv}CR)_4$. *J. Am. Chem. Soc.* **109**, 6896–6898 (1987).

[31] C. P. Casey, T. E. Vos, J. T. Brady, R. K. Hayashi, Indenyl rhenium alkyne complexes: CO substitution via alkyne-assisted ring slippage and CO-catalyzed phosphine substitution. *Organometallics* **22**, 1183–1195 (2003).

[32] W. A. Herrmann, R. A. Fischer, W. Amslinger, E. Herdtweck, Alkin-Komplexe der Organorheniumoxide: Redox-Chemie und Nucleophilie der Oxo-Funktion von

(η^5-Pentamethylcyclopentadienyl)(η^2-diphenylethin)oxorhenium(III). *J. Organomet. Chem.* **362**, 333–343 (1989).

[33] C. R. Groom, I. J. Bruno, M. P. Lightfoot, S. C. Ward, The Cambridge Structural Database. *Acta Crystallogr. B* **72**, 171–179 (2016). (Version of record: Oct. 2025)

[34] I. J. Bruno, J. C. Cole, P. R. Edgington, M. Kessler, C. F. Macrae, P. McCabe, J. Pearson, R. Taylor, New software for searching the Cambridge Structural Database and visualising crystal structures. *Acta Crystallogr. B* **58**, 389–397 (2002).

[35] B. Kanellakopulos, B. Nuber, K. Raptis, M. L. Ziegler, Darstellung und Charakterisierung eines Technetium-Butadien-Komplexes; Röntgenstrukturanalyse von $[Tc_2(CO)_8][\mu\text{-}C_4H_6]$. *Z. Naturforsch. B* **46**, 55–59 (1991).

[36] F. A. Cotton, S. C. Haefner, A. P. Sattelberger, Metal–metal multiply-bonded complexes of technetium. 6. A μ,η^1,η^2-CH_3CN complex prepared via reductive cleavage of the electron-rich Tc≡Tc triple bond in decakis-acetonitrile ditechnetium tetrafluoroborate. *Inorg. Chim. Acta* **266**, 55–63 (1997).

[37] U. Abram, M. Roca Jungfer, Cyclopentadienyl complexes of technetium. *Molecules* **30**, 4813 (2025).

[38] M. Benz, H. Braband, P. Schmutz, J. Halter, R. Alberto, From Tc^{VII} to Tc^{I}: facile syntheses of bis-arene complexes $[^{99m}Tc(arene)_2]^+$ from pertechnetate. *Chem. Sci.* **6**, 165–169 (2015).

[39] J. Cook, A. Davison, W. M. Davis, A. G. Jones, Insertion chemistry of $HTc(CO)_3(PPh_3)_2$. *Organometallics* **14**, 650–655 (1995).

[40] I. Zolle, Eds U. Heilmann, W. McHugh, *Technetium-99m Pharmaceuticals. Preparation and Quality Control in Nuclear Medicine* (Springer, Heidelberg, DE, 2007).

[41] A. T. Taylor, M. Lipowska, L. Hansen, E. Malveaux, L. G. Marzilli, ^{99m}Tc-MAEC complexes: new renal radiopharmaceuticals combining characteristics of ^{99m}Tc-MAG3 and ^{99m}Tc-EC. *J. Nucl. Med.* **45**, 885–886 (2004).

[42] R. Alberto, R. Schibli, A. Egli, A. P. Schubiger, U. Abram, T. A. Kaden, A novel organometallic aqua complex of technetium for the labeling of biomolecules: synthesis of $[^{99m}Tc(OH_2)_3(CO)_3]^+$ from $[^{99m}TcO_4]^-$ in aqueous solution and its reaction with a bifunctional ligand. *J. Am. Chem. Soc.* **120**, 7987–7988 (1998).

[43] M. Wenzel, Tc-99m labelling of cymantrene analogues with different substituents: a new approach to Tc-99m radiodiagnostics. *J. Labelled Compd. Radiopharm.* **31**, 641–650 (1992).

[44] M. Wenzel, M. Saidi, Brain affinity of ^{99m}Tc-labelled esters of cytectrene carbonic acid. *J. Labelled Compd. Radiopharm.* **33**, 77–80 (1992).

[45] M. Wenzel, C. Klinge, M. Saidi, Tc-99m- and deuterium-marked, brain-affine radiodiagnostics: comparison of HMPAO with cytectrene derivatives of cyclic amines. *J. Labelled Compd. Radiopharm.* **33**, 1030–1051 (1993).

[46] D. Kuntschke, M. Wenzel, P. Schulze, New ^{99m}Tc-cytectrene amine compounds as specific brain imaging agents. *J. Labelled Compd. Radiopharm.* **36**, 193–203 (1994).

[47] T. W. Spradau, J. A. Katzenellenbogen, Protein and peptide labeling with (cyclopentadienyl)tricarbonyl rhenium and technetium. *Bioconjugate Chem.* **9**, 765–772 (1998).

[48] T. W. Spradau, W. B. Edwards, C. J. Anderson, M. J. Welch, J. A. Katzenellenbogen, Synthesis and biological evaluation of Tc-99m-cyclopentadienyltricarbonyltechnetium-labeled octreotide. *Nucl. Med. Biol.* **26**, 1–7 (1999).

[49] J. Wald, R. Alberto, K. Ortner, L. A. Candreia, Aqueous one-pot synthesis of derivatized cyclopentadienyl–tricarbonyl complexes of ^{99m}Tc with an in situ CO source: application to a serotonergic receptor ligand. *Angew. Chem. Int. Ed.* **40**, 3062–3066 (2001).

[50] J. Bernard, K. Ortner, B. Spingler, H. J. Pietzsch, R. Alberto, Aqueous synthesis of derivatized cyclopentadienyl complexes of technetium and rhenium directed toward radiopharmaceutical application. *Inorg. Chem.* **42**, 1014–1022 (2003).

[51] C. L. Ferreira, C. B. Ewart, S. R. Bayly, B. O. Patrick, J. Steele, M. J. Adam, C. Orvig, Glucosamine conjugates of tricarbonylcyclopentadienyl rhenium(I) and technetium(I) cores. *Inorg. Chem.* **45**, 6979–6987 (2006).

[52] M. L. Bowen, Z.-F. Chen, A. M. Roos, R. Misri, U. Häfeli, M. J. Adam, C. Orvig, Long-chain rhenium and technetium glucosamine conjugates. *Dalton Trans.* **2009**, 9228–9236 (2009).

[53] T. Uehara, T. Uemura, S. Hirabayashi, S. Adachi, K. Odaka, H. Akizawa, Y. Magata, Z. Irie, Y. Arano, Technetium-99m-labeled long-chain fatty acid analogues metabolized by β-oxidation in the heart. *J. Med. Chem.* **50**, 543–549 (2007).

[54] H. W. Peindy N'Dongo, P. D. Raposinho, C. Fernandes, I. Santos, D. Can, P. Schmutz, B. Spingler, R. Alberto, Preparation and biological evaluation of cyclopentadienyl-based ^{99m}Tc-complexes [(Cp-R)^{99m}Tc(CO)$_3$] mimicking benzamides for malignant melanoma targeting. *Nucl. Med. Biol.* **37**, 255–264 (2010).

[55] N. Malek-Saied, R. El Aissi, S. Ladeira, E. Benoist, Synthesis and biological evaluation of a novel ^{99m}Tc-cyclopentadienyltricarbonyl technetium complex as a new potential brain perfusion imaging agent. *Appl. Organomet. Chem.* **25**, 680–686 (2011).

[56] Z. Li, M. Cui, J. Dai, X. Wang, P. Yu, Y. Yang, J. Jia, H. Fu, M. Ono, H. Jia, H. Saji, L. Liu, Novel cyclopentadienyl tricarbonyl complexes of ^{99m}Tc mimicking chalcone as potential SPECT imaging probes for β-amyloid plaques in brain. *J. Med. Chem.* **56**, 471–482 (2013).

[57] T. Dallagi, M. Saidi, A. Vessières, M. Huché, G. Jaouen, S. Top, Synthesis and antiproliferative evaluation of ferrocenyl and cymantrenyl triarylbutene on breast cancer cells. Biodistribution study of the corresponding technetium-99m tamoxifen conjugate. *J. Organomet. Chem.* **734**, 69–77 (2013).

[58] H. Zeng, H. Zhang, Synthesis and biological evaluation of fatty acid conjugates bearing cyclopentadienyl donors incorporated into [^{99m}Tc/Re(CO)$_3$] for myocardial imaging. *Eur. J. Med. Chem.* **72**, 10–17 (2014).

[59] S. Chen, Y. Zhang, X. Li, H. Jia, J. Lu, Evaluation of ^{99m}Tc cyclopentadienyl tricarbonyl triphenylphosphonium cation for multidrug resistance. *Bioorg. Med. Chem. Lett.* **27**, 3551–3554 (2017).

[60] X. Li, S. Chen, Z. Liu, Z. Zhao, J. Lu, Syntheses and evaluations of methoxy-modified ^{99m}Tc-labeled triphenylphosphonium cations: potential radiometallic probes for multidrug resistance detection. *J. Organomet. Chem.* **871**, 28–35 (2018).

[61] M. Sagnou, B. Mavroidi, A. Shegani, M. Paravatou-Petsotas, C. Raptopoulou, V. Psycharis, J. Pirmettis, M. S. Papadopoulos, M. Pelecanou, Remarkable brain penetration of cyclopentadienyl M(CO)$_3^+$ (M = ^{99m}Tc, Re) derivatives of benzothiazole and benzimidazole: diagnostic SPECT and therapeutic potential for Alzheimer's disease. *J. Med. Chem.* **62**, 2638–2650 (2019).

[62] H. Su, T. Chu, Synthesis and bioevaluation of cyclopentadienyl tricarbonyl technetium-99m 2-nitroimidazole derivatives for tumor hypoxia imaging. *Bioorg. Med. Chem. Lett.* **60**, 128583 (2022).

[63] R. Lengacher, S. Ott, O. Blacque, H. Braband, R. Alberto, A multi-functional tool: cyclopentadienyl Re and ^{99m}Tc complex synthesis on highly functionalised arenes. *J. Organomet. Chem.* **962**, 122281 (2022).

[64] Y. Liu, B. Spingler, P. Schmutz, R. Alberto, Metal-mediated retro-Diels–Alder of dicyclopentadiene derivatives: a convenient synthesis of [(Cp-R)M(CO)$_3$] (M = ^{99m}Tc, Re) complexes. *J. Am. Chem. Soc.* **130**, 1554–1555 (2008).

[65] H. W. Peindy N'Dongo, D. Can, B. Spingler, P. Schmutz, P. Raposinho, I. Santos, R. Alberto, Aqueous syntheses of [(Cp-R)M(CO)$_3$] (M = Mn, ^{99m}Tc, Re) complexes with bioactive functionalities. *J. Organomet. Chem.* **694**, 981–987 (2009).

[66] D. Can, H. W. Peindy N'Dongo, B. Spingler, P. Schmutz, P. Raposinho, I. Santos, R. Alberto, The [(Cp)M(CO)$_3$] (M = Re, ^{99m}Tc) building block for imaging agents and bioinorganic probes: perspectives and limitations. *Chem. Biodiv.* **9**, 1849–1866 (2012).

[67] D. Can, P. Schmutz, S. Sulieman, B. Spingler, R. Alberto, [(Cp-R)M(CO)$_3$] (M = Re or ^{99m}Tc) conjugates for theranostic receptor targeting. *Chimia* **67**, 267–270 (2013).

[68] Q. Nadeem, D. Can, Y. Shen, M. Felber, Z. Mahmood, R. Alberto, Synthesis of tripeptide-derivatized cyclopentadienyl complexes of technetium and rhenium as radiopharmaceutical probes. *Org. Biomol. Chem.* **12**, 1966–1974 (2014).

[69] S. Ursillo, D. Can, H. W. Peindy N'Dongo, P. Schmutz, B. Spingler, R. Alberto, Cyclopentadienyl chemistry in water: synthesis and properties of bifunctionalized [(η^5-C$_5$H$_3${COOR}$_2$)M(CO)$_3$] (M = Re and ^{99m}Tc) complexes. *Organometallics* **33**, 6945–6952 (2014).

[70] Frei, B. Spingler, R. Alberto, Multifunctional cyclopentadienes as a scaffold for combinatorial bioorganometallics in [(η^5-C$_5$H$_2$R$_1$R$_2$R$_3$)M(CO)$_3$] (M = Re, ^{99m}Tc) piano-stool complexes. *Chem. Eur. J.* **24**, 10156–10164 (2018).

[71] A. Frei, E. Fischer, B. C. Childs, J. P. Holland, R. Alberto, Two is better than one: difunctional high-affinity PSMA probes based on a [CpM(CO)$_3$] (M = Re/^{99m}Tc) scaffold. *Dalton Trans.* **48**, 14600–14605 (2019).

[72] L. Eberle, S. Lindenthal, J. Ballmann, To split or not to split: [AsCCAs]-coordinated Mo, W, and Re complexes and their reactivity toward molecular dinitrogen. *Inorg. Chem.* **63**, 3682–3691 (2024).

[73] L. Eberle, F. Kreis, C. A. M. Stein, J.-M. Mörsdorf, J. Ballmann, [AsCCAs]-coordinated rhenium hydrides and their reactivities toward unsaturated hydrocarbons, heterocumulenes, and CO_2. *Inorg. Chem.* **62**, 8635–8646 (2023).

[74] H. K. Wagner, H. Wadepohl, J. Ballmann, Molybdenum-mediated N_2 splitting and functionalization in the presence of a coordinated alkyne. *Angew. Chem. Int. Ed.* **60**, 25804–25808 (2021).

[75] K. Okamoto, Y. Omoto, H. Sano, K. Ohe, Alkyne-coordinating tridentate ligands: structural properties and reactivity of their rhodium complexes. *Dalton Trans.* **41**, 10926–10929 (2012).

[76] K. Sasakura, K. Okamoto, K. Ohe, Incorporation of monatomic cations onto an Ir–Ir bond in a dimeric iridium(II) complex having a 1,3-diene-1,4-diyl backbone. *Organometallics* **37**, 2319–2324 (2018).

[77] P. Federmann, T. Richter, H. Wadepohl, J. Ballmann, Synthesis and reactivity of [PCCP]-coordinated group 5 alkyl and alkylidene complexes featuring a metallacyclopropene backbone. *Organometallics* **38**, 4307–4318 (2019).

[78] N. Liu, L. Liu, X.-X. Zhong, F.-B. Li, F.-Y. Li, H.-M. Qin, Ethynyl π-coordinated and non-coordinated mononuclear Cu(I) halide diphosphine complexes: synthesis and photophysical studies. *New J. Chem.* **46**, 3236–3247 (2022).

[79] D. T. Gordon, R. L. Belaunzaran, R. E. Chafin, G. O. A. Yap, M. M. Deegan, Synthesis of an isostructural series of group 6 complexes supported by an alkyne-based pincer ligand. *Organometallics* **44**, 1296–1303 (2025).

[80] B. Rudin, C. A. M. Stein, J. Ballmann, Tolane-based phosphino- and arsino-ruthenium complexes in three different oxidation states: Ru(I), Ru(II), and Ru(III). *Chem. Eur. J.* **31**, e202404546 (2025).

[81] P. Federmann, H. K. Wagner, P. W. Antoni, J.-M. Mörsdorf, J. L. Pérez Lustres, H. Wadepohl, M. Motzkus, J. Ballmann, P-protected diphosphadibenzo[a,e]pentalenes and their mono- and dicationic P-bridged ladder stilbenes. *Org. Lett.* **21**, 2033–2038 (2019).

[82] I. R. Fejes, G. P. A. Yap, M. M. Deegan, Ruthenium coordination chemistry of a bisphosphine alkyne pincer ligand. *Eur. J. Inorg. Chem.* **2025**, e202500457 (2025).

[83] B. Rudin, L. Eberle, J. Ballmann, 2,2′-Diphosphino- and 2,2′-diarsenotolanes and their Fe-, Co-, and Ni-complexes: pnictogen-dependent cyclization tendencies and metal-dependent stability and reactivity patterns. *Organometallics* **42**, 933–943 (2023).

[84] L. Eberle, J. Ballmann, (Hetero)anilines from dinitrogen via nucleophilic aromatic substitution at tungsten nitrido complexes. *J. Am. Chem. Soc.* **147**, 25123–25128 (2025).

[85] L. Eberle, J. Ballmann, Synthesis of collidine from dinitrogen via a tungsten nitride. *J. Am. Chem. Soc.* **146**, 7979–7984 (2024).

[86] S. Senn, J.-M. Mörsdorf, M.-S. Bertrams, C. Kerzig, J. Ballmann, π Extended Diphosphonium Bridged Ladder Stilbenes: Water Soluble Fluorophores with up to Eight Annulated Rings. *Chem. Sci.* **16**, 20517–20526 (2025).

[87] D. Balcells, O. Eisenstein, M. Tilset, A. Nova, Coordination and insertion of alkenes and alkynes in Au(III) complexes: nature of the intermediates from a computational perspective. *Dalton Trans.* **45**, 5504–5513 (2016).

[88] R. Stegmann, A. Neuhaus, G. Frenking, Theoretical Studies of Organometallic Compounds. 5. Alkyne and Vinylidene Complexes of Molybdenum and Tungsten in High-Oxidation States. *J. Am. Chem. Soc.* **115**, 11930-11938 (1993).

[89] K. Tatsumi, R. Hoffmann, J. L. Templeton, Structural and theoretical evidence for participation of the second acetylene π-orbital in transition-metal alkyne complexes. *Inorg. Chem.* **21**, 466–468 (1982).

[90] J. L. Templeton, B. C. Ward, Carbon-13 chemical shifts of alkyne ligands as variable electron donors in monomeric molybdenum and tungsten complexes. *J. Am. Chem. Soc.* **102**, 3288–3290 (1980).

[91] J. L. Templeton, Four-electron alkyne ligands in molybdenum(II) and tungsten(II) complexes. *Adv. Organomet. Chem.* **29**, (1980).

[92] J. J. Carbó, P. Crochet, M. A. Esteruelas, Y. Jean, A. Lledós, A. M. López, E. Oñate, Two- and four-electron alkyne ligands in osmium-cyclopentadienyl chemistry: consequences of the $\pi_\perp \rightarrow M$ interaction. *Organometallics* **21**, 305–314 (2002).

[93] H. Nuss, N. Claiser, S. Pillet, N. Lugan, E. Despagnet-Ayoub, M. Etienne, C. Lecomte, A comparative study of the topology of the experimental electron density within 2- and 4-electron donor alkyne complexes. *Dalton Trans.* **41**, 6598 (2012).

[94] M. Llunell, D. Casanova, J. Cirera, P. Alemany, S. Alvarez. SHAPE Program for the Stereochemical Analysis of Molecular Fragments by Means of Continuous Shape Measures and Associated Tools. 2013, version 2.1., http://www.ee.ub.edu/index.php?option=com_jdownloads&-Itemid=529&view=viewcategory&catid=4.

[95] M. Pinsky, D. Avnir, Continuous symmetry measures. 5. The classical polyhedra. *Inorg. Chem.*, **37**, 5575-5582 (1998).

[96] S. Alvarez, D. Avnir, M. Llunell, M. Pinsky. Continuous symmetry maps and shape classification. The case of six-coordinated metal compounds. *New J. Chem.*, **26**, 996 (2002).

[97] D. Casanova, P. Alemany, J. M. Bofill, S. Alvarez. Shape and Symmetry of Heptacoordinate Transition ‐ Metal Complexes: Structural Trends. *Chem. Eur. J.*, **9**, 1281 (2003).

[98] A. J. W. Thom, E. J. Sundstrom, M. Head-Gordon, LOBA: a localized orbital bonding analysis to calculate oxidation states, with application to a model water-oxidation catalyst. *Phys. Chem. Chem. Phys.* **11**, 11297–11304 (2009).

[99] M. Gimferrer, J. Van der Mynsbrugge, A. T. Bell, P. Salvador, M. Head-Gordon, Facing the challenges of borderline oxidation-state assignments using state-of-the-art computational methods. *Inorg. Chem.* **59**, 15410–15420 (2020).

[100] I. F. Leach, J. E. M. N. Klein, Oxidation states: intrinsically ambiguous? *ACS Cent. Sci.* **10**, 1406–1414 (2024).

[101] T. P. Curran, A. L. Grant, R. A. Lucht, J. C. Carter, J. Affonso, π-Ligands for generating transition-metal–peptide complexes: coordination of amino acid derivatives to tungsten utilizing alkyne ligands. *Org. Lett.* **4**, 2917–2920 (2002).

[102] J. Baldas, J. F. Boas, J. Bonnyman, G. A. Williams, Studies of technetium complexes. Part 6. The preparation, characterisation, and electron spin resonance spectra of salts of tetrachloro- and tetrabromo-nitridotechnetate(VI): crystal structure of tetraphenylarsonium tetrachloronitridotechnetate(VI). *J. Chem. Soc., Dalton Trans.* **1984**, 2395–2400 (1984).

The following references are only cited in the Supplementary Materials.

[103] U. Mazzi, G. de Paoli, P. di Bernardo, L. Magon, Complexes of technetium(IV) and (III) with tertiary phosphines. *J. Inorg. Nucl. Chem.* **38**, 721–725 (1976).

[104] L. Krause, R. Herbst-Irmer, G. M. Sheldrick, D. Stalke, Comparison of silver and molybdenum microfocus X-ray sources for single-crystal structure determination. *Appl. Cryst.* **48**, 3–10 (2015).

[105] G. M. Sheldrick, SHELXT – integrated space-group and crystal-structure determination. *Acta Cryst.* **A71**, 3–8 (2015).

[106] G. M. Sheldrick, Crystal structure refinement with SHELXL. *Acta Cryst.* **C71**, 3–8 (2015).

[107] APEX, APEX2, SMART, SAINT, SAINT-Plus. Bruker AXS Inc., Madison, Wisconsin (USA), (2007).

[108] C. F. Macrae, I. Sovago, S. J. Cottrell, P. T. A. Galek, P. McCabe, E. Pidcock, M. Platings, G. P. Shields, J. S. Stevens, M. Towler, P. A. Wood, Mercury 4.0: from visualization to analysis, design and prediction. *J. Appl. Cryst.* **53**, 226–235 (2020).

[109] O. V. Dolomanov, L. J. Bourhis, R. J. Gildea, J. A. K. Howard, H. Puschmann, OLEX2: a complete structure solution, refinement and analysis program. *J. Appl. Cryst.* **42**, 339-341 (2009).

[110] High performance computing (HPC) system Curta at Freie Universität Berlin.

[111] M. J. Frisch, G. W. Trucks, H. B. Schlegel, G. E. Scuseria, M. A. Robb, J. R. Cheeseman, G. Scalmani, V. Barone, G. A. Petersson, H. Nakatsuji, X. Li, M. Caricato, A. V. Marenich, J. Bloino, B. G. Janesko, R. Gomperts, B. Mennucci, H. P. Hratchian, J. V. Ortiz, A. F. Izmaylov, J. L. Sonnenberg, D. Williams-Young, F. Ding, F. Lipparini, F. Egidi, J. Goings, B. Peng, A. Petrone, T. Henderson, D. Ranasinghe, V. G. Zakrzewski, J. Gao, N. Rega, G. Zheng, W. Liang, M. Hada, M. Ehara, K. Toyota, R. Fukuda, J. Hasegawa, M. Ishida, T. Nakajima, Y. Honda, O. Kitao, H. Nakai, T. Vreven, K. Throssell, J. A. Montgomery Jr., J. E. Peralta, F. Ogliaro, M. J. Bearpark, J. J. Heyd, E. N. Brothers, K. N. Kudin, V. N. Staroverov, T. A. Keith, R. Kobayashi, J. Normand, K. Raghavachari, A. P. Rendell, J. C. Burant, S. S. Iyengar, J. Tomasi, M. Cossi, J. M. Millam, M. Klene, C. Adamo, R. Cammi, J. W. Ochterski, R. L. Martin, K. Morokuma, O. Farkas, J. B. Foresman, D. J. Fox, Gaussian 16, Revision B.01, Gaussian, Inc., Wallingford CT (2016).

[112] R. Dennington, T. A. Keith, J. M. Millam, GaussView, Version 6, Semichem Inc., Shawnee Mission, KS (2016).

[113] M. D. Hanwell, D. E. Curtis, D. C. Lonie, C. Vandermeersch, E. Zurek, G. R. Hutchison, Avogadro: an advanced semantic chemical editor, visualization, and analysis platform. *J. Cheminform.* **4**, 1–17 (2012).

[114] S. Grimme, S. Ehrlich, L. Goerigk, Effect of the damping function in dispersion-corrected density functional theory. *J. Comput. Chem.* **32**, 1456–1465 (2011).

[115] S. H. Vosko, L. Wilk, M. Nusair, Accurate spin-dependent electron liquid correlation energies for local spin density calculations: a critical analysis. *Can. J. Phys.* **58**, 1200–1211 (1980).

[116] A. D. Becke, Density-functional thermochemistry. III. The role of exact exchange. *J. Chem. Phys.* **98**, 5648–5652 (1993).

[117] C. Lee, W. Yang, R. G. Parr, Development of the Colle-Salvetti correlation-energy formula into a functional of the electron density. *Phys. Rev. B* **37**, 785–789 (1988).

[118] F. Weigend, R. Ahlrichs, Balanced basis sets of split valence, triple zeta valence and quadruple zeta valence quality for H to Rn: Design and assessment of accuracy. *Phys. Chem. Chem. Phys.* **7**, 3297–3305 (2005).

[119] K. A. Peterson, D. Figgen, E. Goll, H. Stoll, M. Dolg, Systematically convergent basis sets with relativistic pseudopotentials. II. Small-core pseudopotentials and correlation consistent basis sets for the post-d group 16–18 elements. *J. Chem. Phys.* **119**, 11113–11123 (2003).

[120] B. Metz, H. Stoll, M. Dolg, Small-core multiconfiguration-Dirac–Hartree–Fock-adjusted pseudopotentials for post-d main group elements: Application to PbH and PbO. *J. Chem. Phys.* **113**, 2563–2569 (2000).

[121] K. L. Schuchardt, B. T. Didier, T. Elsethagen, L. Sun, V. Gurumoorthi, J. Chase, J. Li, T. L. Windus, Basis Set Exchange: A Community Database for Computational Sciences. *J. Chem. Inf. Model.* **47**, 1045–1052 (2007).

[122] T. Lu, F. Chen, Multiwfn: A multifunctional wavefunction analyzer, *J. Comput. Chem.* **33**, 580–592 (2012).

[123] B. G. Janesko, K. B. Wiberg, G. Scalmani, M. J. Frisch, Electron delocalization range in atoms and on molecular surfaces. *J. Chem. Theory Comput.* **12**, 3185–3194 (2016).

[124] B. G. Janesko, G. Scalmani, M. J. Frisch, Quantifying solvated electrons' delocalization. *Phys. Chem. Chem. Phys.* **17**, 18305–18317 (2015).

[125] T. Liu, Q. Chen, Interaction region indicator: a simple real-space function clearly revealing both chemical bonds and weak interactions. *Chemistry – Methods* **1**, 231 (2021).

[126] R. F W. Bader, *Atoms in Molecules: A Quantum Theory*; Clarendon, Oxford, U.K. (1990).

[127] R. Hilal, S. G. Aziz, A. O. Alyoubi, S. Elroby, Quantum topology of the charge density of chemical bonds. QTAIM analysis of the C-Br and O-Br bonds. *Procedia Comput. Sci.*, **51**, 1872-1877, (2015).

[128] C. S. López, A. R. de Lera, Bond ellipticity as a measure of electron delocalization in structure and reactivity. *Curr. Org. Chem*., **15**, 3576-3593 (2011).

[129] L. Zhang, F. Ying, W. Wu, P. C. Hiberty, S. Shaik, Topology of Electron Charge Density for Chemical Bonds from Valence Bond Theory: A Probe of Bonding Types. *Chem. Eur. J*., **15**, 2979-2989 (2009).

[130] W. Wang, B. Ji, Y Zhang, Chalcogen bond: a sister noncovalent bond to halogen bond. *J. Phys. Chem. A,* **113***,* 8132–8135 (2009).

[131] Cabeza, J. A.; van der Maelen, J. F.; García-Granda, S. Topological Analysis of the Electron Density in the N-Heterocyclic Carbene Triruthenium Cluster [Ru_3(μ-H)$_2$(μ^3-MeImCH)$(CO)_9$] (Me2Im = 1,3-dimethylimidazol-2-ylidene). *Organometallics*, **28**, 3666–3672 (2009) and references cited therein.

[132] E. Matito, M. Solà, The role of electronic delocalization in transition metal complexes from the electron localization function and the quantum theory of atoms in molecules viewpoints. *Coord. Chem. Rev*., **253**, 647–665 (2009) and references cited therein.

[133] E. Matito, J. Poater, F. M. Bickelhaupt, M. Solà, Bonding in Methylalkalimetals $(CH_3M)_n$ (M = Li, Na, K; n = 1, 4). Agreement and Divergences between AIM and ELF Analyses. *J. Phys. Chem. B*, **110***,* 7189-7198 (2006).

[134] W. Humphrey, A. Dalke, K. Schulten, VMD: Visual molecular dynamics. *J. Molec. Graphics*, **14**, 33-38 (1996).

[135] F. Neese, Software update: the ORCA program system, version 6.0. WIREs *Comput. Mol. Sci.*, **15**, e70019 (2025).

[136] D. Ganyushin, F. Neese, First-principles calculations of zero-field splitting parameters. *J. Chem. Phys*., **125**, 024103 (2006). https://doi.org/10.1063/1.2213976

[137] G. L. Stoychev, A. A. Auer, F. Neese, Automatic Generation of Auxiliary Basis Sets. *J. Theor. Comput. Chem.*, **13**, 554–562 (2017).

[138] C. Kollmar, K. Sivalingam, B. Helmich-Paris, C. Angeli, F. Neese, A perturbation-based super-CI approach for the orbital optimization of a CASSCF wave function. J*. Comput. Chem*., **40**, 1463–1470 (2019).

[139] L. Lang, F. Neese, Spin-dependent properties in the framework of the dynamic correlation dressed complete active space method. *J. Chem. Phys*., **150**, 104104 (2019).

[140] F. Neese, The SHARK Integral Generation and Digestion System. *J. Comput. Chem.*, **44**, 381 (2022).

[141] M. Ugandi, M. Roemelt, A recursive formulation of one-electron coupling coefficients for spin-adapted configuration interaction calculations featuring many unpaired electrons. *Int. J. Quantum Chem*., **123**, e27045 (2023).

[142] F. Neese, The ORCA program system. WIREs *Comput. Mol. Sci.*, **2**, 73–78 (2012).

[143] F. Neese, Software update: the ORCA program system, version 4.0. WIREs *Comput. Mol. Sci*., **8**, 1–6 (2018).

[144] F. Neese, F. Wennmohs, U. Becker, C. Riplinger, The ORCA quantum chemistry program package. *J. Chem. Phys*., **152**, 224108 (2020).

[145] F. Neese, Software update: The ORCA program system—Version 5.0. WIREs *Comput. Mol. Sci.*, **12**, e1606 (2022).

[146] F. Neese, Approximate second-order SCF convergence for spin unrestricted wavefunctions. *Chem. Phys. Lett*., **325**, 93–98 (2000).

[147] G. Knizia, Intrinsic atomic orbitals: An unbiased bridge between quantum theory and chemical concepts. *J. Chem. Theory Comput*., **9**, 4834 (2013).

[148] G. Knizia, J.E.M.N. Klein, Electron flow in reaction mechanisms --- revealed from first principles. *Angew. Chem. Int. Ed.*, **54**, 5518 (2015).

[149] D. Andrae, U. Häußermann, M. Dolg, H. Stoll, H. Preuß, Energy-adjusted ab initio pseudopotentials for the second and third row transition elements. *Theor. Chim. Acta*, **77**, 123–141 (1990).

[150] F. Weigend, F. Furche, R. Ahlrichs, Gaussian basis sets of quadruple-zeta valence quality for atoms H–Kr. *J. Chem. Phys.*, **119**, 12753–12762 (2003).
